# Competitive binding of E3 ligases TRIM26 and WWP2 controls SOX2 in glioblastoma

**DOI:** 10.1038/s41467-021-26653-6

**Published:** 2021-11-03

**Authors:** Tatenda Mahlokozera, Bhuvic Patel, Hao Chen, Patrick Desouza, Xuan Qu, Diane D. Mao, Daniel Hafez, Wei Yang, Rukayat Taiwo, Mounica Paturu, Afshin Salehi, Amit D. Gujar, Gavin P. Dunn, Nima Mosammaparast, Allegra A. Petti, Hiroko Yano, Albert H. Kim

**Affiliations:** 1 Department of Neurological Surgery, Washington University School of Medicine, St. Louis, MO USA; 2 Department of Neurology, Washington University School of Medicine, St. Louis, MO USA; 3 Department of Pathology and Immunology, Washington University School of Medicine, St. Louis, MO USA; 4 The Brain Tumor Center, Siteman Cancer Center, Washington University School of Medicine, St. Louis, MO USA; 5 Department of Genetics, Washington University School of Medicine, St. Louis, MO USA; 6 Department of Developmental Biology, Washington University School of Medicine, St. Louis, MO USA

**Keywords:** Ubiquitylation, Cancer stem cells

## Abstract

The pluripotency transcription factor SOX2 is essential for the maintenance of glioblastoma stem cells (GSC), which are thought to underlie tumor growth, treatment resistance, and recurrence. To understand how SOX2 is regulated in GSCs, we utilized a proteomic approach and identified the E3 ubiquitin ligase TRIM26 as a direct SOX2-interacting protein. Unexpectedly, we found TRIM26 depletion decreased SOX2 protein levels and increased SOX2 polyubiquitination in patient-derived GSCs, suggesting TRIM26 promotes SOX2 protein stability. Accordingly, TRIM26 knockdown disrupted the SOX2 gene network and inhibited both self-renewal capacity as well as in vivo tumorigenicity in multiple GSC lines. Mechanistically, we found TRIM26, via its C-terminal PRYSPRY domain, but independent of its RING domain, stabilizes SOX2 protein by directly inhibiting the interaction of SOX2 with WWP2, which we identify as a *bona fide* SOX2 E3 ligase in GSCs. Our work identifies E3 ligase competition as a critical mechanism of SOX2 regulation, with functional consequences for GSC identity and maintenance.

## Abstract (web summary)

SOX2 is required for the maintenance of glioblastoma stem cells (GSCs). Here the authors identify that the RING family E3 ubiquitin ligase TRIM26 promotes SOX2 stability in a non-canonical ligase-independent manner and thus, increases the tumorigenicity of GSCs.

## Introduction

Despite advances in surgery and chemoradiation therapies, the prognosis of the brain cancer glioblastoma (GBM) remains challenging^1–3^. In addition to the blood–brain barrier, therapy resistance in glioblastoma has been attributed to the intrinsic heterogeneity of these tumors. Beyond genetic heterogeneity, GBM tumors also exhibit appreciable epigenetic and cellular heterogeneity. One particular GBM cell type, which has been the focus of an intense investigation, is the brain tumor-initiating cell, also known as the glioblastoma stem cell (GSC)^4–8^. GSCs are thought to be therapy-resistant and therefore drive tumor recurrence posttreatment^6,9^.

The core pluripotency-related transcription factor SRY (sex-determining region Y)-box 2 (SOX2) is highly expressed in glioblastoma relative to the normal brain and indispensable for key GSC phenotypes, including self-renewal and tumor initiation^10–13^. We have previously shown that proteasomal regulation of SOX2 is critical to the maintenance and function of GSCs^13^, but the major regulators of this biochemical process in these cells—and in particular the identity of the E3 ligase that targets SOX2 for degradation—remains to be elucidated.

Herein, we report that precise regulation of SOX2 protein turnover is a shared feature of primary patient-derived GSCs, highlighting this phenomenon as a potential common therapeutic target. We identify the E3 ubiquitin ligase TRIM26 as a SOX2-interacting protein and show that TRIM26 surprisingly protects SOX2 from polyubiquitination and proteasomal degradation through a noncanonical ligase-independent mechanism. Accordingly, we find that TRIM26 regulates GSC self-renewal in a SOX2-dependent manner and is required for GSC tumorigenicity. We then identify WWP2 as a *bona fide* E3 ubiquitin ligase that targets SOX2 for proteasomal degradation in GSCs in vitro and glioblastoma cells in vivo. Finally, we elucidate the mechanism by which SOX2 is protected by TRIM26, which competes with WWP2 for binding to SOX2, resulting in inhibition of WWP2-mediated SOX2 polyubiquitination and subsequent proteasomal degradation. These results highlight that regulation of SOX2 at the posttranslational level is essential for the maintenance of GSC identity and biologic phenotypes.

## Results

### TRIM26 is a SOX2-interacting E3 ubiquitin ligase in GSCs

**Fig. 1:**
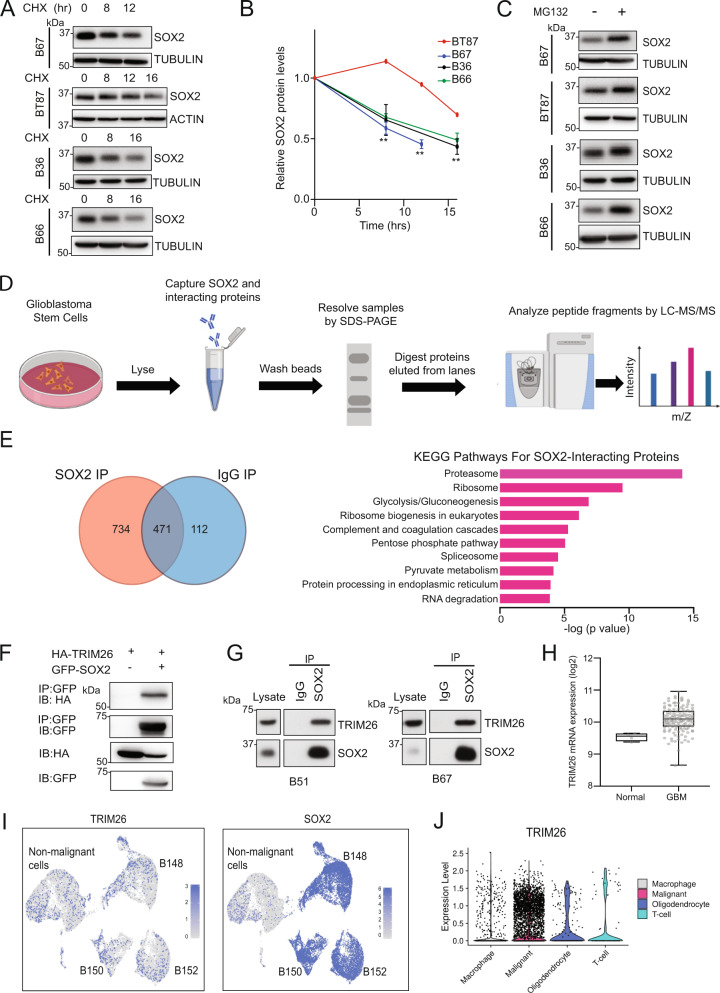
TRIM26 E3 ubiquitin ligase is a SOX2-interacting protein in GSCs. **A** GSC lysates treated with 100 μM cycloheximide (CHX) were subjected to immunoblotting. Data were representative of three independent experiments for B67, B36, and B66 and of two for BT87. **B** Quantification of protein expression levels for cycloheximide chase experiments in (**A**). Data represent mean ±  SEM (*n* = 3 for B67, B36, and B66. *n* = 2 for BT87. Unpaired *t*-test, **P < 0.005 compared to 0 h). See Source Data File for exact *P* values and statistical parameters. **C** GSCs were treated with vehicle (DMSO) or 10 μM MG132 for 8 h. Lysates were subjected to immunoblotting. Data were representative of three independent experiments. **D** Schematic workflow for SOX2 immunoprecipitation from B36 GSC lysates followed by mass spectrometry (SOX2 IP-MS) to identify SOX2 interactors. **E** SOX2 IP-MS. (Left) 734 SOX2 interactors were identified. (Right) KEGG analysis for interactors was performed using Enrichr^47^. **F** HEK293T cells were transfected with indicated DNA plasmids. Lysates were subjected to anti-GFP immunoprecipitation (IP) and immunoblotting (IB). Data were representative of three independent experiments. **G** GSC lysates were subjected to anti-SOX2 immunoprecipitation. Input lysates and IP samples were subjected to immunoblotting. Data were representative of three independent experiments. **H** Box plots for TRIM26 mRNA expression in TCGA *IDH1/2* wild-type glioblastoma (*n* = 142) and normal brain tissue (*n* = 4) based on RNA-seq platform (Unpaired *t*-test, *P* = 0.0076). The boxes represent the 25th percentile, median, and 75th percentile values. The whiskers represent the maximum and minimum values for the plotted data See also Supplementary Fig. 1A. See Source Data File for statistical parameters. **I** Patient tumors were subjected to single-cell RNA-sequencing (scRNA-seq). Malignant cells were identified by expression-based inference of copy number alterations. Cells are colored by expression levels for TRIM26 (left) and SOX2 (right). See also Supplementary Fig. 2. **J** An independent scRNA-seq dataset (Neftel et al, *Cell*, 2019)^27^ was examined for TRIM26 expression in 28 glioblastomas and plotted as a violin plot. Smart-seq2 data were downloaded with cell type annotations. (Malignant: *n* = 6863, Macrophage: *n* = 754, Oligodendrocyte: *n* = 219, T-cell: *n* = 94; two-sided *t*-test (malignant vs. nonmalignant), *P* = 0.002662).

We have previously demonstrated that SOX2 is controlled at the protein level in GSCs^13^. However, the precise regulation of SOX2 proteostasis in GSCs has not been well-characterized, and a SOX2-specific E3 ubiquitin ligase in the GSC context has not yet been identified. To assess the ambient regulation of SOX2 proteostasis in GSCs, we first performed a cycloheximide chase experiment in four patient-derived, low passage GSC lines. Pharmacologic inhibition of *de novo* protein synthesis using cycloheximide (100 μM) in GSCs led to a time-dependent decrease in SOX2 protein levels (Fig. 1A, B). In addition, exposure of GSCs to proteasome inhibitor MG132 (10 μM) resulted in increased SOX2 protein levels compared to vehicle (Fig. 1C), indicating active ongoing turnover through ubiquitin-mediated degradation.

Given the dynamic regulation of SOX2 protein stability in GSCs, we sought to identify key regulators of SOX2 proteostasis using an unbiased proteomic approach. GSC protein lysates were subjected to immunoprecipitation (IP) with either anti-SOX2 or control IgG antibody. Immunoprecipitates were resolved by SDS-PAGE, digested, and subjected to liquid chromatography coupled with mass spectrometry (MS) analysis (Fig. 1D). Overall, we identified 734 potential SOX2-interacting proteins, including known SOX2 interactors (Supplementary Data 1)^14^. KEGG pathway analysis of these proteins revealed the proteasome as the top enriched biological process, further highlighting the significance of protein homeostasis mechanisms in the regulation of SOX2 (Fig. 1E). To identify E3 ubiquitin ligases that interact with SOX2 in GSCs, we intersected the SOX2 interactors identified in the IP-MS experiment with a comprehensive list of predicted human E3 ubiquitin ligases^15^. We identified ten predicted E3 ligases that might be candidate SOX2 interactors (Supplementary Table 1), of which five (ITCH, KLHL22, TRIM26, TRIP12, and TTC3) have established functions as E3 ubiquitin ligases in the ubiquitin-proteasome system^16–20^.

To validate the IP-MS results, we first performed co-IP experiments in a heterologous system. We focused on the TRIM26 E3 ubiquitin ligase, which has well-characterized immune-related functions and has also been associated with pluripotency in other cell contexts^14,17,21,22^. TRIM26 was also identified in a discovery set of SOX2-interacting proteins in a medulloblastoma cell line, but the interaction has not yet been validated or functionally examined^14,17,22^. Epitope-tagged SOX2 and TRIM26 were found in a complex by immunoprecipitation in transiently transfected HEK293T cells (Fig. 1F). To further validate this interaction in the relevant context, we found that endogenous SOX2 and endogenous TRIM26 proteins interact in two distinct primary GSC lines (Fig. 1G), together suggesting that TRIM26 is poised to impact SOX2 function. Interrogation of the TCGA database confirmed that TRIM26 is highly expressed in GBM tumor specimens relative to normal brain (Fig. 1H and Supplementary Fig. 1). To verify that TRIM26 is expressed in GBM tumor cells, we subjected freshly dissociated tumor samples from three patients to single-cell RNA-sequencing (scRNA-seq). Malignant cells were identified based on the presence of copy number-altered genomic loci, including chr7p, chr7q, chr10p, chr10q, and chr22q, which are frequently observed in GBM tumors, and were inferred using the CONICSmat tool (Supplementary Fig. 2, Methods). TRIM26 was detectable in malignant cells from all three tumor samples (Fig. 1I). As described previously, SOX2 was expressed by the majority (80%) of malignant cells across all three tumors (Fig. 1I)^12,23^. To provide further validation for TRIM26 expression in malignant cells, we used an independent, published scRNA-seq dataset of 28 GBM tumors, which demonstrated that TRIM26 is more highly expressed in GBM tumor cells compared to nonmalignant cells (Fig. 1J).

### TRIM26 promotes SOX2 protein stability and regulates genes downstream of SOX2

**Fig. 2:**
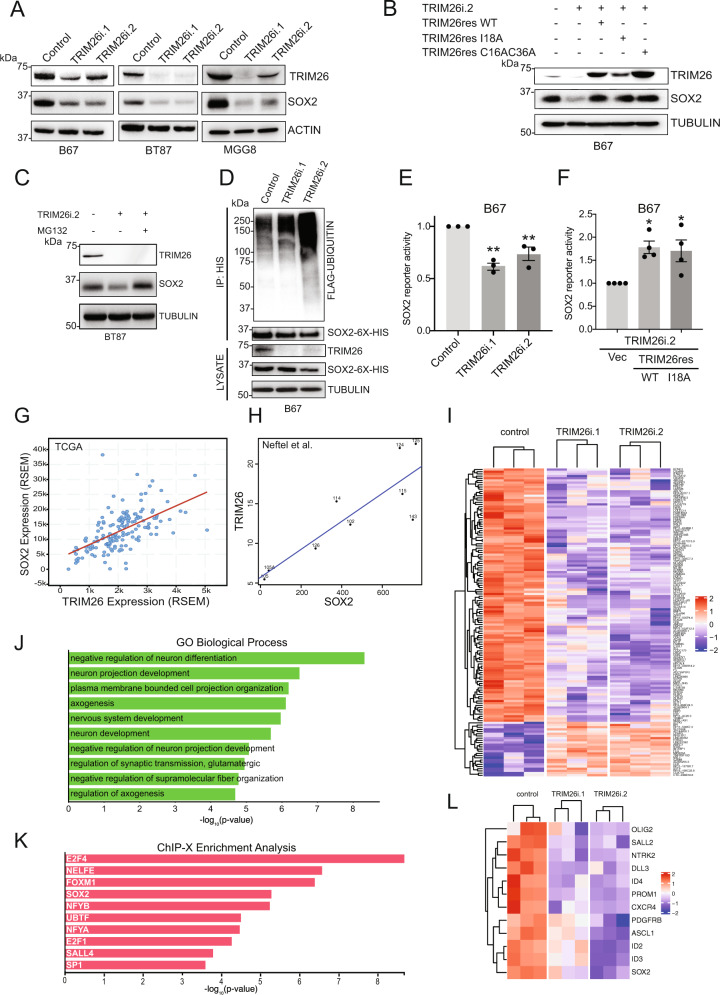
TRIM26 promotes SOX2 protein stability and transcriptional activity in GSCs. **A** GSCs were transduced with TRIM26 RNAi or control. Five days later, lysates were analyzed by immunoblotting. Data were representative of three independent experiments. **B** GSCs transduced with TRIM26 RNAi or control were transduced with either vector, wild-type (WT) TRIM26res, or RING domain-mutant TRIM26res (I18A and C16AC36A) lentiviruses. Four days later, lysates were subjected to immunoblotting. Data were representative of three independent experiments. **C** GSCs transduced with TRIM26 RNAi or control were treated 5 days later with vehicle (DMSO) or 10 μM MG132 for 8 h. Lysates were subjected to immunoblotting. Data were representative of three independent experiments. **D** GSCs stably expressing histidine-tagged SOX2 and FLAG-tagged ubiquitin were transduced with TRIM26 RNAi or control. Lysates were subjected to immunoprecipitation (IP) under denaturing conditions using nickel-NTA beads. IP and input samples were analyzed by immunoblotting. Data were representative of three independent experiments. **E** GSCs transduced with Firefly luciferase-based SOX2 reporter and Renilla luciferase lentiviruses were transduced with TRIM26 RNAi or control. Five days later, luciferase activities were measured. Firefly activity was divided by Renilla activity for normalization. Data represent mean ±  SEM (*n* = 3, ANOVA, **P < 0.01). See Source Data File for exact *P* values and statistical parameters. See also Supplementary Fig. 4A. **F** GSCs stably transduced with luciferase-based SOX2 reporter lentiviruses were measured as in (**E**). Luciferase values were divided by protein concentration for normalization. Data represent mean ±  SEM (*n* = 3, ANOVA, *P < 0.03). See Source Data File for exact *P* values and statistical parameters. **G** Correlation analysis for TRIM26 and SOX2 mRNA in TCGA glioblastoma RNA-seq dataset (*n* = 141, Pearson *r* = 0.59, *P* < 0.0001). **H** An independent scRNA-seq dataset (10X data, Neftel et al, *Cell*, 2019)^27^ was used to derive pseudobulk expression values for TRIM26 and SOX2 only in tumor cells for correlation analysis (*n* = 9 tumors, linear regression, *R*^2^ = 0.69, *P* = 0.00330). See also Supplementary Fig. 4C. **I** B67 GSCs transduced with TRIM26 RNAi or control were harvested for total RNA and subjected to RNA-seq. The top 141 significantly differentially expressed (compared to control), directionally consistent genes between the two TRIM26 RNAi are plotted as a heatmap (*n* = 3 per condition, FDR <0.05). See Source Data for data points. See also Supplementary Table 3. **J** Gene Ontology (GO) analysis was performed using significantly downregulated genes (FDR <0.05) shared by both TRIM26 RNAi (compared to control) from (**I**) using Enrichr^47^. The top ten GO terms related to the biological process are shown. **K** Chromatin-immunoprecipitation-X (ChIP-X) enrichment analysis was performed using all significantly differentially regulated genes (FDR <0.05) shared by both TRIM26 RNAi from (**I**) using X2K^48^. The top ten transcription factors with enriched target genes are shown. **L** Significantly differentially regulated (FDR <0.05) and directionally consistent genes shared by both TRIM26 RNAi (compared to control) were analyzed for expression of 27 GSC marker genes, and significant genes (FDR <0.05) shown by heatmap. See Source Data for data points. See also Supplementary Fig. 4D.

To investigate the potential biochemical role of TRIM26 in regulating SOX2, we subjected GSCs to RNA interference (RNAi)-mediated TRIM26 knockdown. Unexpectedly, TRIM26 knockdown decreased SOX2 protein levels in three distinct GSC lines (Fig. 2A), suggesting that TRIM26 does not promote SOX2 degradation but instead may promote SOX2 stability. We then performed structure-function studies to delineate the mechanism by which TRIM26 regulates SOX2 in GSCs. We first introduced synonymous mutations in wild-type TRIM26 to generate a TRIM26 rescue cDNA (TRIM26res) insensitive to RNAi. In the context of this rescue mutant, we then generated two catalytically inactive mutants of TRIM26—TRIM26-I18A and TRIM26 C16AC36A^17^—both of which disrupt the zinc finger RING domain. Intriguingly, lentiviral expression of each of these three rescue mutants in the setting of TRIM26 RNAi was sufficient to rescue SOX2 protein levels in GSCs (Fig. 2B), suggesting that the catalytic activity of TRIM26 is dispensable for SOX2 regulation. To further characterize the mechanism by which this regulation occurs, we tested the effect of TRIM26 knockdown on exogenous, epitope-tagged SOX2 driven by an elongation factor 1-alpha promoter in GSCs (Supplementary Fig. 3A). TRIM26 knockdown also decreased levels of exogenous SOX2, suggesting TRIM26-dependent regulation of SOX2 at the posttranscriptional level. Indeed, the decrease in endogenous SOX2 protein levels triggered by TRIM26 knockdown could be reversed by treatment with proteasome inhibitor MG132 (Fig. 2C), and cycloheximide chase experiments revealed that TRIM26 knockdown in GSCs increased the rate of SOX2 protein degradation relative to control (Supplementary Fig. 3B). Together, these results suggest that TRIM26 stabilizes SOX2 protein. Accordingly, we observed that TRIM26 knockdown resulted in a robust increase in SOX2 polyubiquitination in GSCs (Fig. 2D). Taken together, these observations indicate that TRIM26, in a manner independent of its E3 ligase function, regulates SOX2 proteostasis, specifically by protecting SOX2 from polyubiquitination and subsequent proteasomal degradation.

Consistent with the observation that TRIM26 promotes SOX2 protein stability, TRIM26 knockdown decreased SOX2-dependent transcription, as measured using an established SOX2-responsive reporter^13,24^ (Fig. 2E and Supplementary Fig. 4A). Expression of both wild-type and catalytically inactive (I18A) TRIM26 rescue mutants could restore SOX2 reporter activity in the setting of TRIM26 knockdown (Fig. 2F), in accordance with the SOX2 protein results (Fig. 2B). In further support of this positive relationship between TRIM26 and SOX2, in TCGA glioblastoma samples, we observed a significant correlation between TRIM26 and SOX2 mRNA expression levels (Fig. 2G), consistent with the ability of SOX2 to stimulate its own transcription^25,26^. Since TCGA data represent bulk tumor RNA and are thus not specific for malignant cells, we leveraged the independent scRNA-seq dataset of GBMs (as in Fig. 1J) and examined the expression of TRIM26 and SOX2 in only malignant cells within each tumor^27^. This tumor cell-specific pseudobulk expression analysis also revealed a significant correlation between TRIM26 and SOX2 expression with a trend towards correlation in a second scRNA-seq dataset (Fig. 2H and Supplementary Fig. 4C).

To investigate the downstream consequences of TRIM26 loss in GSCs, we performed RNA-sequencing in TRIM26 knockdown GSCs vs. control cells (Supplementary Data 2 and Fig. 2I). The two TRIM26 shRNAs commonly and significantly altered the expression of 2835 genes, with 1224 downregulated and 1054 upregulated genes. Based on the gene set downregulated by TRIM26 RNAi, Gene Ontology analysis demonstrated enrichment of biological processes related to nervous system development, with “negative regulation of neuron differentiation” the most enriched process (Fig. 2J). These data suggested that TRIM26 might promote a progenitor or stem cell-like phenotype in GSCs, consistent with the developmentally aberrant GSC state and also with SOX2’s known role in early neural development. Indeed, chromatin-immunoprecipitation-X enrichment analysis (ChEA) to infer over-represented transcription factor targets in the TRIM26 knockdown-associated gene network demonstrated that SOX2 was among the most significant upstream transcription factors (Fig. 2K), in agreement with the biochemical effects of TRIM26 on SOX2 stability and reporter activity. Furthermore, analysis of known GSC markers in the RNA-seq dataset revealed that TRIM26 RNAi significantly downregulated several GSC marker genes, overall suggesting that TRIM26 plays a role in the maintenance of the GSC state (Fig. 2L and Supplementary Fig. 4D).

### TRIM26 regulates GSC self-renewal via its C-terminal PRYSPRY domain

**Fig. 3:**
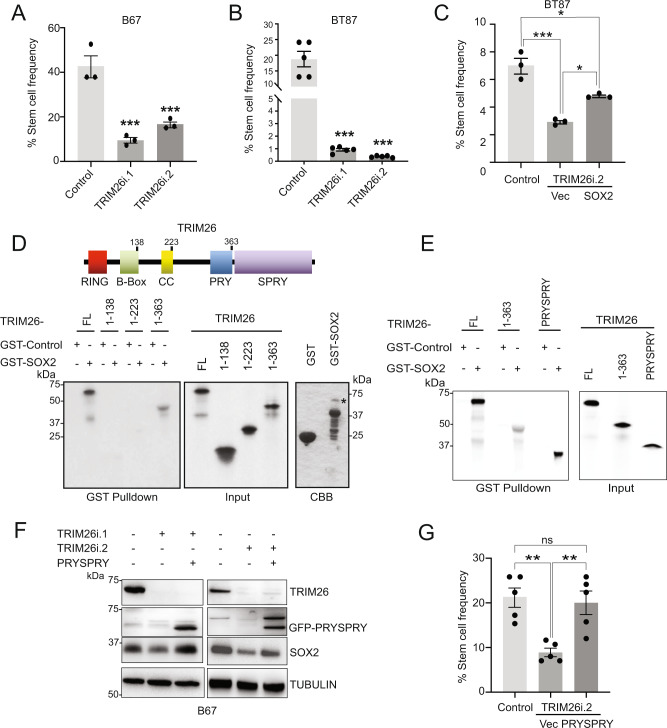
TRIM26 regulates GSC self-renewal via its C-terminal PRYSPRY domain. **A** GSCs were transduced with TRIM26 RNAi or control and subjected to the extreme limiting dilution assay (ELDA). Data represent mean ±  SEM (*n* = 3, ANOVA, ***P < 0.005). See Source Data File for exact *P* values and statistical parameters. **B** GSCs treated as in (**A**) were subjected to ELDA. Data represent mean ±  SEM (*n* = 5, ANOVA, ***P < 0.0001). See Source Data File for exact *P* values and statistical parameters. **C** GSCs transduced with TRIM26 RNAi or control were transduced 2 days later with SOX2-expressing or vector (Vec) lentiviruses and subjected to ELDA. Data represent mean ±  SEM (*n* = 3, ANOVA, *P < 0.05, ***P < 0.0005). See Source Data File for exact *P* values and statistical parameters. **D** In vitro translated, ^35^S-labeled full-length (FL) TRIM26 and domain deletion mutants were used in GST pulldown assays using glutathione beads and bacterially produced GST control or GST-SOX2 (*FL SOX2). TRIM26 proteins were visualized by gel electrophoresis and fluorography (middle panel: input). GST-fusion proteins were visualized by Coomassie brilliant blue (CBB) staining. Data were representative of three independent experiments. **E** In vitro translated, ^35^S-labeled full-length (FL) TRIM26, TRIM26 1-363, and TRIM26 PRYSPRY domain were used in GST pulldown assays with GST control or GST-SOX2 as in (**D**). Data were representative of three independent experiments. **F** GSCs transduced with TRIM26 RNAi or control were treated 2 days later with either vector or TRIM26 GFP-PRYSPRY-expressing lentiviruses. Four days later, lysates were subjected to immunoblotting. Data were representative of three independent experiments. **G** GSCs transduced with TRIM26 RNAi or control were transduced 2 days later with either vector (Vec) or TRIM26 GFP-PRYSPRY-expressing lentiviruses and then subjected to ELDA as in (**A**). Data represent mean ±  SEM (*n* = 5, ANOVA, **P < 0.01, ns nonsignificant). See Source Data File for exact *P* values and statistical parameters.

We next examined the role of TRIM26 in regulating GSC biology first by testing the effect of TRIM26 manipulation on GSC self-renewal, a phenotype associated with tumorigenic potential^11^. Using the extreme limiting dilution assay to measure the frequency of self-renewing cells, we found that TRIM26 RNAi reduced GSC self-renewal capacity in two different GSC lines (Fig. 3A, B). We then asked if TRIM26 functions through SOX2 to regulate GSC self-renewal. SOX2 overexpression restored 44% of the self-renewal capacity of TRIM26 knockdown GSCs (Fig. 3C and Supplementary Fig. 4E), suggesting that SOX2 is downstream of TRIM26 in the regulation of GSC self-renewal. Since self-renewal can be affected by other cellular processes, we also tested if TRIM26 regulates cell viability (Supplementary Fig. 4B). TRIM26 knockdown in two GSC lines moderately decreased cell viability as measured by a luminescence-based ATP assay. Together, these results indicate that TRIM26 promotes GSC self-renewal at least in part by maintaining cell viability.

To determine if the mechanism of SOX2 regulation by TRIM26 occurs through direct interaction, we first performed GST pulldown assays using in vitro synthesized TRIM26 and recombinant GST-SOX2 (Fig. 3D, E). Under these conditions, TRIM26 is directly bound to GST-SOX2 but not to GST control. We then sought to map the domain of TRIM26 that interacts with SOX2 and found that the RING, B-box, and Coiled-coil (CC) domains of TRIM26 were not required for the interaction with GST-SOX2 (Fig. 3D). Instead, we found that the C-terminal PRY subdomain and the PRYSPRY domain of TRIM26 were sufficient to bind SOX2, with PRYSPRY binding SOX2 more strongly (Fig. 3D, E). We further investigated the effects of the PRYSPRY domain since structural analyses of related PRYSPRY domains demonstrated the SPRY subdomain is cradled within the PRY subdomain^28^. Remarkably, overexpression of the TRIM26 PRYSPRY domain alone in the setting of TRIM26 RNAi was sufficient to rescue the decrease in SOX2 protein triggered by TRIM26 knockdown (Fig. 3F). Accordingly, expression of the TRIM26 PRYSPRY domain reversed the TRIM26 RNAi-induced decrease in GSC self-renewal capacity (Fig. 3G). Together, these data suggest that TRIM26 regulates GSC self-renewal by directly interacting with and stabilizing SOX2 protein via the TRIM26 C-terminal PRYSPRY domain.

### WWP2 is a SOX2 E3 ubiquitin ligase in GSCs

**Fig. 4:**
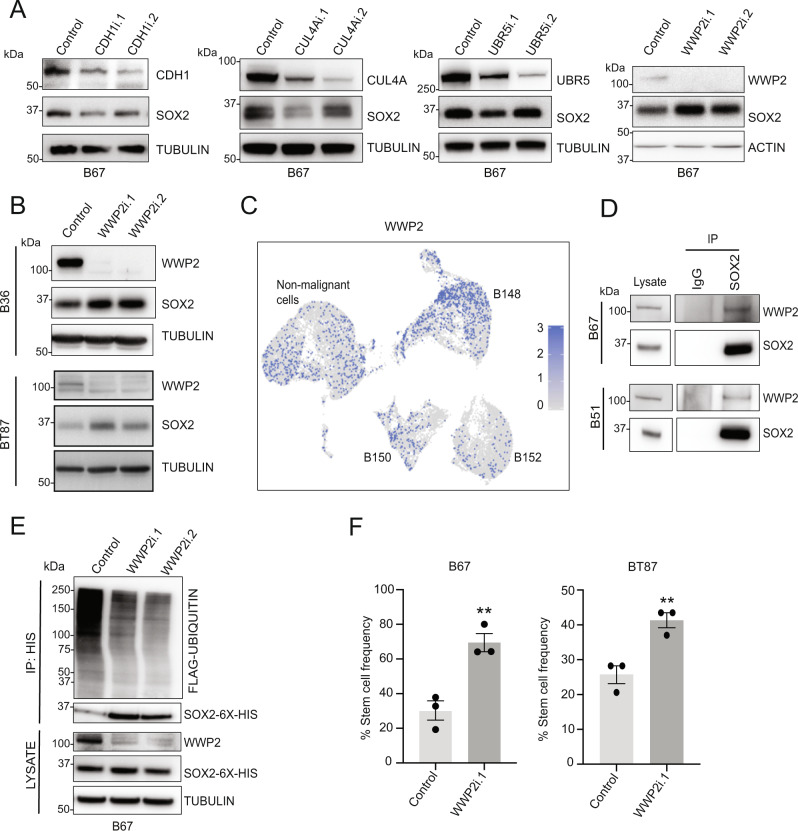
WWP2 is a *bona fide* SOX2 E3 ubiquitin ligase in GSCs. **A** GSCs transduced with control or RNAi targeting CDH1, CUL4A, UBR5, and WWP2 were harvested 6 days later and subjected to immunoblotting. Data were representative of three independent experiments. **B** GSCs transduced with WWP2 RNAi or control were harvested 6 days later and subjected to immunoblotting. Data were representative of three independent experiments. **C** Cells isolated from patient tumors were subjected to scRNA-seq. Malignant cells were identified by expression-based inference of copy number variation as in Fig. 1I. Cells are colored by expression level for WWP2. **D** GSC lysates were subjected to anti-SOX2 immunoprecipitation (IP). Input lysates and IP samples were subjected to immunoblotting. Data were representative of three independent experiments. **E** GSCs transduced with lentiviruses to stably express 6X-Histidine epitope-tagged SOX2 and FLAG epitope-tagged ubiquitin were transduced with WWP2 RNAi or control. Lysates were subjected to IP under denaturing conditions using nickel-NTA beads. IP and input samples were subjected to immunoblotting. Data were representative of three independent experiments. **F** GSCs transduced with WWP2 RNAi or control were subjected to ELDA as in Fig. 3A. Data represent mean ±  SEM (*n* = 3, unpaired *t*-test, **P < 0.01). See Source Data File for exact *P* values and statistical parameters.

Next, we sought to identify the downstream SOX2-modifying E3 ubiquitin ligase that is inhibited by the binding of TRIM26 to SOX2. Since no SOX2-targeting E3 ubiquitin ligase has yet been identified in glioblastoma cells, we tested the role of four E3 ubiquitin ligases (CDH1-APC, CUL4A, UBR5, and WWP2) which have been demonstrated to target SOX2 in other cellular contexts through an RNA interference-based approach in GSCs^29–32^. Among the four E3 ligases, only WWP2 knockdown was found to increase SOX2 protein levels in GSCs (Fig. 4A). The increase in ambient SOX2 protein triggered by WWP2 RNAi was further confirmed in two additional GSC lines (Fig. 4B). Interrogation of the TCGA database confirmed that WWP2 is expressed in glioblastoma patient tumors, although at lower levels compared to normal brain (Supplementary Fig. 5A, B). Examination of our scRNA-seq data from three GBM tumors revealed that WWP2 was detectable in malignant cells across all three samples (Fig. 4C).

To evaluate the biochemical role of WWP2 in regulating SOX2 in GSCs, we first performed IP experiments and found that endogenous WWP2 and SOX2 exist in a complex in two distinct GSC lines (Fig. 4D). To assess SOX2 polyubiquitination, we generated a GSC line that stably expresses FLAG-tagged Ubiquitin and histidine-tagged SOX2. WWP2 knockdown with two distinct RNAi led to a robust decrease in SOX2 polyubiquitination compared to control RNAi in GSCs (Fig. 4E). To test the specificity of the knockdown phenotype, we generated a rescue mutant of WWP2 (WWP2res) with synonymous mutations that render it insensitive to RNAi. Expression of WWP2res in the setting of WWP2 RNAi reversed the increase in SOX2 protein levels to nearly ambient levels (Supplementary Fig. 5C). In sum, these biochemical results indicate that WWP2 actively targets SOX2 for polyubiquitination and proteasomal degradation in GSCs.

We next asked if WWP2 plays a biological role in GSC maintenance. Given the critical role of SOX2 in GSC self-renewal and tumor formation, we hypothesized that WWP2 knockdown might promote these GSC phenotypes. Using the WWP2 RNAi validated by cDNA-specific rescue (Supplementary Fig. 5C), we found that WWP2 knockdown increased GSC self-renewal capacity in two different GSC lines (Fig. 4F), consistent with WWP2’s role as an E3 ubiquitin ligase that targets SOX2 protein for proteasomal degradation.

### TRIM26 blocks WWP2 interaction with SOX2 and WWP2-mediated polyubiquitination and proteasomal degradation of SOX2

**Fig. 5:**
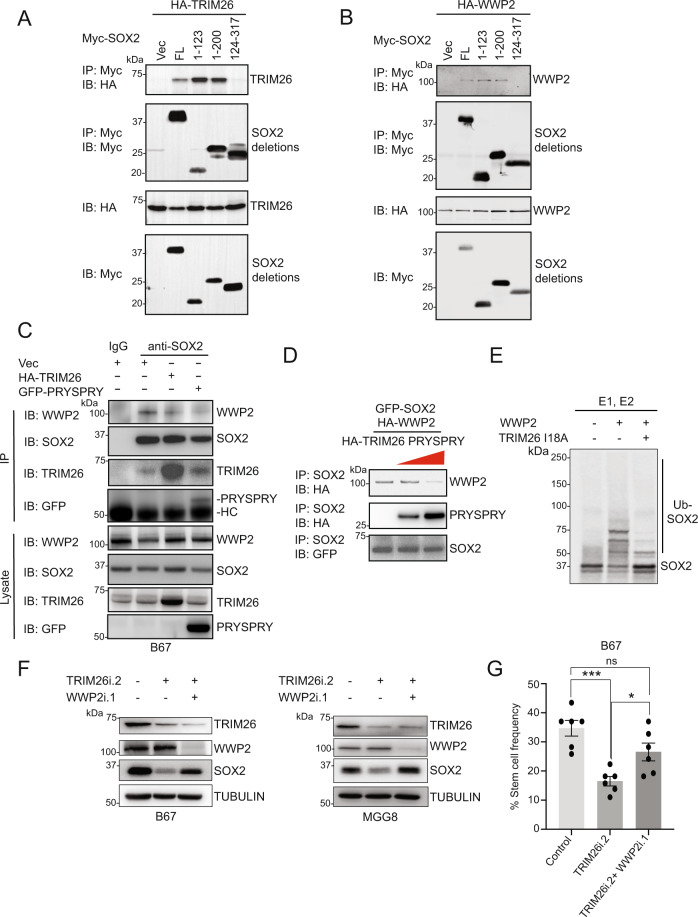
TRIM26 blocks WWP2-mediated polyubiquitination and proteasomal degradation of SOX2. **A** Transfected HEK293T cell lysates were subjected to immunoprecipitation (IP). Input lysates and IP samples were subjected to immunoblotting (IB). Data were representative of three independent experiments. **B** Transfected HEK293T cell lysates were subjected to immunoprecipitation (IP). Input lysates and IP samples were subjected to immunoblotting (IB). Data were representative of three independent experiments. **C** GSCs transduced with vector (Vec) or HA-TRIM26- or GFP-TRIM26 PRYSPRY-expressing lentiviruses were treated 4 days later with MG132 (10 μM) for 4 h to maintain SOX2 protein levels, and lysates were subjected to anti-SOX2 immunoprecipitation (IP). Input lysates and IP samples were subjected to immunoblotting. Data were representative of three independent experiments. **D** HEK293T cells were singly transfected with expression plasmids for HA-TRIM26 PRYSPRY, HA-WWP2, and GFP-SOX2, and lysates harvested. GFP-SOX2 and HA-WWP2 expressing lysates were added to all three conditions in equal amounts. HA-TRIM26 PRYSPRY-expressing lysate was excluded from the first condition and added in incremental amounts to the last two conditions. Lysates were subjected to anti-SOX2 immunoprecipitation (IP). IP samples were assayed by immunoblotting (IB). Data were representative of three independent experiments. **E** HEK293T cells singly transfected with HA-WWP2 and HA-TRIM26-I18A expression plasmids were harvested and subjected to anti-HA immunoprecipitation. Recombinant E1 and E2 (UBE2D3) enzymes were added to all three conditions. HA-WWP2 IP beads, HA-TRIM26-I18A IP beads, and in vitro synthesized, ^35^S-labeled SOX2 were added as indicated. In vitro ubiquitination reactions were performed and subjected to electrophoresis and fluorography to detect SOX2. Data were representative of three independent experiments. **F** GSCs transduced with WWP2 RNAi or control were transduced with TRIM26 RNAi or control 2 days later. Four days later, lysates were subjected to immunoblotting. Data were representative of three independent experiments. **G** GSCs transduced with WWP2 RNAi or control were transduced with TRIM26 RNAi or control 2 days later and then subjected to ELDA as in Fig. 3A. Data represent mean ±  SEM (*n* = 5, ANOVA, ***P < 0.0005, *P < 0.05, ns nonsignificant). See Source Data File for exact *P* values and additional statistical parameters.

We hypothesized, based on our structure-function studies, that TRIM26 might inhibit WWP2-mediated polyubiquitination of SOX2 by competing with WWP2 for binding to SOX2. We thus performed SOX2 deletion mapping experiments in a heterologous system to identify the SOX2 region that binds TRIM26 and WWP2 (Fig. 5A). HEK293T cells were transfected with epitope-tagged, full-length TRIM26 or WWP2 along with SOX2 deletion mutants that span its entire 317 amino acid length. Co-immunoprecipitation experiments using protein lysates from these transfected cells revealed that both TRIM26 and WWP2 interact with the N-terminal 123 amino acids of SOX2, consistent with a possible competitive binding model. To directly test this hypothesis, we first asked if TRIM26 overexpression could disrupt the endogenous interaction between WWP2 and SOX2 in GSCs (Fig. 5C). Indeed both TRIM26 full length and the PRYSPRY domain decreased endogenous co-precipitation of WWP2 with SOX2. Second, we performed in vitro binding experiments using lysates from HEK293T cells transfected separately with expression plasmids for HA-tagged TRIM26 PRYSPRY, HA-tagged WWP2, and GFP-tagged SOX2. We mixed HA-WWP2- and GFP-SOX2-expressing lysates in vitro, together with increasing amounts of HA-PRYSPRY-expressing lysates. Following SOX2 IP, immunoblot analysis revealed that increasing amounts of HA-PRYSPRY lysate decreased WWP2 interaction with GFP-SOX2 (Fig. 5D), overall consistent with a competitive binding model. To test if TRIM26 impacts WWP2-mediated SOX2 ubiquitination, we performed in vitro ubiquitination experiments using in vitro translated ^35^S-labeled SOX2 as substrate (Fig. 5E). We first verified that WWP2 ubiquitinates SOX2 in vitro. The addition of RING domain-mutant TRIM26-I18A effectively blocked WWP2-mediated polyubiquitination of SOX2 (Fig. 5E). In the context of GSCs, the TRIM26 RNAi-induced decrease in SOX2 protein could be reversed by simultaneous WWP2 knockdown in two distinct GSC lines (Fig. 5F). Consistent with this biochemical result, we found that the TRIM26 knockdown-induced inhibition of self-renewal could also be reversed by WWP2 knockdown in GSCs (Fig. 5G). Together, these results indicate that WWP2 is a *bona fide* SOX2-directed E3 ubiquitin ligase in GSCs and that TRIM26 directly disrupts WWP2-SOX2 binding and SOX2 polyubiquitination, leading to the maintenance of SOX2 protein levels and GSC biologic phenotypes.

### The TRIM26/WWP2 ubiquitin signaling pathway controls the tumorigenic capacity of GSCs

**Fig. 6:**
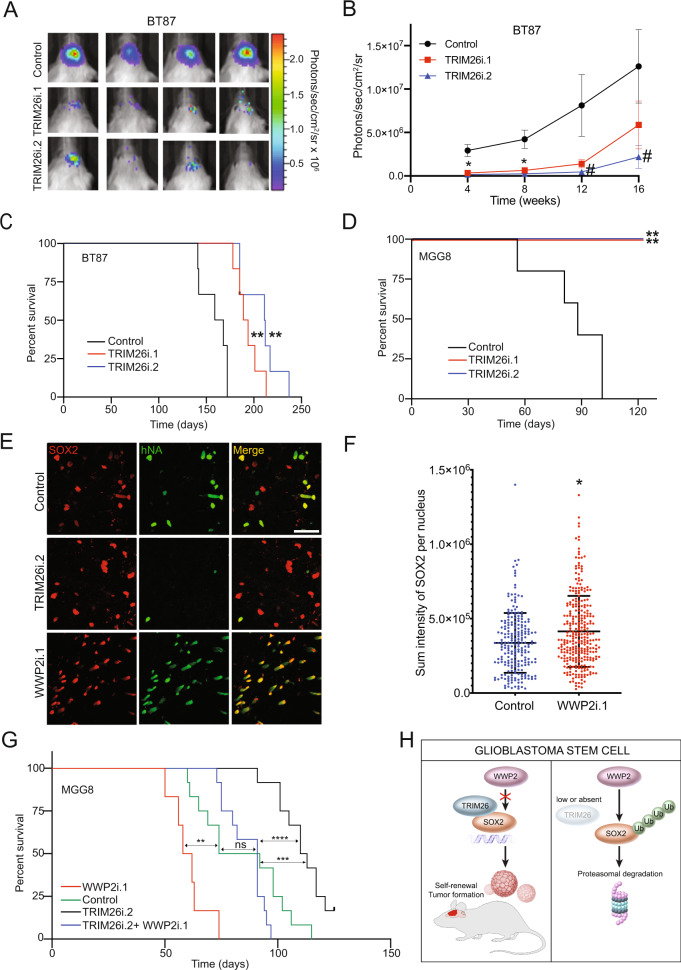
The TRIM26/WWP2 ubiquitin signaling pathway controls GSC tumorigenicity in vivo. **A** GSCs expressing Firefly luciferase were transduced with TRIM26 RNAi or control. Six days later, 2.5 × 10^5^ GSCs were injected into the brains of mice. Bioluminescent live imaging (BLI) was performed 8 weeks post-injection. Four representative animals are shown. **B** Quantification of BLI in (**A**). Data represent mean ± SEM (Control: *n* = 12 for 4, 8 weeks; 6 for 12, 16 weeks. TRIM26i.1: *n* = 11 for 4, 8 weeks; 5 for 12, 16 weeks. TRIM26i.2: *n* = 13 for 4, 8 weeks; 7 for 12, 16 weeks. ANOVA, *P < 0.001 for RNAi compared to control, #*P* < 0.04 for TRIM26i.2 only). See Source Data File for exact *P* values and statistical parameters. **C** Neurological deficit-free survival of mice treated as in (**A**) is shown (*n* = 6, log-rank, **P < 0.001). See Source Data File for exact *P* values and statistical parameters. **D** About 1 × 10^4^ GSCs transduced with TRIM26 RNAi or control were injected in mice as in (**A**). Neurological deficit-free survival is shown (*n* = 5, log-rank, **P < 0.005). See Source Data File for exact *P* values and statistical parameters. **E** About 5 × 10^4^ MGG8 GSCs transduced with TRIM26 RNAi, WWP2 RNAi, or control were injected into mice. Four weeks later, brains were processed for human nuclear antigen (hNA) and SOX2 immunofluorescence. Bar = 50 μm. Data were representative of three independent experiments. **F** MGG8 GSCs transduced with WWP2 RNAi or control were injected into mice, and brains subjected to immunofluorescence as in (**E**). SOX2 intensity per cell nucleus was quantified by z-stack (Control: *n* = 225 nuclei; WWP2i.1: *n* = 293 nuclei in three animals per condition, unpaired *t*-test, *P* < 0.001). See Source Data File for exact *P* values and statistical parameters. **G** GSCs transduced with WWP2 RNAi or control were transduced 2 days later with TRIM26 RNAi or control. About 5 × 10^4^ GSCs were injected into mice. Neurological deficit-free survival is shown (*n* = 6 for WWP2i and *n* = 12 for Control, TRIM26i.2, and TRIM26i.2 + WWP2i.1. log-rank, **P < 0.005, ***P < 0.001, ****P < 0.0001, ns nonsignificant). See Source Data File for exact *P* values and statistical parameters. **H** Schematic of TRIM26/WWP2 competitive binding model with SOX2 in GSCs.

Finally, we asked if the TRIM26/WWP2 ubiquitin signaling pathway is relevant for in vivo GBM tumor formation in GSC-based mouse models. We first generated a GSC line transduced with a GFP-T2A-luciferase lentivirus to enable bioluminescent live imaging (BLI) to monitor tumor burden in vivo. GSCs were infected with TRIM26 RNAi or control RNAi and injected into the right putamen of NOD-SCIDγ mice. BLI over a period of 20 weeks demonstrated diminished tumor growth in animals bearing TRIM26 knockdown GSCs, particularly in the first 16 weeks (Fig. 6A, B and Supplementary Fig. 6A). Importantly, mice injected with TRIM26 knockdown GSCs demonstrated increased neurologic deficit-free survival compared to animals injected with control-infected GSCs (Fig. 6C). These results were confirmed using a second GSC line (Fig. 6D). Overall, consistent with the biochemical and cellular results, these data confirm the importance of TRIM26 in GSC tumorigenicity in animals.

Next, we tested if TRIM26 and WWP2 control SOX2 protein levels in tumor cells in vivo. Mice were orthotopically injected with TRIM26 knockdown-, WWP2 knockdown, or control-infected GSCs, and 4 weeks later, brains were harvested for immunofluorescence. Using a human nuclear antigen-antibody to detect human glioblastoma cells in the mouse brain, we then used confocal microscopy to image tumor cells and SOX2 immunofluorescence intensity in individual cell nuclei (Fig. 6E and Supplementary Fig. 6B). Strikingly, there were few tumor cells observed in mice injected with TRIM26 knockdown GSCs, precluding any robust quantitative analysis of SOX2 immunofluorescence in these brains. However, these results do highlight that TRIM26 is critical for GSC maintenance in vivo. In contrast, mice injected with WWP2 knockdown GSCs showed a high number of tumor cells (Fig. 6E and Supplementary Fig. 6B). Quantitative confocal analysis of SOX2 immunofluorescence in WWP2 knockdown tumor cells demonstrated a significant increase in SOX2 intensity in individual cell nuclei compared to control tumor cells (Fig. 6F), suggesting WWP2 is a SOX2 E3 ligase in glioblastoma cells in vivo.

Finally, we tested if the TRIM26/WWP2 competition model operates in GSC tumorigenicity in vivo. Consistent with the data above, mice injected with TRIM26 knockdown GSCs exhibited longer survival than those injected with control-infected cells (Fig. 6G). In accordance with WWP2’s function as a SOX2 E3 ligase, mice bearing WWP2 knockdown GSCs exhibited shorter survival than those injected with control-infected cells. In agreement with our TRIM26/WWP2 model, mice injected with TRIM26 RNAi + WWP2 RNAi GSCs had significantly shorter survival than mice injected with TRIM26 RNAi GSCs. In fact, mice injected with TRIM26 RNAi + WWP2 RNAi exhibited similar survival to those injected with control-infected GSCs, demonstrating that WWP2 RNAi can reverse the survival benefit of TRIM26 RNAi (Fig. 6G). In summary, these in vivo results demonstrate the functional antagonism between WWP2 and TRIM26, confirming the competitive biochemical mechanism of TRIM26 and WWP2 (Fig. 6H).

## Discussion

In this study, we identify the TRIM26 E3 ubiquitin ligase as a key regulator of SOX2 proteasomal degradation in GSCs and demonstrate that TRIM26, in a manner independent of its catalytic activity, inhibits WWP2, a SOX2-directed E3 ubiquitin ligase in GSCs, and thus protects SOX2 from polyubiquitination and proteasomal degradation. Consistently, we find that modulation of TRIM26 and WWP2 expression levels impacts GSC self-renewal capacity and in vivo tumorigenicity in orthotopic mouse models.

The pluripotency-related transcription factor SRY (sex-determining region Y)-box 2 (SOX2) has been extensively studied in glioblastoma. SOX2 has been reported to be highly expressed in most glioblastoma biopsy specimens, with SOX2 expression levels correlating with increased tumor grade and aggressiveness^33^. Similar to SOX2’s role in normal tissue stem cell homeostasis, various studies have demonstrated that SOX2 expression and transcriptional activity is required for the self-renewal and tumorigenicity of GSCs^10,11^. It has been reported that while oncogenic receptor tyrosine kinase (RTK) signaling induces SOX2 expression during transformation in engineered glioblastoma mouse models, the maintenance of SOX2 expression and its tumor-driving transcriptional network appears independent of ongoing aberrant RTK activity^12^. Further, scRNA-seq analysis of *IDH*-wild-type glioblastomas revealed that SOX2 expression is maintained, albeit at differing levels, across all four defined cellular states and is associated with divergent oncogenic drivers^27,34^. Taken together, this accumulating functional and expression evidence positions SOX2 as an important and common signaling node for glioblastomas. However, there has been limited success in the development of direct small molecule and nucleotide-based SOX2 inhibitors for clinical application^33,35^. Therefore, targeting the regulation of SOX2 protein expression and function has emerged as an alternative strategy to interrupt the oncogenic functions of SOX2.

We found that SOX2 protein stability is dynamically regulated across multiple primary patient-derived GSC lines. In the context of cancer, and glioblastoma in particular, the functional consequences of altering SOX2 proteostasis have not been well established. We demonstrate, by manipulation of the counter-regulatory TRIM26 and WWP2 E3 ubiquitin ligases, that modulation of SOX2 protein stability has a significant impact on GSC self-renewal in vitro and tumorigenicity in vivo in mouse models. These findings add to increasing evidence in normal development that SOX2 protein stability is actively regulated by the ubiquitin-proteasome system (UPS) in both embryonic and neural stem cells, with functional implications for stem cell maintenance and fate commitment^29,31^. Further, our observations in glioblastoma provide the rationale for future studies to elucidate additional regulators of SOX2 protein stability in GSCs and suggest that SOX2-specific UPS-based strategies might represent a potentially viable avenue to therapeutically target GSCs.

Mechanistically, we found that TRIM26 non-catalytically inhibits WWP2 by competing with WWP2 for binding to the same N-terminal region on SOX2. This direct inhibitory counter-regulation between two non-paralogous E3 ubiquitin ligases reveals an unappreciated level of complexity to mammalian ubiquitin signaling. It has been shown that the E3 ubiquitin ligase TRIM67 outcompetes TRIM9 for binding to the actin polymerase VASP and prevents TRIM9-mediated polyubiquitination and degradation of VASP, with consequences for filopodial dynamics and axon guidance^36^. However, distinct from our mechanism, TRIM67 and TRIM9 are paralogs, with identical domains and 63% sequence identity. In addition to direct binding competition, the regulation of TRIM9 by TRIM67 also required TRIM67 ligase activity. Another study described competition between paralogous SCF E3 ligase adapter proteins FBXL3 and FBXL21 for ubiquitination and degradation of the circadian clock regulator CRY. While both SCF-FBXL3 and SCF-FBXL21 were able to ubiquitinate CRY, SCF-FBXL3 was found to be more potent at this activity, such that SCF-FBXL21-bound CRY in the nucleus was degraded at a slower rate and thus relatively protected compared to SCF-FBXL21-bound CRY^37^. Our study, by contrast, describes direct inhibitory competition between two non-paralogous E3 ubiquitin ligases (TRIM26 is a RING domain E3 ligase, while WWP2 is a HECT-type E3 ligase) that share essentially no sequence homology and have diametrically opposite roles in regulating SOX2 ubiquitination. Further, our data revealed that the antagonistic roles between TRIM26 and WWP2 could be fully attributed to competitive binding to SOX2 and did not require TRIM26’s ubiquitin ligase activity.

Our structure-function analysis of TRIM26 binding to SOX2 identified the C-terminal PRYSPRY domain of TRIM26 as sufficient to interact with SOX2, with consequences for SOX2 protein stability and GSC self-renewal. Additionally, our findings raise the intriguing possibility that other PRYSPRY-containing proteins might bind to and regulate SOX2 stability and transcriptional activity in GSCs and other stem cell contexts. In the same vein, it is possible that TRIM26 regulates other factors through this non-catalytic mechanism.

While our results delineate a clear and biologically important role for TRIM26 in regulating SOX2 protein stability in GSCs, whether TRIM26 has other important functions in GSC biology remains an open question. Potentially, TRIM26 could help to localize in addition to stabilizing SOX2 protein, and furthermore, SOX2-independent functions of TRIM26 may play a role in GSC maintenance. Finally, it would be of interest to elucidate the upstream signaling pathways that regulate TRIM26 expression in glioblastoma, including the potential role of interferon signaling as observed in other cellular contexts^38^.

## Methods

### Institutional approvals

All human study research related to this study has been approved by the Institutional Review Board (IRB #201211019 and #201409046, Washington University School of Medicine). All participants donating tissue signed informed consent prior to tissue banking. Our animal protocol (#21-0083) adheres to NIH and American Association for Laboratory Animal Science (AALAS) guidelines and has been approved by our Institutional Animal Care and Use Committee (IACUC).

### Antibodies and drugs

Antibodies used for this study include monoclonal rabbit anti-SOX2 (3579) (1:1000, Cell Signaling Technology), polyclonal goat anti-SOX2 (sc-17320) (2 μg antibody per 500 μg lysate protein, Santa Cruz Biotechnology), monoclonal rabbit anti-Sox2 SP76 (1:200, Cell Marque), monoclonal rabbit anti-SOX2 D9B8N (2 μg antibody per 500 μg lysate protein, Cell Signaling Technology), monoclonal mouse anti-alpha-Tubulin (1:4000, T5168) (Sigma), monoclonal mouse anti-HA.11 epitope tag (901502) (1:1000, Biolegend), anti-HA, rabbit monoclonal C29F4, (1:1000, Cell Signaling Technology), anti-Myc, mouse monoclonal, clone 4 A6, (1:1000, Millipore-Sigma), anti-Myc, mouse monoclonal, clone 9E10, (2 μg antibody per 500 μg lysate protein, Millipore-Sigma), polyclonal rabbit anti-GFP (A6455) (1:5000, Thermo Fisher), monoclonal mouse anti-GFP (A11120) (1 μg/mL lysate, Thermo Fisher), monoclonal mouse anti-TRIM26 (sc-393832) (1:200, Santa Cruz Biotechnology), monoclonal mouse anti-beta-Actin (sc-47778) (1:1000, Santa Cruz Biotechnology), monoclonal mouse anti-FLAG M2 (F3165) (1:2000, Sigma), polyclonal rabbit anti-His-Tag (1:1000, 2365) (Cell Signaling Technology), monoclonal mouse anti-FZR1(CDH1) (ab3242) (1:500, Abcam), polyclonal rabbit anti-Cullin-4A (1:1000, C0371) (Sigma), polyclonal rabbit anti-EDD1(UBR5) (A300-573A) (1:2000, Bethyl Laboratories), polyclonal rabbit anti-WWP2 (A302-935A) (1:2000, Bethyl Laboratories), monoclonal mouse anti-Human nuclear antigen 235-1 (1:500, Novus Biological), and Normal goat IgG (NI02) (Millipore-Sigma). Drugs used for this study include cycloheximide (Cayman chemical 14126) and proteasome inhibitor MG132 (Millipore-Sigma 474787).

### Plasmids

The following human gene target-directed shRNA plasmids from the Mission shRNA consortium purchased from Sigma-Aldrich were used: TRIM26 (TRCN0000004050 and TRCN0000004051), WWP2 (TRCN0000320849 and TRCN0000320847), UBR5 (TRCN0000003408 and TRCN0000003411), and CUL4A (TRCN0000320897 and TRCN0000320898). Control nontargeting plasmids were obtained from Sigma-Aldrich (SHC002 and SHC202). For CDH1, the following RNAi constructs were obtained via the Washington University RNAi Core: CDH1i.1 (5′-CAACGACAACAAGCTGCTGGT-3′), CDH1i.2 (5′-CCAGTCAGAACCGGAAAGCCA-3′), and a nontargeting control (5′- TGTTCGCATTATCCGAACCAT-3′). HA-WWP2, HA-TRIM26, and TRIM26 truncation mutants (HA-TRIM26 1-138, HA-TRIM26 1-223, HA-TRIM26 1-363, and HA-TRIM26 PRYSPRY) were cloned into pcDNA3 for transient transfections. GFP-SOX2 cDNA was cloned into the N103 vector (kindly provided by Dr. Andrew Yoo at Washington University). Lentiviral expression constructs were generated by cloning the following cDNAs into the pLV-EF1a-IRES-Blast plasmid (Addgene 85133): TRIM26 (WTres, I18Ares, and C16AC36Ares), GFP-TRIM26 PRYSPRY, WWP2res, SOX2-6X-His, and Renilla luciferase. Flag-Ubiquitin cDNA was cloned into lentiviral vector pCDH (kindly provided by Dr. Jeremy Rich (UCSD)). GST-SOX2 was generated by using the bacterial expression plasmid pGEX-4T1. The human *SOX2* regulatory region 2 (hSRR2)/minimal CMV promoter (mCMV)-driven GFP-T2A luciferase lentiviral constructs were generated using the pGreenfire1 mCMV-GFP-T2A luciferase plasmid (Systems Biosciences) as previously described in ref. ^13^. All plasmids were confirmed by sequencing.

### Cell culture

The generation of adherent human GSC cultures has been described in ref. ^39^. In brief, surgical tumor samples were dissociated mechanically and by incubation in Accutase (SIGMA) for 20–60 min at 37 ^**°**^C. Cell suspensions were passed through a 70-micron cell strainer (Falcon) and plated using NeuroCult NS-A Basal Medium (Human) supplemented with N2 supplement, B27 supplement, Glutamax, 75 μg/ml BSA, and EGF and FGF-2 (Peprotech) (hereafter “GSC media”), each at 20 ng/ml, on polyornithine and laminin (SIGMA)-coated Primaria dishes/flasks (BD Bioscience). Growth factors (EGF/FGF-2) were replenished every 2–3 days. Cells were routinely used between passages 5 and 20. Informed consent was obtained from patients for use of human tissue and cells, and all human tissue-related protocols used in this study were approved by the Institutional Review Board (Washington University). BT87 GSCs were a kind gift from Dr. Sunit Das at the University of Toronto and were cultured as described above. MGG8 GSCs were the kind gift of Dr. Daniel Cahill at the Massachusetts General Hospital. MGG8 GSCs were cultured as non-adherent spheres in ultralow attachment culture dishes (Corning) using Neurobasal Media supplemented with N2 supplement, B27 supplement, sodium pyruvate, Nonessential amino acids (NEAA), and EGF and FGF-2 at 20 ng/ml each. Human embryonic kidney 293 T (HEK293T) cells were cultured in Dulbecco’s modified Eagle’s medium with 10% fetal bovine serum (FBS) and penicillin/streptomycin (Life Technologies). All cell lines were incubated at 37 ^**°**^C with 5% CO_2._ Lentiviral transduction was performed by adding virus with 4 μg/mL of polybrene for 4 h to cells. For rescue and epistasis experiments, GSCs were transduced with TRIM26 or WWP2 RNAi or control lentivirus 1 day after plating and then transduced with TRIM26, WWP2, or SOX2 expression virus or control virus on the second day after the initial transduction. Cells were selected in 2 μg/mL of puromycin 1–2 days after infection. For self-renewal and in vivo tumorigenicity experiments, GSCs were utilized 6 days following indicated viral infections.

### Extreme limiting dilution analysis

Cells were plated at fivefold dilutions (3000, 600, 120, 24, 5, or 1 cell/well) in Corning ultralow attachment 96-well plates. Fourteen days later, the number of wells containing spheres was counted and used to calculate the frequency of self-renewing GSCs by online software (http://bioinf.wehi.edu.au/software/elda/)^8,40^.

### Xenotransplantation

Animals were used in accordance with a protocol approved by the Animal Studies Committee of the Washington University School of Medicine per the recommendations of the Guide for the Care and Use of Laboratory Animals (NIH). About 250,000 cells per animal (BT87 GSCs TRIM26 RNAi experiment), 10,000 cells per animal (MGG8 GSCs TRIM26 RNAi experiment), or 50,0000 cells per animal (MGG8 GSCs WWP2 RNAi experiment and MGG8 TRIM26/WWP2 RNAi epistasis experiment) were injected stereotactically into the right putamen of ~6-week-old female NOD-SCIDγ mice. The coordinates used were: 1 mm rostral to bregma, 2 mm lateral, and 2.5 mm deep.

### Live bioluminescence imaging

GSCs stably expressing GFP-T2A luciferase by lentiviral transduction were infected with indicated lentiviruses and injected into the brains of NOD-SCIDγ mice as above. For bioluminescence imaging, animals were anesthetized with 2.5% (vol/vol) isoflurane, injected intraperitoneally with 150 μg/mL d-luciferin (Biosynth) in PBS, and imaged with a charge-coupled device (CCD) camera-based bioluminescence imaging system (IVIS 100; Perkin-Elmer; exposure time 3–5 min, binning 16, a field of view 12, f/stop 1, open filter; Molecular Imaging Center, Washington University). Signal was displayed as photons per s/cm^2^ per steradian.

### Transient transfection

Polyethyleneimine (PEI, Polysciences #24765-2) was dissolved in ddH20 to a concentration of 1 mg/mL. The solution was then adjusted to pH 7.0 and filter sterilized. A mixture of plasmid DNA and PEI solution was made in OPTI-MEM (Life Technologies) and incubated at room temperature for 15 min. DNA/PEI complexes were applied to cells, and media was changed after 12–16 h. Experiments were performed 36 h following transfection.

### Lentiviral production

HEK293T cells were plated with a goal density of 70–80% after 1 day. The next day, transfection was performed using the PEI transfection method to introduce the plasmid of interest along with packaging plasmid psPAX2 and envelope plasmid pCMV-VSVG to Opti-MEM (Life Technologies). On day 6, the medium from the plates was collected and spun down at 1200x*g* for 5 min at 4 °C. The supernatant was filtered through 0.45-micron filters. Lenti-X Concentrator (Clontech) was then added to the filtrate and mixed, and the tubes were incubated at 4 °C for 6–7 h. Lentiviruses were then centrifuged at 1500x*g* for 45 min at 4 °C. The supernatant was aspirated; pellets were resuspended in one-tenth of the original medium volume of cold PBS and stored at −80 °C in aliquots. Viral copy number was adjusted for transduction of GSCs on the basis of titer measured using the Lenti-X qRT-PCR titration kit (Clontech).

### SOX2-interacting protein sequence analysis by LC-MS/MS

Immunoprecipitations of endogenous human SOX2 were performed with anti-SOX2 antibody (goat, Santa Cruz) or control antibody (goat IgG control) using 1% NP-40 lysates from GSC line B36. Immune complexes were pulled down with protein G-sepharose beads, and beads were washed 7 times with lysis buffer. Immunoprecipitates were boiled in 1X Laemmli buffer and resolved by SDS-PAGE. Following Coomassie brilliant blue staining, gel lanes were excised, avoiding heavy and light chain bands.

LC-MS/MS was then performed (Taplin Biological Mass Spectrometry Facility, Harvard Medical School). Excised gel bands were cut into ~1 mm^3^ pieces. Gel pieces were then subjected to a modified in-gel trypsin digestion procedure^41^. Gel pieces were washed and dehydrated with acetonitrile for 10 min. followed by the removal of acetonitrile. Pieces were then completely dried in a speed-vac. Rehydration of the gel pieces was with 50 mM ammonium bicarbonate solution containing 12.5 ng/µl modified sequencing-grade trypsin (Promega, Madison, WI) at 4 °C. After 45 min, the excess trypsin solution was removed and replaced with 50 mM ammonium bicarbonate solution to just cover the gel pieces. Samples were then placed in a 37 °C room overnight. Peptides were later extracted by removing the ammonium bicarbonate solution, followed by one wash with a solution containing 50% acetonitrile and 1% formic acid. The extracts were then dried in a speed-vac (~1 h). The samples were then stored at 4 °C until analysis.

On the day of analysis, the samples were reconstituted in 5–10 µl of HPLC solvent A (2.5% acetonitrile, 0.1% formic acid). A nano-scale reverse-phase HPLC capillary column was created by packing 2.6 µm C18 spherical silica beads into a fused silica capillary (100 µm inner diameter x ~30 cm length) with a flame-drawn tip^42^. After equilibrating the column each sample was loaded via a Famos autosampler (LC Packings, San Francisco CA) onto the column. A gradient was formed and peptides were eluted with increasing concentrations of solvent B (97.5% acetonitrile, 0.1% formic acid).

As peptides were eluted they were subjected to electrospray ionization and then entered into an LTQ Orbitrap Velos Pro ion-trap mass spectrometer (Thermo Fisher Scientific, Waltham, MA). Peptides were detected, isolated, and fragmented to produce a tandem mass spectrum of specific fragment ions for each peptide. Peptide sequences (and hence protein identity) were determined by matching protein databases with the acquired fragmentation pattern by the software program, Sequest (Thermo Fisher Scientific, Waltham, MA)^43^. All databases include a reversed version of all the sequences and the data was filtered to between a one and two percent peptide false discovery rate.

### Co-immunoprecipitation and immunoblot analysis

Cells were lysed in 1% NP-40 lysis buffer containing 20 mM Tris [pH 8], 200 mM NaCl, 10% glycerol, 1 mM EDTA, 10 mM NaF, 1 mM sodium orthovanadate, and a protease inhibitor cocktail (Calbiochem). Clarified lysates were precleared with protein A/G-sepharose (Life Technologies), and immunoprecipitations were performed overnight at 4°C with indicated antibodies followed by protein A/G-sepharose beads. Beads were washed six times with lysis buffer and boiled in Laemmli sample buffer. Samples were separated by SDS-PAGE and transferred to 0.45 μm Immobilon-P PVDF membrane (EMD Millipore). Membranes were blocked in 5% Milk in Tris-buffered saline with Tween-20 (TBST) at room temperature and incubated with primary antibodies at 4 °C overnight or at room temperature for 4 h. Membranes were washed with TBST and incubated with appropriate horseradish peroxidase-conjugated secondary antibodies for 1 h at room temperature. Membranes were then washed with TBST and developed using Pierce ECL western blotting substrate (Thermo Scientific).

### Cellular ubiquitination assays

GSCs were transduced with pCDH-CMV-Flag-Ubiquitin and pLV-EF1α-SOX2-6X-Histidine plasmids to stably express Flag-Ubiquitin and C-terminal 6X-His-tagged human SOX2 (SOX2-6X-His). GSCs were then transduced with either of 2 TRIM26- or WWP2-targeting RNAi constructs or the respective controls. On day 5 after RNAi transduction, cells were treated with 10 μM MG132 for 8 h. Cell were washed with cold PBS and detached from culture dishes using Accutase (Sigma). About 10% of the cell pellet (for input) was lysed in 1% NP-40 lysis buffer containing 20 mM Tris [pH 8], 200 mM NaCl, 10% glycerol, 1 mM EDTA, 10 mM NaF, 1 mM sodium orthovanadate, and a protease inhibitor cocktail (Calbiochem). Input lysates were clarified by centrifugation, subjected to Bradford assay to quantitate protein concentrations, and boiled in Laemmli sample buffer for immunoblot analysis. The rest of the cell pellet (90%) was resuspended in guanidium lysis buffer (6 M Guanidinium-HCl, 0.1 M Na_2_HPO_4_/NaH_2_PO_4_, 10 mM Tris-HCl (pH 8), 0.005 M imidazole, 0.01 M β-mercaptoethanol, and EDTA-free protease inhibitor cocktail). The cells were sonicated, centrifuged, and the supernatant was transferred to a 15 ml conical containing 4 ml of guanidium lysis buffer. Immunoprecipitation was performed using Ni^2+^-NTA-agarose beads for 4 h at room temperature. The agarose beads were then washed once with guanidium lysis buffer, once with Urea wash buffer (8 M Urea, 0.1 M Na_2_HPO_4_/NaH_2_PO_4_, 10 mM Tris-HCl (pH 6.8), 0.005 M Imidazole, and 0.01 M β-mercaptoethanol), twice with urea wash buffer containing 0.1% Triton X-100, and twice with His-Protein Lysis buffer:50 mM Tris (pH 7.3), 250 mM NaCl, 0.05% Triton X-100, 20 mM Imidazole, and 3–5 mM 2-mercaptoethanol. Bound protein was eluted from beads using His-Protein Lysis Elution Buffer: 50 mM Tris (pH 7.3), 250 mM NaCl, 0.05% Triton X-100, 400 mM Imidazole (add fresh: 3–5 mM- 2-mercaptoethanol). Laemmli sample buffer was added, and the eluate was boiled and subsequently analyzed by immunoblot.

### In vitro ubiquitination assays

Radiolabeled substrate, ^35^S-myc-SOX2, was in vitro translated from pcdna3-myc-hSOX2 plasmid template using the TNT-coupled reticulocyte lysate system (Promega). SOX2 from this reaction was purified by immunoprecipitation with SOX2 antibody using the same protocol as above for co-immunoprecipitation experiments. HEK293T cells were transfected with an empty-vector (pcdna3) or with pcdna-HA-TRIM26-I18A-mut or HA-WWP2. 36 h post-transfection, lysates from HEK293T cells were clarified and subjected to immunoprecipitation with HA-tag antibody. Immunoprecipitated HA-TRIM26-I18A and empty-vector (pcdna3) samples were peptide-eluted from IP beads by incubation with fivefold excess (by weight) HA-peptide for 2 h at room temperature in PBS. ^35^S-myc-SOX2 was preincubated with peptide-eluted HA-TRIM26-I18A or eluted control (empty pcdna3) for 16 h at 4°. Control beads were added to the ^35^S-myc-SOX2/Contol-pcdna3 preincubated sample for the negative ubiquitination reaction control. HA-WWP2 IP beads were added to the ^35^S-myc-SOX2/Contol-pcdna3 and ^35^S-myc-SOX2/HA-TRIM26-I18A preincubated samples. About 100 μM ubiquitin, 100 nM E1 enzyme, 1 μM UBE2D3 E2 enzyme, and 1x E3 ligase reaction buffer (Boston Biochem/R&D Systems) were added to each of the reactions. The ubiquitination reaction was initiated by the addition of 100 μM MgATP. The reaction was incubated at 37 °C for 60 min with gentle agitation. The reaction was terminated by the addition of Laemelli sample buffer and boiling. Samples were separated by SDS-PAGE using 4–12% gradient gels (Invitrogen). The gel was fixed and dried and subsequently analyzed by autoradiography to detect unmodified and polyubiquitinated ^35^S-myc-SOX2.

### In vitro binding

GST-human SOX2 and the corresponding GST control proteins were bacterially expressed and isolated using Glutathione-Sepharose 4 Fast Flow (GE Healthcare) according to the manufacturer’s instructions. Using pcDNA3-HA-TRIM26 full length and deletion mutant plasmids, ^35^S-labeled products were in vitro translated using the TNT-coupled reticulocyte lysate system (Promega) and incubated with indicated GST-fusion proteins bound to glutathione-sepharose 4 Fast Flow beads in 1% NP-40 lysis buffer at 4 °C for 16 h (GE Healthcare). The beads were washed six times with lysis buffer and boiled in Laemmli sample buffer. Proteins were resolved by SDS-PAGE, and ^35^S-labeled proteins were visualized by autoradiography. GST-fusion proteins were assessed by Coomassie Brilliant Blue R-250 (Biorad).

### In vitro luciferase assay

Cells seeded on PLO and laminin-coated −96-well plates were stably infected with SOX2 reporter pGreenfire1 2X-hSRR2/mCMV-GFP-T2A-Firefly-luciferase. To normalize SOX2 reporter activity, control pLV-EF1α-Renilla-Luciferase lentivirus was co-transduced or protein levels in the same well were measured after luciferase measurement. Cells were transduced with indicated RNAi viruses and selected with puromycin. Six days later, cells were assayed for luciferase activity using the One-Glo Dual-Luciferase reporter system (Promega). Luciferase signal was quantified by luminometry using a Biotek Cytation 5 Multiwell plate reader. Firefly luciferase signal from SOX2 reporter activity was divided by the corresponding Renilla Luciferase reading or protein level to control for cell number. Data were then normalized to the corresponding control RNAi condition.

### Cell dissociation and tissue processing

Fresh tumor samples were dissociated using the gentleMACS Octo Dissociator and Brain tumor dissociation kit (Miltenyi). Myelin and red blood cells were removed using the MACS Myelin removal beads II and ACK Red Blood Cell lysis buffer, respectively. Dead cells were removed (MACS Dead cell removal microbeads) and viable cells were counted by Trypan blue exclusion.

### RNA-sequencing

GSCs were transduced with shRNA-expressing lentiviruses (*n* = 3 independent samples per condition), and 5 days later, total RNA was isolated using the RNeasy Plus Mini Kit (Qiagen). Ribosomal RNA was removed by a hybridization method using Ribo-ZERO kits (Illumina). mRNA was fragmented and reverse transcribed to yield double-stranded cDNA. cDNA was blunt-ended, had an A base added to the 3′ ends, and then had Illumina sequencing adapters ligated to the ends. Ligated fragments were then amplified for 12 cycles using primers incorporating unique index tags. Fragments were sequenced on an Illumina HiSeq-2500 using single-end reads extending 50 bases. RNA-seq reads were aligned to the Ensembl release 76 assembly with STAR version 2.0.4b. Gene counts were derived from the number of uniquely aligned unambiguous reads by Subread:featureCount version 1.4.5. Transcript counts were produced by Sailfish version 0.6.3. Sequencing performance was assessed for a total number of aligned reads, a total number of uniquely aligned reads, genes and transcripts detected, ribosomal fraction known junction saturation, and read distribution over known gene models with RSeQC version 2.3. All gene-level and transcript counts were then imported into the R/Bioconductor package EdgeR and TMM normalization size factors were calculated to adjust for samples for differences in library size. Genes or transcripts not expressed in any sample were excluded from further analysis. The TMM size factors and the matrix of counts were then imported into R/Bioconductor package limma, and weighted likelihoods based on the observed mean–variance relationship of every gene/transcript were then calculated for all samples with the Voom function. The performance of the samples was assessed with a Spearman correlation matrix and multidimensional scaling plots. Gene/transcript performance was assessed with plots of residual SD of every gene to their average log-count with a robustly fitted trend line of the residuals. Generalized linear models with robust dispersion estimates were then created to test for gene/transcript-level differential expression. Differentially expressed genes and transcripts were then filtered for FDR adjusted *P* ≤ 0.05.

### 3′ single-cell RNA library construction and sequencing

Dissociated tumor cells were processed using the 10x Genomics Chromium Controller and the Chromium Single Cell 3′V2 Library & Gel Bead Kit following the manufacturer’s protocols (https://tinyurl.com/ybpg2pfz). This yielded cDNA libraries for 47,510 cells (including 18,383 from two B148 libraries, 13,398 from B150, and 15,729 from B152), which were sequenced on an Illumina NovaSeq S2 flow cell to a median per-library depth of 104,535 reads per cell.

### scRNA-seq expression analysis

Demultiplexing, alignment, and transcript quantification was performed using the Cell Ranger pipeline (10x Genomics, default settings, Version 3.0.1)^44^ Using the Seurat R package (V3)^45^, cells that contained fewer than 700 expressed genes, were ranked higher than the 93rd percentile with respect to UMI count, or contained more than 5% mitochondrial transcripts were removed. Genes that were expressed in fewer than ten cells were also removed. For each cell, the expression of each gene was normalized to the sequencing depth of the cell, scaled to a constant depth (1 × 10^6^), and log-transformed. The 2000 most variable genes were selected using the variance stabilizing transformation provided in Seurat. Principal component analysis was performed on the variable genes, and 11 components were retained for downstream analyses, including UMAP visualization and unsupervised graph-based clustering (clustering resolution = 0.7). The cell cycle phase was determined using the methodology provided in Seurat, based on the relative expression of phase-specific genes, and cell cycle signal was removed via multiple regression.

Tumor and normal cells were distinguished based on their expression of *PTPRC* (encoding CD45), and the presence of copy number alterations (CNAs). CNAs were identified using the CONICSmat package for R^46^. The default filtering and normalization procedures for CONICSmat were followed, as outlined in https://goo.gl/tFYLEh. Mixture model results were obtained for each chromosome using three clusters. Cells were then clustered (*k* = 2) according to their *z*-scored posterior probabilities for all candidate regions (3q, 7p, 7q, 9p, 10p, 10q, 14q, 19p, 20p, 20q, and 22q), and the cluster of cells containing CNAs was taken to represent tumor cells. The resulting tumor/normal designations were consistent with *PTPRC* expression (low in tumor cells, high in normal cells). Finally, cells in the UMAP were colored according to their *z*-scored posterior probabilities for the largest alterations (7p, 7q, 10p, 10q, and 22q).

For analyses with an independent scRNA-seq dataset (Neftel et al, *Cell*, 2019)^27^, both the 10X Genomics and Smart-seq2 datasets were downloaded. Pseudobulk analysis of expression was performed as follows: [(Sum of counts overall tumor cells)/(total sequencing reads)]*[total sequencing reads/10E6]. This provides relative expression in counts-per-million (CPM) of the gene (i.e., relative to all counted transcripts from all genes).

### Immunofluorescence and quantitative confocal microscopy

Mice injected with lentivirally infected MGG8 cells were perfused, brains harvested, fixed with 4% PFA, cryopreserved with 30% sucrose, flash-frozen, and sectioned at 35 μm thickness along the coronal plane using the tissue near the needle tract for tumor cell injection based on stereotaxic coordinates. Brain sections were washed with PBS, blocked with BSA and goat serum solution, and membrane permeabilized with 0.4% Triton X in blocking buffer. Sections were then incubated overnight at 4° with primary antibodies (rabbit anti-Sox2 SP76 (Cell Marque, 1:200), mouse anti-Human nuclear antigen 235-1 (Novus Biological, 1:500) and 2 h at RT with secondary antibodies (Goat Anti-Rb Alexa 568, Goat Anti-Ms Alexa 647 (Invitrogen, 1:500). Sections were counterstained with Hoechst 33258 (Sigma, Cat.94403, 1:5000) 5 min at RT and mounted with Fluoromount-G (Southern Biotech, Cat.0100-01). Brain sections containing the needle tract were selected for SOX2 quantification. Images were taken with Zeiss LSM 880 Confocal microscope. High NA oil immersion with 63X objective and z-stack imaging were further used for SOX2 quantification. For groups of control and WWP2 RNAi GSC injections, 3–4 sections per animal, three animals per group were imaged for statistical analysis. MGG8 tumor cell nuclei were 3D-segmented by Human nuclear antigen in Imaris (Oxford Instruments). Only intact nuclei were selected for segmentation. Based on Hoechst counterstain, doublet nuclei were manually split or excluded when the splitting was not feasible (<1% total cells). To reduce bias, segmentation was completed by two independent analysts in a blinded manner. Sum intensities of SOX2 within individual segmented nuclei were calculated and exported from Imaris. In total, about 250 cells in each group were segmented and quantified.

### Cell viability assay

Cellular viability of neurosphere GSC lines was assessed by measuring the amount of ATP generated by viable cells using the CellTiter-Glo luminescent cell viability assay (Promega) per the manufacturer’s instructions. ATP values were normalized to control RNAi (=100%).

### Statistics and other software

Statistical analyses were performed with Prism version 8 and 9 as well as Microsoft Excel v16.41. The unpaired Student’s *t*-test was used for comparisons in experiments with only two groups. In experiments with more than two comparison groups, a one-way analysis of variance (ANOVA) was performed followed by the Bonferroni test for pairwise comparisons. All statistical analyses and parameters are summarized in the Source Data file for each relevant Figure panel. Select figure panels (Fig. 1D) were created using Biorender.com. Uncropped and unprocessed scans of blots and other biochemistry experiments are provided in Supplementary Information.

### Reporting Summary

Further information on research design is available in the Nature Research Reporting Summary linked to this article.

## Supplementary information

Supplementary Information

Description of Additional Supplementary Files

Supplementary Data 1

Supplementary Data 2

Reporting Summary

## Data Availability

The single-cell RNA-seq data generated in this study have been deposited in the Zenodo and Annotare databases under the following accession codes—Zenodo 10.5281/zenodo.4031852 (https://zenodo.org/record/4031852#.YS7KZy2cbOR), Annotare (Array Express) Accession # E-MTAB-9435 (https://www.ebi.ac.uk/fg/annotare/login/). The mass spectrometry proteomics data have been deposited to the ProteomeXchange Consortium via the PRIDE partner repository with the dataset identifier PXD028933 and 10.6019/PXD028933 and in Zenodo under the following accession codes—10.5281/zenodo.4031852 (https://zenodo.org/record/4031852#.YR8S7i2cZ-U). The RNA-seq data generated in this study have been deposited in Annotare (Array Express) under the following accession codes—Accession # E-MTAB-10899. The TCGA data can be found at https://portal.gdc.cancer.gov/projects/TCGA-GBM. Additional source data are provided with this paper. The Neftel et al. (2019) 10X and Smart-seq2 data were downloaded from the following publicly accessible site: https://singlecell.broadinstitute.org/single_cell/study/SCP393/single-cell-rna-seq-of-adult-and-pediatric-glioblastoma. Source data are provided with this paper.

## Source data

Source Data
