# Supplementary material for: Competitive binding of E3 ligases TRIM26 and WWP2 controls SOX2 in glioblastoma
Source: Nat Commun. 2021 Nov 3;12:6321. doi: 10.1038/s41467-021-26653-6 (PMC8566473; doi:10.1038/s41467-021-26653-6)
Supplement: Supplementary file 1 — Supplementary Information [file 41467_2021_26653_MOESM1_ESM.pdf]

SUPPLEMENTARY FIGURES

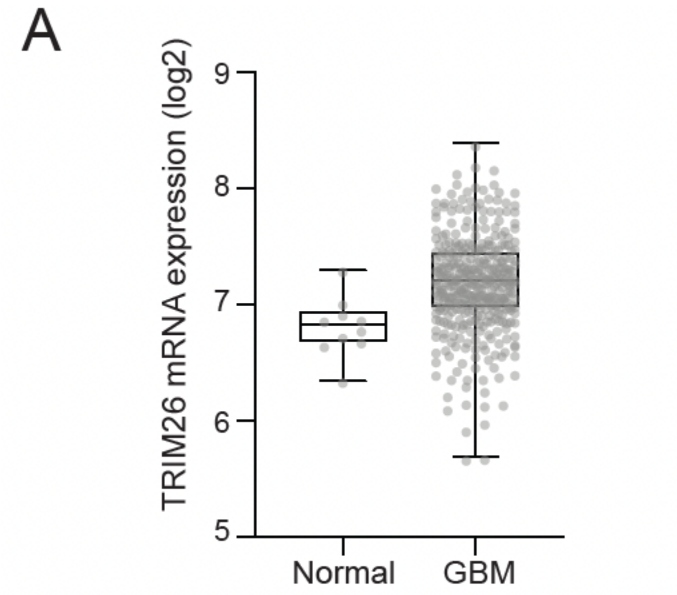

Supplementary Fig. 1: TRIM26 is highly expressed in glioblastoma patient samples, Related to Fig 1.

A. Box plots for TRIM26 mRNA expression in TCGA *IDH1/2* wildtype glioblastoma (n = 372) and normal brain tissue (n = 10) based on the HG-U133A Microarray platform (unpaired t test, $P < 0.01$). The boxes represent the 25th percentile, median, and 75th percentile values. The whiskers represent the maximum and minimum values for the plotted data. See Source Data File for exact P values and statistical parameters.

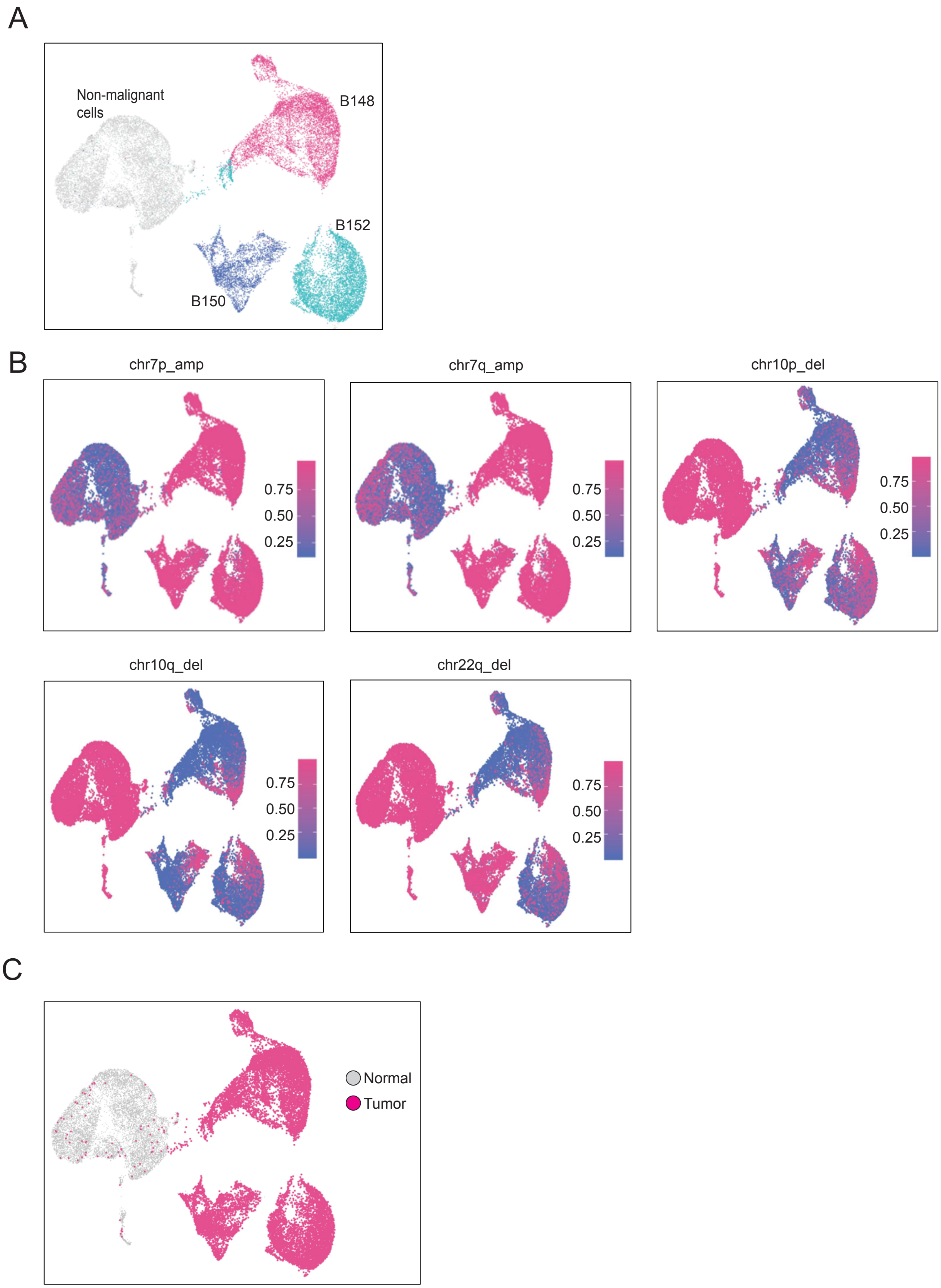
A
Non-malignant cells
B148
B152
B150
B
chr7p_amp
chr7q_amp
chr10p_del
chr10q_del
chr22q_del
0.75
0.50
0.25
C
Normal
Tumor

Supplementary Fig. 2: Identification of malignant cells in glioblastoma scRNA-seq data by expression-based inference of GBM-associated copy number alterations, Related to Fig 1I and Fig 4C

A. t-SNE projection of cells colored by patient from scRNA-seq data for three glioblastoma patients based on k-means clustering and differential expression analysis. Three distinct clusters representing putative tumor cells from each of the three patients (B148, B150, and B152) were identified. An additional cluster containing cells from all three patients was also identified (non-tumor).

B. Cells clustered as in (A) and colored by their z-scored posterior probabilities for the labeled glioblastoma-associated chromosomal copy number alterations. Canonical CNAs were largely restricted to and taken to represent putative tumor cells across the three patients.

C. Final identification of tumor versus normal cells based on an integration of k-means clustering, differential gene expression, and z-scored probabilities for the occurrence of tumor-associated CNAs.

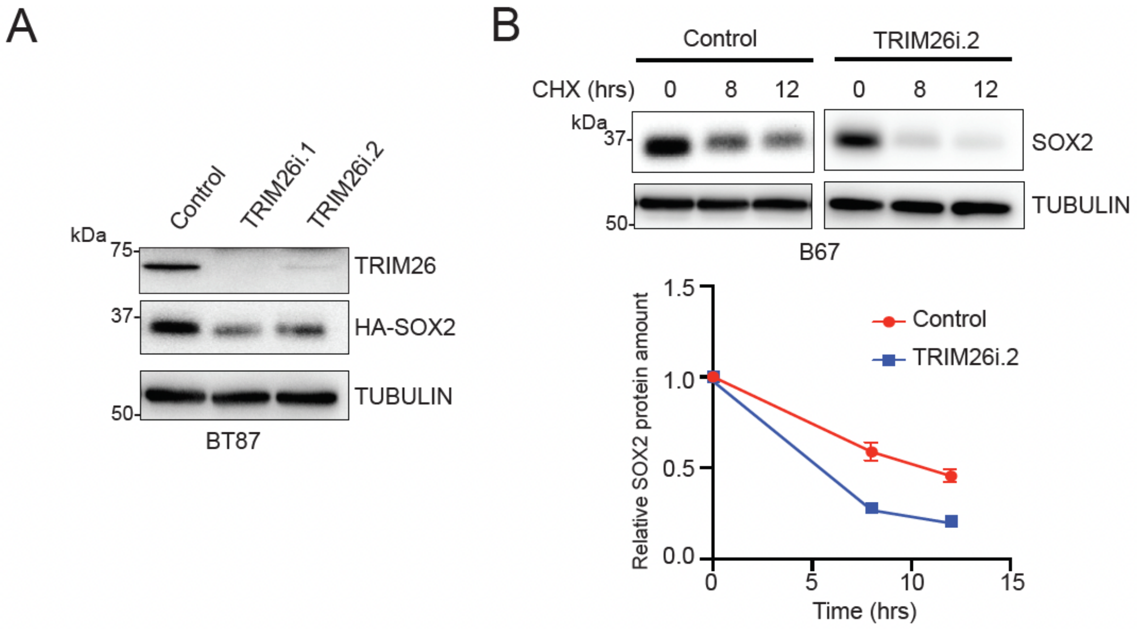

Supplementary Fig. 3: TRIM26 promotes SOX2 protein stability in GSCs, Related to Fig 2.

A. GSCs stably expressing exogenous HA-epitope-tagged SOX2 were transduced with TRIM26 RNAi or control. 5 days later, lysates were analyzed by immunoblotting. Data are representative of three independent experiments.

B. GSCs transduced with TRIM26 RNAi or control were treated 5 days later with 100µM cycloheximide. Lysates were subjected to immunoblotting (top). Protein bands were quantified by pixel intensity measurement using ImageJ. SOX2 expression levels were first normalized to the respective tubulin loading control. Normalized SOX2 protein levels relative to the untreated control (time 0) were subsequently calculated and plotted for each of the 2 experimental conditions (bottom) (n = 3 Control, n = 2 TRIM26i.2)

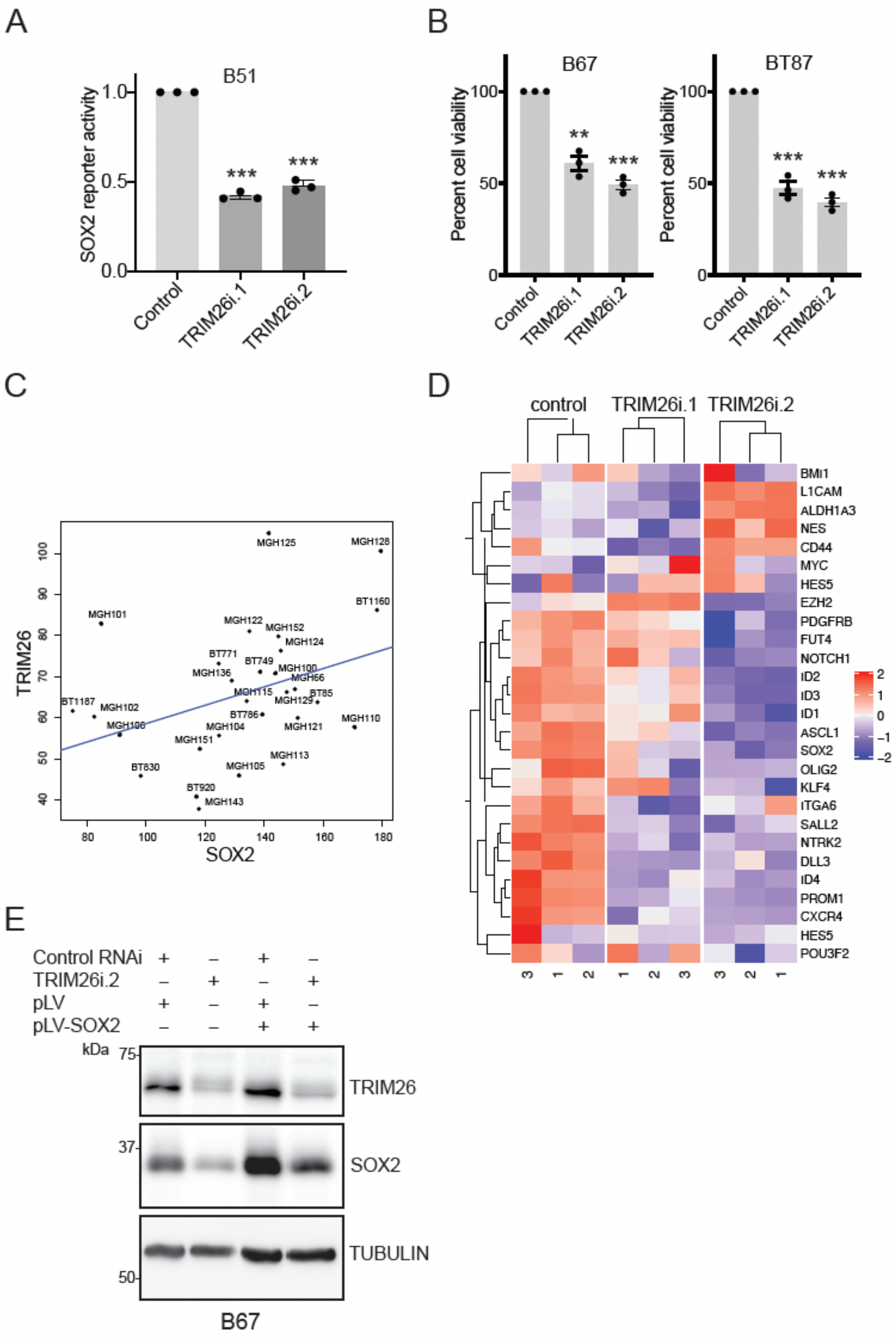
A
B51
SOX2 reporter activity
Control
TRIM26i.1
TRIM26i.2
B
B67
BT87
Percent cell viability
C
TRIM26
SOX2
D
control
TRIM26i.1
TRIM26i.2
BMI1
L1CAM
ALDH1A3
NES
CD44
MYC
HES5
EZH2
PDGFRB
FUT4
NOTCH1
ID2
ID3
ID1
ASCL1
SOX2
OLIG2
KLF4
ITGA6
SALL2
NTRK2
DLL3
ID4
PROM1
CXCR4
HES5
POU3F2
E
Control RNAi
TRIM26i.2
pLV
pLV-SOX2
kDa
TRIM26
SOX2
TUBULIN
B67

Supplementary Fig. 4: TRIM26 loss decreases SOX2 protein, SOX2 reporter activity, GSC viability, and decreases expression of GSC markers, and TRIM26 correlation with SOX2 in independent scRNA-seq data, Related to Fig 2 and Fig 3.

A. GSCs transduced with Firefly luciferase-based SOX2 reporter and Renilla luciferase lentiviruses were transduced with TRIM26 RNAi or control. 5 days later, Firefly and Renilla luciferase activities were measured. Firefly luciferase activity was divided by the Renilla luciferase activity for normalization. Data represent mean +/- SEM (n = 3, ANOVA, ***P < 0.0001). See Source Data File for exact P values and statistical parameters.
B. GSCs transduced with TRIM26 RNAi or control were grown as neurospheres and subjected 14 days later to a cell viability assay (luminescence-based ATP assay). Data represent mean +/- SEM (n=3, ANOVA, **P < 0.001, ***P < 0.0001). See Source Data File for exact P values and statistical parameters.
C. An independent scRNA-seq dataset (Smart-seq2 data, Neftel et al, *Cell*, 2019)[27] was filtered for malignant cells only, and the pseudobulk value for each gene calculated. TRIM26 and SOX2 showed a trend towards correlation (n = 28 tumors, linear regression, $R^2 = 0.11$, $P = 0.0508$).
D. B67 GSCs transduced with TRIM26 RNAi or control were harvested for total RNA 5 days later and subjected to RNA-seq. 27 GSC marker genes were analyzed in this dataset by heatmap (n=3).
E. GSCs stably expressing exogenous SOX2 were transduced with TRIM26 RNAi or control, and 5 days later, lysates were analyzed by immunoblotting with indicated antibodies. Data are representative of three independent experiments.

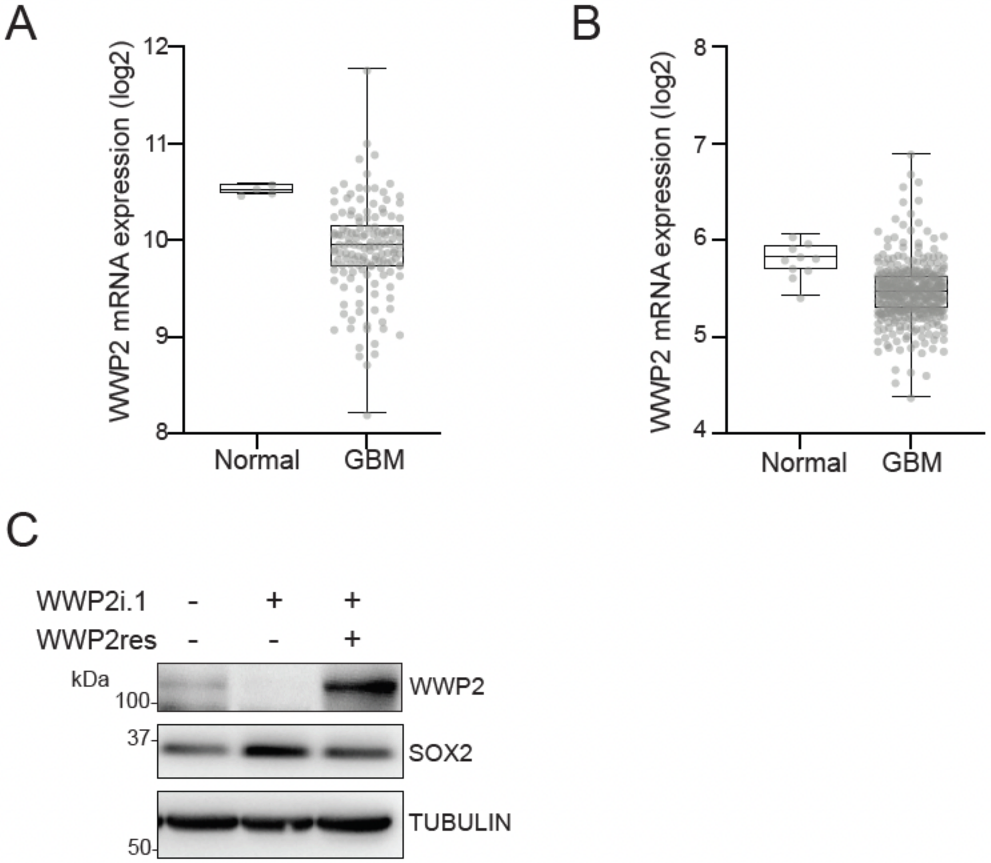

Supplementary Fig. 5: WWP2 is downregulated in glioblastoma relative to normal brain samples and regulates SOX2 protein levels in GSCs, Related to Fig 4.

A. Box plots for WWP2 mRNA expression in TCGA *IDH* wildtype glioblastoma (n = 142) and normal brain tissue (n = 4) based on the RNA-seq platform. WWP2 expression is lower in glioblastoma specimens compared to normal brain tissue (unpaired t-test, $P < 0.05$). The boxes represent the 25th percentile, median, and 75th percentile values. The whiskers represent the maximum and minimum values for the plotted data See Source Data File for exact *P* values and statistical parameters.

B. Box plots for WWP2 mRNA expression in TCGA IDH wildtype glioblastoma (n = 372) and normal brain tissue (n = 10) based on the HG-U133A Microarray platform. WWP2

expression is lower in glioblastoma specimens compared to normal brain tissue (unpaired t-test, $P < 0.005$). The boxes represent the 25th percentile, median, and 75th percentile values. The whiskers represent the maximum and minimum values for the plotted data See Source Data File for exact P values and statistical parameters.

C. GSCs transduced with WWP2 RNAi or control were transduced 2 days later with either empty vector or a WWP2 cDNA rescue (WWP2res) construct. 4 days later, lysates were subjected to immunoblotting. Data are representative of three independent experiments.

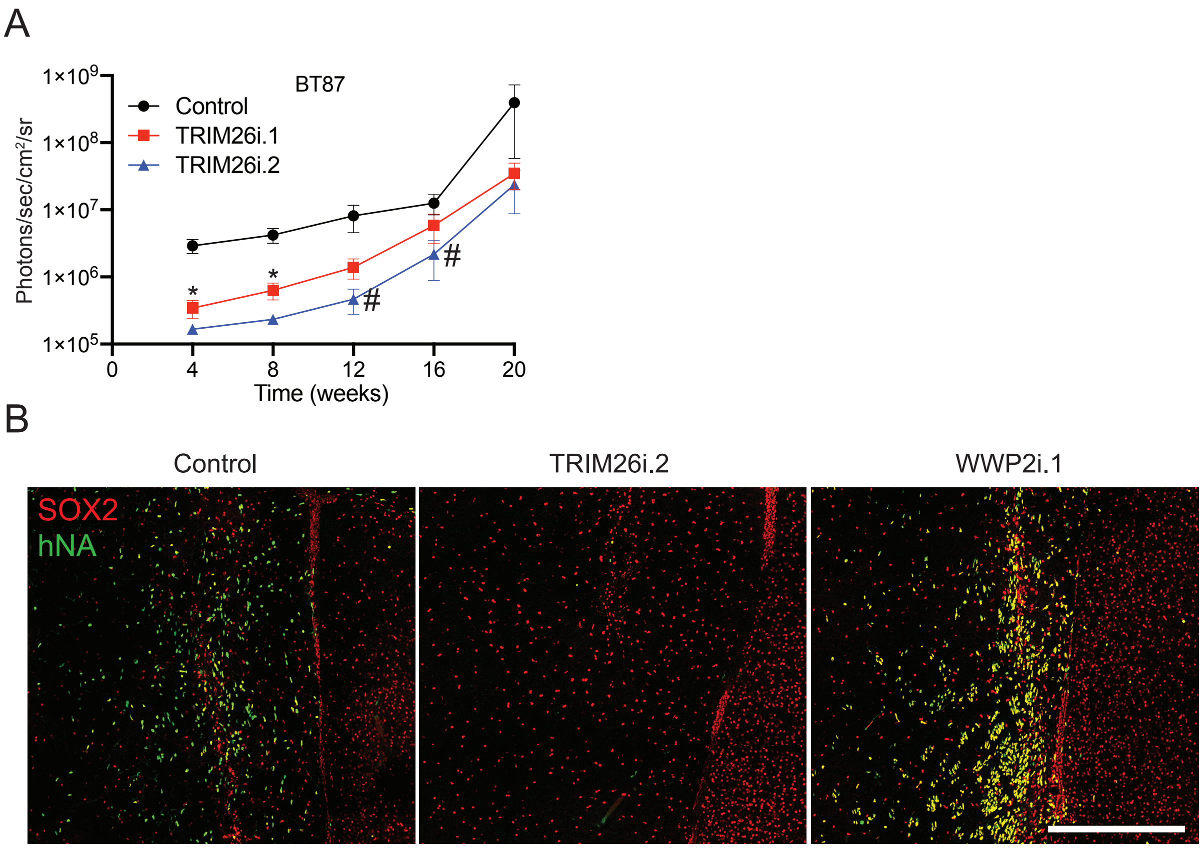

Supplementary Fig. 6: *In vivo* tumorigenicity in TRIM26 RNAi and WWP2 RNAi GSCs, Related to Fig 6.

A. GSCs stably expressing Firefly luciferase were transduced with TRIM26 RNAi or control. 6 days post-transduction, $2.5x10^5$ GSCs were injected into the brains of mice. Bioluminescent live imaging (BLI) was performed. Data represent mean ± SEM (Control: n = 12 for 4, 8 weeks; 6 for 12-20 weeks. TRIM26i.1: n = 11 for 4, 8 weeks; 5 for 12-20 weeks. TRIM26i.2: n = 13 for 4, 8 weeks; 7 for 12-20 weeks. ANOVA, *$P < 0.001$ for both RNAi compared to control, #$P < 0.04$ for TRIM26i.2 only). See Source Data File for exact P values and statistical parameters.

B. $5x10^4$ MGG8 GSCs transduced with TRIM26 RNAi, WWP2 RNAi, or control were injected 6 days later into the brains of mice. 4 weeks later, brains were processed for

immunofluorescence using human nuclear antigen and SOX2 antibodies and visualized by confocal microscopy. Bar = 500 μm.

Supplementary Table I: List of SOX2-interacting predicted E3 ligases in glioblastoma stem cells

GENE SYMBOL
TTC3
TRIM26
TRIM56
PRPF19
TCEB3
KCTD15
KCTD21
KLHL22
ITCH
TRIP12
