# Supplementary material for: Competitive binding of E3 ligases TRIM26 and WWP2 controls SOX2 in glioblastoma
Source: Nat Commun. 2021 Nov 3;12:6321. doi: 10.1038/s41467-021-26653-6 (PMC8566473; doi:10.1038/s41467-021-26653-6)
Supplement: Supplementary file 2 — Description of Additional Supplementary Files [file 41467_2021_26653_MOESM2_ESM.pdf]

Description of Additional Supplementary Files

File Name: Supplementary Data 1
Description: Proteomic discovery of SOX2-interacting proteins by anti-SOX2 immunoprecipitation from B36 GSC lysates followed by LC-MS/MS.

File Name: Supplementary Data 2
Description: RNA-sequencing analysis of GSCs transduced with TRIM26 RNAi. B67 GSCs transduced with TRIM26 RNAi or control were harvested for total RNA and subjected to RNA-seq. Please see Methods for further details. (n=3 per condition, FDR < 0.05).
