# Supplementary material for: Competitive binding of E3 ligases TRIM26 and WWP2 controls SOX2 in glioblastoma
Source: Nat Commun. 2021 Nov 3;12:6321. doi: 10.1038/s41467-021-26653-6 (PMC8566473; doi:10.1038/s41467-021-26653-6)
Supplement: Supplementary file 5 — Reporting Summary [file 41467_2021_26653_MOESM5_ESM.pdf]

nature research

Corresponding author(s): Hiroko Yano, Albert H. Kim, NCOMMS-20-36159A

Last updated by author(s): 8/18/2021

# Reporting Summary

Nature Research wishes to improve the reproducibility of the work that we publish. This form provides structure for consistency and transparency in reporting. For further information on Nature Research policies, see our Editorial Policies and the Editorial Policy Checklist.

## Statistics

For all statistical analyses, confirm that the following items are present in the figure legend, table legend, main text, or Methods section.

| n/a | Confirmed | |
|---|---|---|
| ☐ | ☒ | The exact sample size (*n*) for each experimental group/condition, given as a discrete number and unit of measurement |
| ☐ | ☒ | A statement on whether measurements were taken from distinct samples or whether the same sample was measured repeatedly |
| ☐ | ☒ | The statistical test(s) used AND whether they are one- or two-sided<br>*Only common tests should be described solely by name; describe more complex techniques in the Methods section.* |
| ☐ | ☒ | A description of all covariates tested |
| ☐ | ☒ | A description of any assumptions or corrections, such as tests of normality and adjustment for multiple comparisons |
| ☐ | ☒ | A full description of the statistical parameters including central tendency (e.g. means) or other basic estimates (e.g. regression coefficient) AND variation (e.g. standard deviation) or associated estimates of uncertainty (e.g. confidence intervals) |
| ☐ | ☒ | For null hypothesis testing, the test statistic (e.g. *F*, *t*, *r*) with confidence intervals, effect sizes, degrees of freedom and *P* value noted<br>*Give P values as exact values whenever suitable.* |
| ☒ | ☐ | For Bayesian analysis, information on the choice of priors and Markov chain Monte Carlo settings |
| ☒ | ☐ | For hierarchical and complex designs, identification of the appropriate level for tests and full reporting of outcomes |
| ☐ | ☒ | Estimates of effect sizes (e.g. Cohen's *d*, Pearson's *r*), indicating how they were calculated |

*Our web collection on statistics for biologists contains articles on many of the points above.*

## Software and code

Policy information about availability of computer code

| | |
|---|---|
| Data collection | Microsoft Excel v16.41, Prism 8 and 9, Imaris 9.7.2, Typhoon FLA 9500 Version 1.1, Living Image 2.60.1 (IVIS), GeneSys 1.6.4.0, Gen5 3.8.1.0, |
| Data analysis | Microsoft Excel v16.41,Prism 8 and 9, Imaris 9.7.2, Typhoon FLA 9500 Version 1.1, http://bioinf.wehi.edu.au/software/elda/, Cell Ranger pipeline (10x Genomics, default settings, Version 3.0.1), Seurat R package (V3), CONICSmat package for R, Living Image 2.60.1, Enrichr (https://maayanlab.cloud/Enrichr/), X2K (https://maayanlab.cloud/X2K/), ImageJ 1.53k |

For manuscripts utilizing custom algorithms or software that are central to the research but not yet described in published literature, software must be made available to editors and reviewers. We strongly encourage code deposition in a community repository (e.g. GitHub). See the Nature Research guidelines for submitting code & software for further information.

## Data

Policy information about availability of data

All manuscripts must include a data availability statement. This statement should provide the following information, where applicable:

- Accession codes, unique identifiers, or web links for publicly available datasets
- A list of figures that have associated raw data
- A description of any restrictions on data availability

single cell RNA-seq data: Zenodo DOI: 10.5281/zenodo.4031852 as well as Annotare (Array Express) Accession #: E-MTAB-9435
Proteomic data: Zenodo DOI: 10.5281/zenodo.4031852
RNA-seq data: Annotare (Array Express) Accession #: E-MTAB-10899
TCGA data: https://portal.gdc.cancer.gov/projects/TCGA-GBM
All other data are included in the main and supplementary figures.

# Field-specific reporting

Please select the one below that is the best fit for your research. If you are not sure, read the appropriate sections before making your selection.

☒ Life sciences ☐ Behavioural & social sciences ☐ Ecological, evolutionary & environmental sciences

For a reference copy of the document with all sections, see nature.com/documents/nr-reporting-summary-flat.pdf

# Life sciences study design

All studies must disclose on these points even when the disclosure is negative.

| | |
|---|---|
| Sample size | Sample size is based on effect sizes from prior publications. Unless otherwise stated, experiments were carried out three or more times in three biologically independent samples. |
| Data exclusions | No data exclusions. |
| Replication | Experiments were performed in three or more independent biological replicates. Data were reproducible. |
| Randomization | Samples, cells, and mice used in all experiments were randomized. |
| Blinding | For all experiments, investigators were blinded as to group allocation during data collection and analysis. For all mouse experiments, experiments were double-blinded--in regard to condition upon cell injection into mice and in regard to endpoints of live bioluminescence and neurological deficit-free survival. |

# Reporting for specific materials, systems and methods

We require information from authors about some types of materials, experimental systems and methods used in many studies. Here, indicate whether each material, system or method listed is relevant to your study. If you are not sure if a list item applies to your research, read the appropriate section before selecting a response.

## Materials & experimental systems

| n/a | Involved in the study | |
|---|---|---|
| ☐ | ☒ | Antibodies |
| ☐ | ☒ | Eukaryotic cell lines |
| ☒ | ☐ | Palaeontology and archaeology |
| ☐ | ☒ | Animals and other organisms |
| ☐ | ☒ | Human research participants |
| ☒ | ☐ | Clinical data |
| ☒ | ☐ | Dual use research of concern |

## Methods

| n/a | Involved in the study | |
|---|---|---|
| ☒ | ☐ | ChIP-seq |
| ☒ | ☐ | Flow cytometry |
| ☒ | ☐ | MRI-based neuroimaging |

## Antibodies

Antibodies used

Immunoblotting antibodies:
1) Anti-SOX2, rabbit monoclonal, clone D6D9- Cat # 3579 (Cell Signaling Technologies).
2) Anti-alpha-Tubulin, mouse monoclonal, clone B-5-1-2- Cat # T5168 (Sigma).
3) Anti-HA.11 epitope tag antibody, mouse monoclonal, clone 16B12- Cat # 901502 (BioLegend).
4) Anti-HA, rabbit monoclonal C29F4, Cat #3724 (Cell SIgnaling Technology)
5) Anti-Myc, mouse monoclonal, clone 4A6, Cat #05-724, (Millipore Sigma)
6) Anti-GFP, rabbit polyclonal- Cat # A6455 (ThermoFisher Scientific).
7) Anti-TRIM26, mouse monoclonal, clone A-7- Cat # sc-393832 (Santa Cruz Biotechnology).
8) Anti-Beta-Actin, mouse monoclonal, clone C4- Cat # sc-47778 (Santa Cruz Biotechnology).
9) Anti-Flag, mouse monoclonal, clone M2- Cat # F3165 (Sigma).
10) Anti-His tag, rabbit polyclonal- Cat # 2365 (Cell Signaling Technology).
11) Anti-FZR1(CDH1), mouse monoclonal, clone DH01 (DCS-266)- Cat # ab3242 (AbCam).
12) Anti-Cullin-4A, rabbit polyclonal- Cat # C0371 (Sigma).
13) Anti-EDD1(UBR5), rabbit polyclonal- Cat # A300-573A (Bethyl Labs).
14) Anti-WWP2, rabbit polyclonal- Cat # A302-935A (Bethyl Labs).

Immunoprecipitation antibodies:
1) Anti-SOX2, goat polyclonal- Cat # 17320 (Santa Cruz Biotechnology).
2) Anti-SOX2, monoclonal rabbit anti-SOX2 D9B8N (Cell Signaling Technology)
3) Anti-GFP, mouse monoclonal, clone 3E6- Cat # A11120 (ThermoFisher Scientific).
4) Anti-Myc, mouse monoclonal, clone 9E10, Cat #05-419 (Millipore Sigma)
5) Anti-Myc, mouse monoclonal, clone 4A6, Cat #05-724, (Millipore Sigma)

6) Normal Goat IgG, polyclonal. Cat # NI02 (EMD-Millipore).

Immunofluorescence antibodies:
1) monoclonal rabbit anti-Sox2 SP76 , Cat # 371R-15 (Cell Marque, from Millipore Sigma)
2) monoclonal mouse anti-Human nuclear antigen 235-1 (Novus Biological)
3) Goat Anti-Rb Alexa 568, Cat # A-11011 (Invitrogen)
4) Goat Anti-Ms Alexa 647, Cat # A-21235 (Invitrogen)

Validation

Immunoblotting antibodies:
1) Anti-SOX2, rabbit monoclonal- Cat # 3579 (Cell Signaling Technologies). Recommended by manufacturer for immunoblotting for detection of human SOX2, among other species, at 1:1000 dilution. Validated by RNAi-mediated SOX2 knockdown as well as SOX2 overexpression followed by immunoblotting in human breast cancer cell line MDA-MB-231 (Oncogene. 2017 Sep 7;36(36):5199-5211).
2) Anti-alpha-Tubulin, mouse monoclonal- Cat # T5168 (Sigma). Recommended by manufacturer for immunoblotting for detection of human alpha-tubulin, among other species, at 1:4000 dilution. Validated in alpha-tubulin immunoprecipitation followed by immunoblotting experiments relative to control in C. elegans neurons (PLoS Genet. 2010 Jan; 6(1): e1000820).
3) Anti-HA.11 epitope tag antibody, mouse monoclonal- Cat # 901502 (BioLegend). Recommended by manufacturer for immunoblotting at a dilution to 1:2,000-1:20,000 for detection of HA-epitope-tagged recombinant proteins. Validated by immunoblots for HA-MeCP2 in transgenic versus wildtype monkey brain tissues (Nature 2016 Feb 4;530(7588):98-102.).
4) Anti-HA, rabbit monoclonal C29F4, Cat #3724 (Cell SIgnaling Technology). Recommended by manufacturer for immunoblotting for detection of HA epitope at 1:1,000 dilution. Validated by immunoblots in transfected vs. control MCF10A cell (Cell. 2020 Jun 25; 181(7): 1596–1611.e27).
5) Anti-Myc, mouse monoclonal, clone 4A6, Cat #05-724, (Millipore Sigma).Recommended by manufacturer for immunoblotting for detection of myc epitope at 1:1,000 dilution. Validated by immunoblots in budding yeast with and without inserted tag at endogenous locus (Nature. 2013 May 2; 497(7447): 118–121).
6) Anti-GFP, rabbit polyclonal- Cat # A6455 (ThermoFisher Scientific). Recommended by manufacturer for detection of GFP-tagged recombinant proteins by immunoblot at 1:5,000 dilution. Validated by immunoblots for GFP-CDC20 expression in transfected versus control HEK293 cells (Cell Rep 2015 Jun 23;11(11):1809-21).
7) Anti-TRIM26, mouse monoclonal- Cat # sc-393832 (Santa Cruz Biotechnology). Recommended by manufacturer for immunoblotting for detection of human TRIM26, among other species, at 1:100-1,000 dilution. The antibody is validated in the current manuscript immunoblots for TRIM26 expression in 3 glioblastoma stem cell lines following RNAi-mediated knockdown of TRIM26 with 2 distinct TRIM26-targeting RNAi constructs relative to control (Fig 2A).
8) Anti-Beta-Actin, mouse monoclonal- Cat # sc-47778 (Santa Cruz Biotechnology). Recommended by manufacturer for immunoblotting for detection of human Beta-Actin, among other species, at 1:100-1,000 dilution. Validated by the previously described interaction of SHTN1 and Actin using co-immunoprecipitation followed by immunoblotting and appropriate controls (J Mol Biol 2020 Jun 26;432(14):4154-4166).
9) Anti-Flag-M2, mouse monoclonal- Cat # F3165 (Sigma). Recommended by manufacturer for detection of Flag-tagged recombinant proteins by immunoblot at a dilution of 1:2000. We have previously validated this antibody in GSCs expressing Flag-ID1 compared to empty-vector-transduced cells (Proc Natl Acad Sci U S A 2016 Dec 20;113(51)).
10) Anti-His tag, rabbit polyclonal- Cat # 2365 (Cell Signaling Technology). Recommended by manufacturer for detection of His-epitope-tagged recombinant proteins by immunoblot at a dilution of 1:1,000. Validated by expression and immunoblot detection of His-Ubiquitin in transfected LN229 and U87 GBM cell lines compared to untransfected control cells (Nat Commun 2019 Sep 5;10(1):4013).
11) Anti-FZR1(CDH1), mouse monoclonal- Cat # ab3242 (AbCam). Recommended by manufacturer for immunoblotting for detection of human and mouse FZR1 (CDH1) at a dilution of 1:500. Validated by RNAi-mediated CDH1 knockdown followed by immunoblotting in HeLa cells (Nat Cell Biol. 2010 Jul; 12(7): 686–695.).
12) Anti-Cullin-4A, rabbit polyclonal- Cat # C0371 (Sigma). Recommended by manufacturer for immunoblotting for detection of human Cul-4A. Validated by RNAi-mediated Cul-4A knockdown followed by immunoblotting in NPCs (Nat Commun 2018 Nov 7;9(1):4648).
13) Anti-EDD1(UBR5), rabbit polyclonal- Cat # A300-573A (Bethyl Labs). Recommended by manufacturer for immunoblotting for detection of human and rat UBR5 at 1:2,000-1:10,000 dilution. Validated by the manufacturer using immunoprecipitation and subsequent immunoblot compared to control IgG. Validated by RNAi-mediated UBR5 knockdown followed by immunoblotting in esophageal cancer stem cells (Oncogene 2019 Jun;38(26):5250-5264).
14) Anti-WWP2, rabbit polyclonal- Cat # A302-935A (Bethyl Labs). Recommended by manufacturer for immunoblotting for detection of human WWP2 at 1:2,000-1:10,000 dilution. Validated by the manufacturer using immunoprecipitation and subsequent immunoblot compared to control IgG. Validated by RNAi-mediated WWP2 knockdown followed by immunoblotting in U2OS cells (Genes Dev 2019 Jun 1;33(11-12):684-704).

Immunoprecipitation antibodies:
1) Anti-SOX2, goat polyclonal- Cat # 17320 (Santa Cruz Biotechnology). Recommended by manufacturer for immunoprecipitation of human SOX2, among other species, at 2 microg antibody per 500 microg lysate protein. Validated by SOX2 immunoprecipitation experiments relative to normal IgG control from mouse ESCs (Stem Cells. 2010 Oct; 28(10): 1715–1727).
2) Anti-SOX2, monoclonal rabbit anti-SOX2 D9B8N (Cell Signaling Technology). Recommended by manufacturer for immunoprecipitation of human SOX2, among other species, at 1:100::antibody:lysate volume, which we used at approximately 2 microg antibody per 500 microg lysate. Validated by manufacturer SOX2 immunoprecipitation experiments relative to normal IgG control IgG from F9 cells.
3) Anti-GFP, mouse monoclonal- Cat # A11120 (ThermoFisher Scientific). Recommended by manufacturer for immunoprecipitation of GFP-tagged recombinant proteins at a concentration of 0.1 to 1 microg/ml. Validated by co-immunoprecipitation followed by immunoblotting for the interaction of GFP-Raw with Trc-kinase in Drosophila S2 cells compared to appropriate control (Development 2015 Jan 1;142(1):162-73).
4) Anti-Myc, mouse monoclonal, clone 9E10, Cat# 05-419 (Millipore Sigma). Recommended by manufacturer for immunoprecipitation of myc-tagged recombinant proteins at a concentration of 4 microg per 500 microg lysate. Validated in transfected vs. non-transfected NIH3T3 cells (Sci Signal. 2020 Jan 28;13(616):eaax9730.).
5) Anti-Myc, mouse monoclonal, clone 4A6, Cat #05-724, (Millipore Sigma). Recommended by manufacturer for immunoprecipitation of myc-tagged recombinant proteins. Validated in transfected vs. non-transfected NIH3T3 cells (Sci Signal. 2020 Jan 28;13(616):eaax9730.).

6) Normal Goat IgG, polyclonal. Cat # NI02 (EMD-Millipore). Per manufacturer: "This Normal Goat IgG is validated for use in ELISA Flow Cytometry, Immunoblotting, Immunofluorescence, Immunohistochemistry, and Immunoprecipitation for the detection of Goat IgG, Non-immune."

Immunofluorescence antibodies:
1) monoclonal rabbit anti-Sox2 SP76 , Cat # 371R-15 (Cell Marque, from Millipore Sigma). Recommended by manufacturer for immunofluorescence to detect human Sox2 at an antibody dilution of 1:200. Validated by immunohistochemistry of control vs. SOX2-oxerexpressing Caov3 cells (Cancer Res.2013 Sep 1;73(17):5544-55).
2) monoclonal mouse anti-Human nuclear antigen 235-1 (Novus Biological). Recommended by manufacturer for immunofluorescence to detect a human nuclear antigen at an antibody dilution of 1:500. Validated by immunofluorescence to detect hESCs grafted into chick embryos (Nature. 2018 Jun;558(7708):132-135.).
3) Goat Anti-Rb Alexa 568, Cat # A-11011 (Invitrogen). Recommended by manufacturer for immunofluorescence at an antibody dilution of 1:500. Validated by immunofluorescence for murine hippocampal neurons. (Journal of Neuroscience 25 April 2018, 38 (17) 4093-4103).
4) Goat Anti-Ms Alexa 647, Cat # A-21235 (Invitrogen). Recommended by manufacturer for immunofluorescence at an antibody dilution of 1:500. Validated by immunofluorescence for circulating small cell lung cancer cells. (Nat Commun. 2016 7, 13322).

# Eukaryotic cell lines

Policy information about cell lines

| | |
|---|---|
| Cell line source(s) | The human glioblastoma stem cell lines B36, B51, B66, and B67 were generated in the laboratory from patient tumors. BT87 line was a kind gift from Dr. Sunit Das at University of Toronto. MGG8 line was a kind gift of Dr. Daniel Cahill at the Massachusetts General Hospital. HEK293T cells were from Clontech (now Takara). |
| Authentication | B36, B66, BT87, and MGG8 have been previously characterized (Mao, Gujar et al. Cell Reports. 2015, Gujar et al. Methods Mol Biol. 2018, Sachdeva et al. Cancer Research. 2019, Wakimoto et al. Neuro-Oncology. 2012). B51 and B67 were derived in the laboratory from patient tumor specimens in the same manner as B36, B66 using established standardized methods (Gujar et al. Methods Mol Biol. 2018). All cell lines were authenticated by STR profiling and used for experiments within 20 passages. |
| Mycoplasma contamination | All lines were confirmed negative for mycoplasma using a sensitive, enzyme-based detection kit (Lonza MycoAlert PLUS Assay). |
| Commonly misidentified lines (See ICLAC register) | No commonly misidentified lines were used in this study. |

# Animals and other organisms

Policy information about studies involving animals; ARRIVE guidelines recommended for reporting animal research

| | |
|---|---|
| Laboratory animals | 6-week-old female NOD-SCID gamma mice (Jackson Labs) were used for the study.<br>Mice were housed in static microisolator caging on 1/8 inch corncob bedding with ad libitum access to Lab Diet 5053 chow and autoclaved water. Temperature was maintained between 70° +/- 2° F, humidity within 30-70%, and 12:12 hour dark to light cycle. All mice were free of Pneumonia Virus of Mice (PVM), Reovirus 3 (REO3), Sendai virus, Mycoplasma pulmonis, Minute Virus of Mice (MVM), Theiler's Murine Encephalomyelitis Virus (GDVII), Lymphocytic Choriomeningitis Virus (LCMV), Polyoma Virus, Mouse Rotavirus (EDIM), Ectromelia Virus (Mousepox), Mouse Adenovirus, K Virus, Mouse Parvovirus (MPV), Cytomegalovirus, Mouse Hepatitis Virus (MHV), Clostridium piliforme,Streptococcus pneumonia, Bordetella bronchiseptica, Streptobacillus moniliformis, Corynebacterium kutcheri, Salmonella spp. Citrobacter rodentium, murine pinworms and fur mites. |
| Wild animals | No wild animals were used in this study. |
| Field-collected samples | No field collected samples were used in the study. |
| Ethics oversight | Informed consent was obtained from patients for use of human tissue and cells, and all human tissue-related protocols used in this study were approved by the Institutional Review Board (Washington University). Our animal protocol (21-0083) adheres to NIH and American Association for Laboratory Animal Science (AALAS) guidelines and has been approved by our Institutional Animal Care and Use Committee (IACUC). |

Note that full information on the approval of the study protocol must also be provided in the manuscript.

# Human research participants

Policy information about studies involving human research participants

| | |
|---|---|
| Population characteristics | For human cell lines, six distinct patient lines from glioblastoma tumors were used for experiments (age and sex: B36: 55, M; B51: 43, F; B66: 36, M; B67: 60, F; MGG8: not reported, Neuro Oncol. 2012 Feb; 14(2): 132–144; BT87: not reported, Oncotarget. 2017; 8:82217-82230. For single cell RNA-seq, three patient glioblastoma tumors were used (age and sex: B148: 55, M; B150: 68, F; B152: 71, M). All patient tumors were IDH1/2 WT glioblastomas. |
| Recruitment | Patient samples were obtained through the CNS Tumor Bank Protocol, an IRB-approved protocol at our institution. |
| Ethics oversight | Informed consent was obtained from patients for use of human tissue and cells, and all human tissue-related protocols used in this study were approved by the Institutional Review Board (Washington University) (IRB #201211019). |

Note that full information on the approval of the study protocol must also be provided in the manuscript.
