# Supplementary material for: Competitive binding of E3 ligases TRIM26 and WWP2 controls SOX2 in glioblastoma
Source: Nat Commun. 2021 Nov 3;12:6321. doi: 10.1038/s41467-021-26653-6 (PMC8566473; doi:10.1038/s41467-021-26653-6)
Supplement: Supplementary file 6 — Source Data [file 41467_2021_26653_MOESM6_ESM.zip › Uncropped Images.FINAL.pdf]

FIG 1A

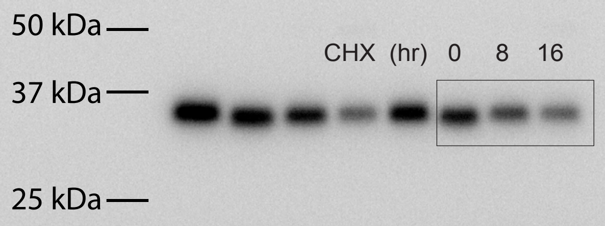

B36

SOX2 ANTIBODY

FIG 1A

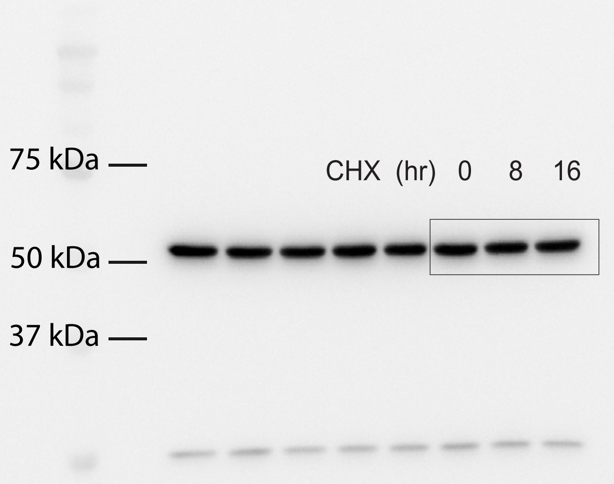

B36

TUBULIN ANTIBODY

FIG 1A

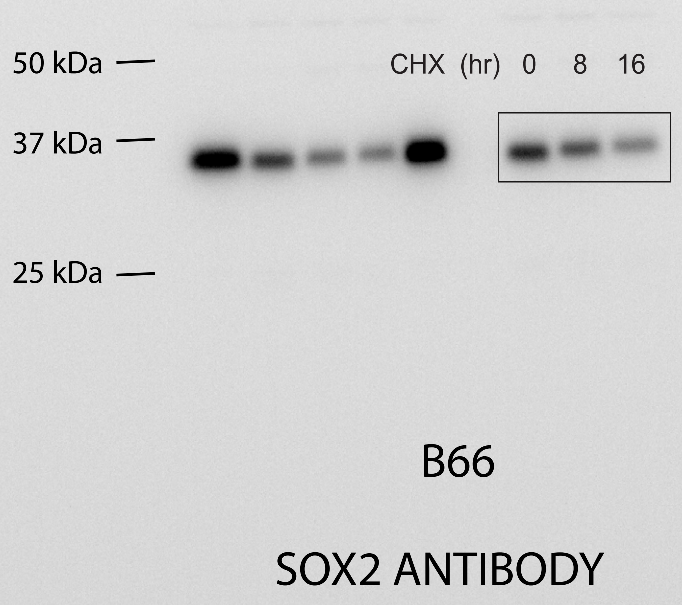

FIG 1A

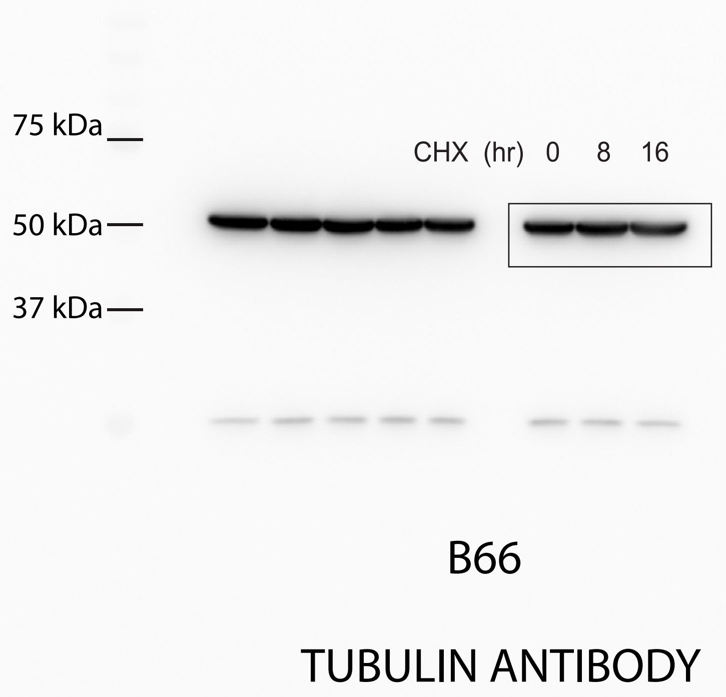

FIG 1A

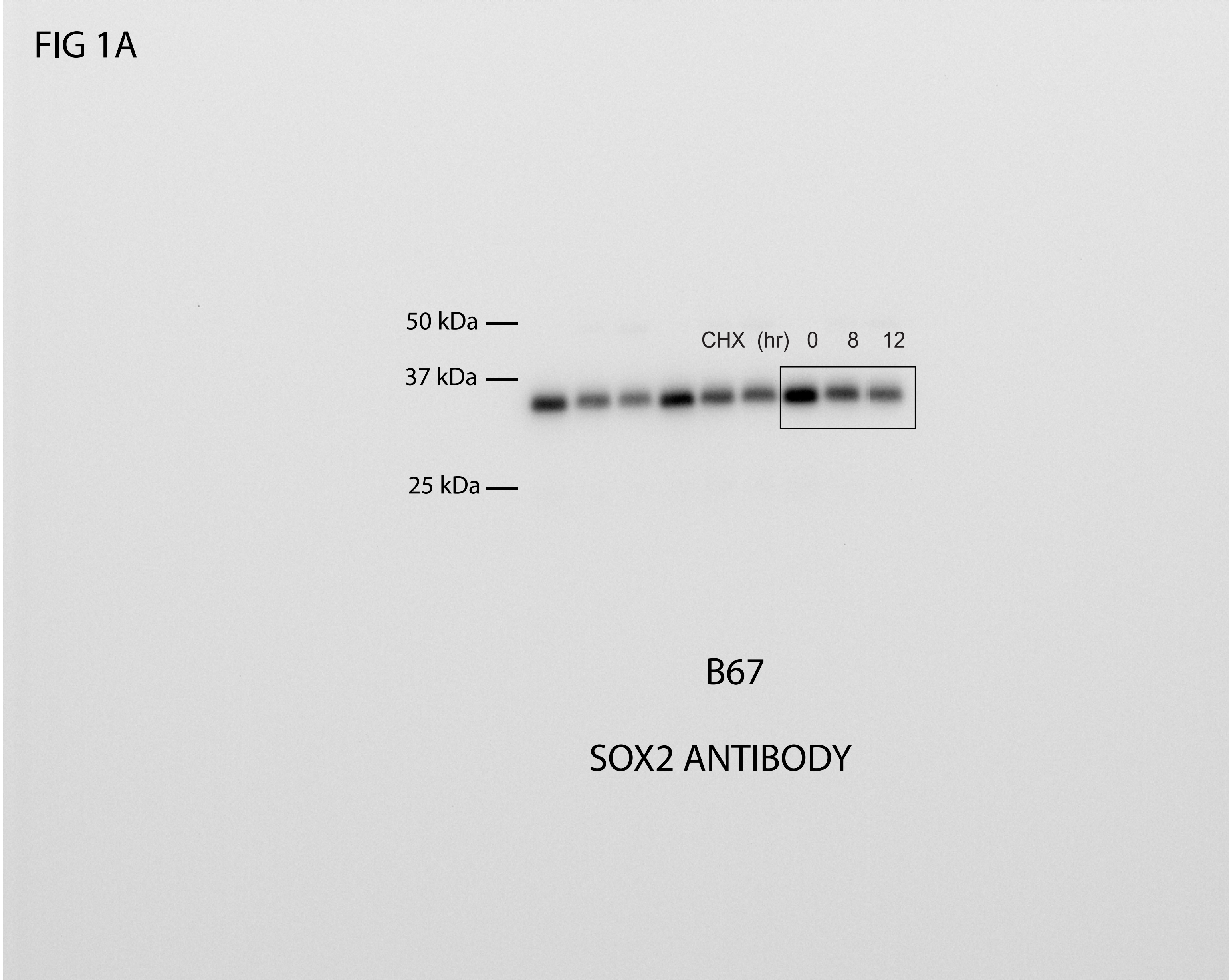

FIG 1A

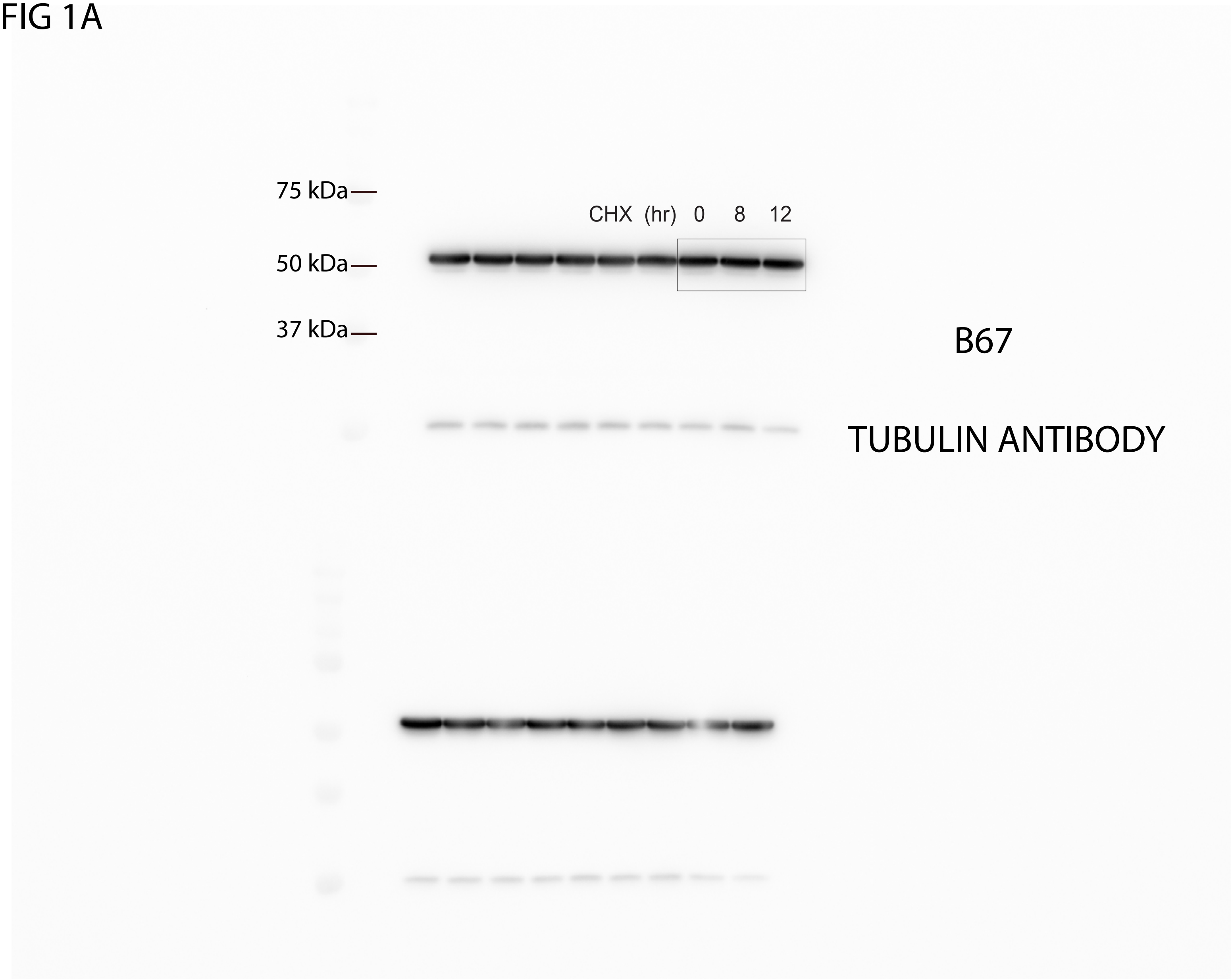

FIG 1A

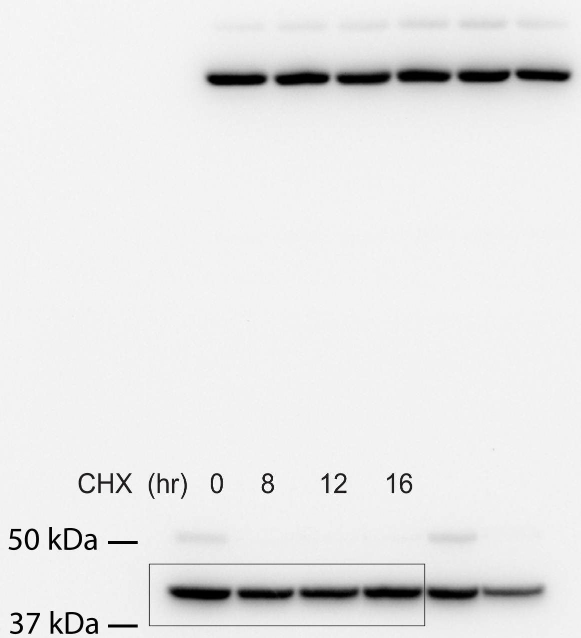

BT87

BETA-ACTIN ANTIBODY

FIG 1A

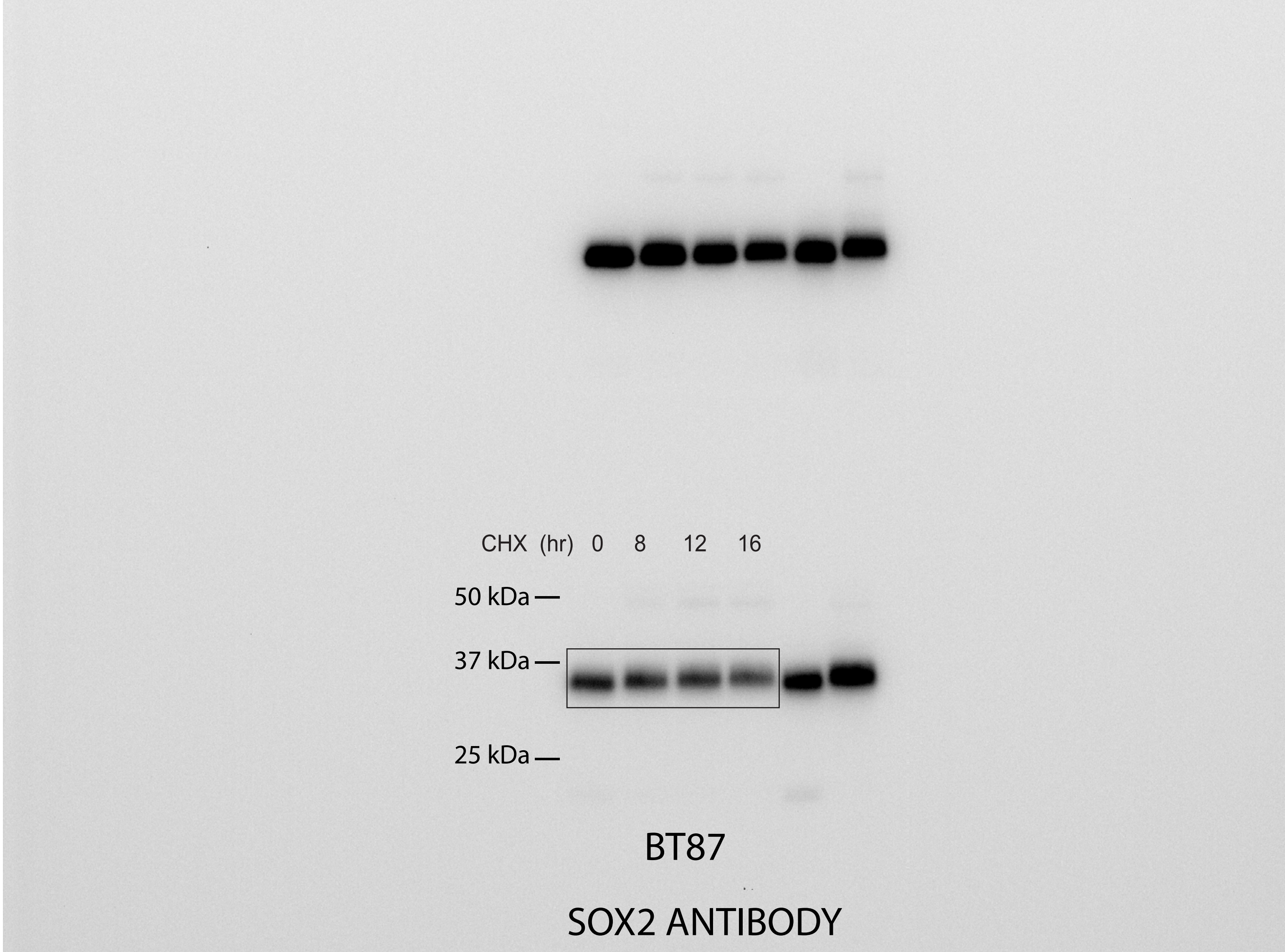

FIG 1C

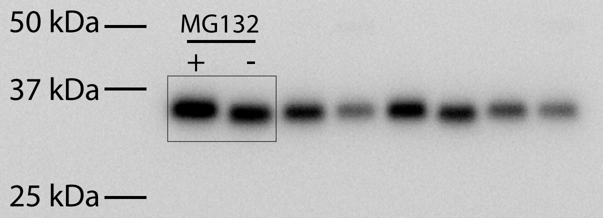

B36

SOX2 ANTIBODY

FIG 1C

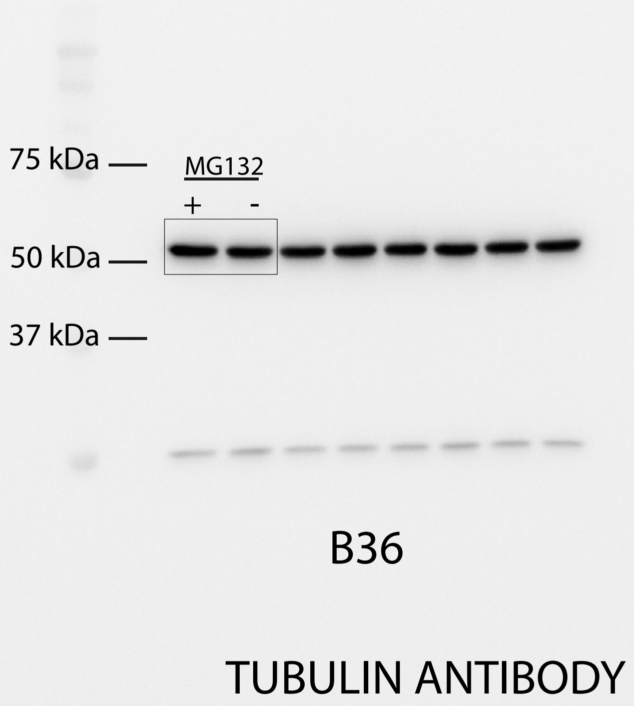

FIG 1C

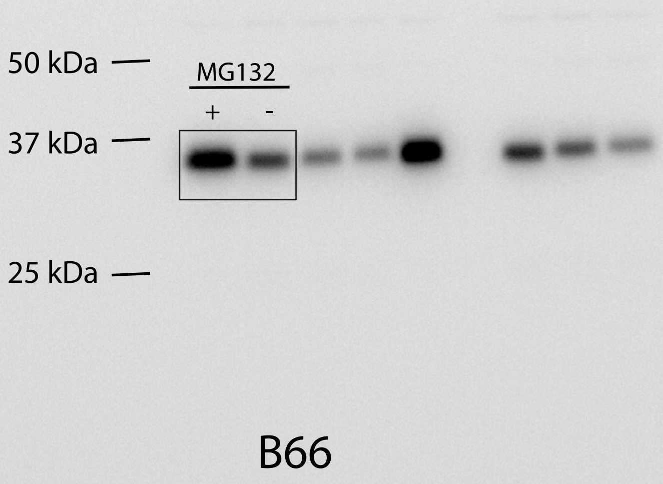

SOX2 ANTIBODY

FIG 1C

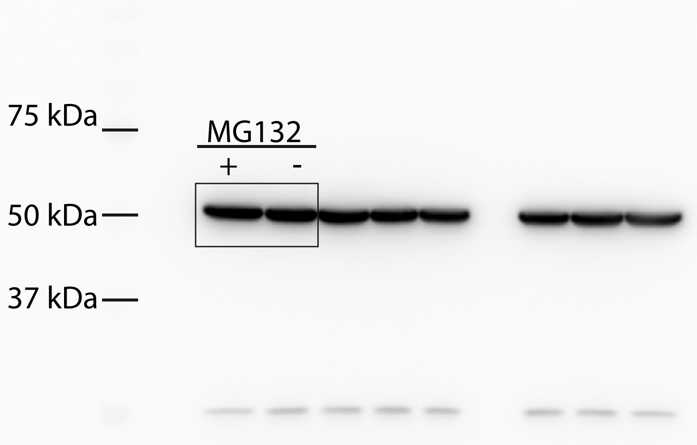

B66

TUBULIN ANTIBODY

FIG 1C

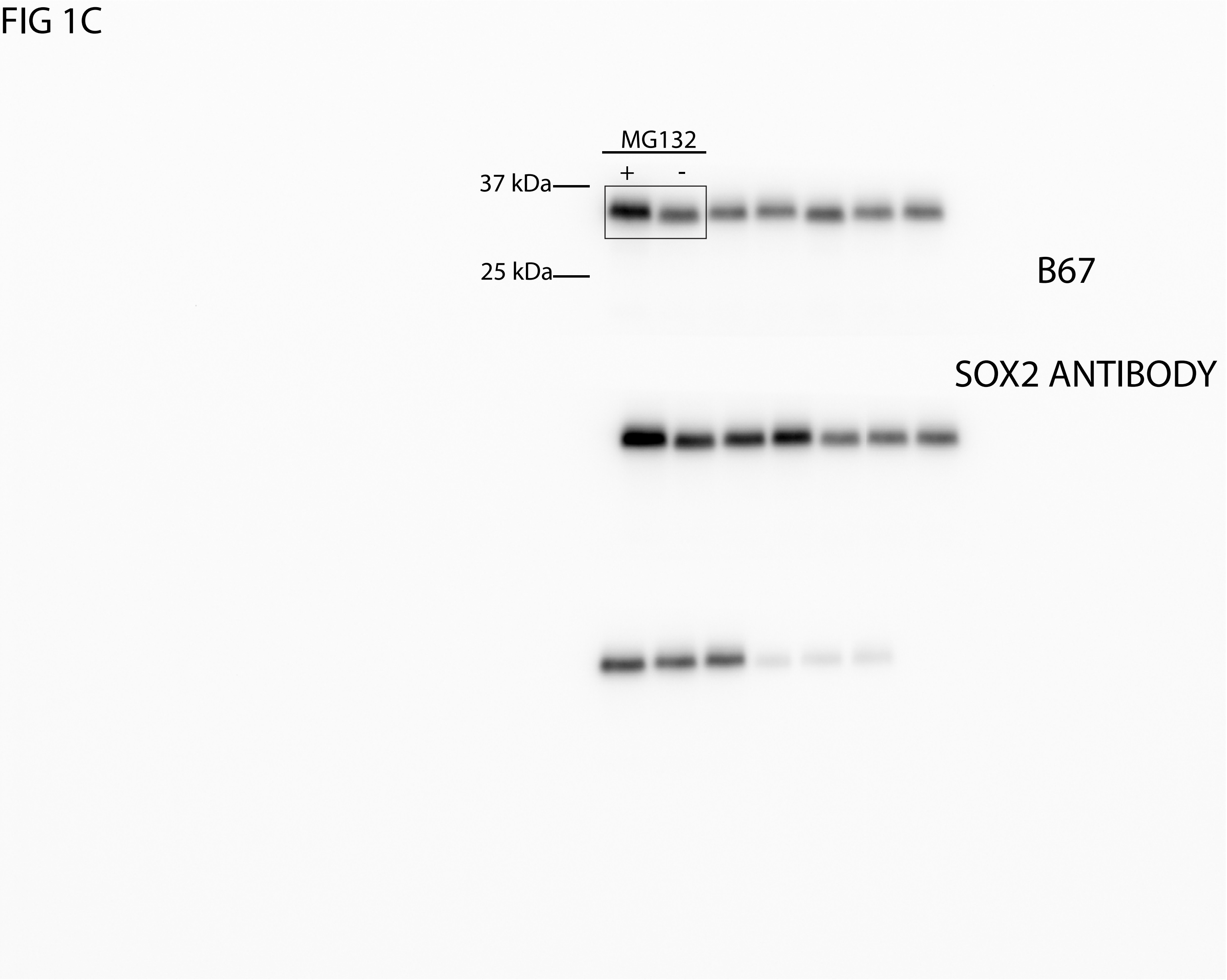

FIG 1C

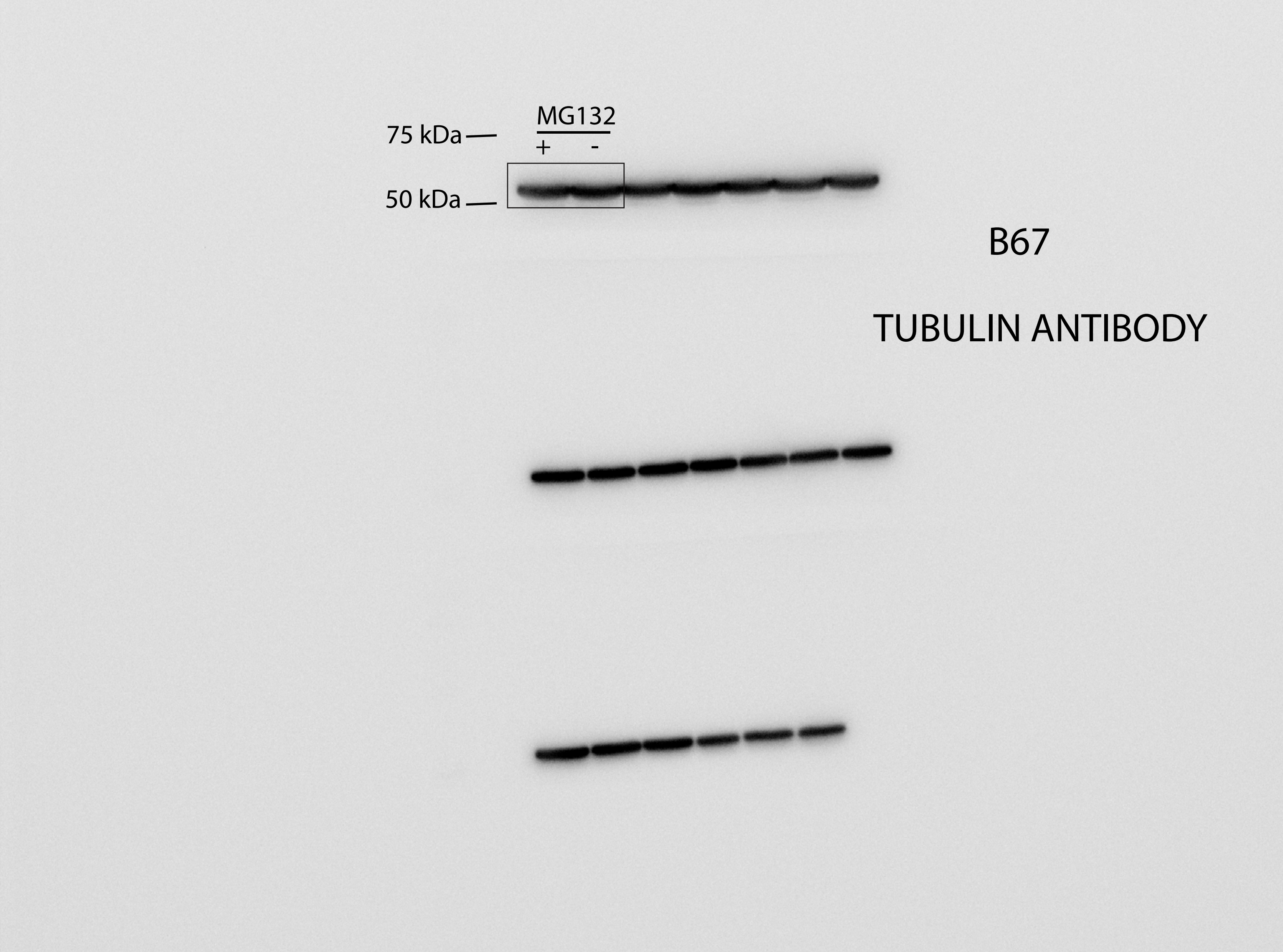

FIG 1C

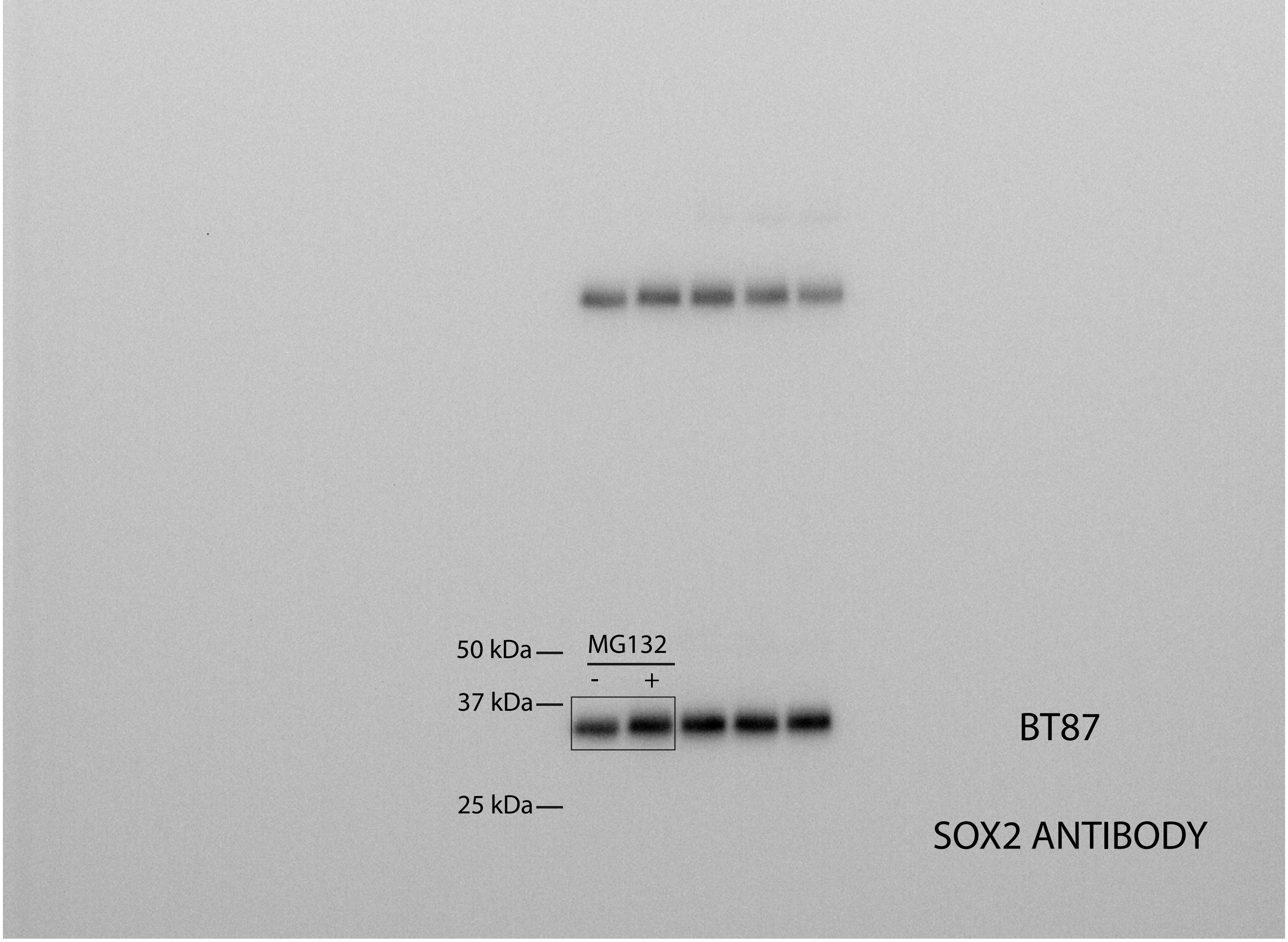

FIG 1C

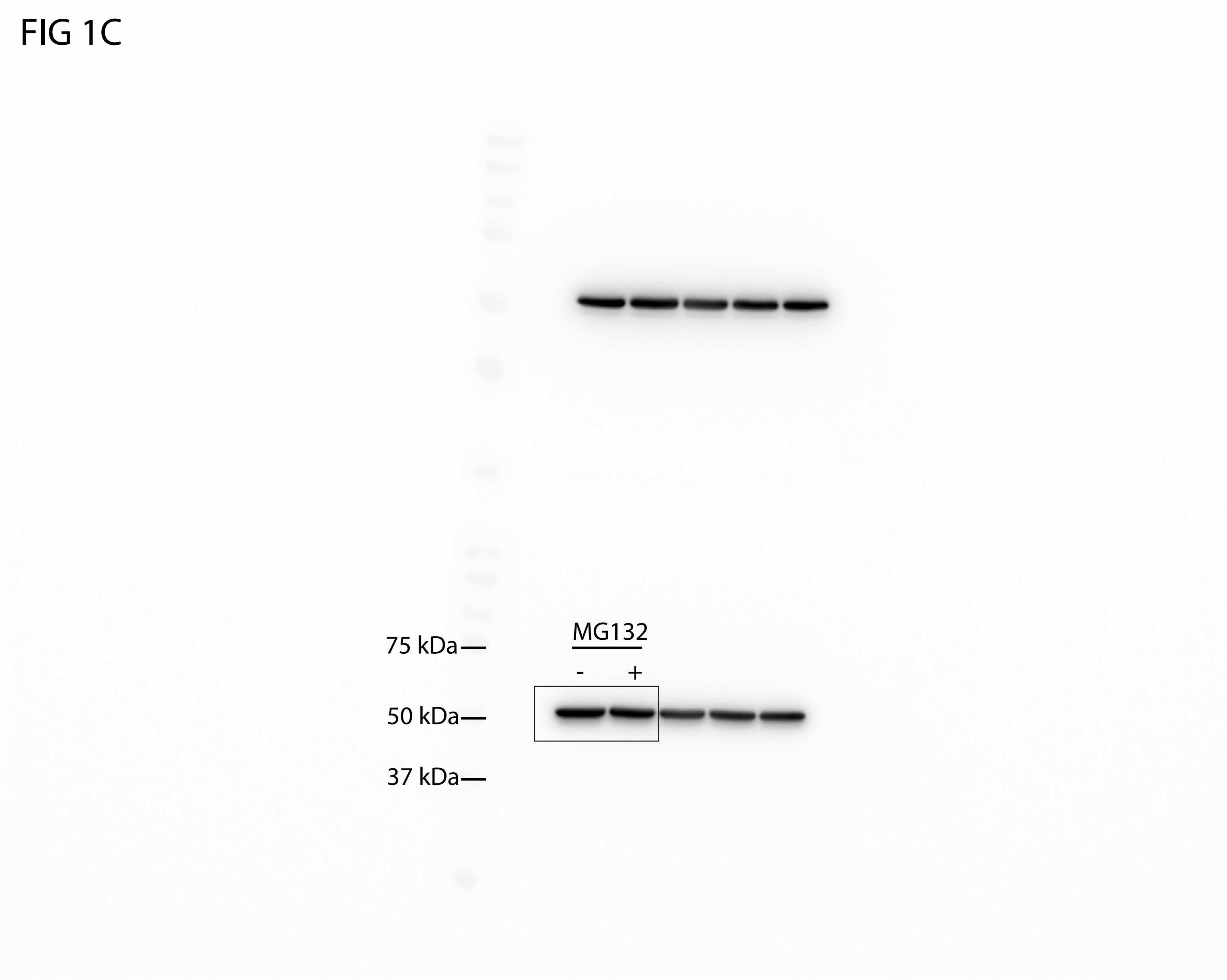

BT87

TUBULIN ANTIBODY

FIG 1F

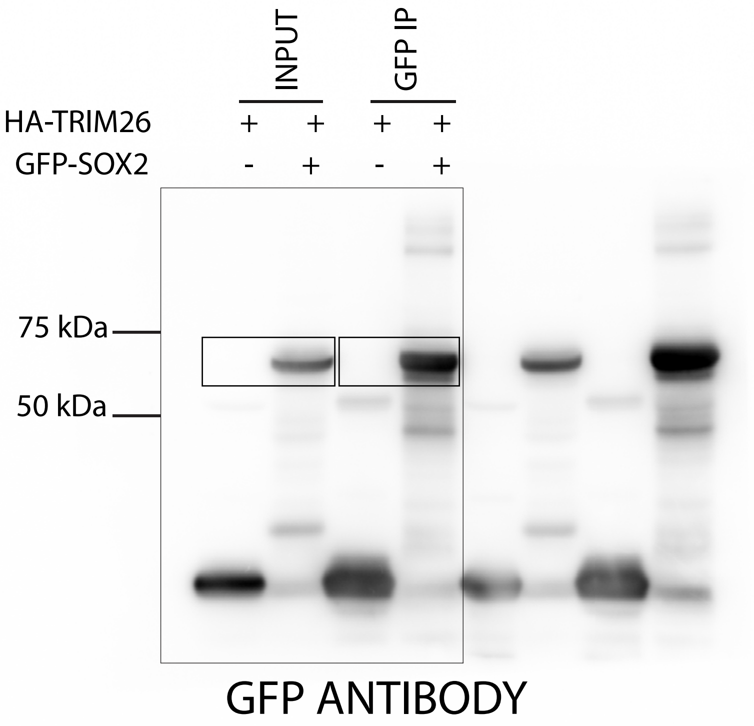

FIG 1F

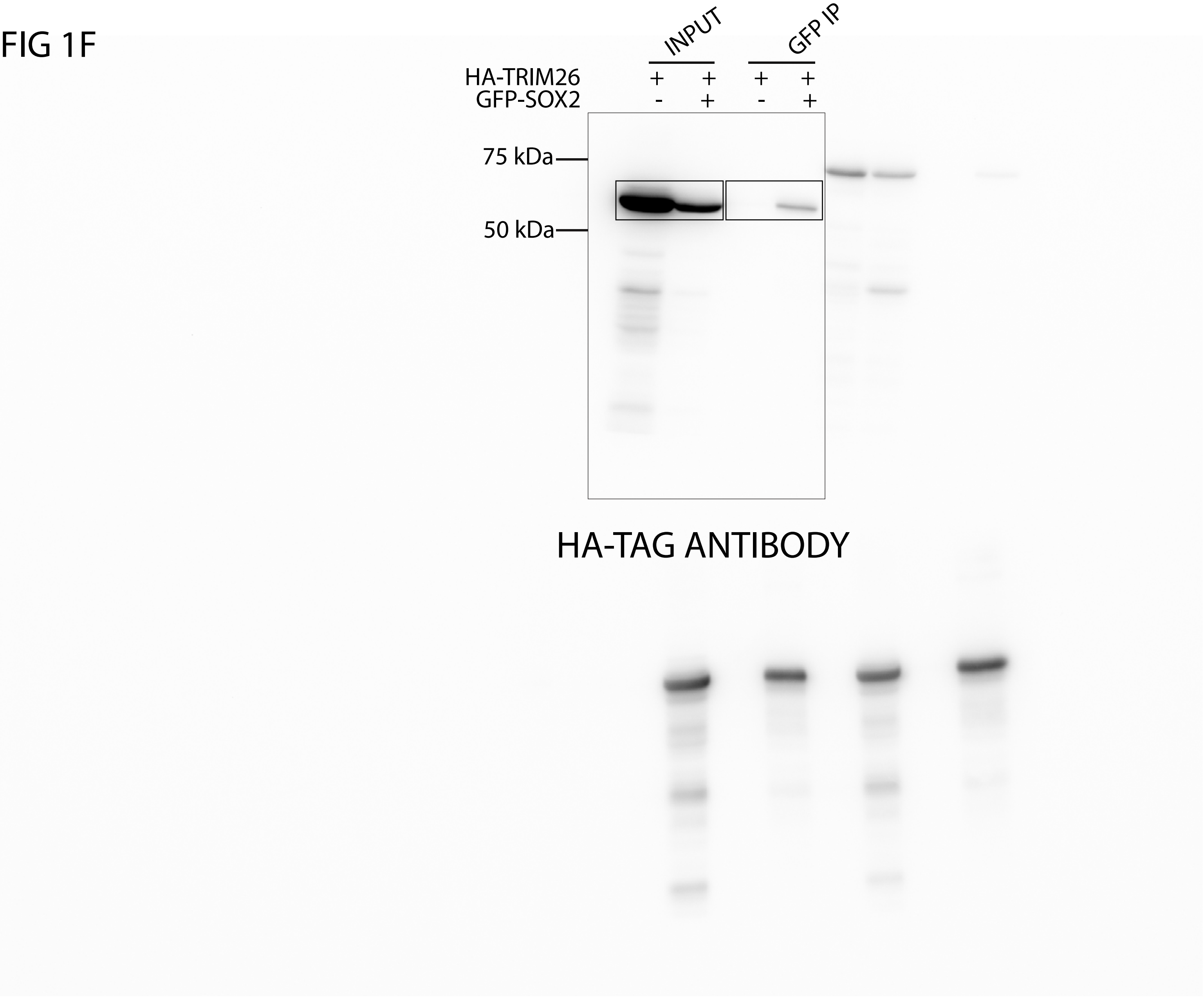

FIG 1G

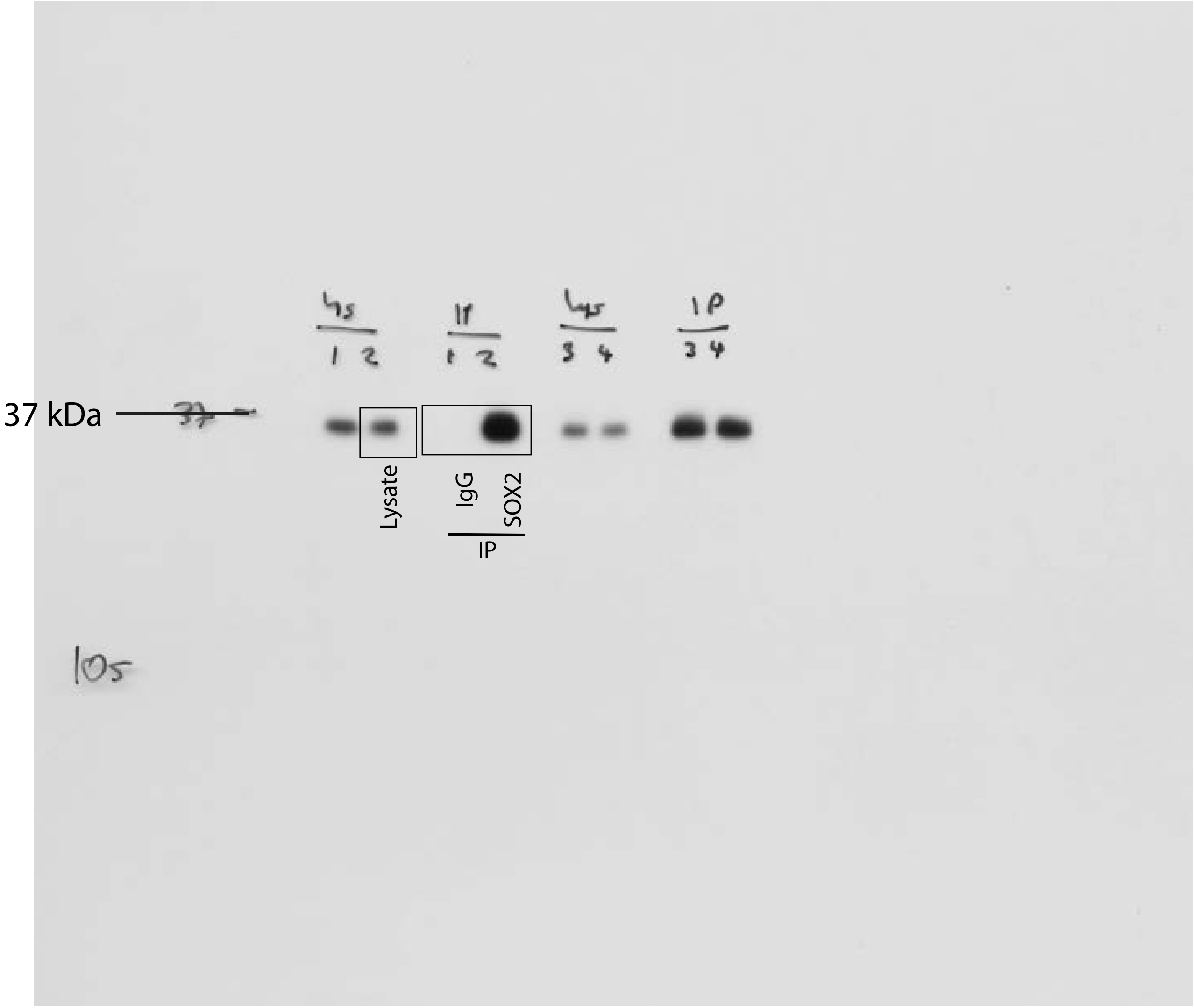

B51

SOX2 ANTIBODY

FIG 1G

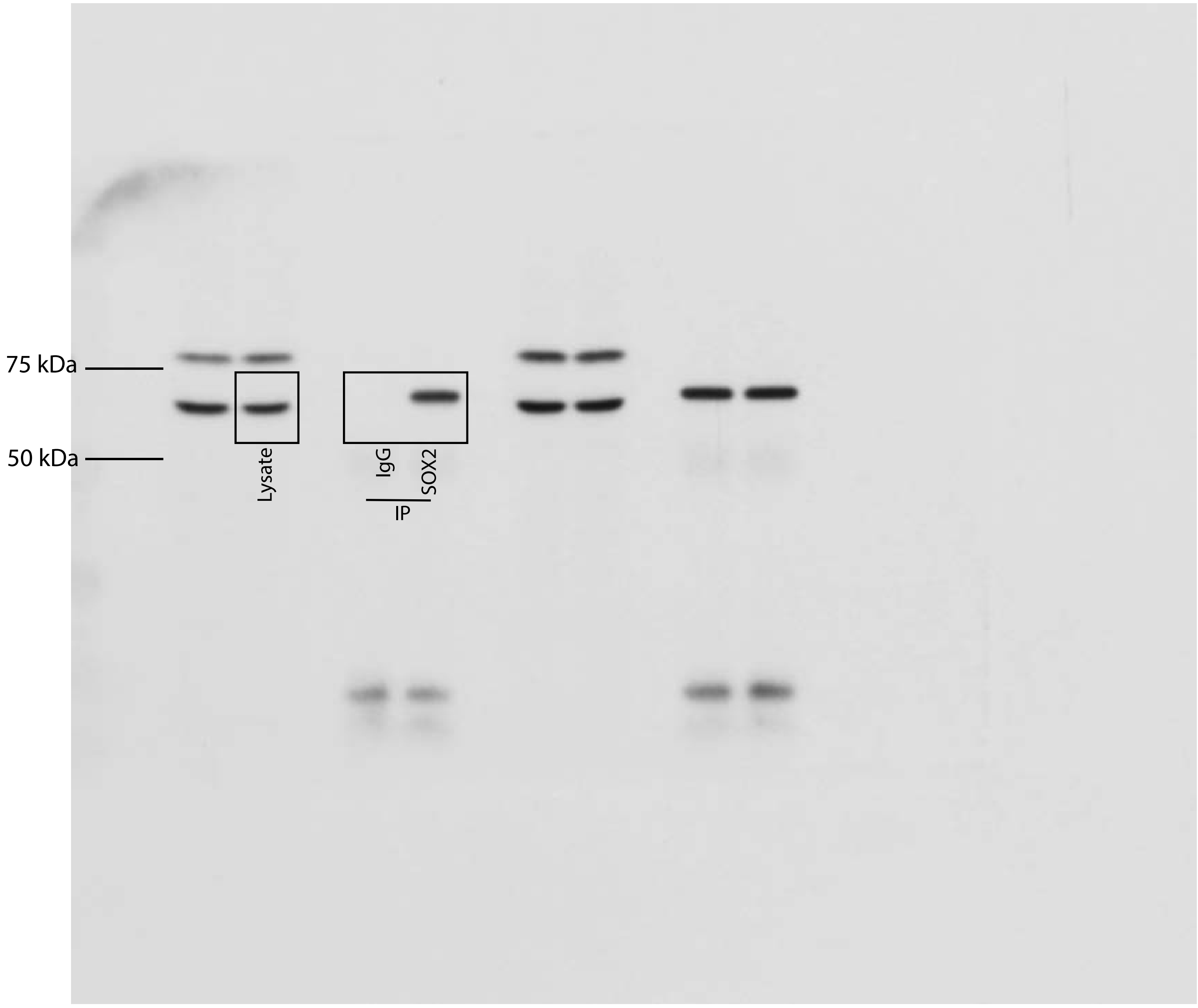

B51

TRIM26 ANTIBODY

FIG 1G

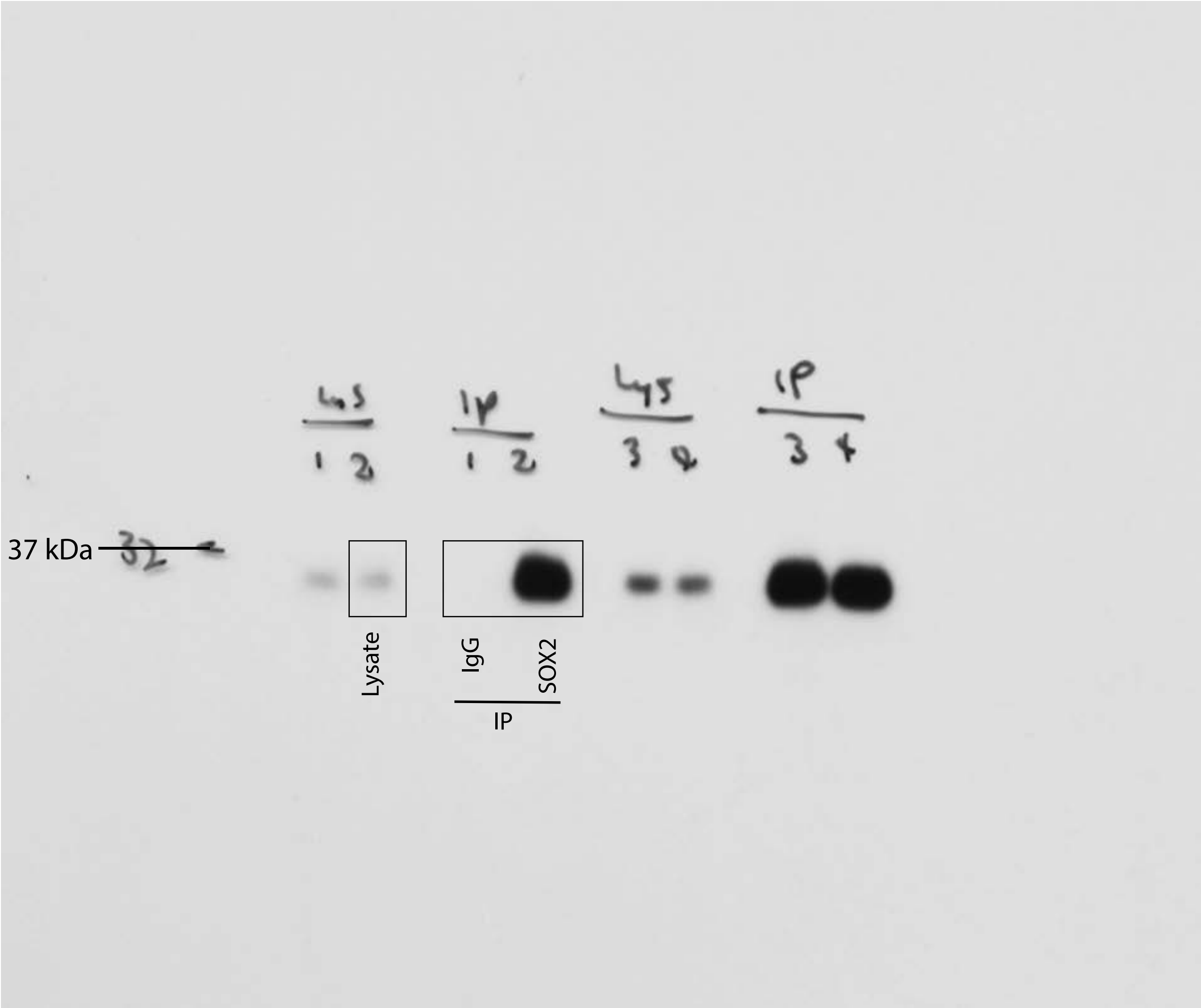

B67

SOX2 ANTIBODY

FIG 1G

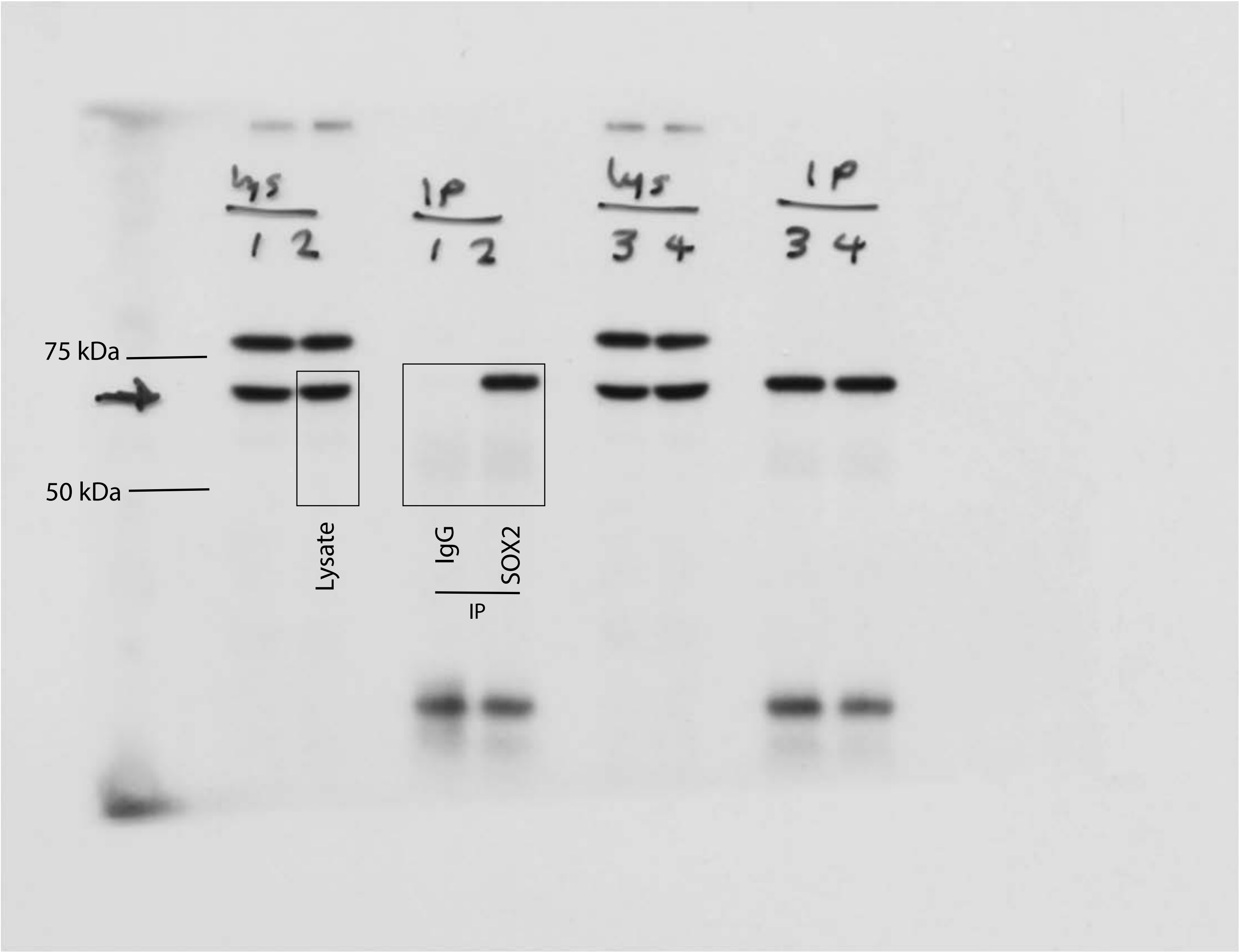

B67

TRIM26 ANTIBODY

FIG 2A

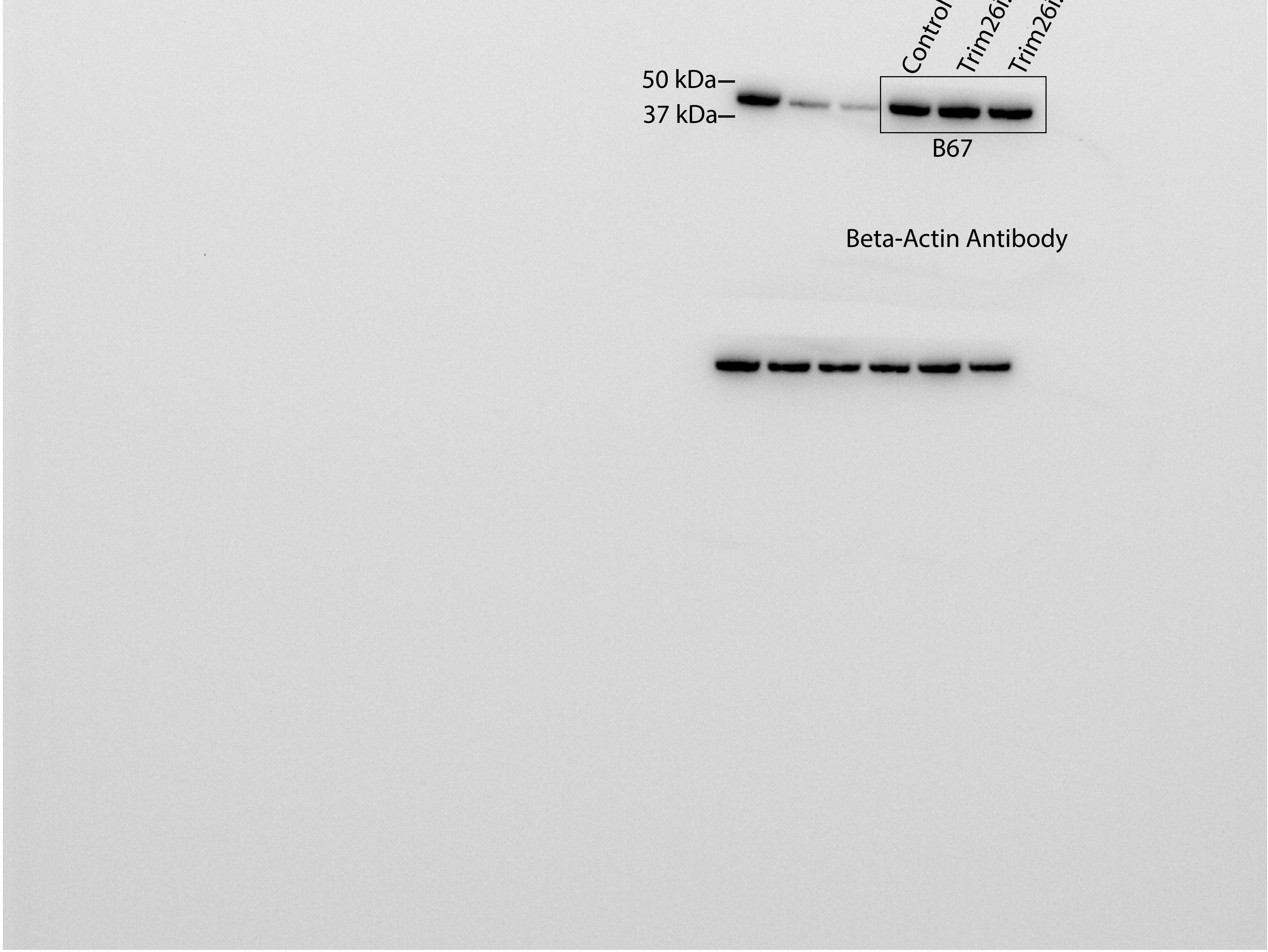

Control
Trim26i.1
Trim26i.2
50 kDa
37 kDa
B67
Beta-Actin Antibody

FIG 2A

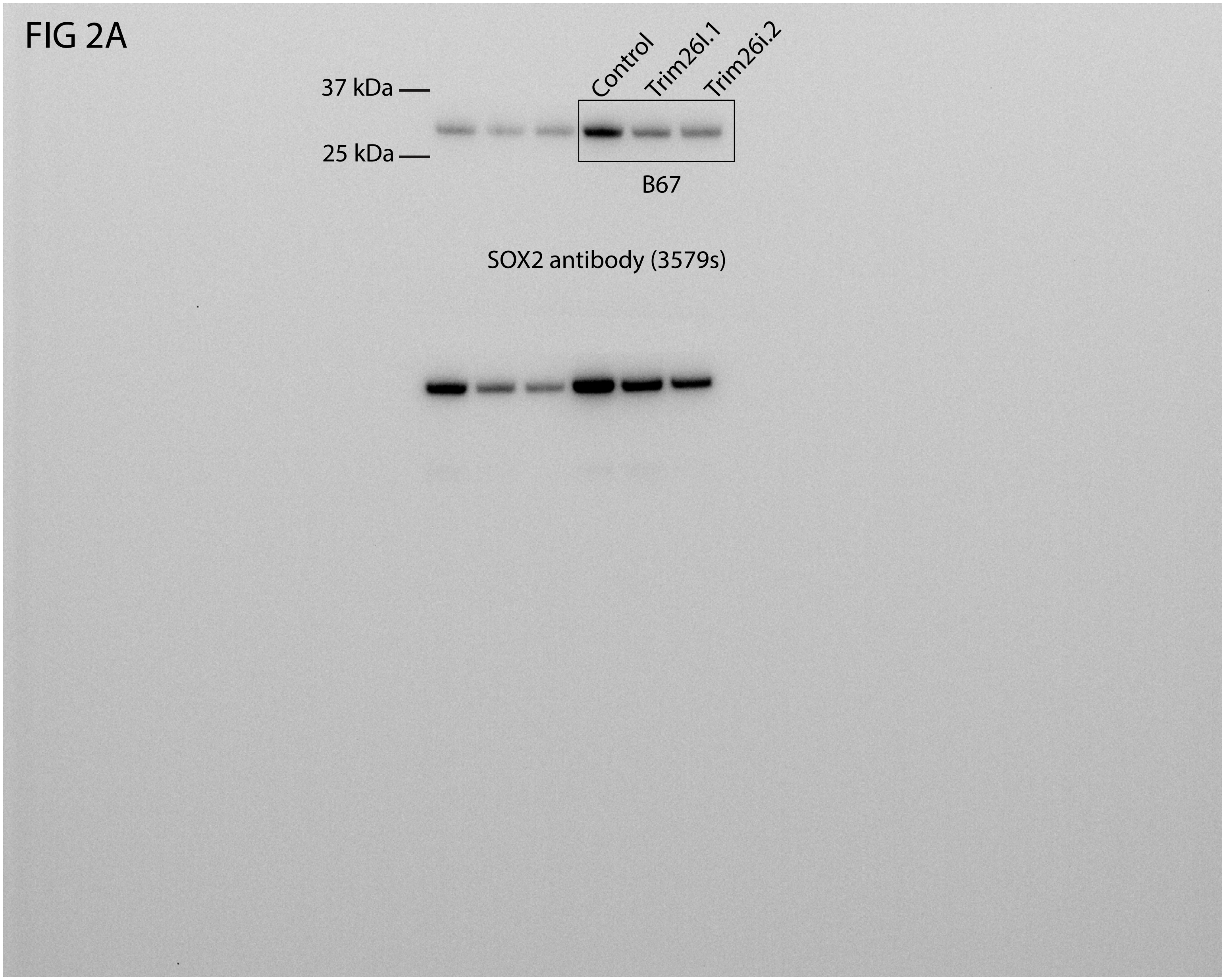

37 kDa
25 kDa
Control
Trim26I.1
Trim26i.2
B67
SOX2 antibody (3579s)

FIG 2A

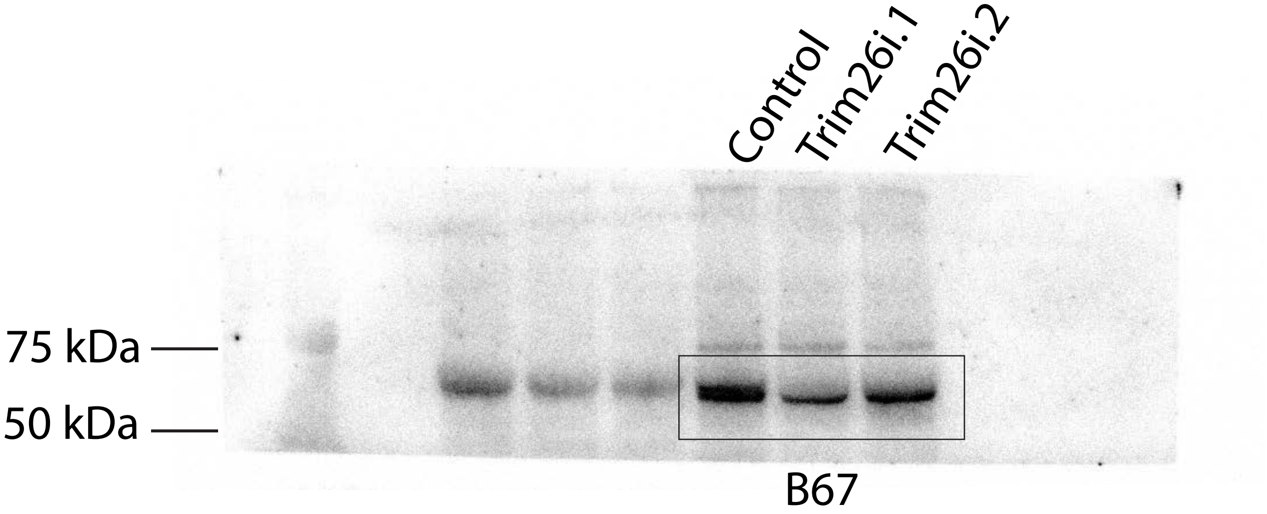

TRIM26 Aantibody (sc-393832)

FIG 2A

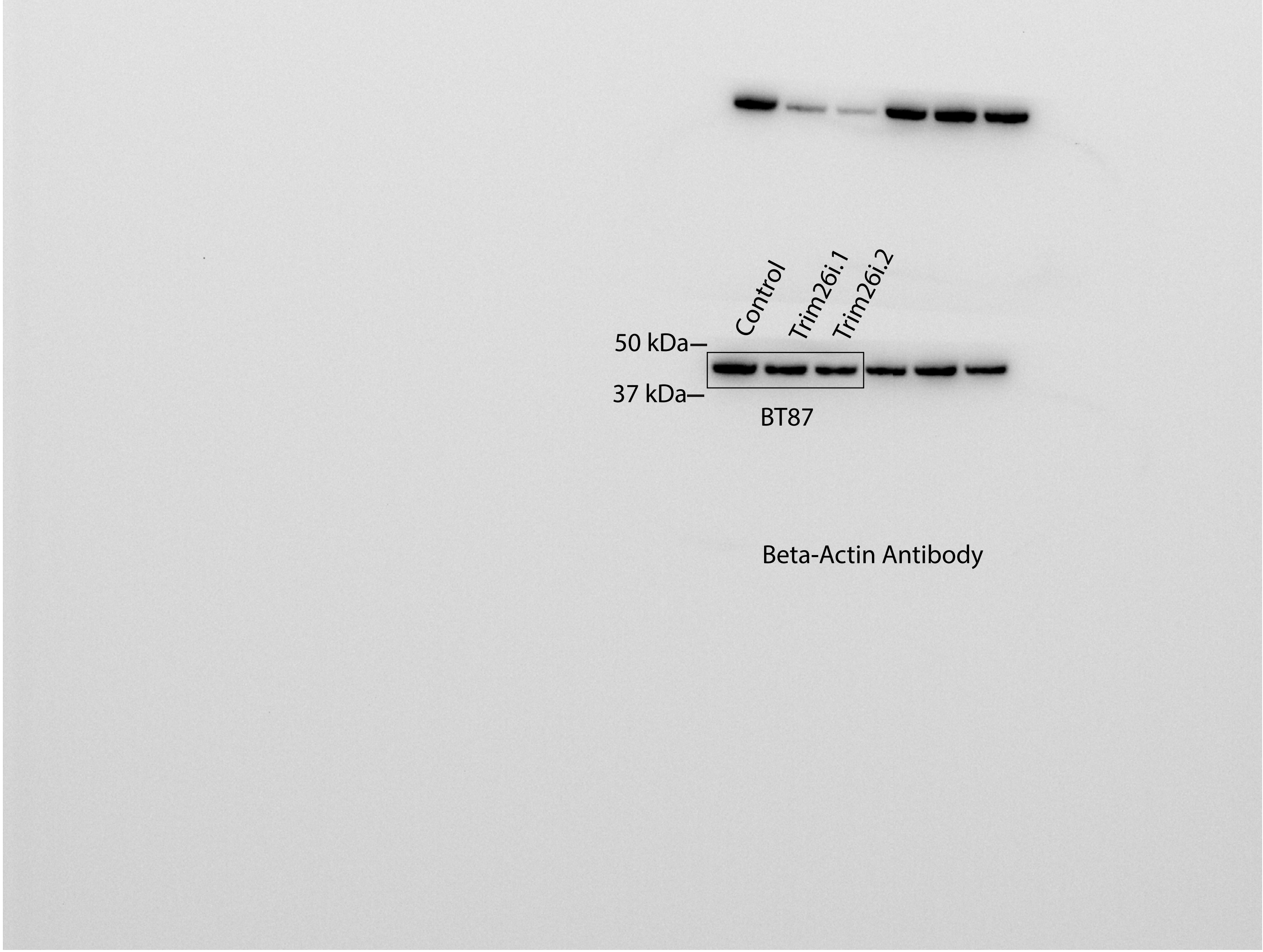

Control
Trim26i.1
Trim26i.2
50 kDa
37 kDa
BT87
Beta-Actin Antibody

FIG 2A

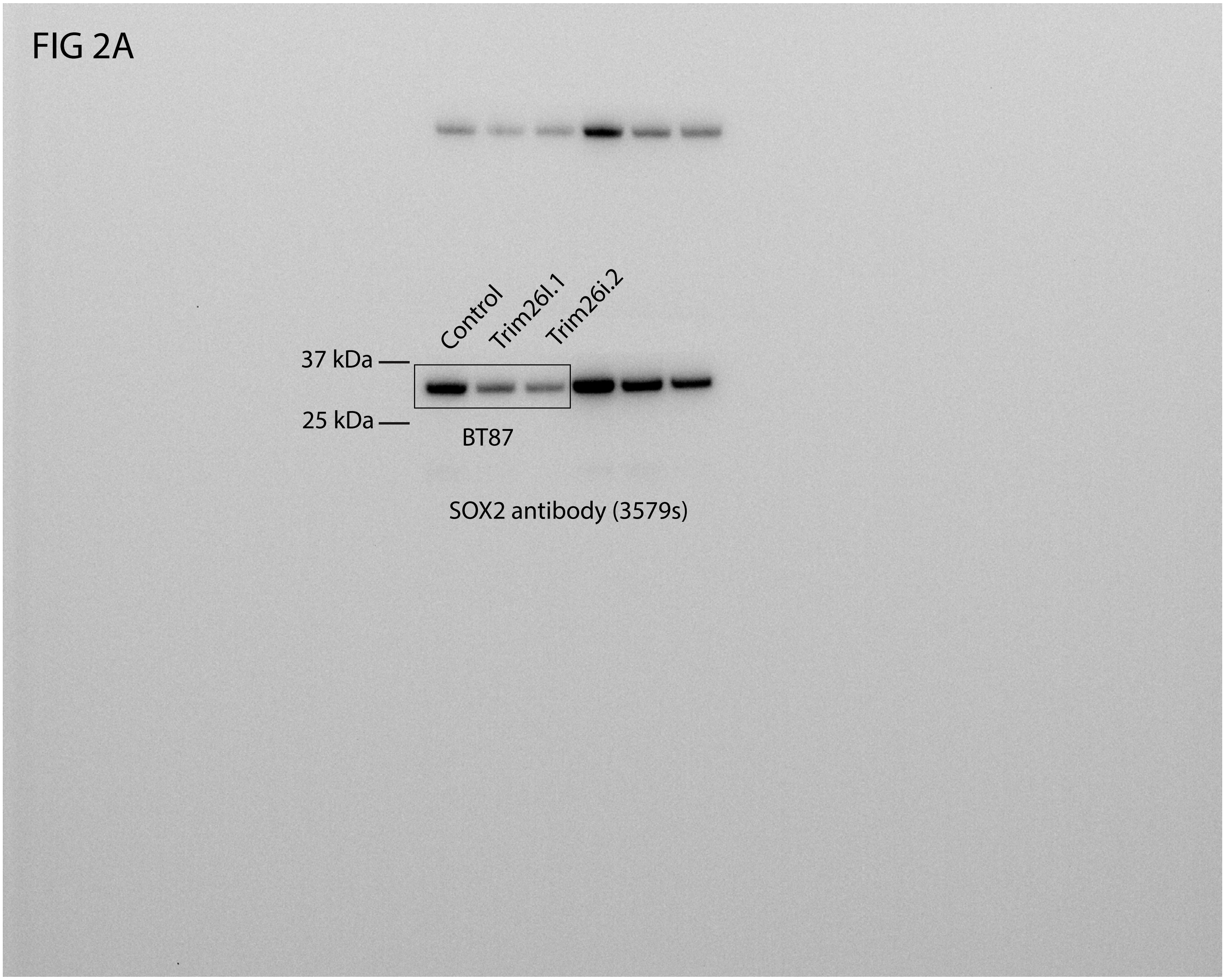
Control
Trim26I.1
Trim26i.2
37 kDa
25 kDa
BT87
SOX2 antibody (3579s)

FIG 2A

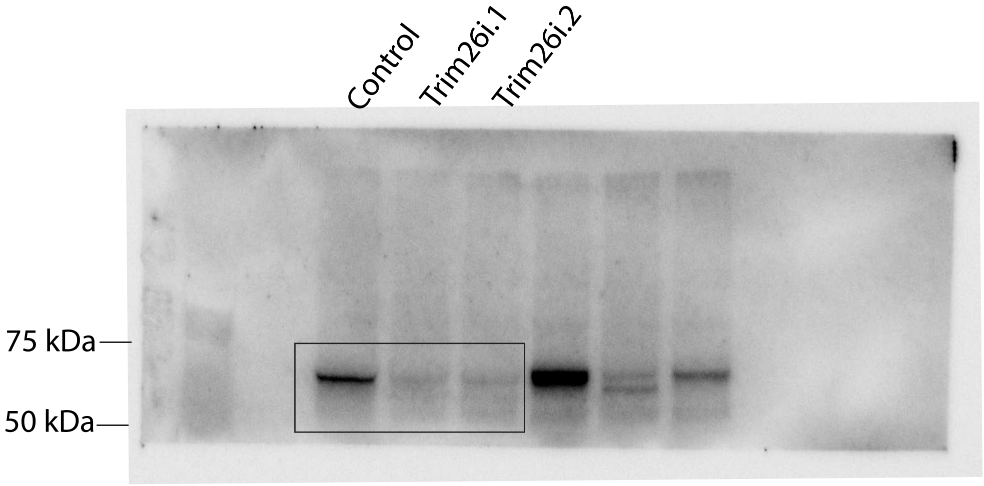

BT87

TRIM26 antibody (sc-393832)

FIG 2A

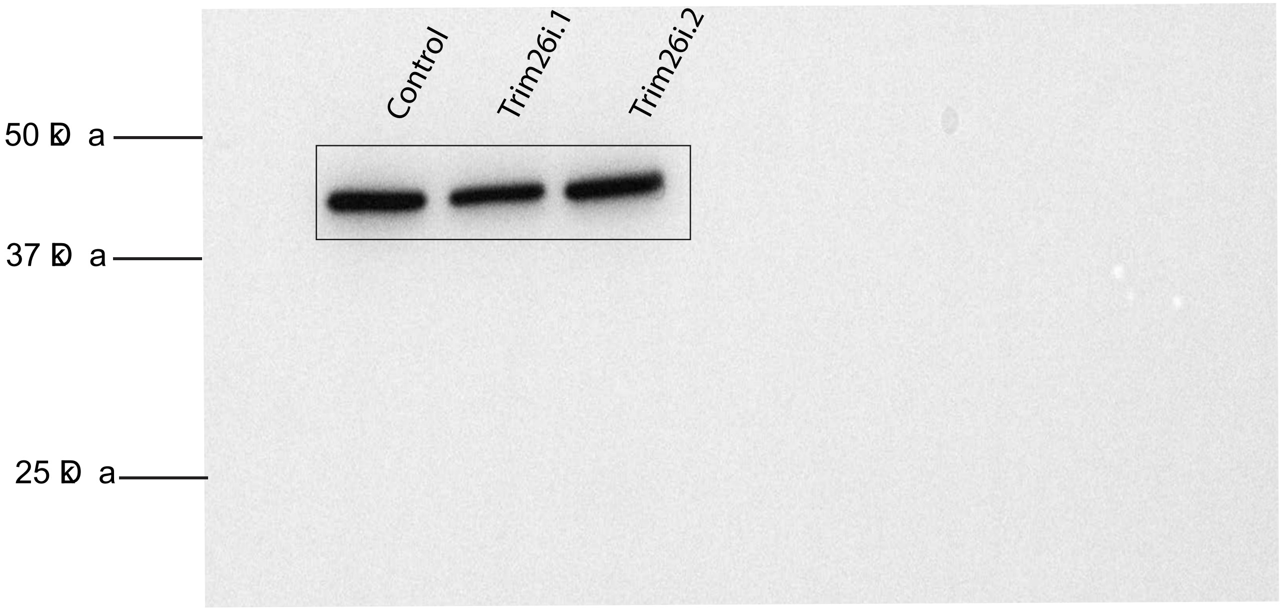

MGG8

ACTIN ANTIBODY

FIG 2A

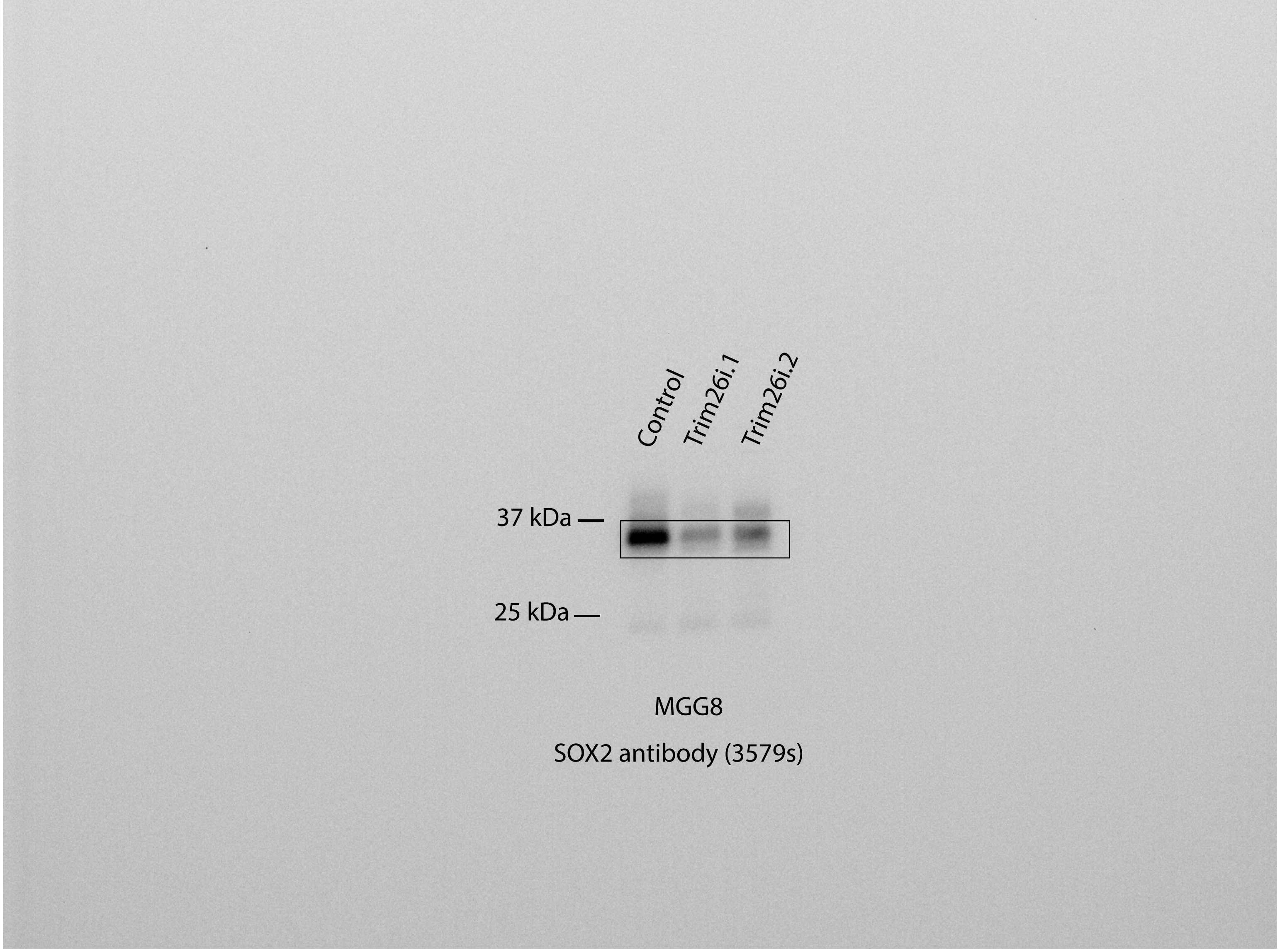

Control
Trim26i.1
Trim26i.2
37 kDa
25 kDa
MGG8
SOX2 antibody (3579s)

FIG 2A

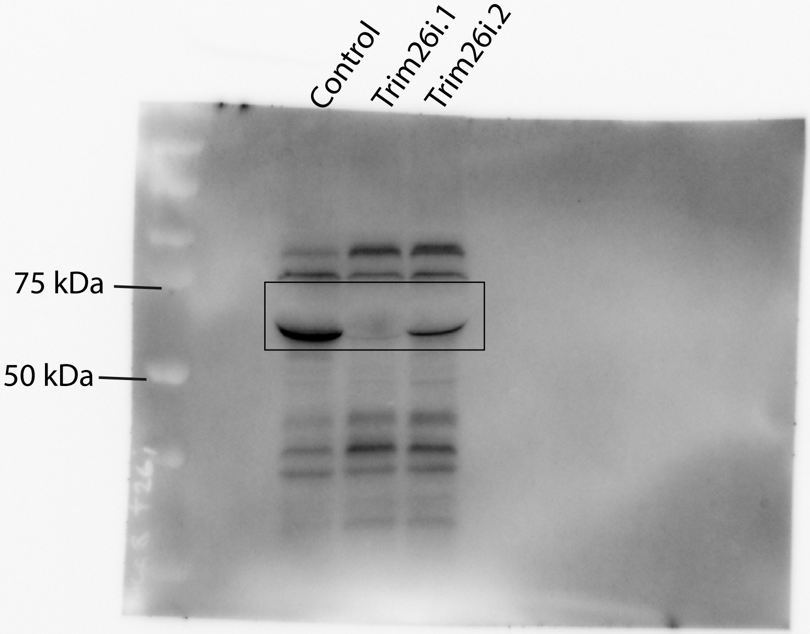

MGG8

TRIM26 antibody (sc393832)

FIG 2B

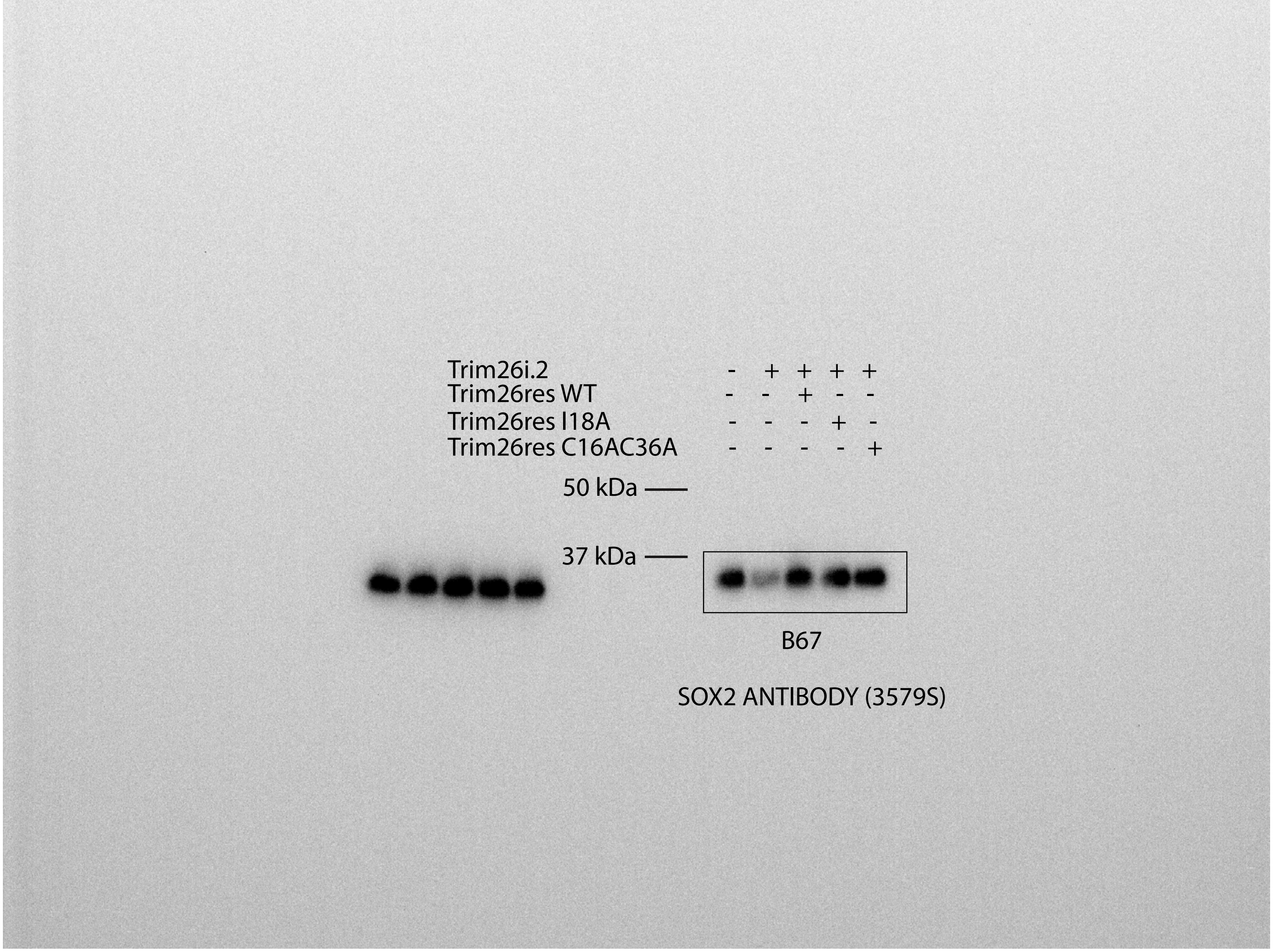

Trim26i.2 - + + + +
Trim26res WT - - + - -
Trim26res I18A - - - + -
Trim26res C16AC36A - - - - +
50 kDa
37 kDa
B67
SOX2 ANTIBODY (3579S)

FIG 2B

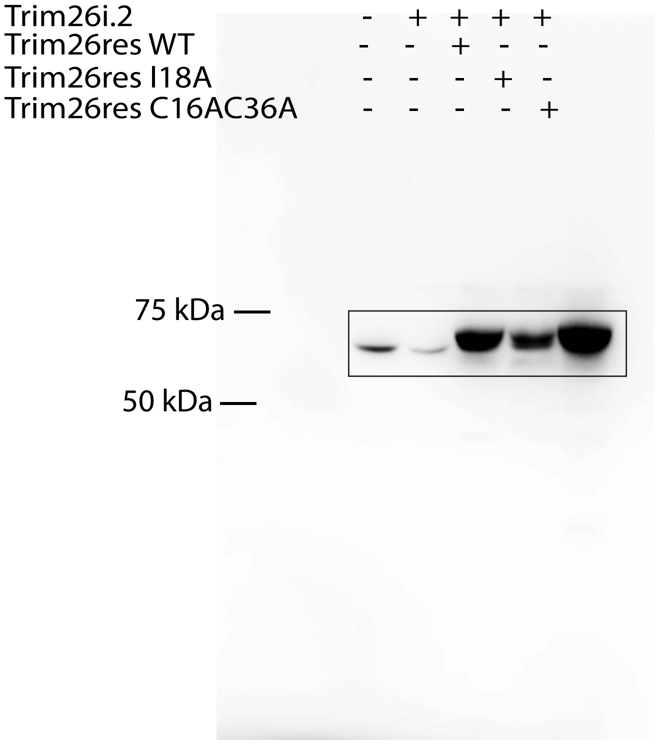

B67

TRIM26 ANTIBODY (SC393832)

FIG 2B

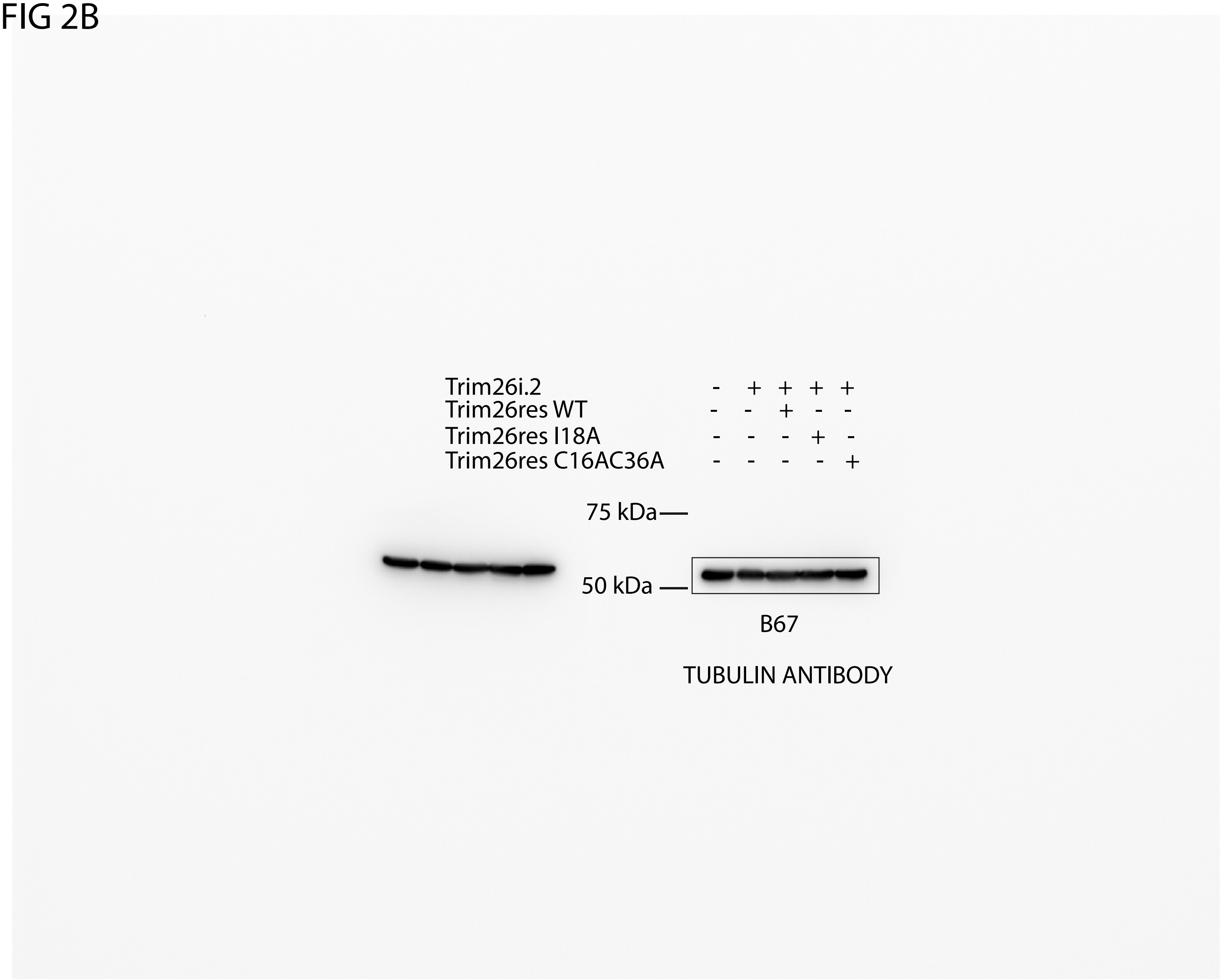

Trim26i.2 - + + + +
Trim26res WT - - + - -
Trim26res I18A - - - + -
Trim26res C16AC36A - - - - +
75 kDa
50 kDa
B67
TUBULIN ANTIBODY

FIG 2C

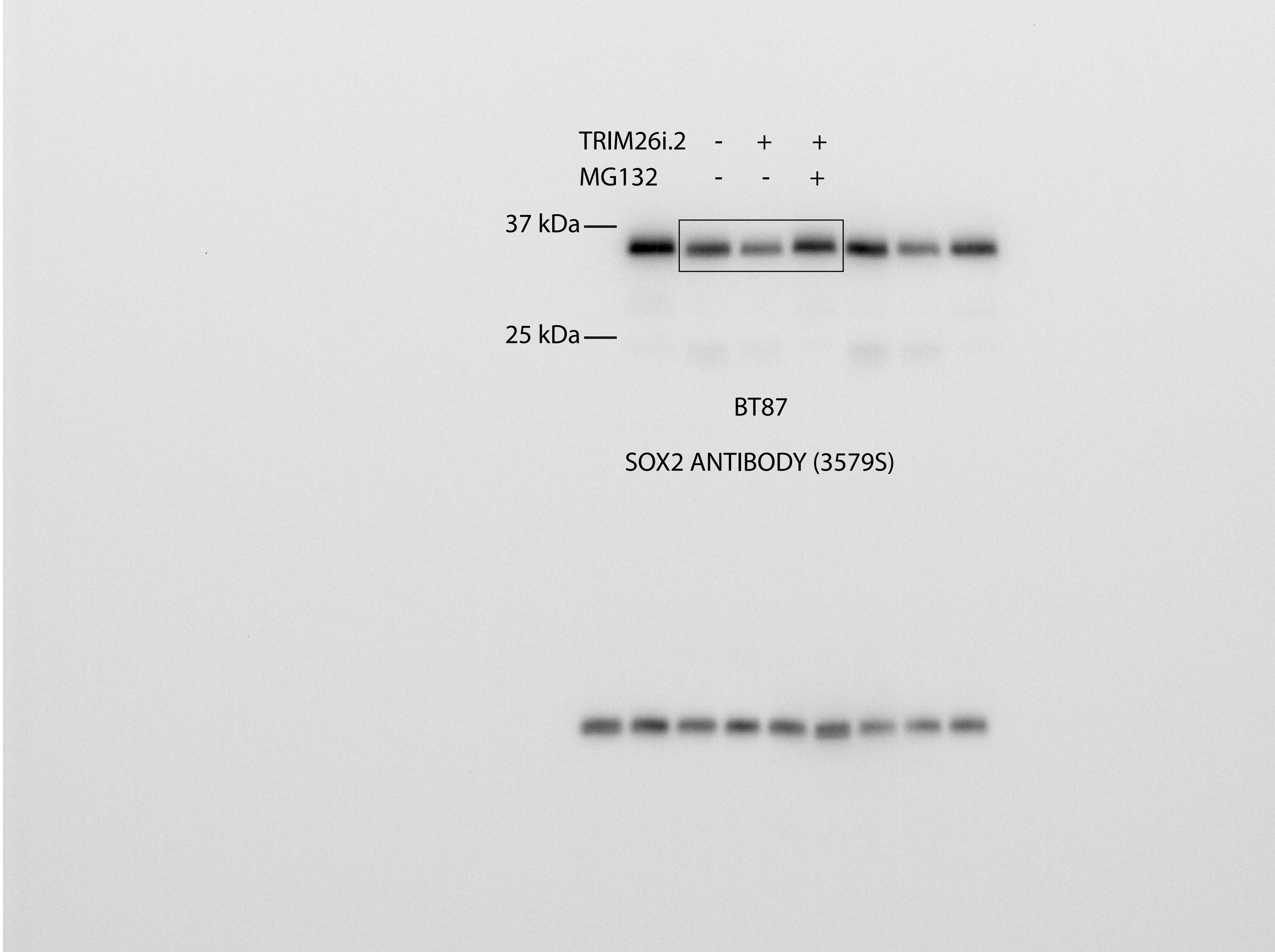

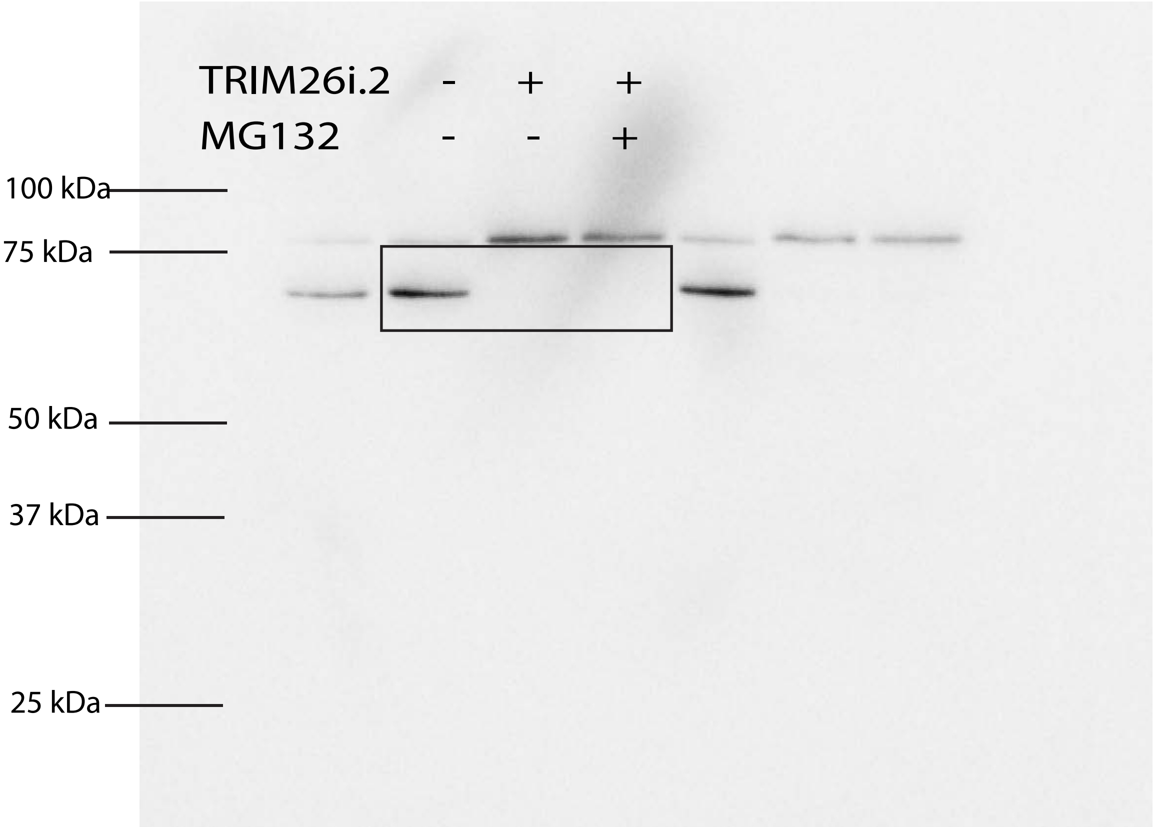

BT87

TRIM26 ANTIBODY

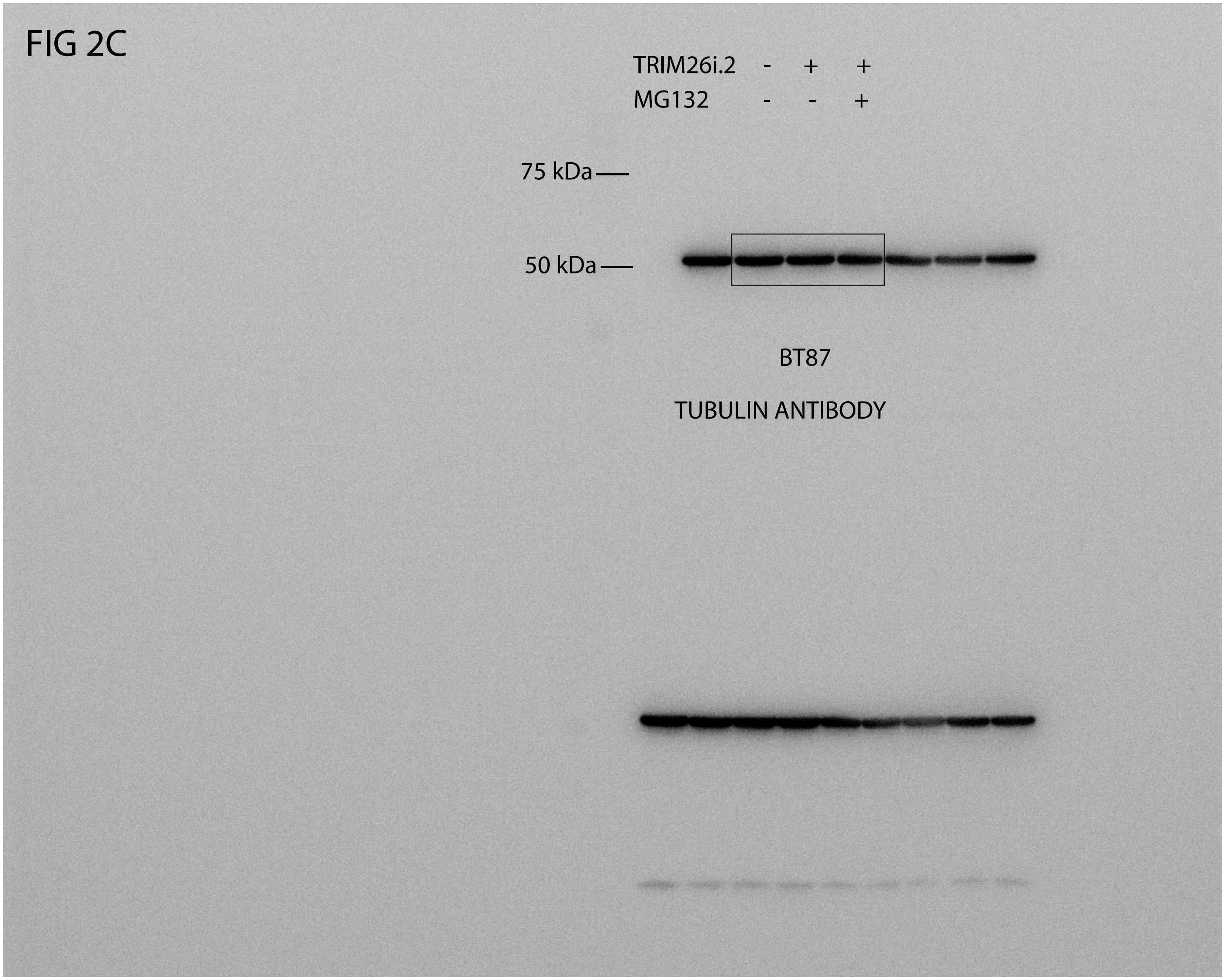
FIG 2C
TRIM26i.2 - + +
MG132 - - +
75 kDa
50 kDa
BT87
TUBULIN ANTIBODY

FIG 2D

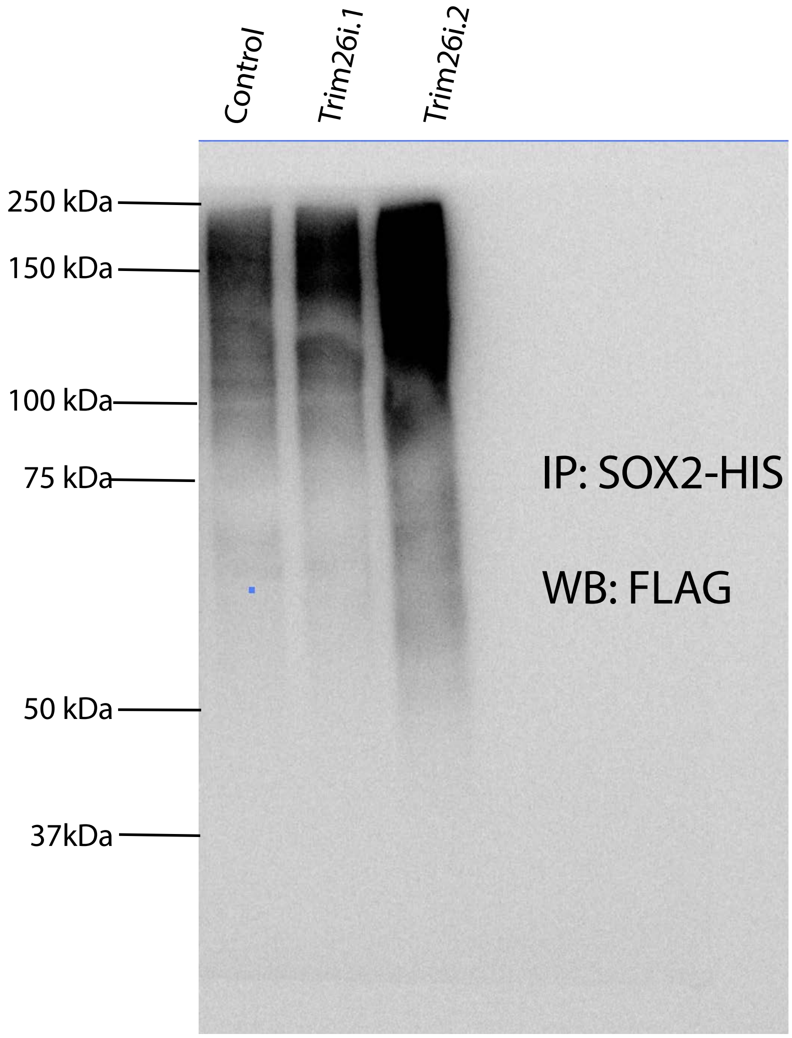

B67

FLAG ANTIBODY

FIG 2D

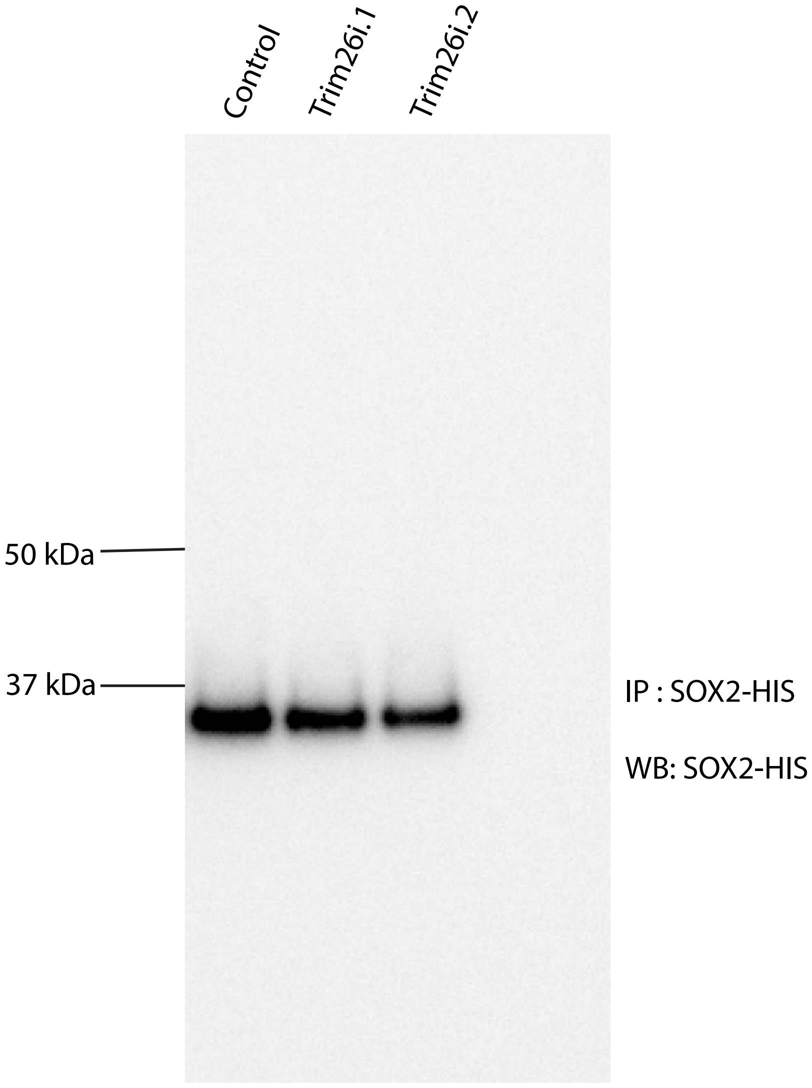

B67

HIS-TAG ANTIBODY

FIG. 2D

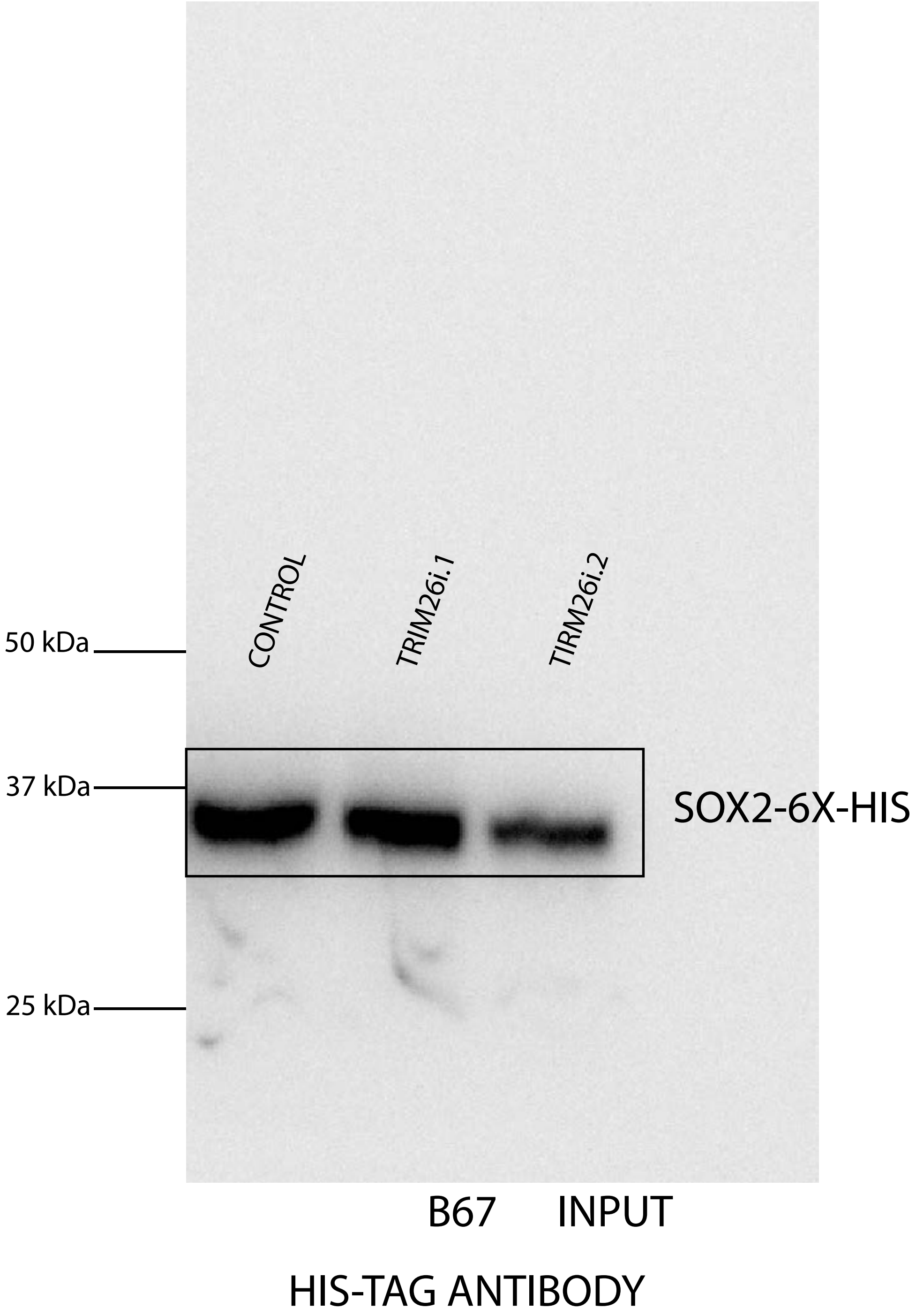

50 kDa
37 kDa
25 kDa
CONTROL
TRIM26i.1
TIRM26i.2
SOX2-6X-HIS
B67
INPUT
HIS-TAG ANTIBODY

FIG 2D

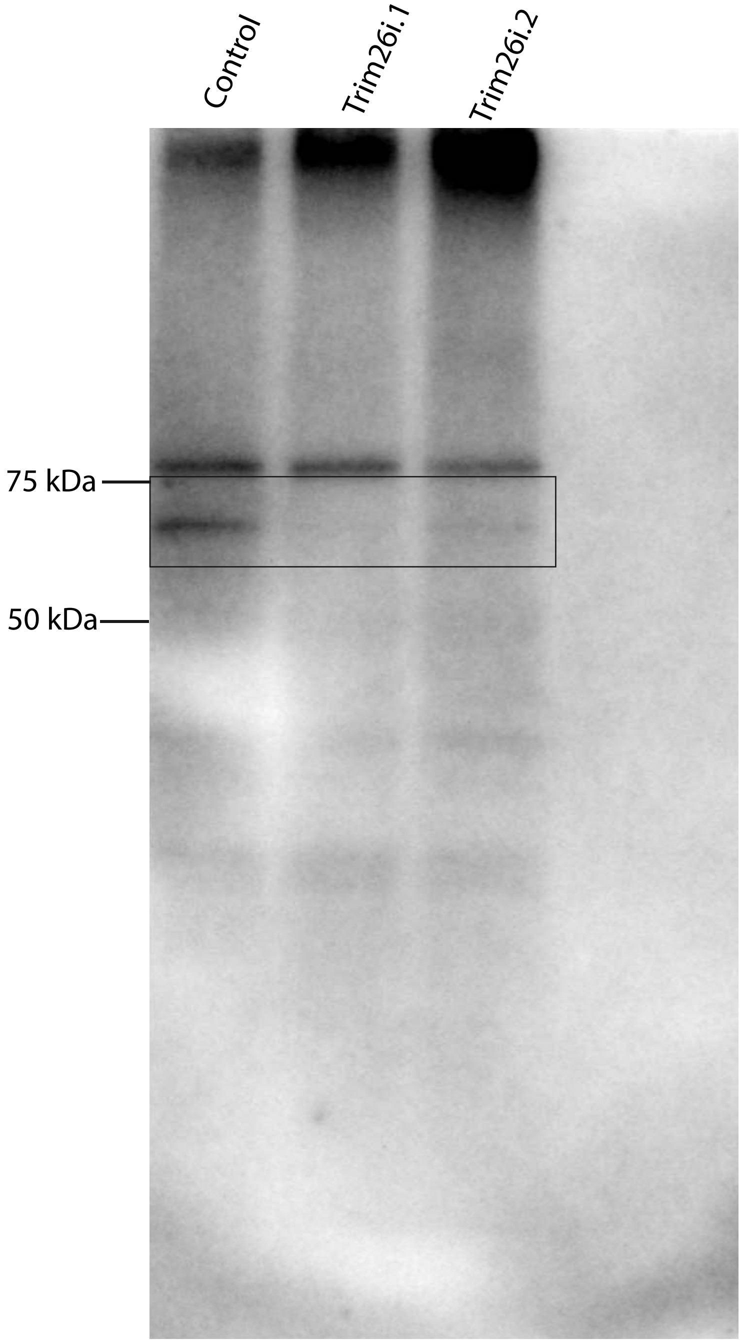

B67 INPUT

TRIM26 ANTIBODY (SC393832)

FIG. 2D

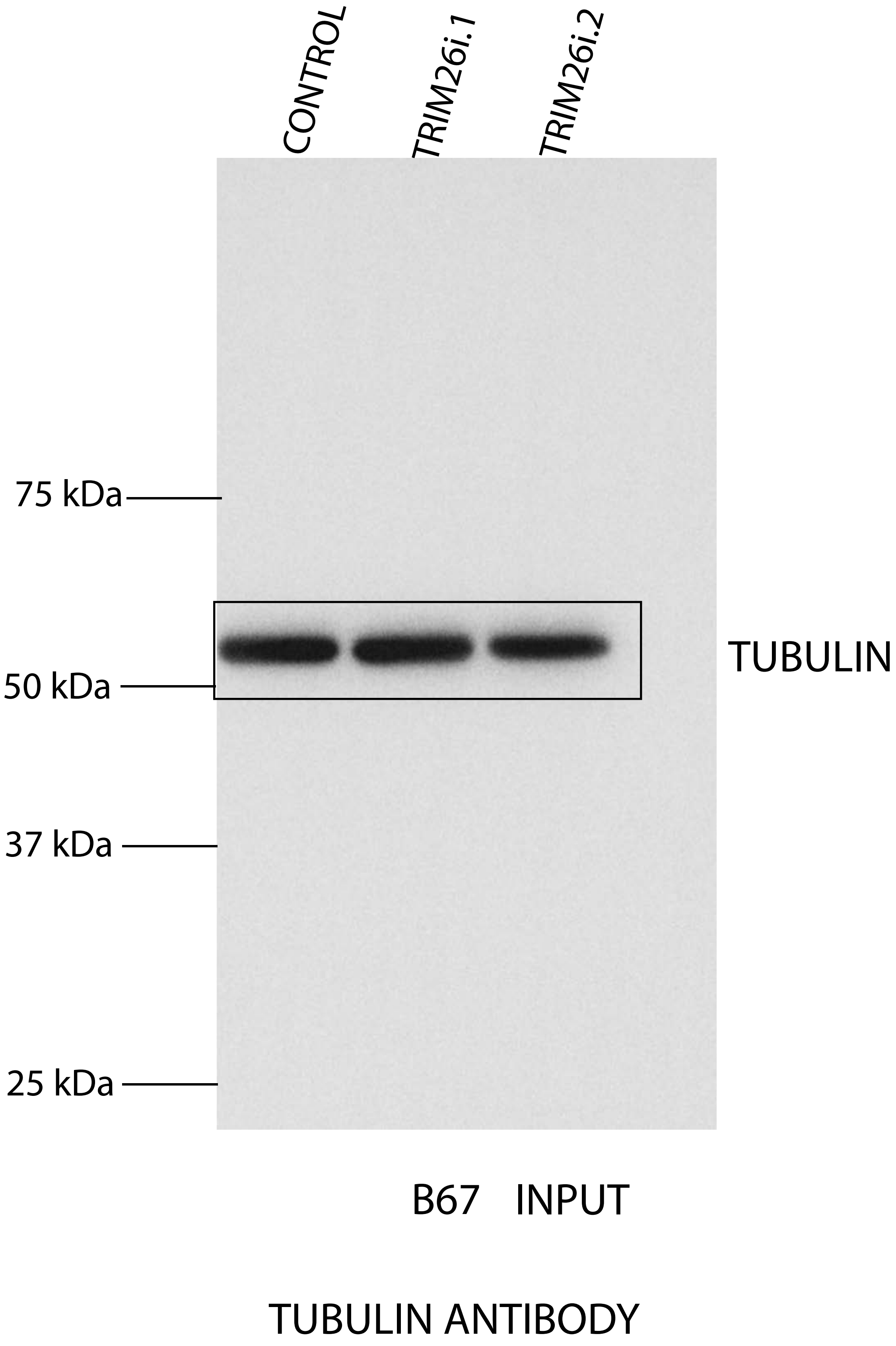

CONTROL
TRIM26i.1
TRIM26i.2
75 kDa
50 kDa
37 kDa
25 kDa
TUBULIN
B67 INPUT
TUBULIN ANTIBODY

FIG 3D

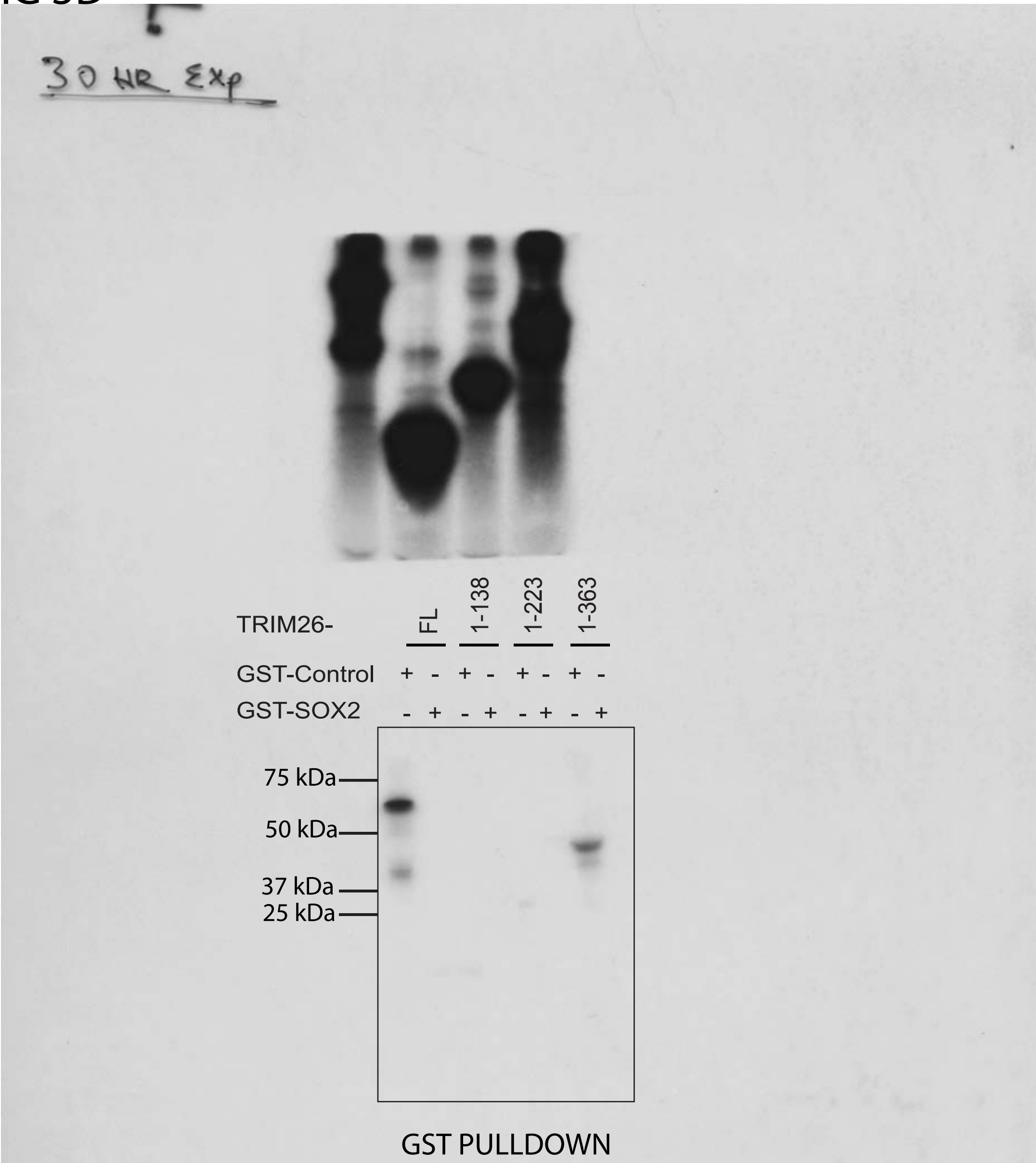

^{35}S AUTORADIOGRAPH

FIG 3D

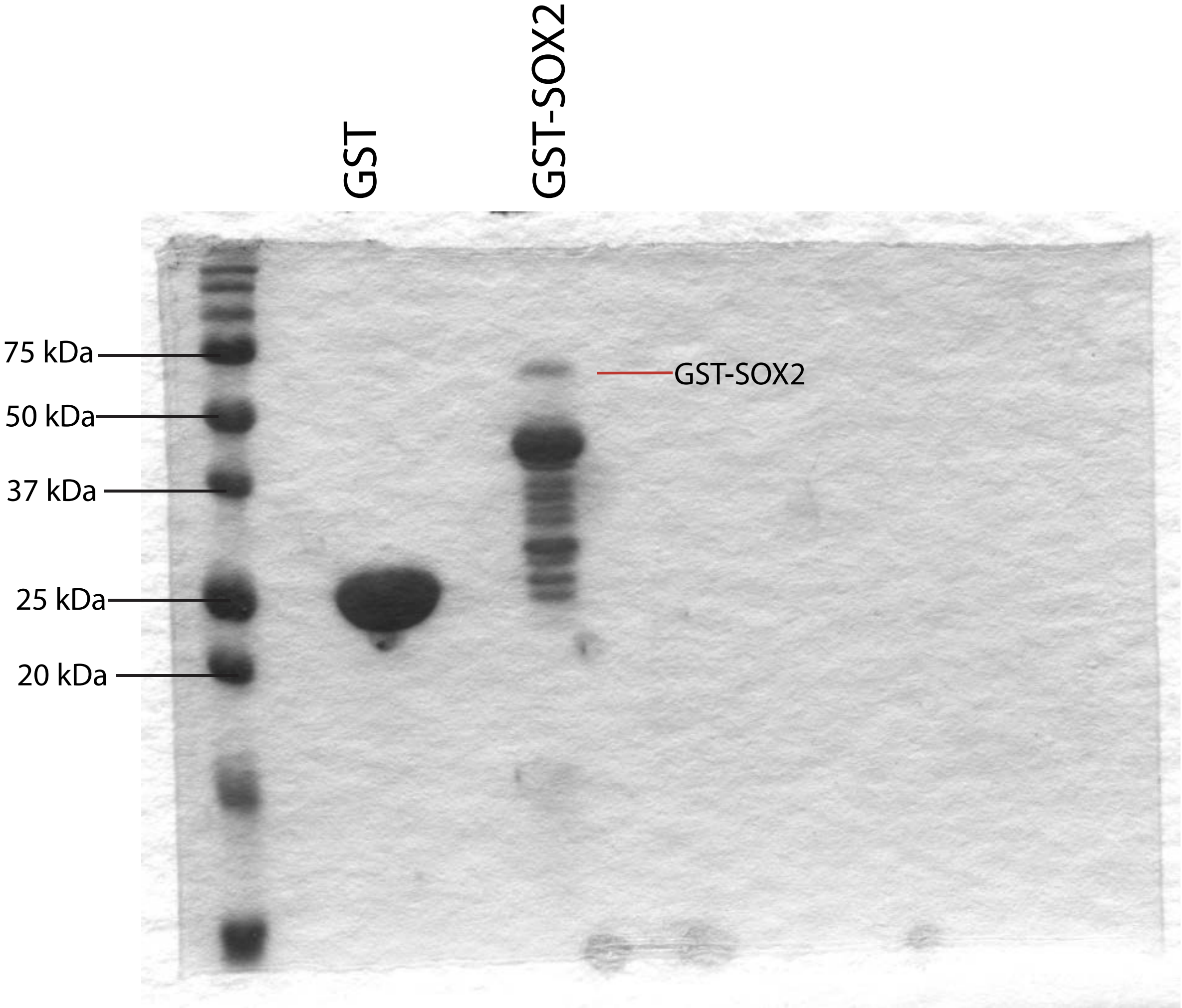

COOMASSIE BLUE GEL

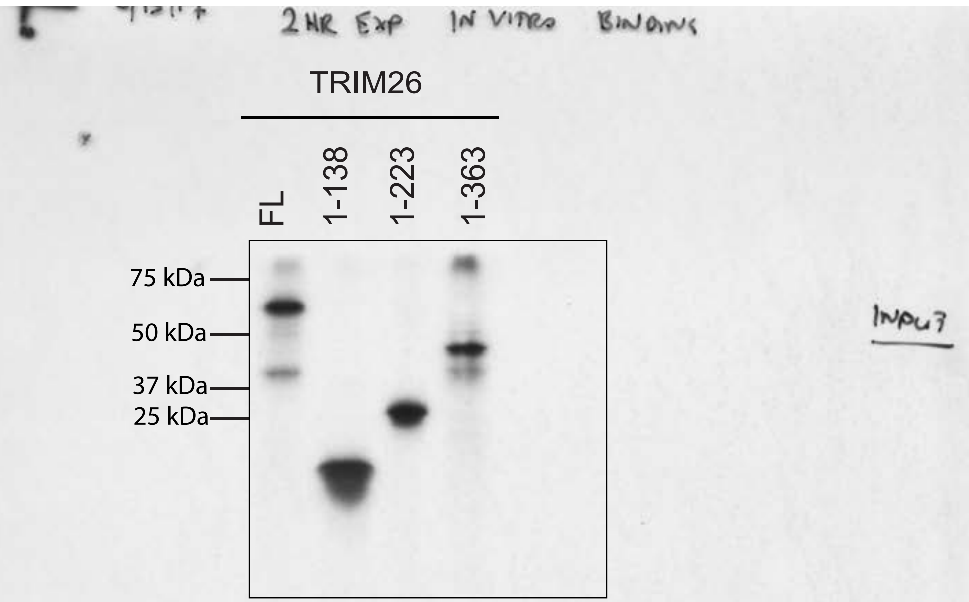

INPUT

^{35}S AUTORADIOGRAPH

FIG 3E

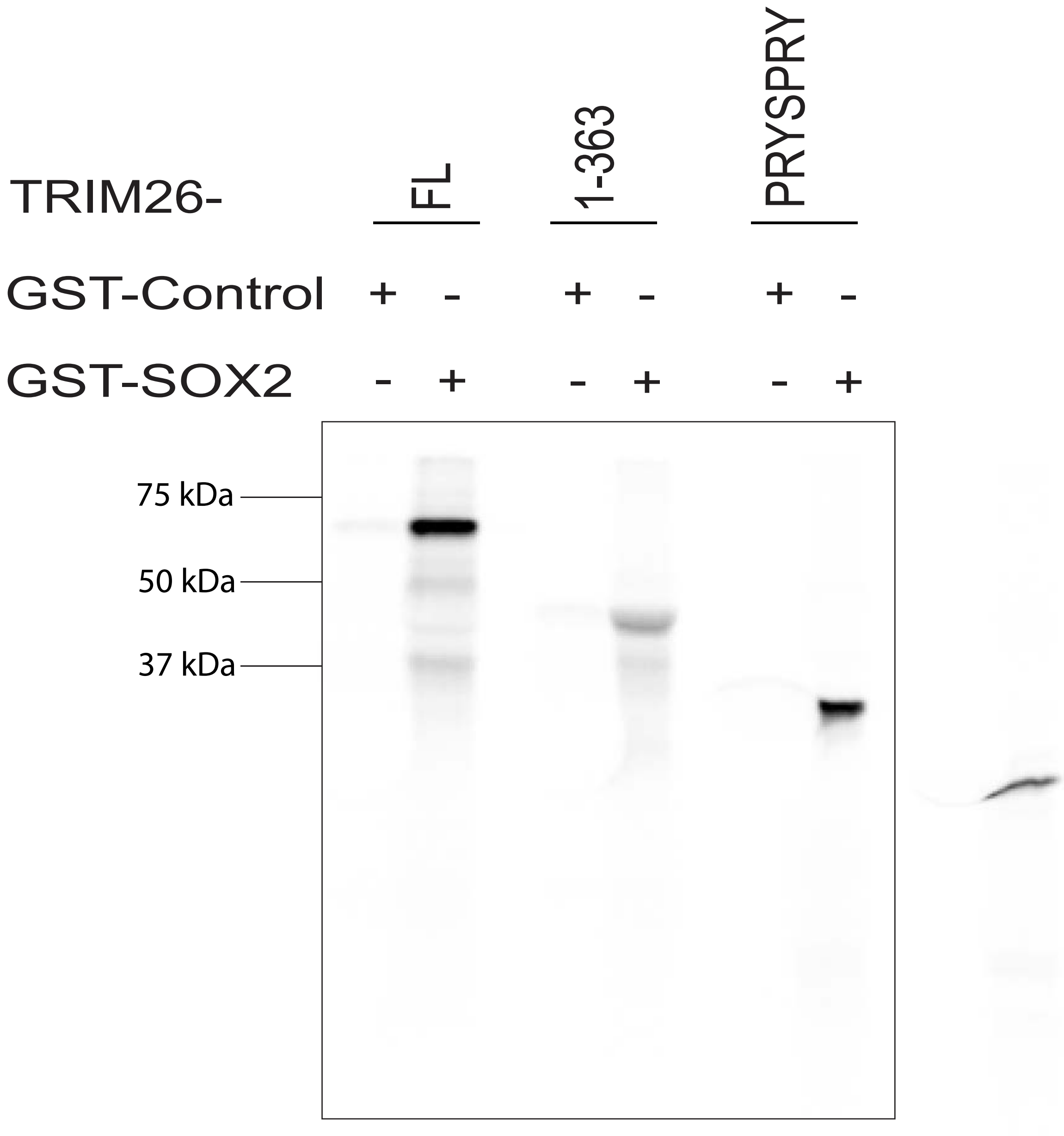

GST PULLDOWN

^{35}S AUTORADIOGRAPH

FIG 3E

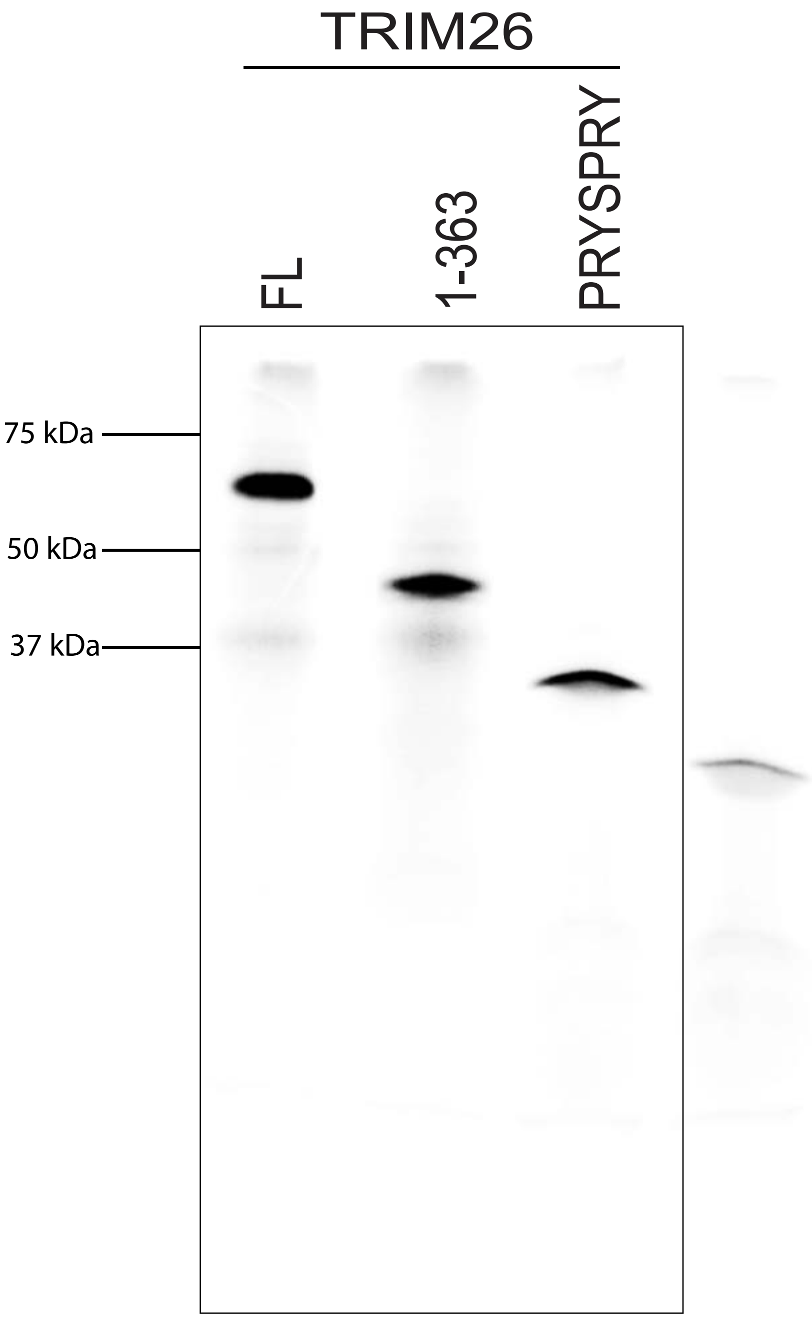

INPUT

^{35}S AUTORADIOGRAPH

FIG 3F

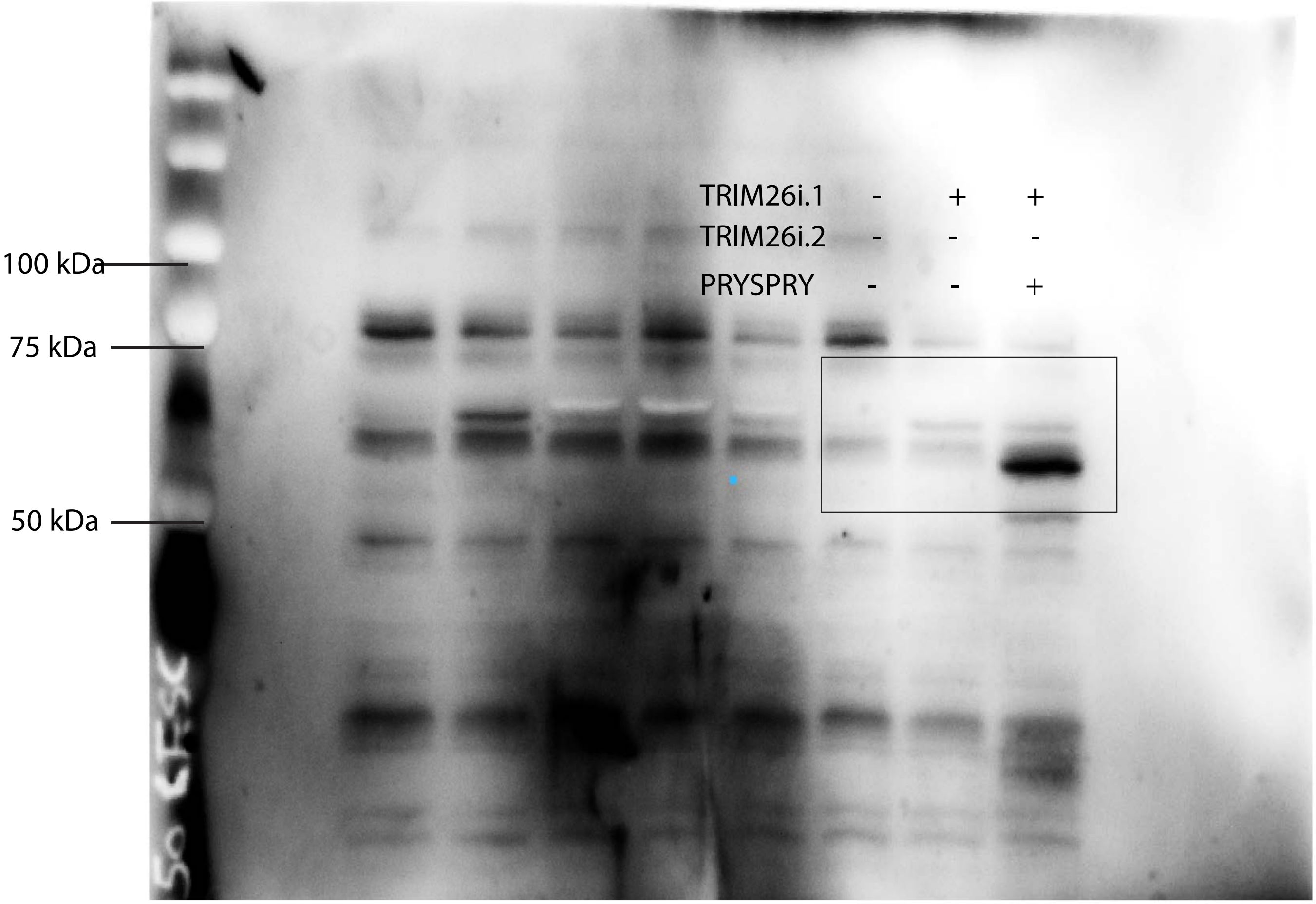

B67

GFP ANTIBODY

FIG 3F

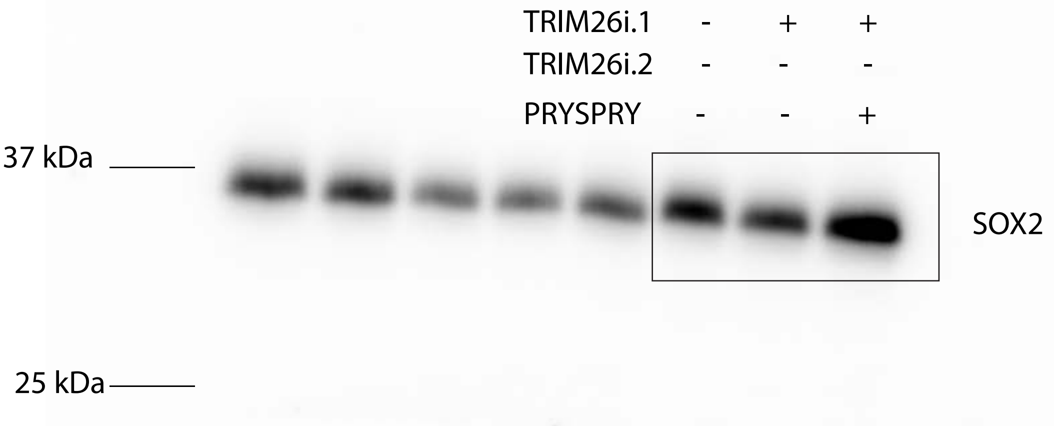

B67

SOX2 ANTIBODY

FIG 3F

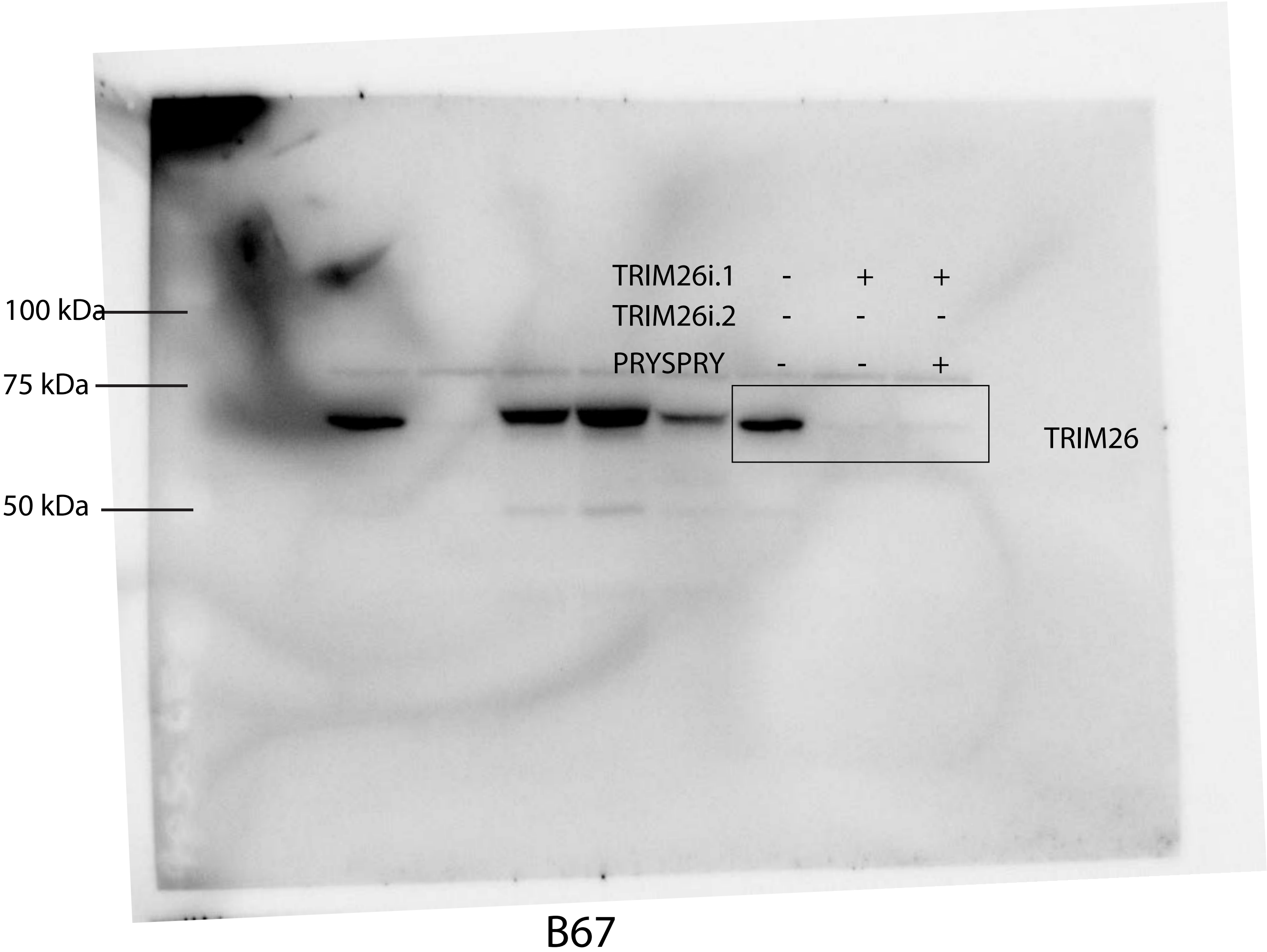

B67

TRIM26 ANTIBODY (SC393832)

FIG 3F

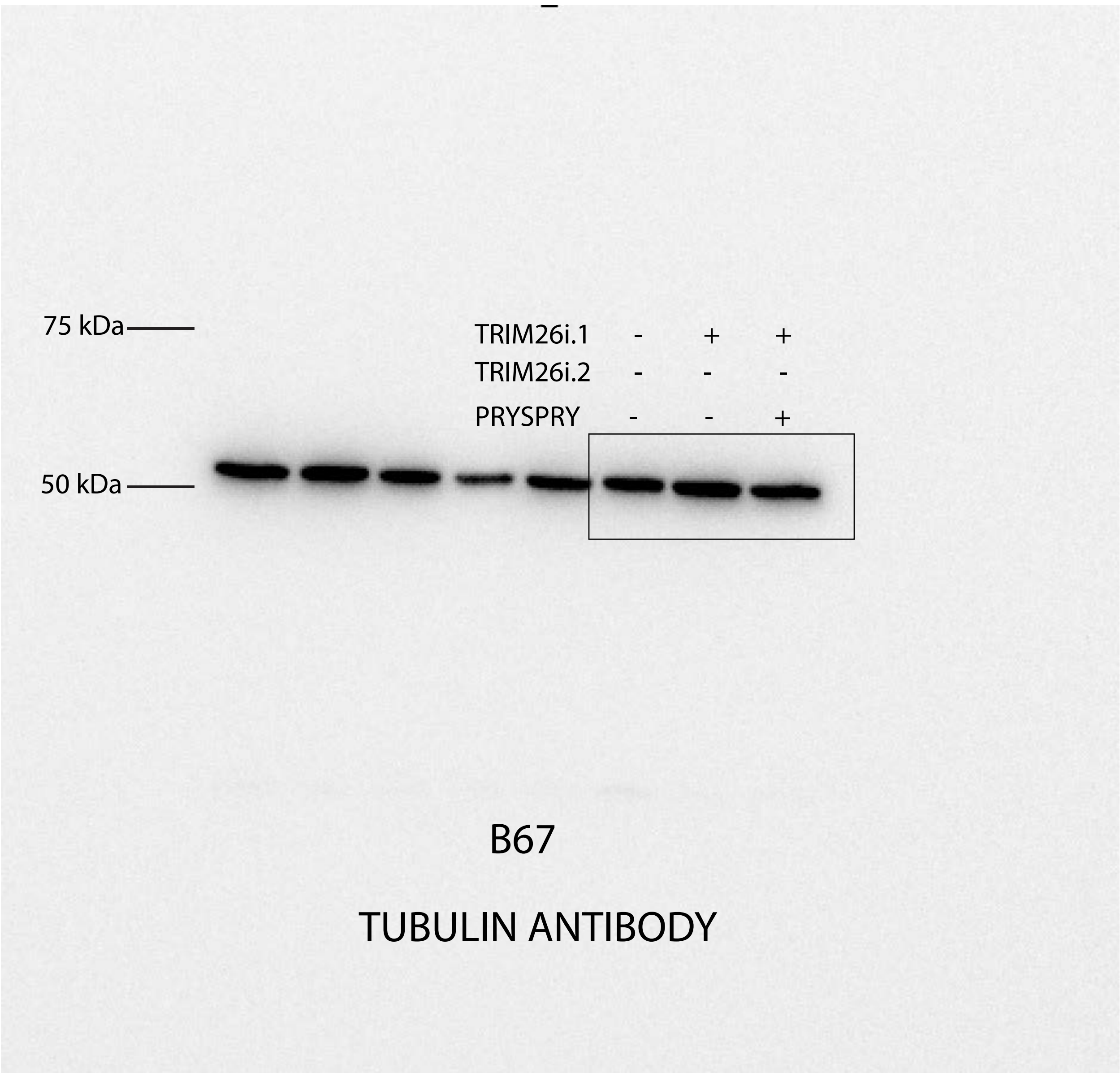
75 kDa
50 kDa
TRIM26i.1 - + +
TRIM26i.2 - - -
PRYSPRY - - +
B67
TUBULIN ANTIBODY

FIG 3F

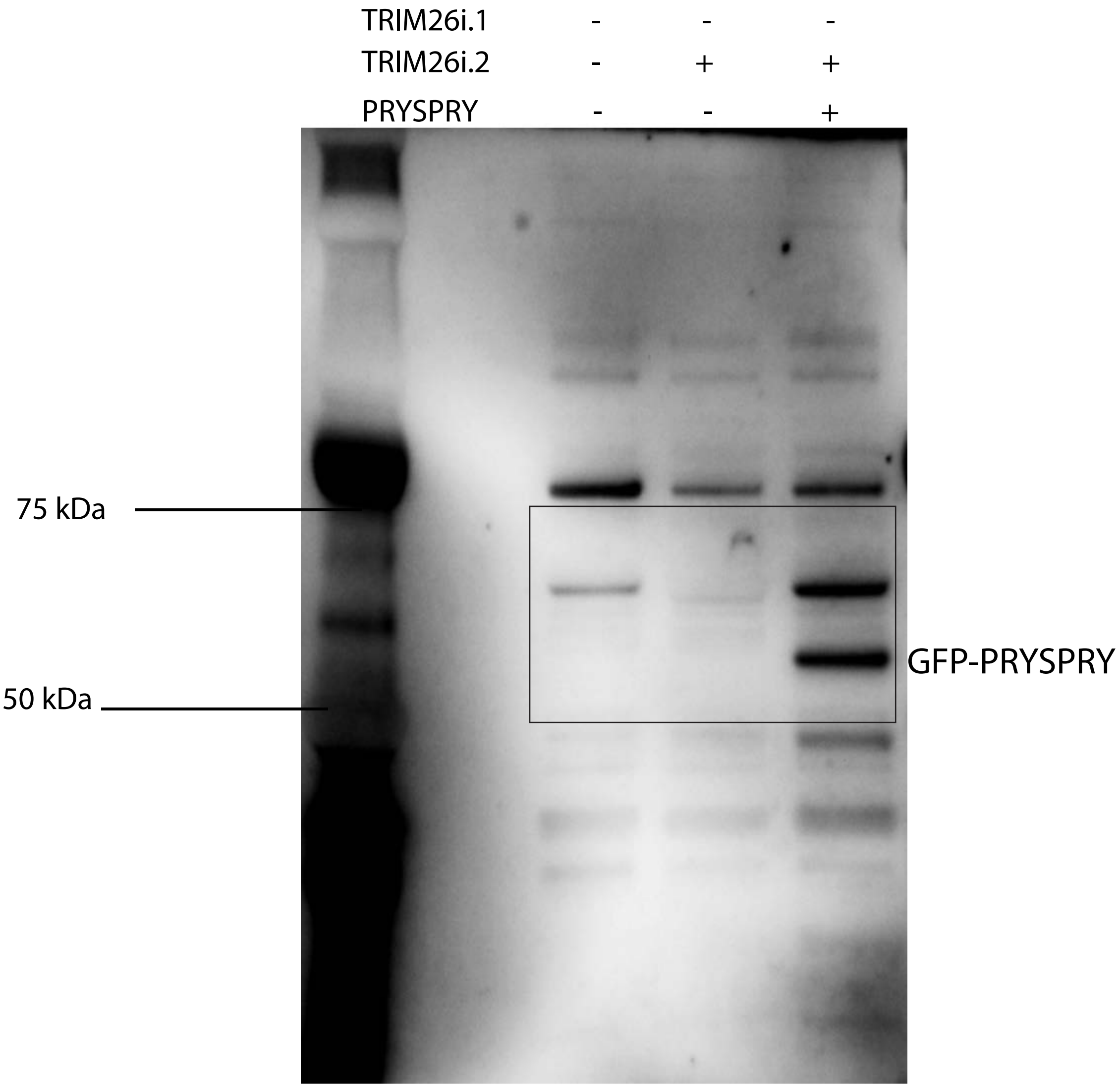

B67

GFP ANTIBODY

FIG 3F

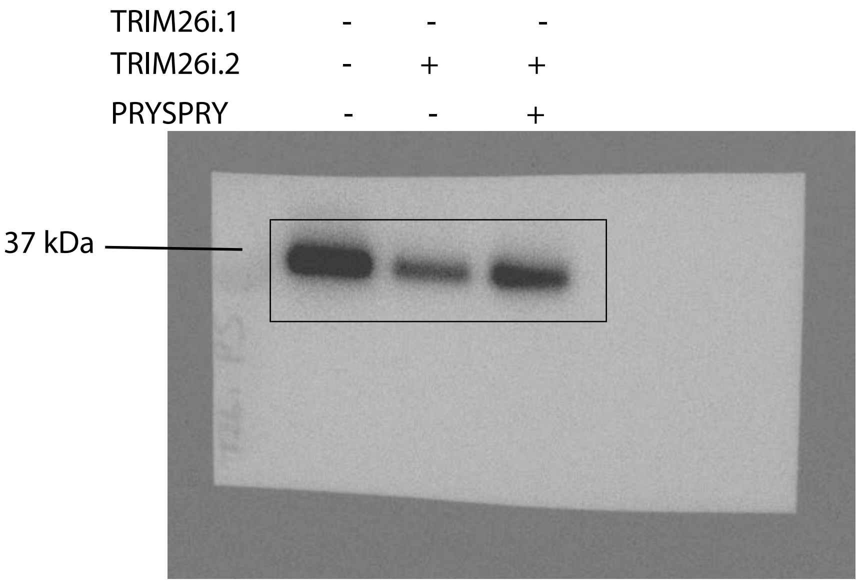

B67

SOX2 ANTIBODY (3579S)

FIG 3F

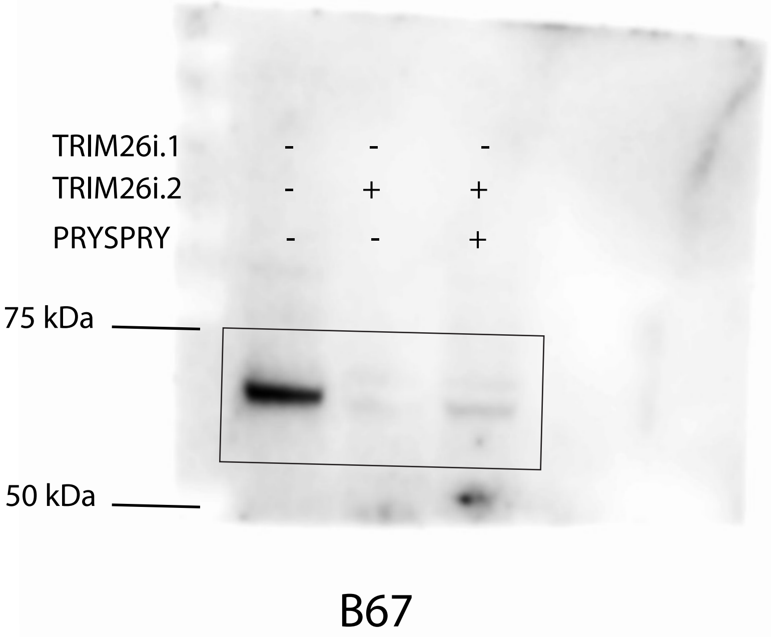

TRIM26 ANTIBODY (SC393832)

FIG 3F

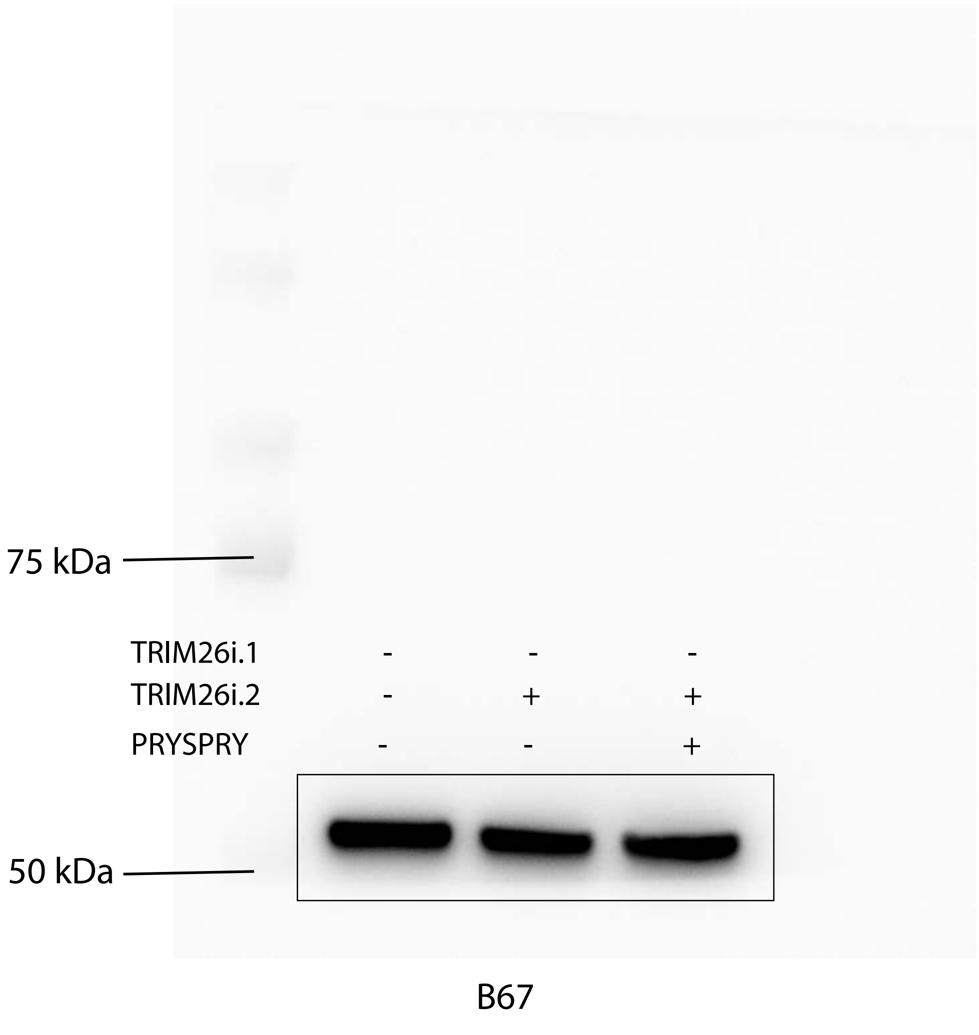

TUBULIN ANTIBODY

FIG 4A

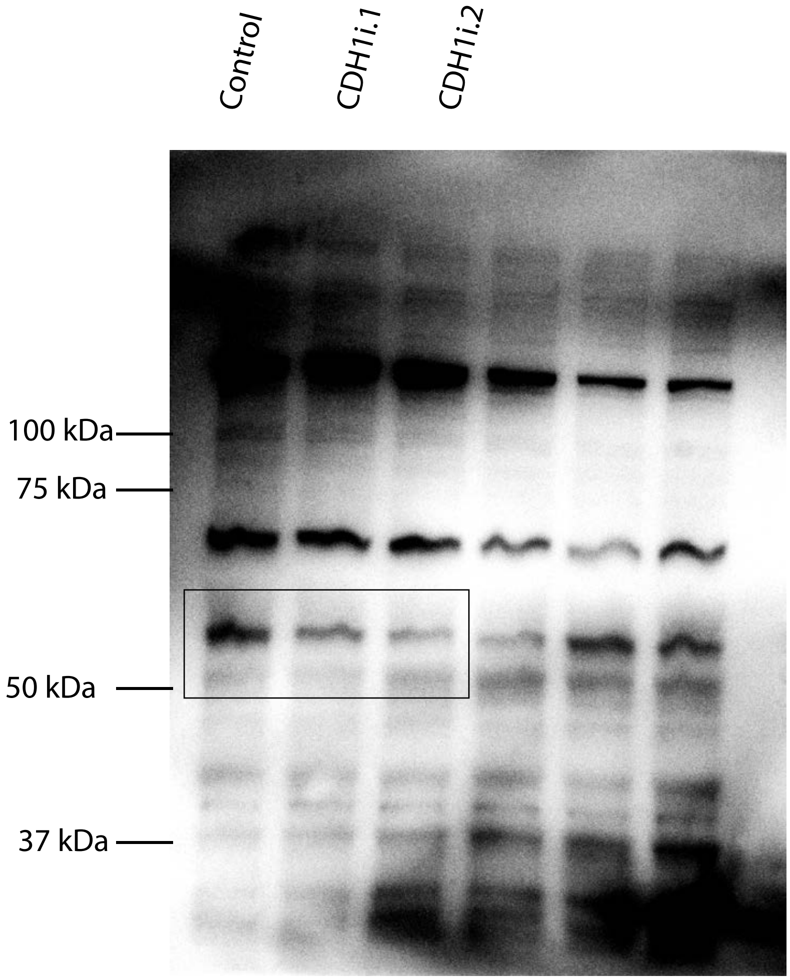

B67

CDH1 ANTIBODY

FIG 4A

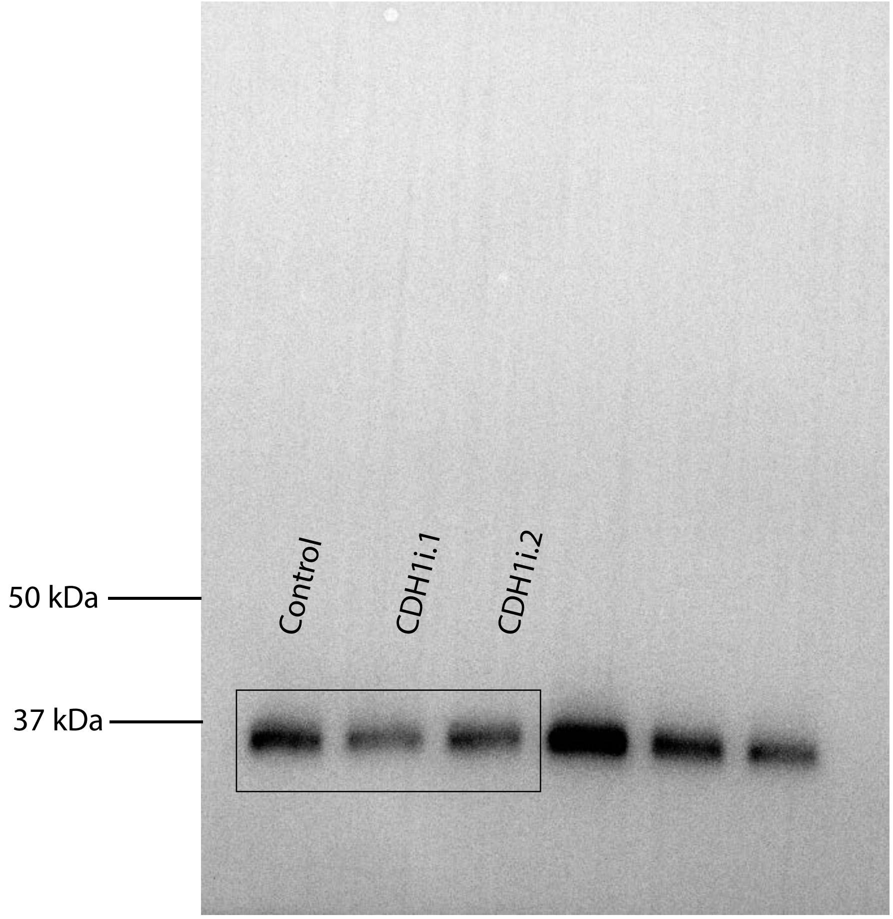

B67

SOX2 ANTIBODY (3579S)

FIG 4A

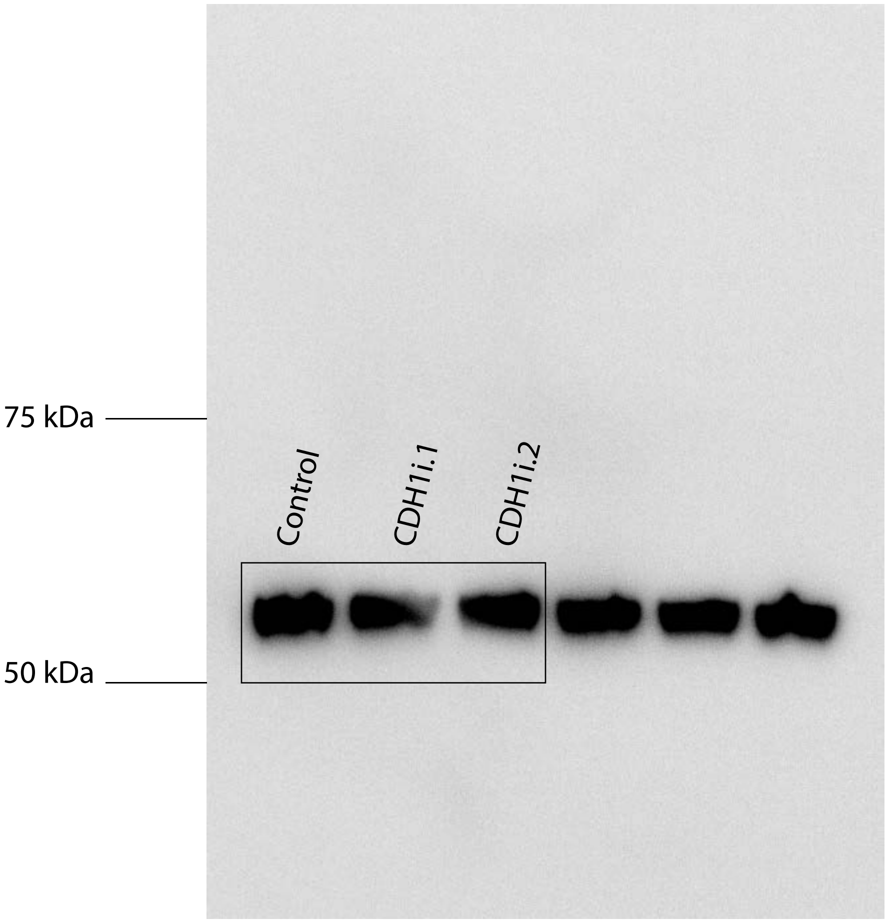

B67

TUBULIN ANTIBODY

FIG 4A

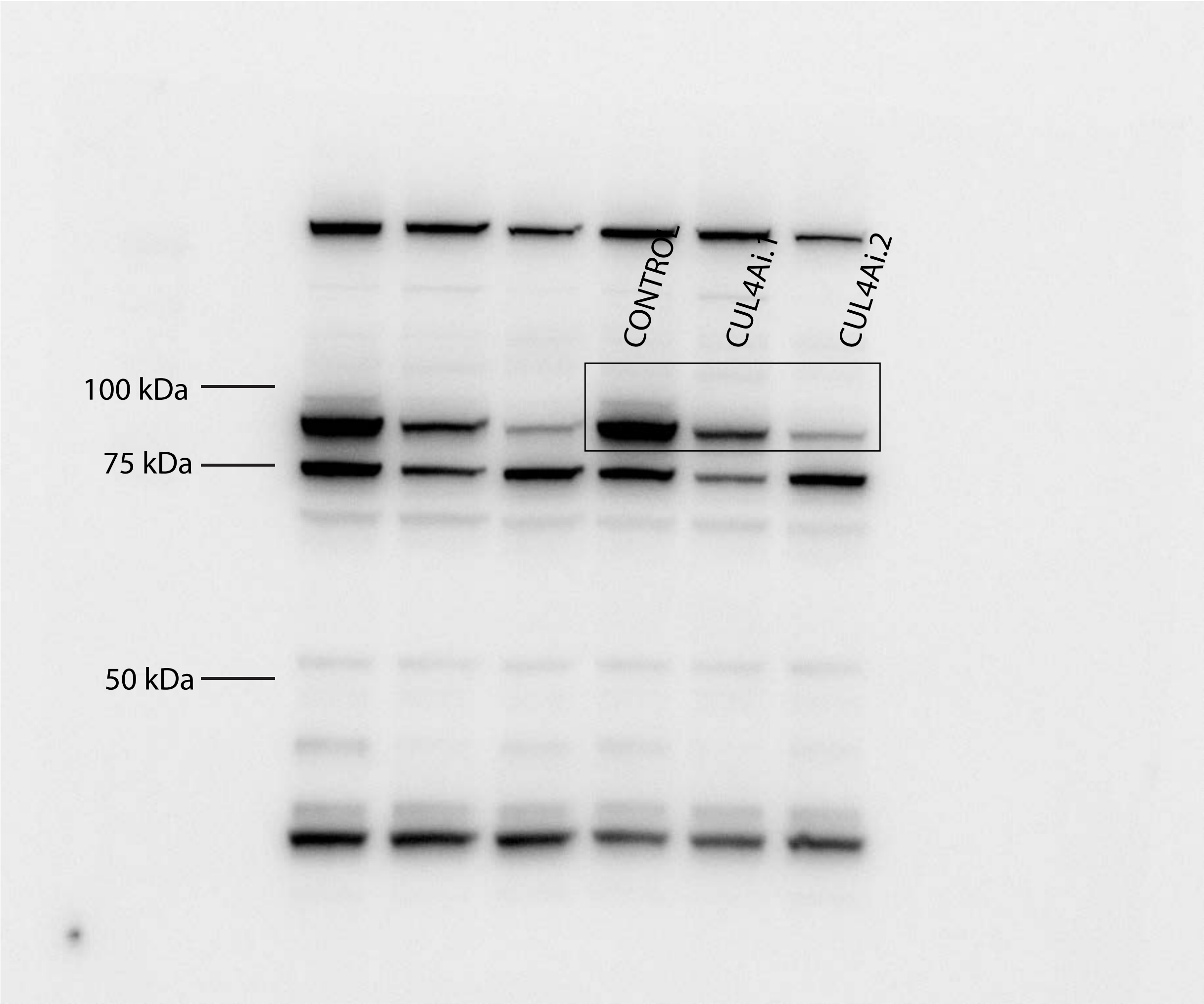

B67

CUL4A ANTIBODY

FIG 4A

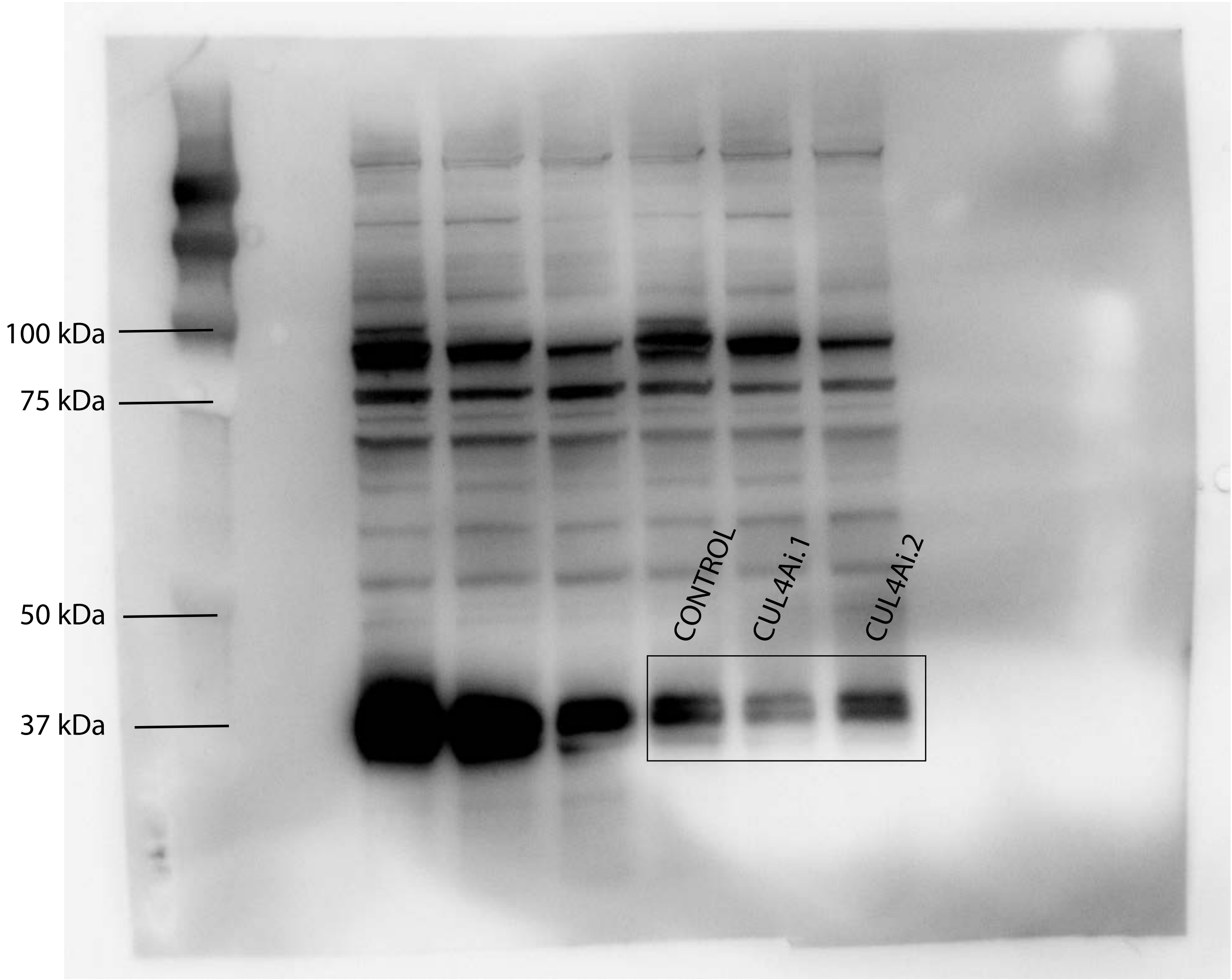

B67

SOX2 ANTIBODY

FIG 4A

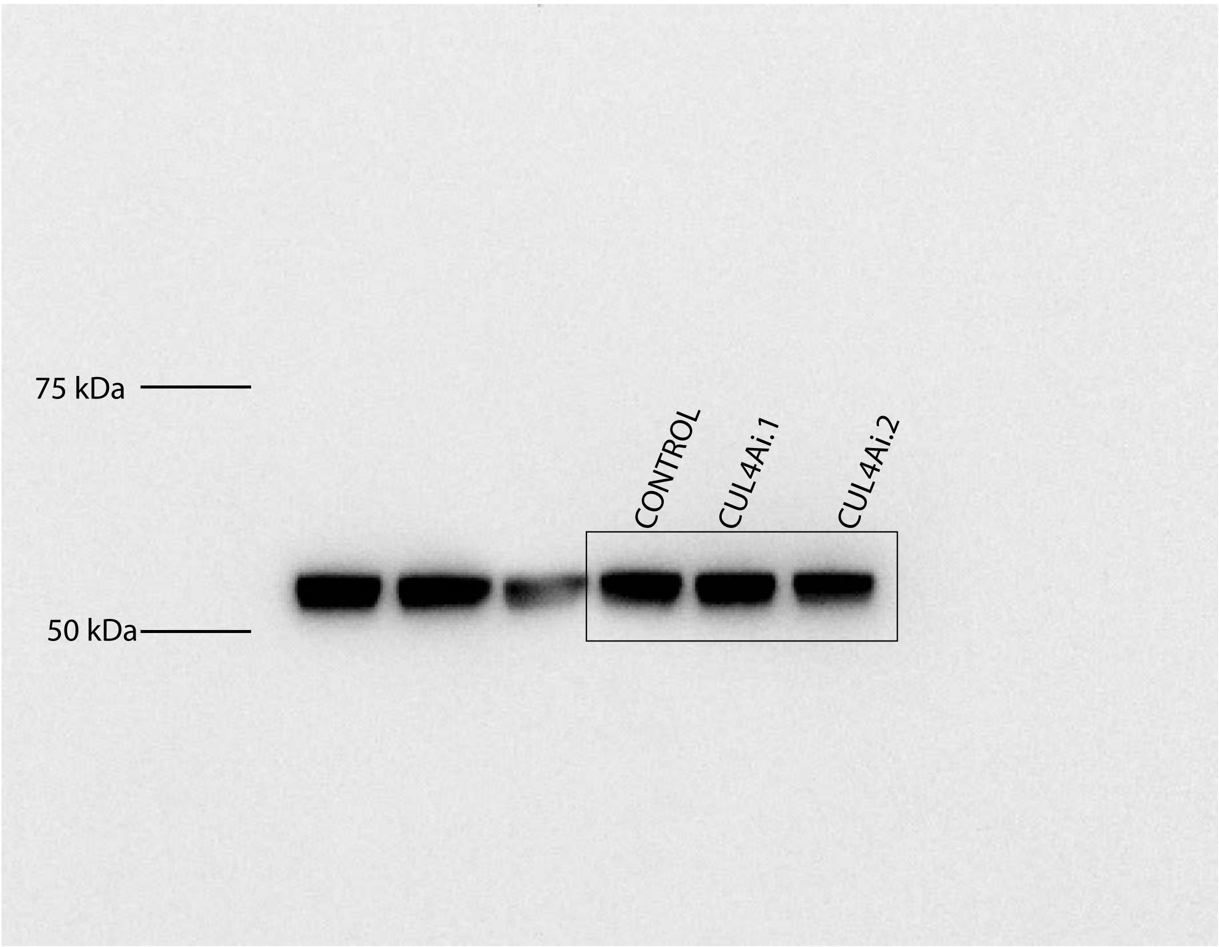

B67

TUBULIN ANTIBODY

FIG 4A

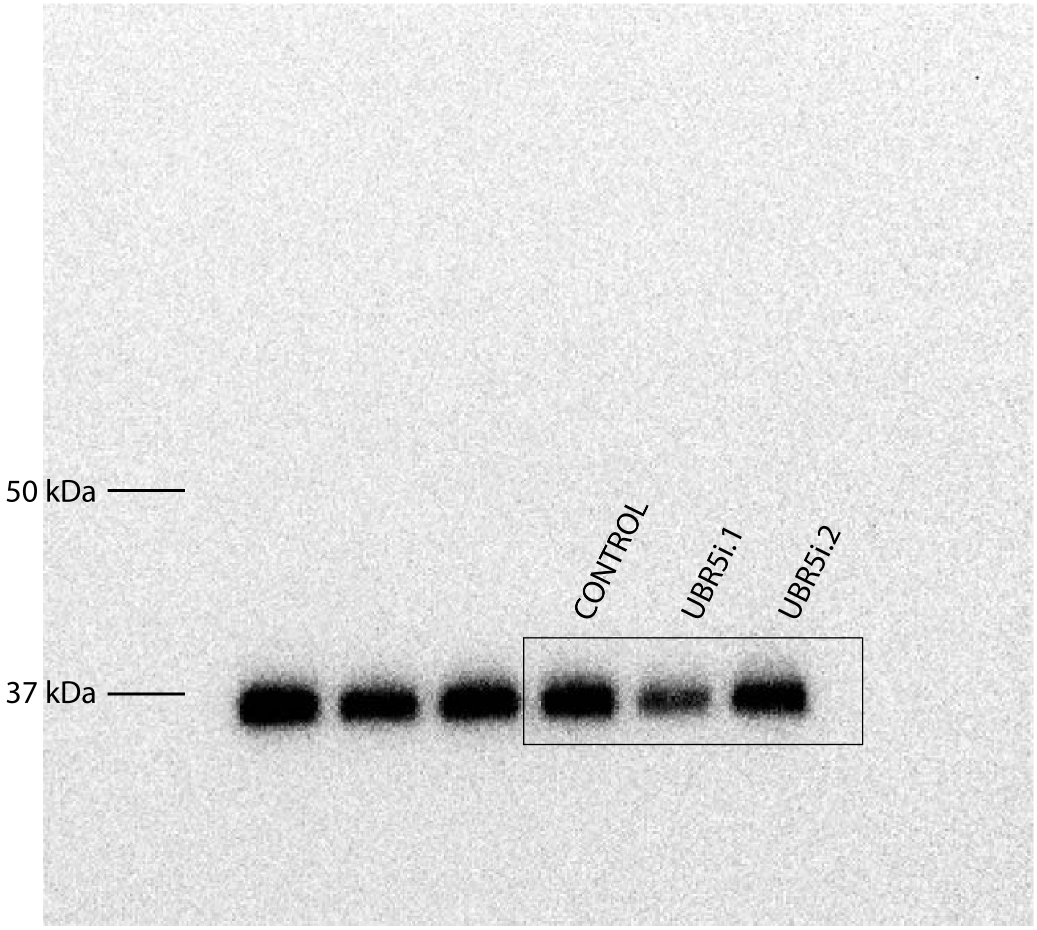

B67

SOX2 ANTIBODY

FIG 4A

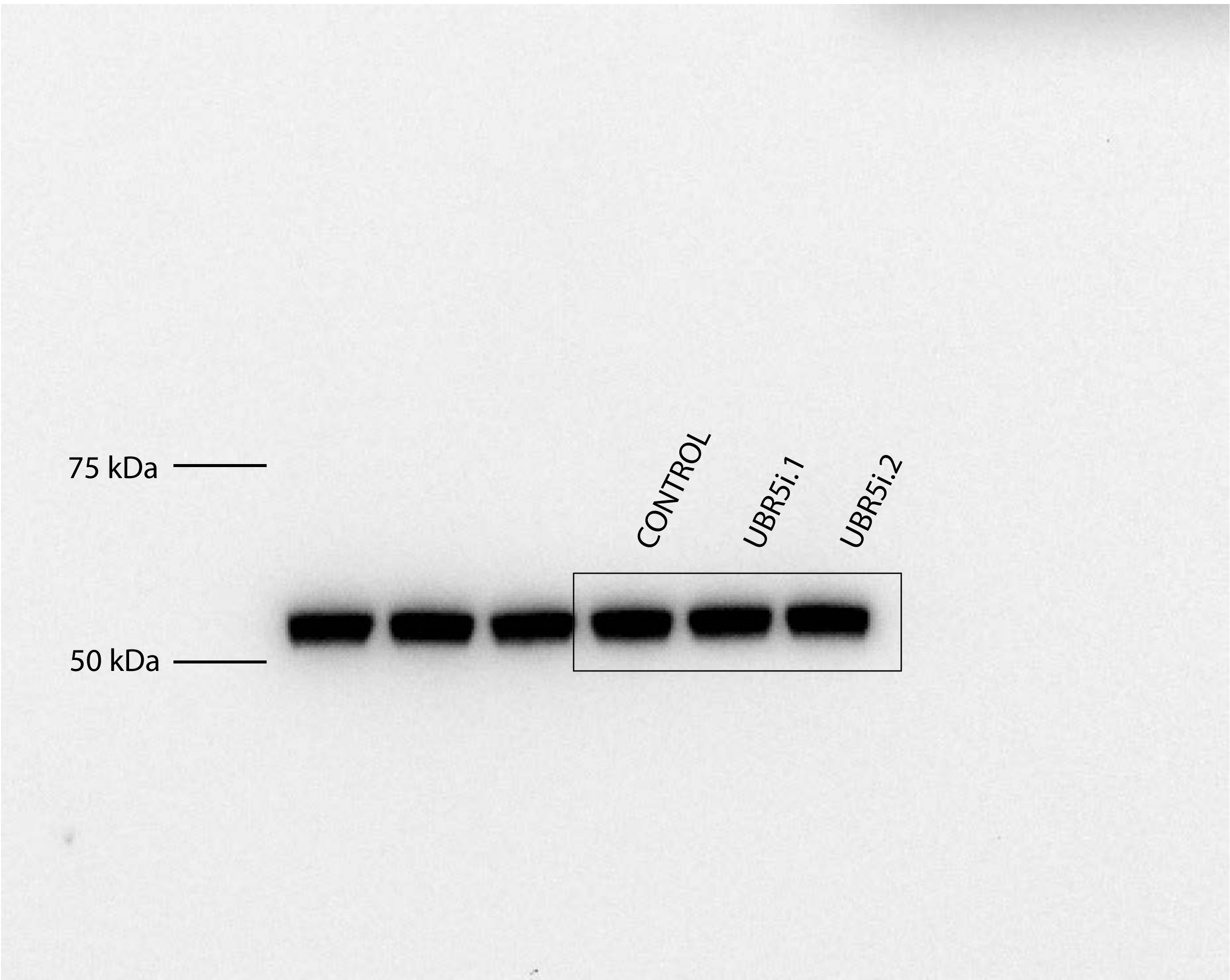

B67

TUBULIN ANTIBODY

FIG 4A

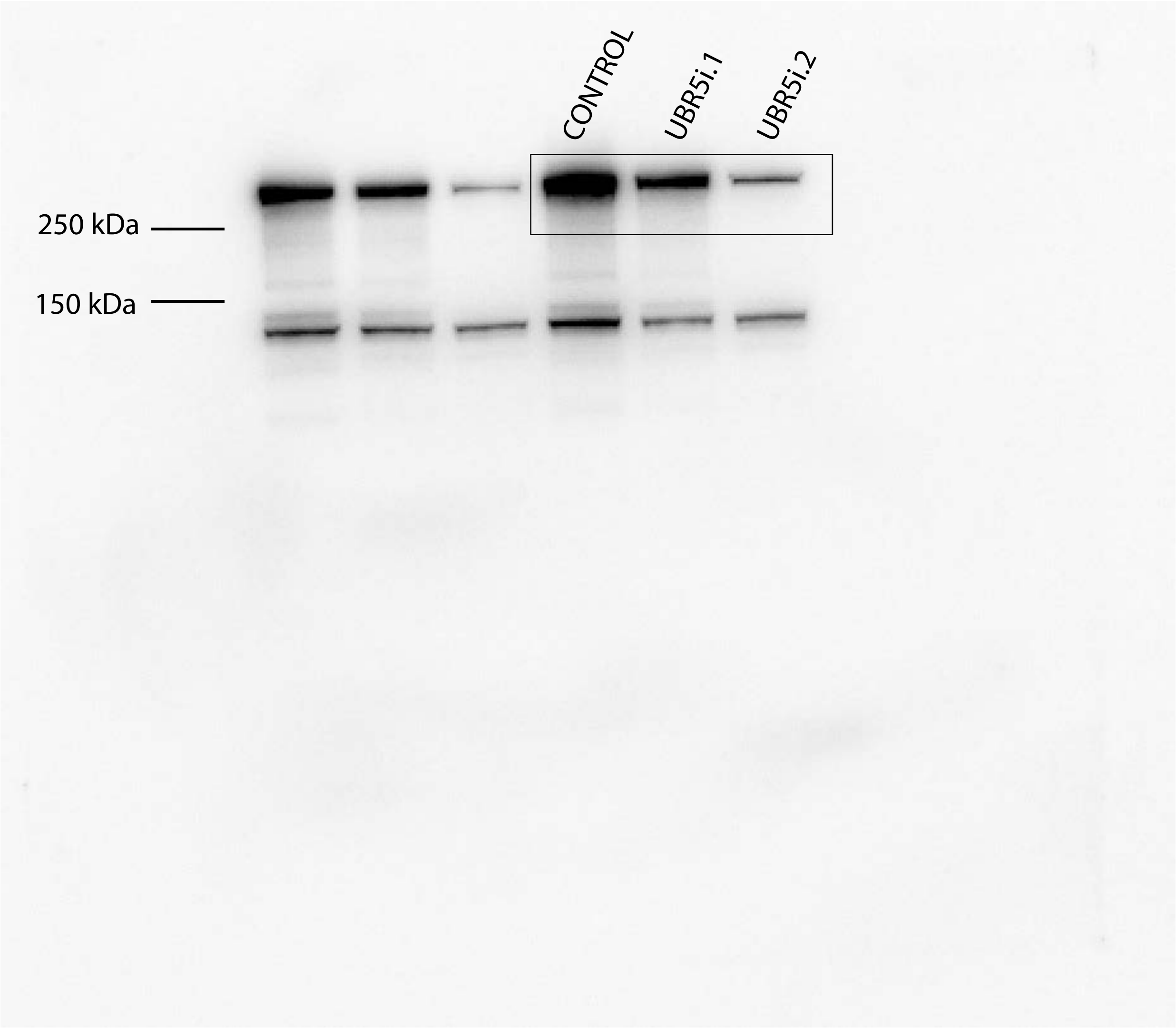

B67

UBR5 ANTIBODY

FIG 4A

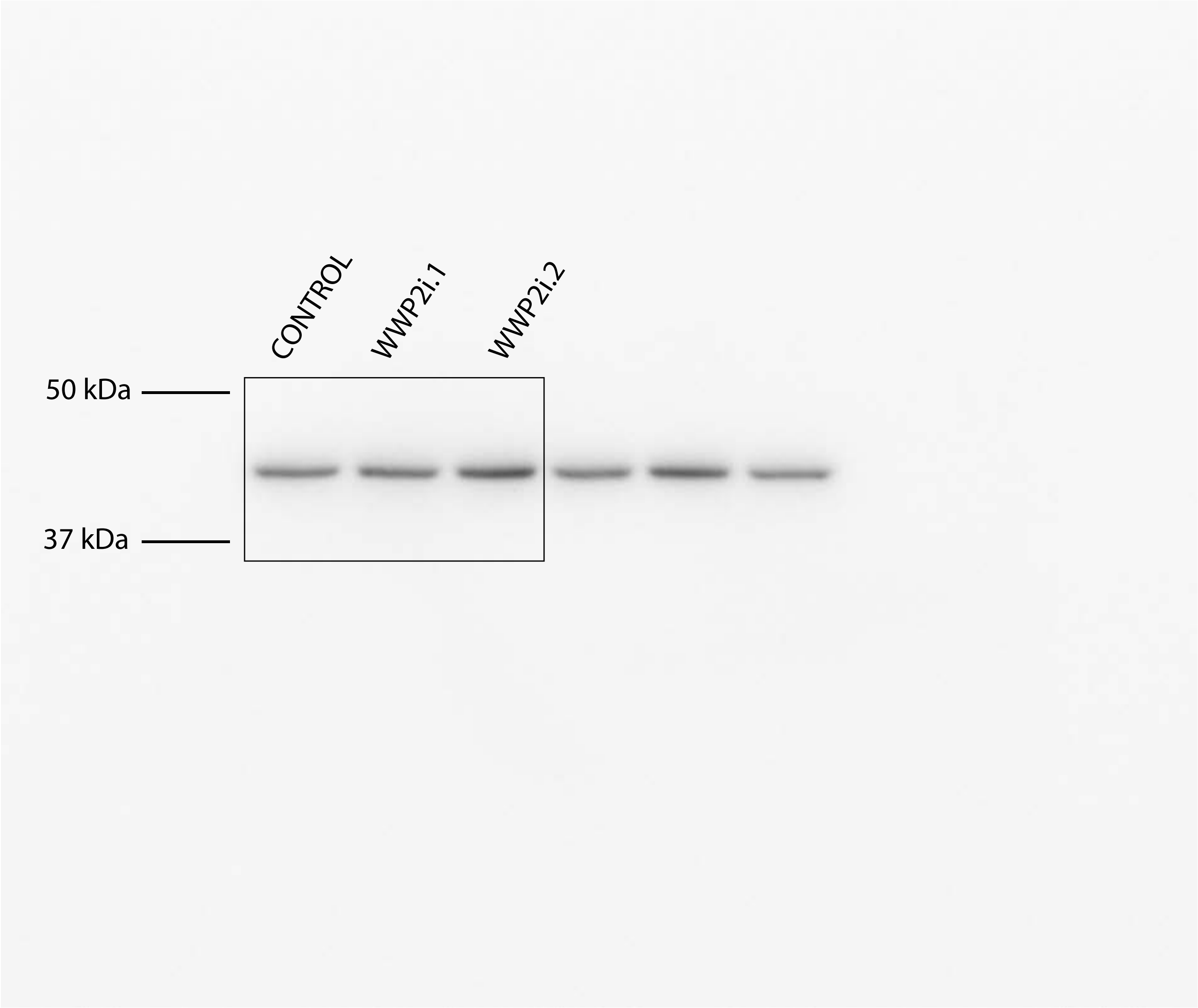

B67

ACTIN ANTIBODY

FIG 4A

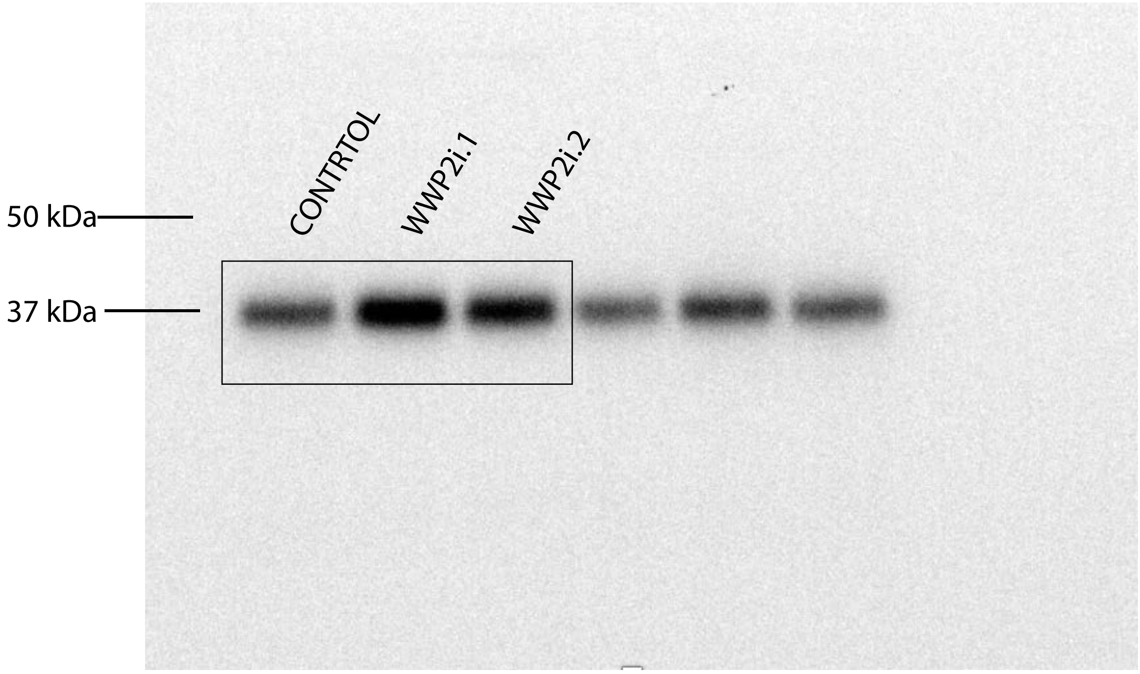

B67

SOX2 ANTIBODY

FIG 4A

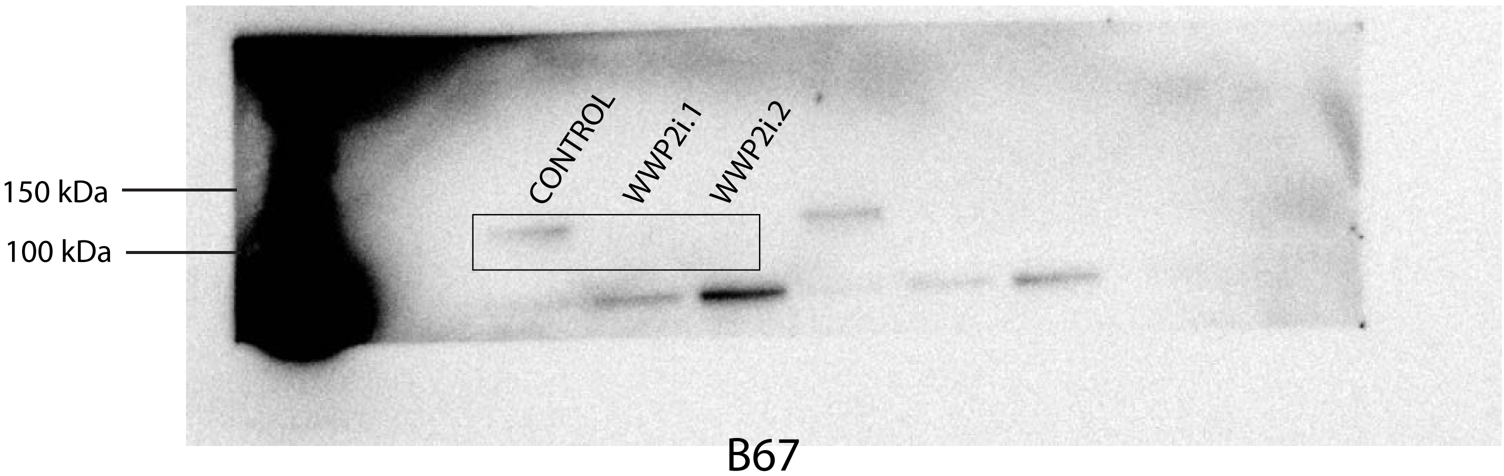

B67

WWP2 ANTIBODY

FIG 4B

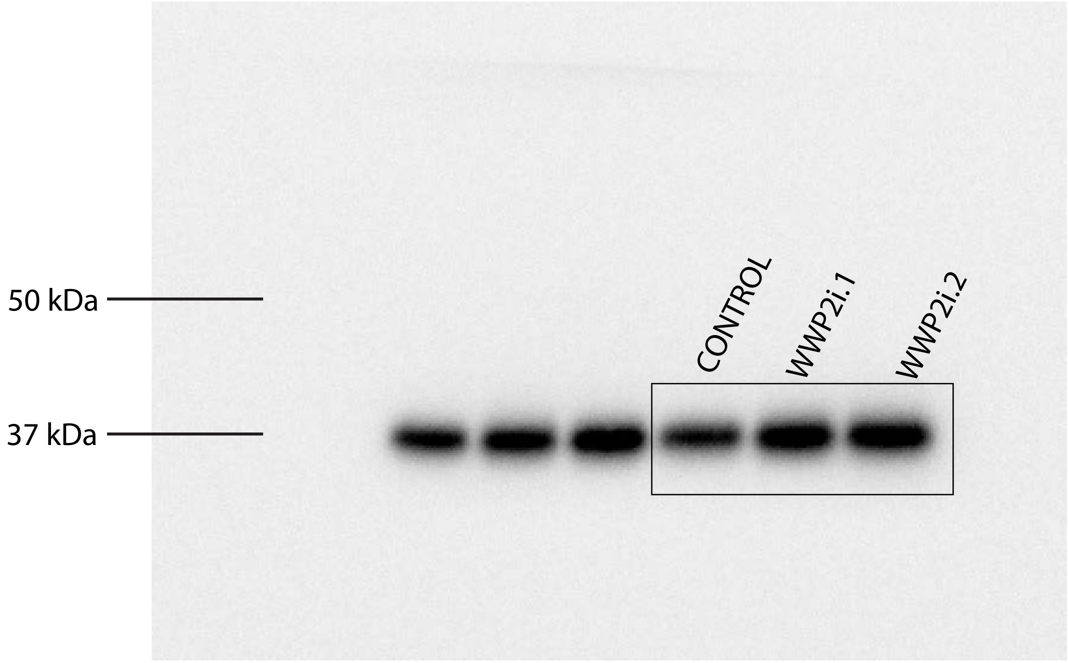

B36

SOX2 ANTIBODY

FIG 4B

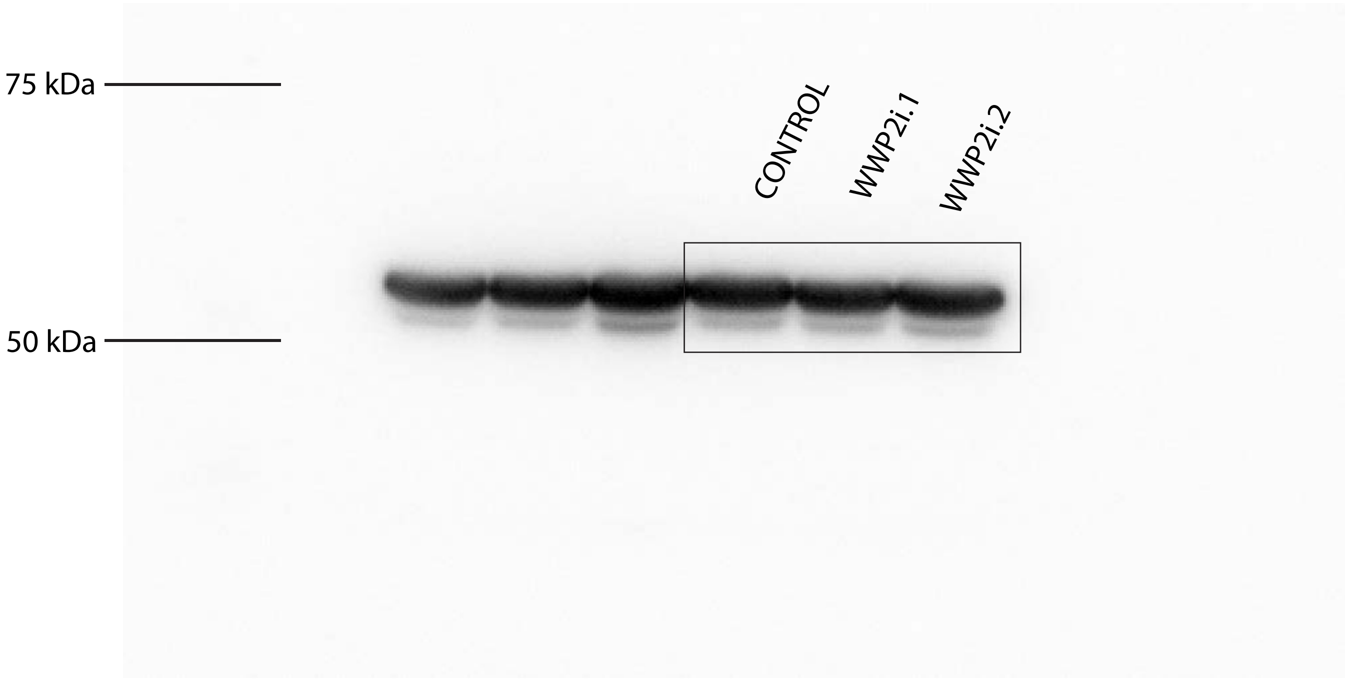

B36

TUBULIN ANTIBODY

FIG 4B

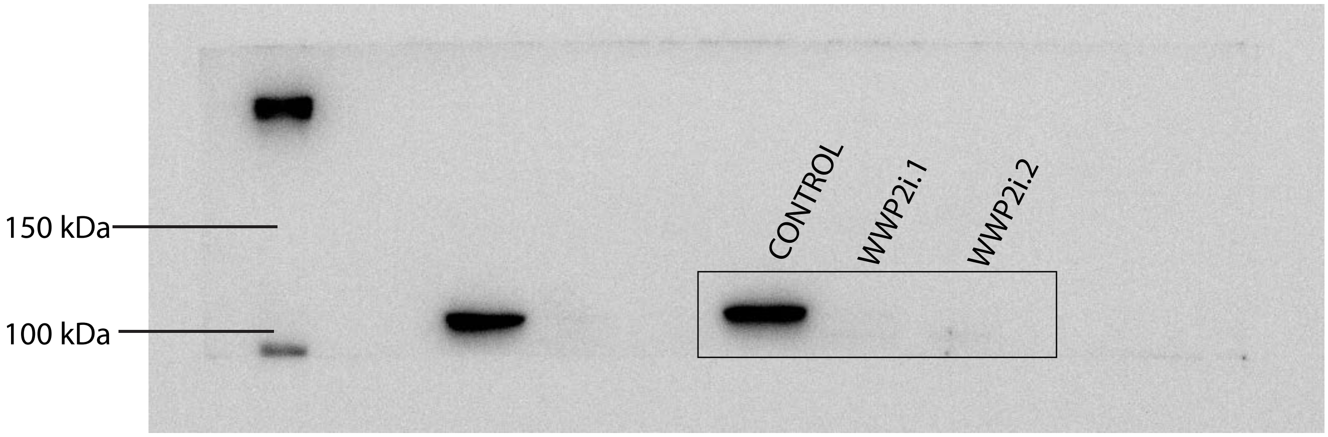

B36

WWP2 ANTIBODY

FIG 4B

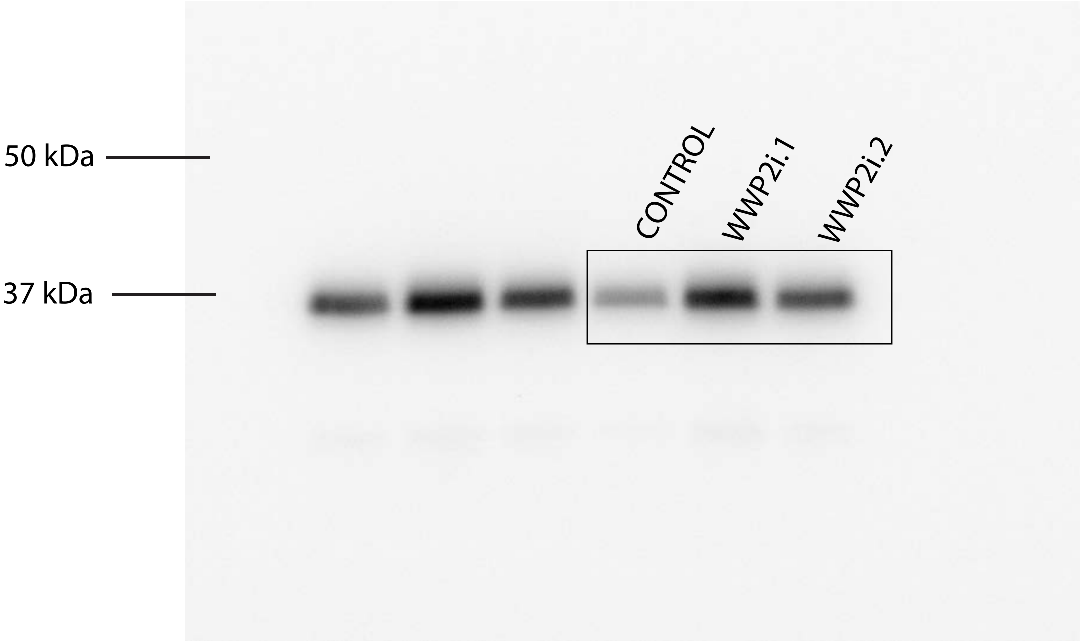

BT87

SOX2 ANTIBODY

FIG 4B

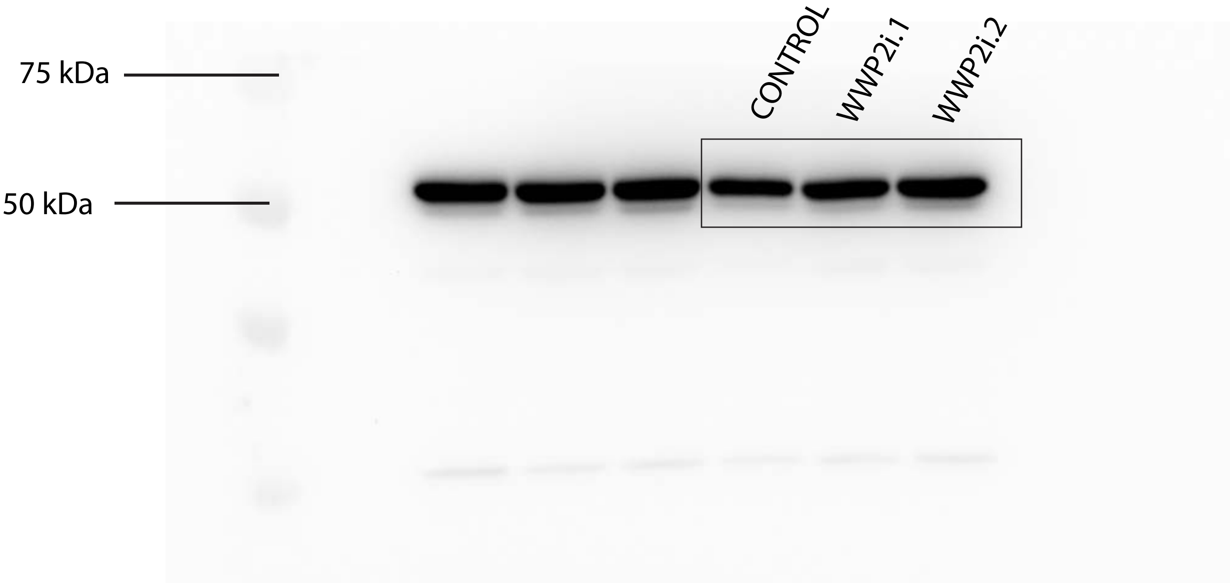

BT87

TUBULIN ANTIBODY

FIG 4B

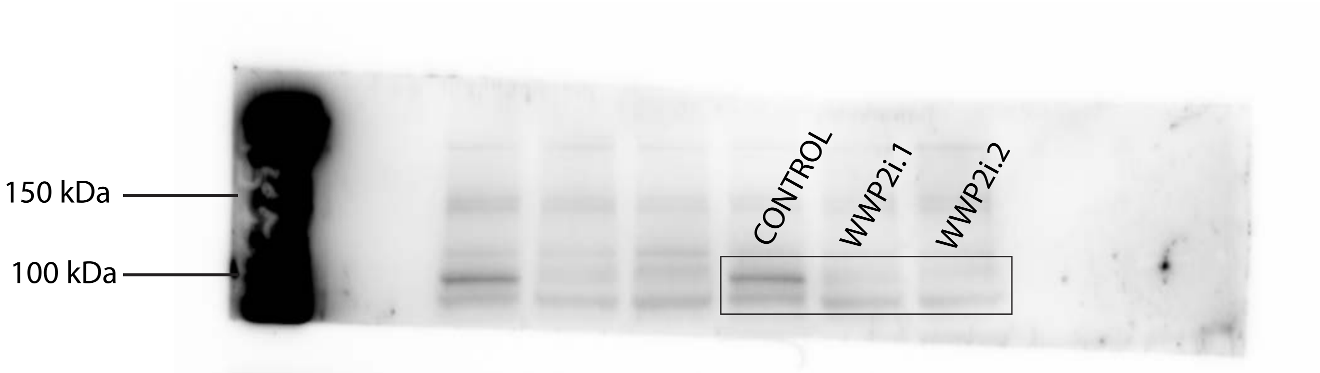

BT87

WWP2 ANTIBODY

FIG 4D

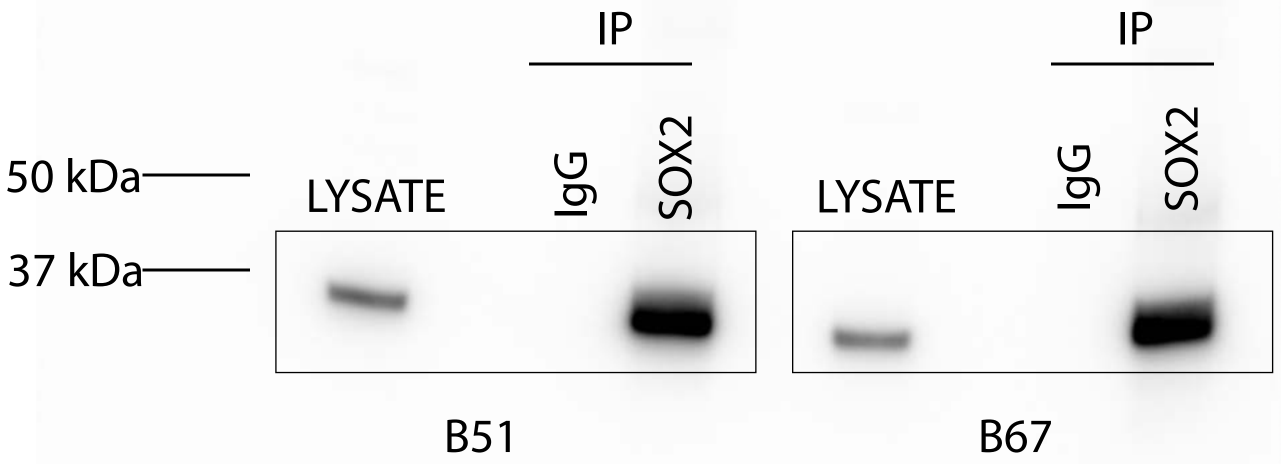

SOX2 ANTIBODY

FIG 4D

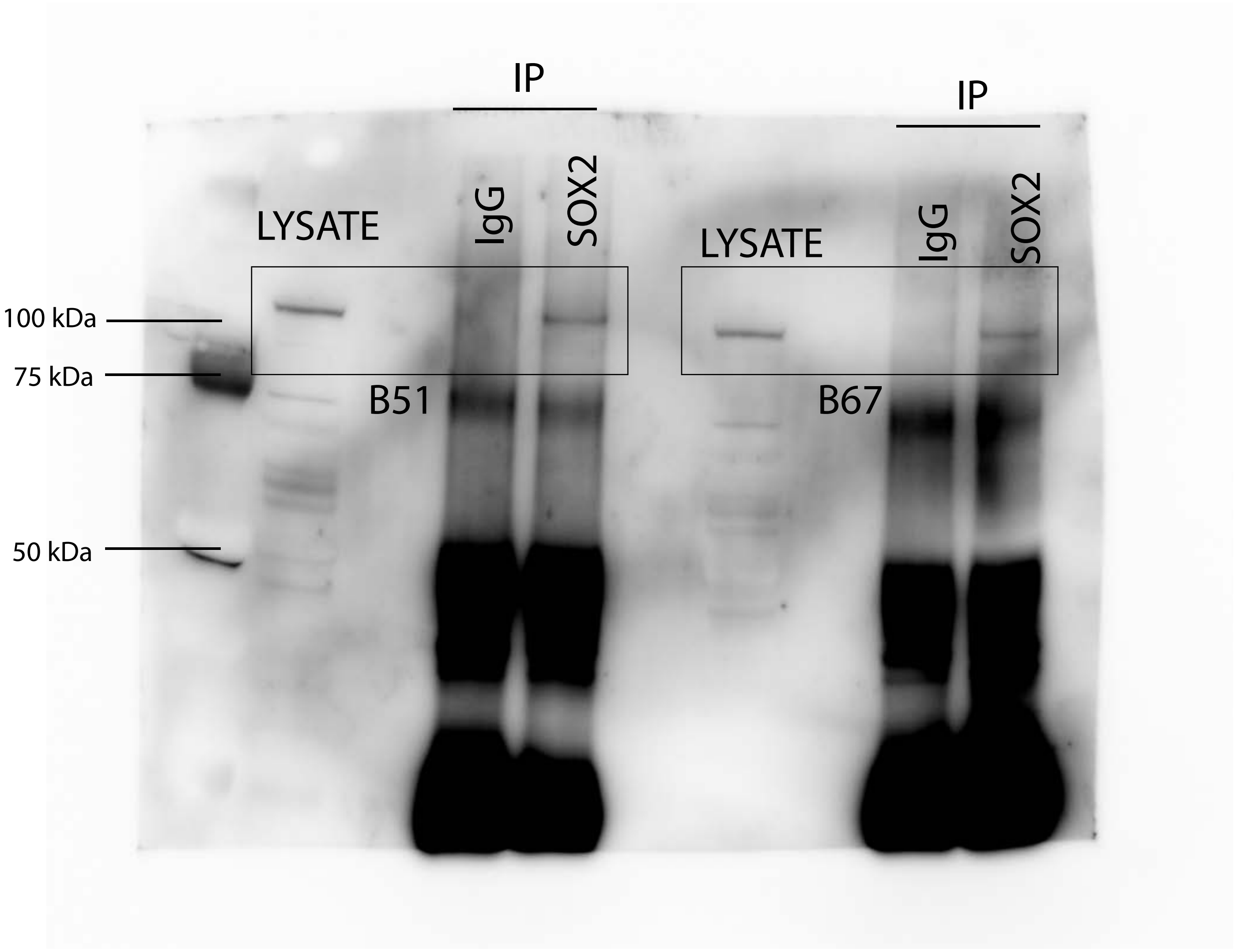

WWP2 ANTIBODY

FIG 4E

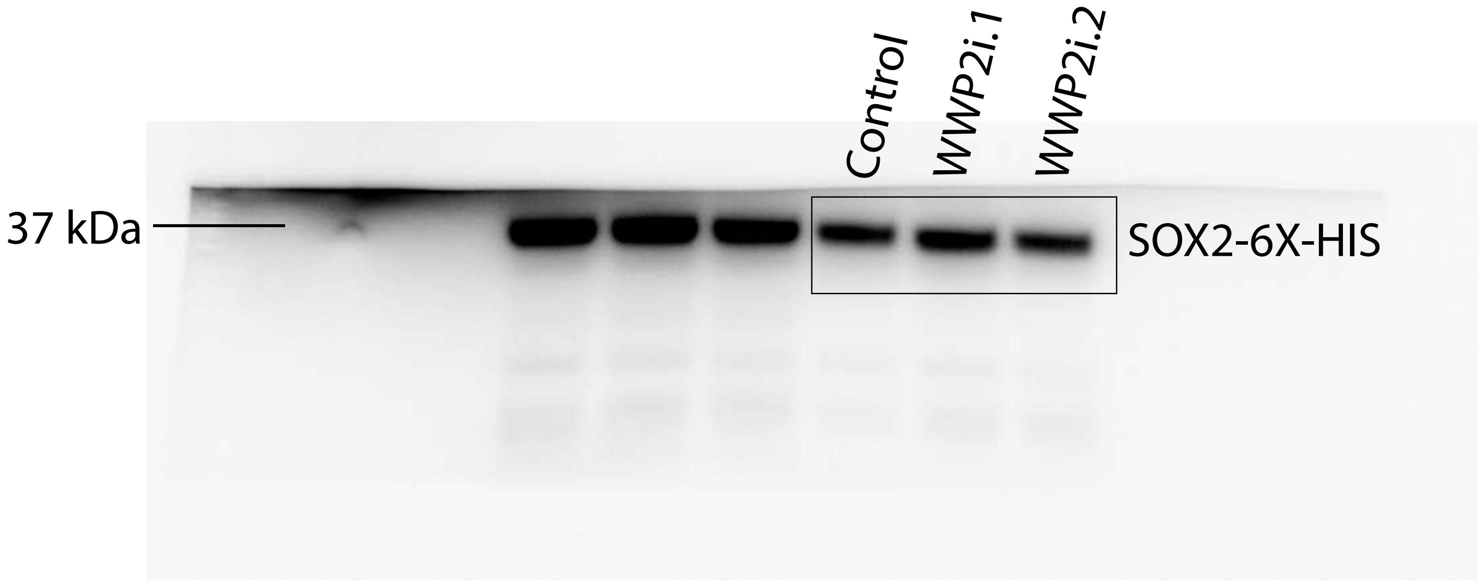

B67 INPUT

HIS-TAG ANTIBODY

FIG 4E

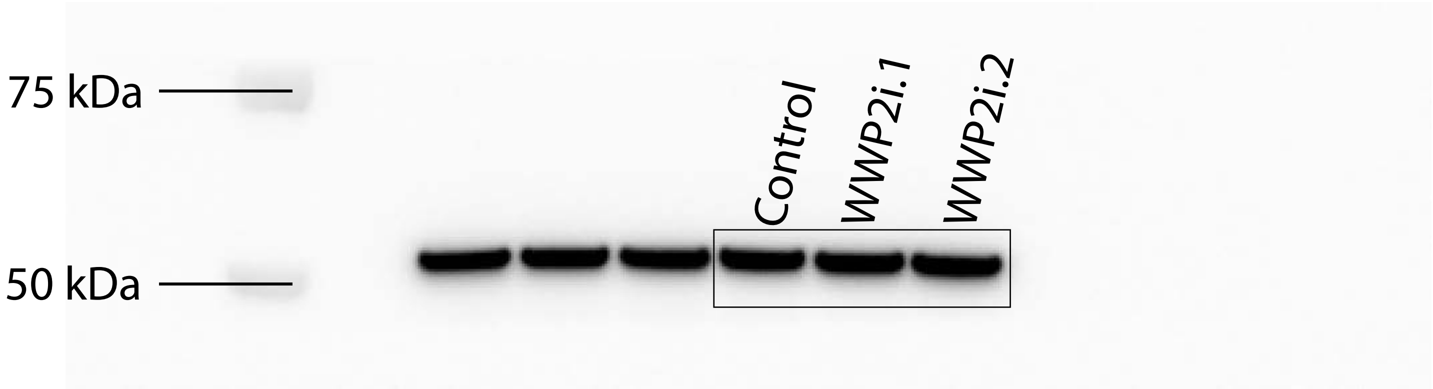

B67 INPUT

TUBULIN ANTIBODY

FIG 4E

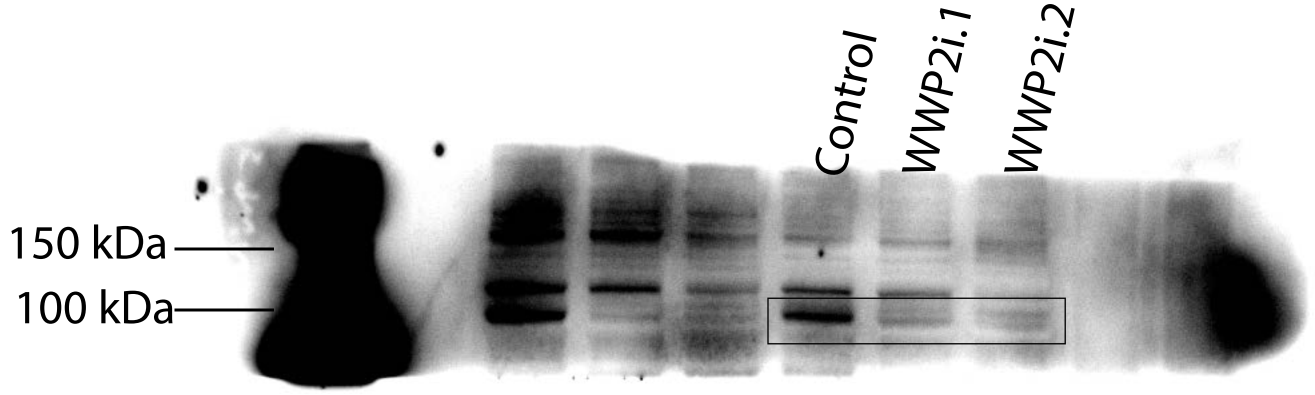

B67 INPUT

WWP2 ANTIBODY

FIG 4E

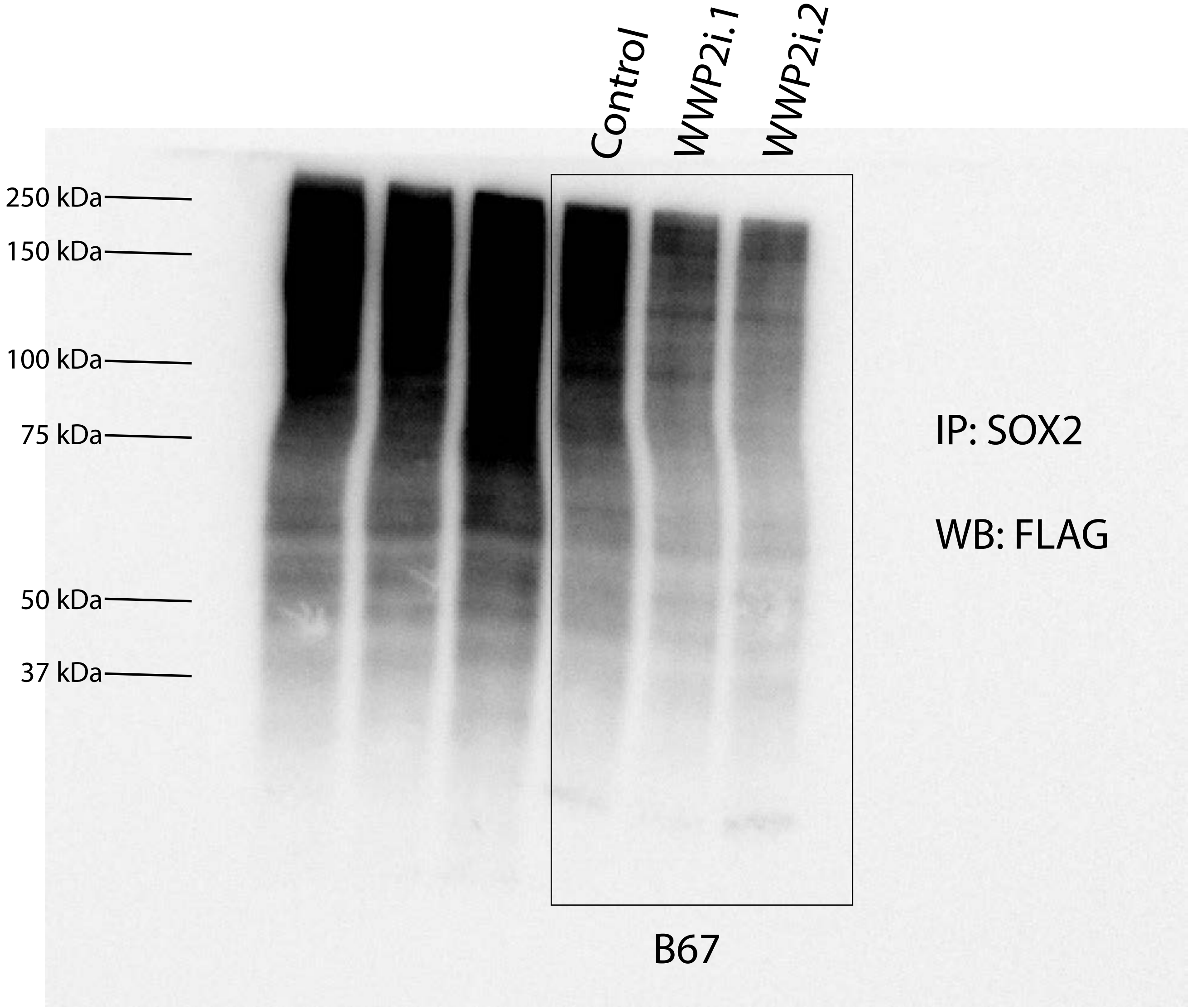

FLAG TAG ANTIBODY

FIG 4E

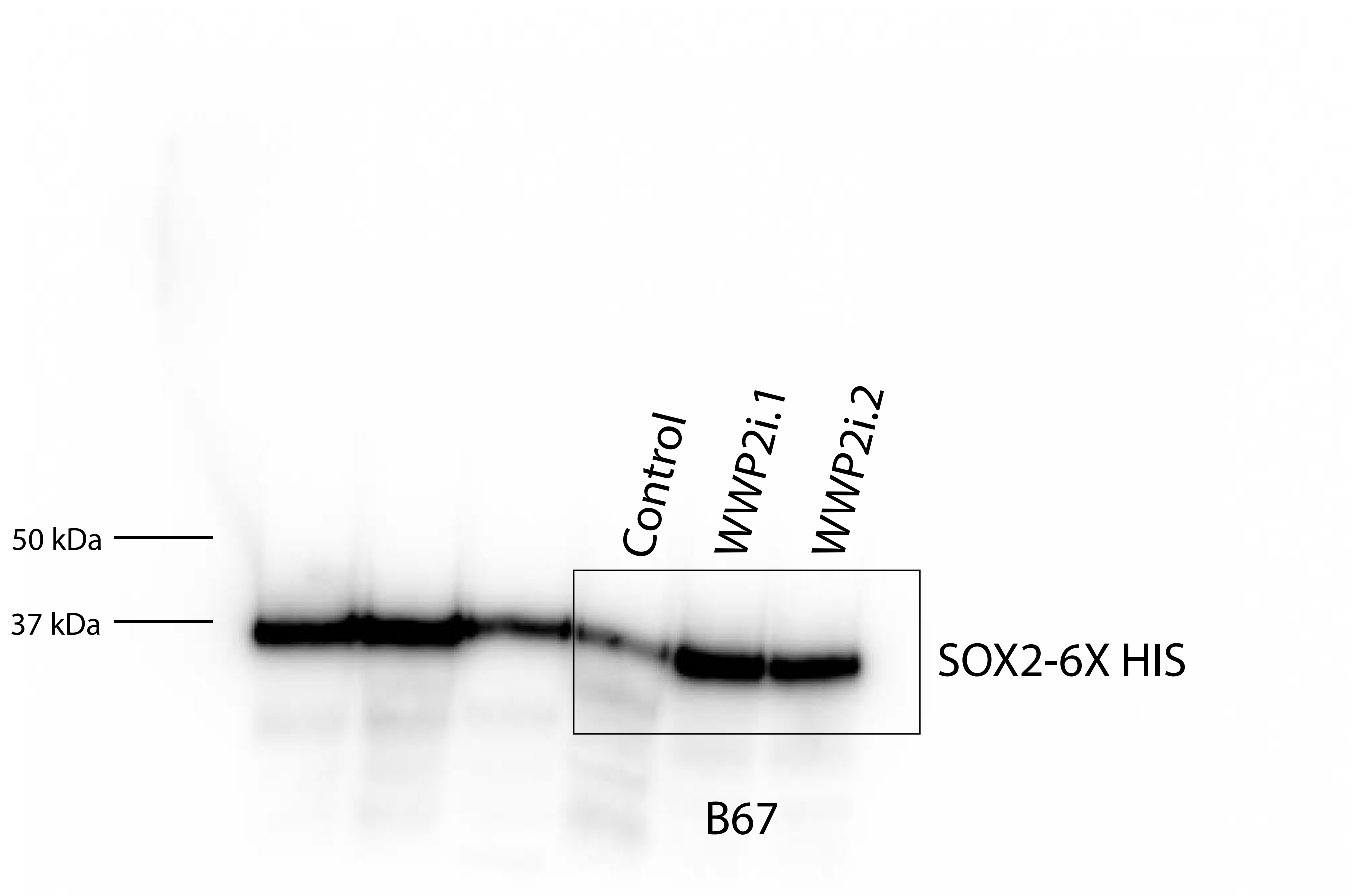

IP: SOX2-6X-HIS

WB: HIS-TAG ANTIBODY

FIG 5A

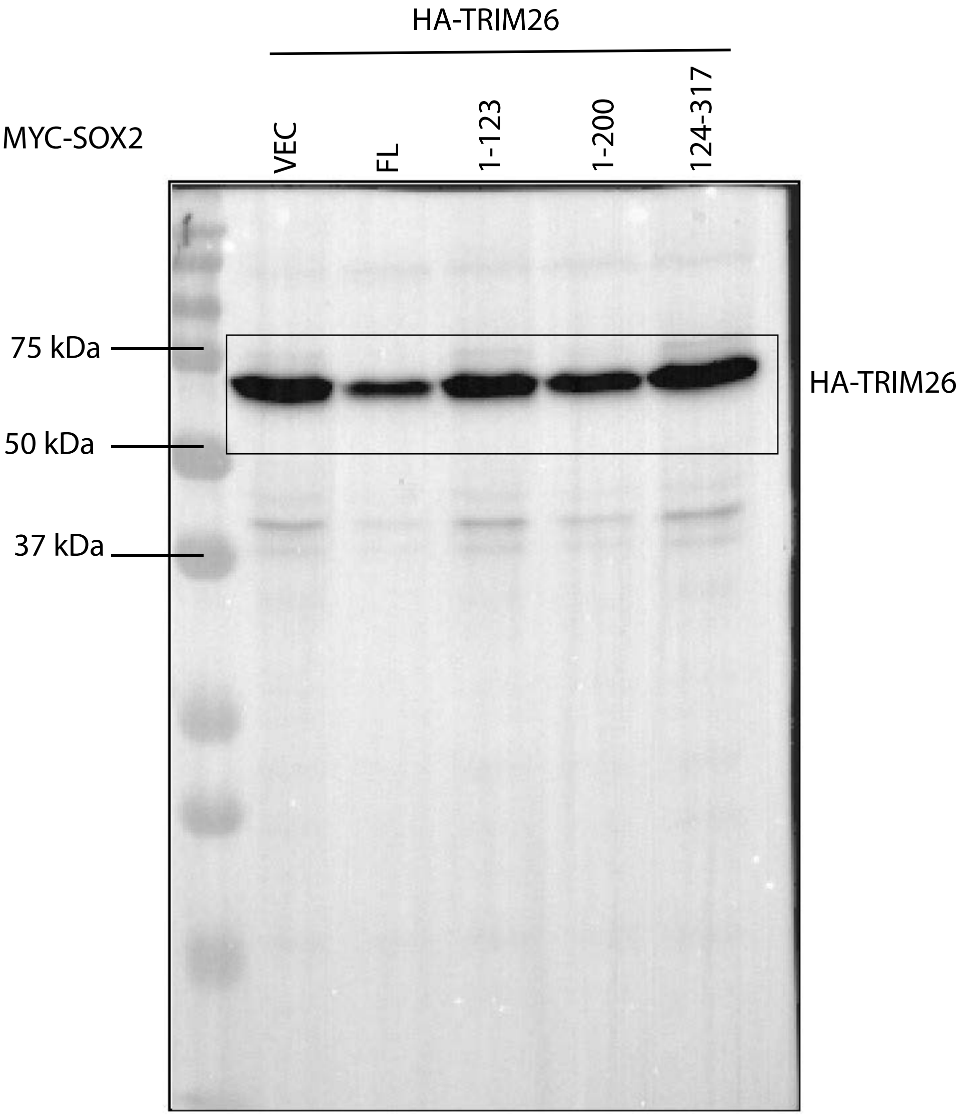

INPUT BLOT

HA-TAG ANTIBODY

FIG 5A

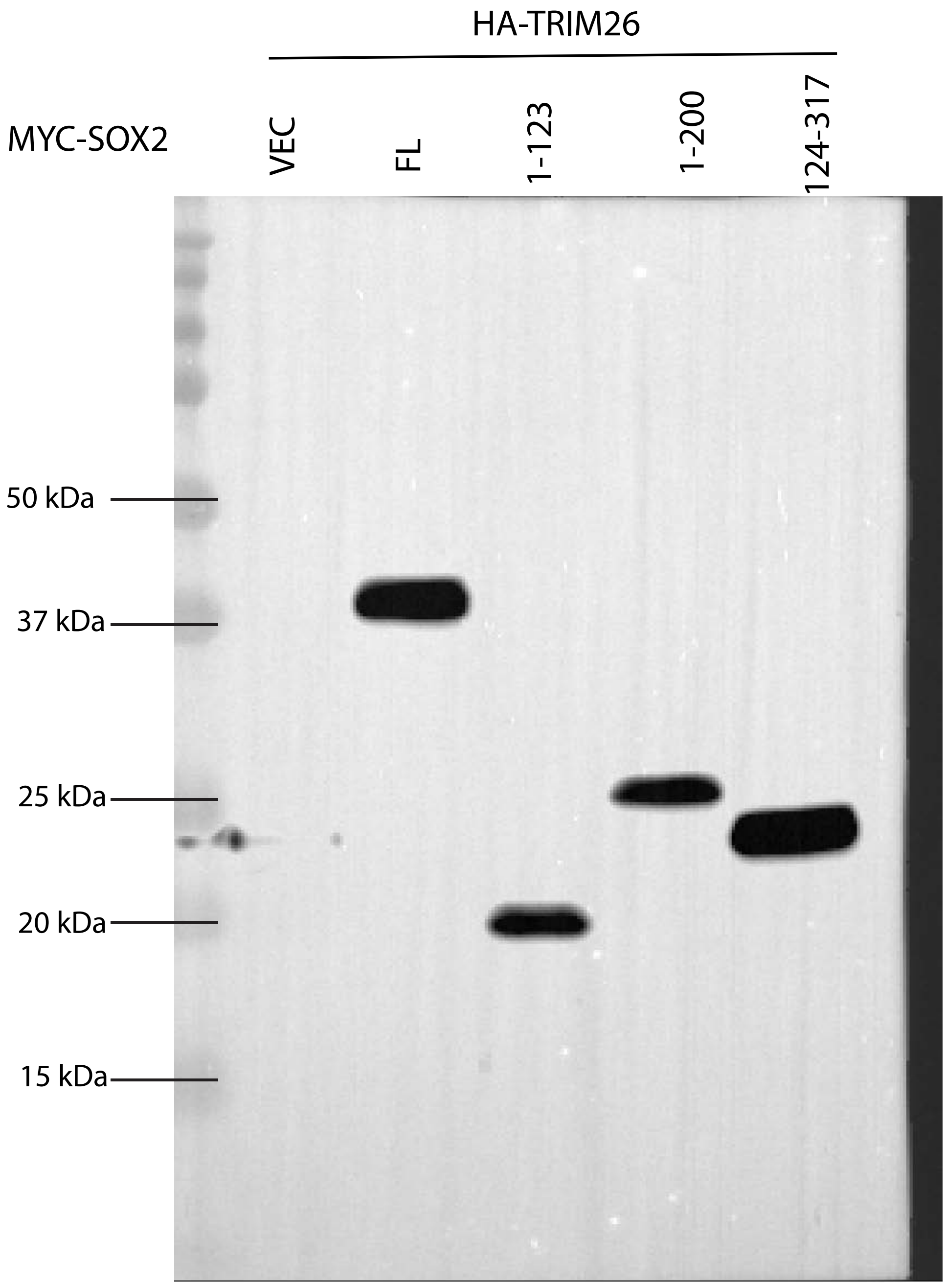

INPUT BLOT

MYC-TAG ANTIBODY

FIG 5A

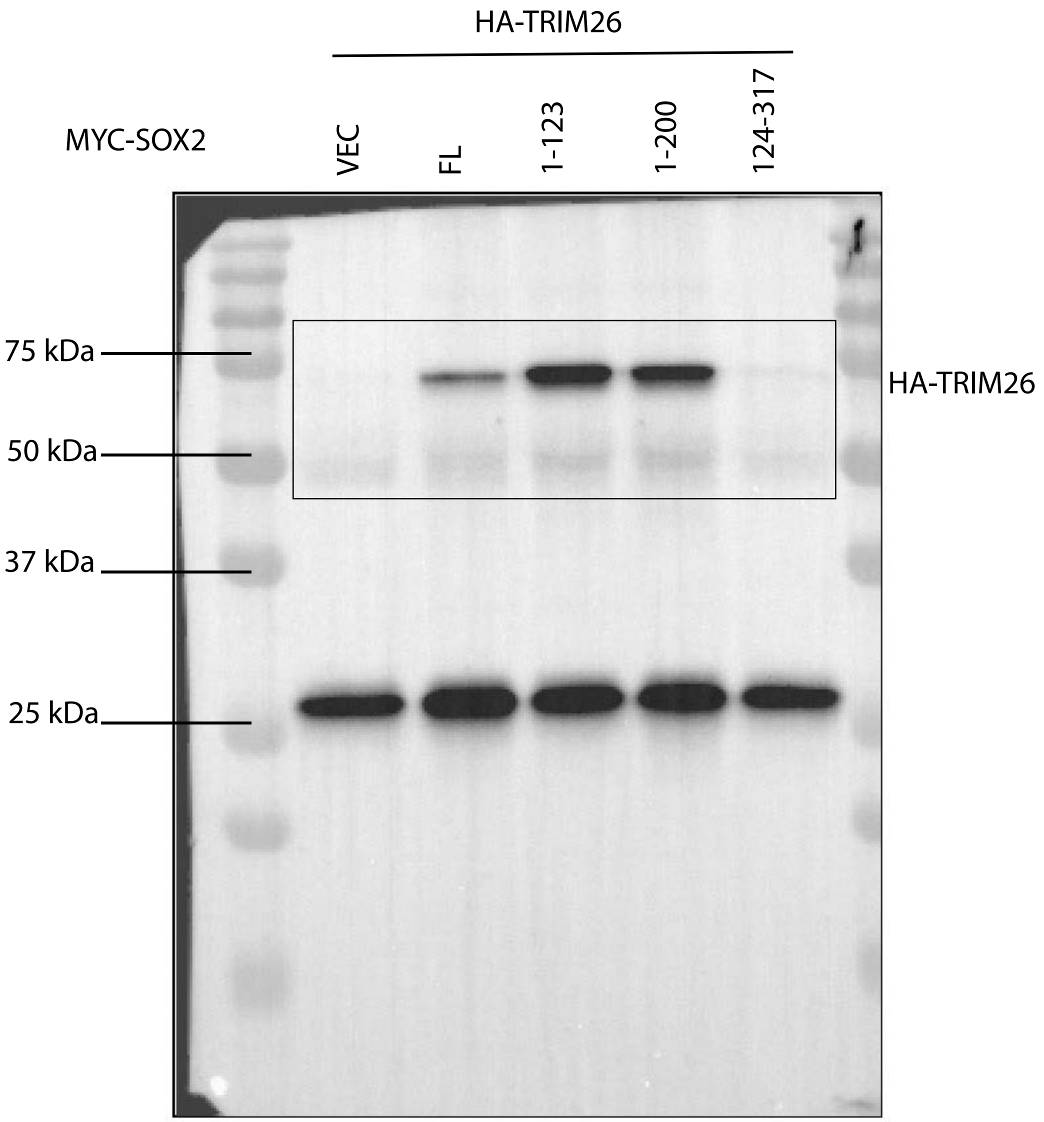

MYC IP BLOT

HA-TAG ANTIBODY

FIG 5A

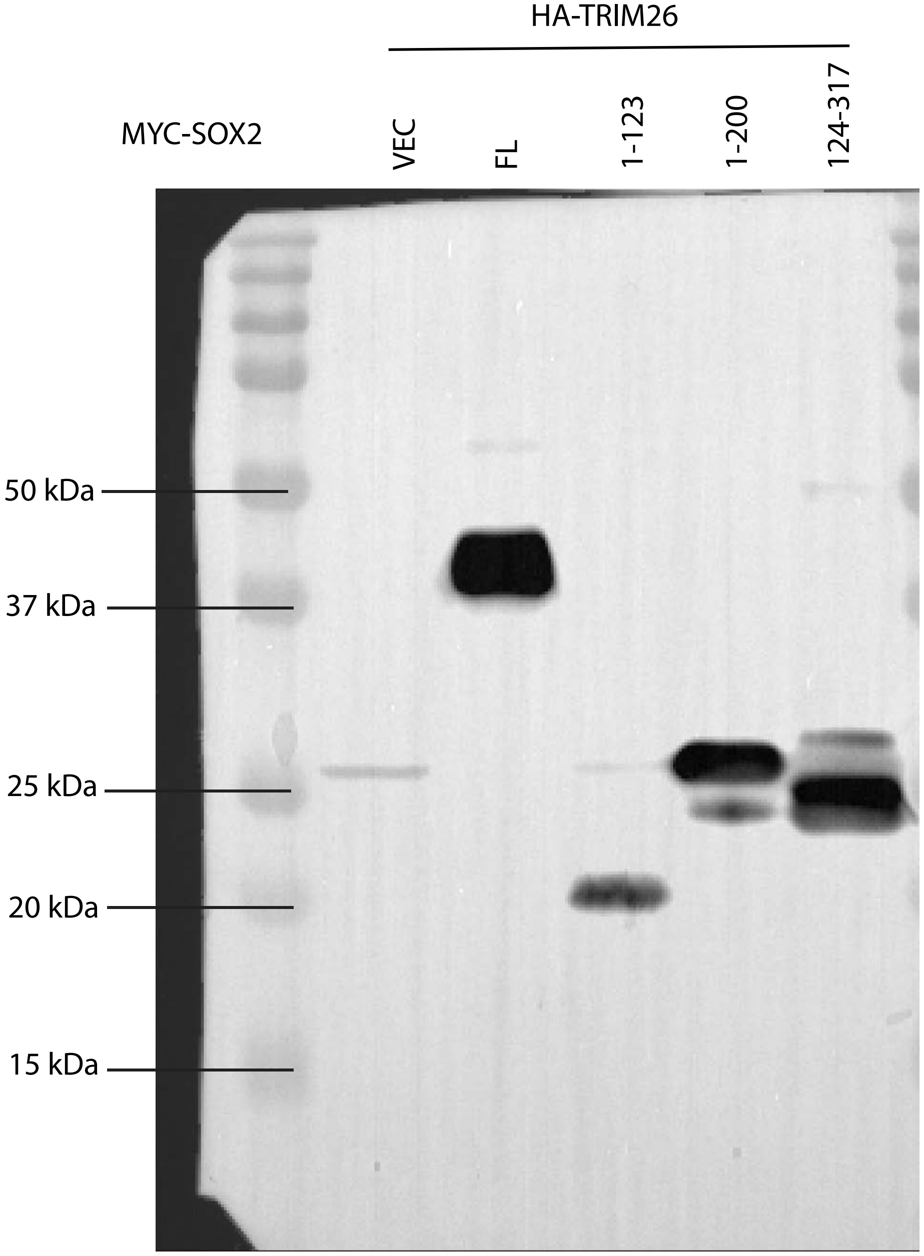

MYC IP BLOT

MYC-TAG ANTIBODY

FIG 5B

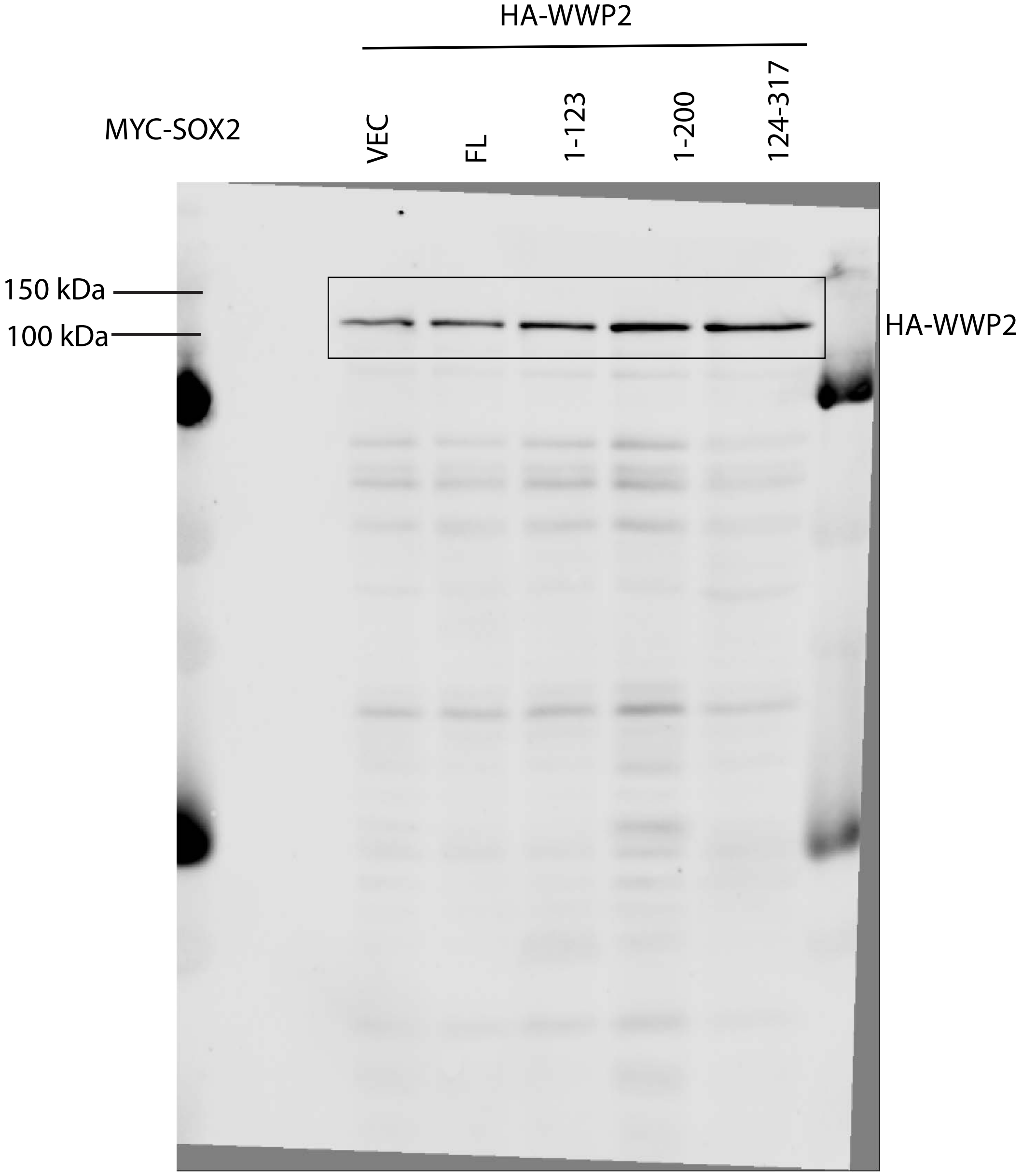

INPUT BLOT

HA-TAG ANTIBODY

FIG 5B

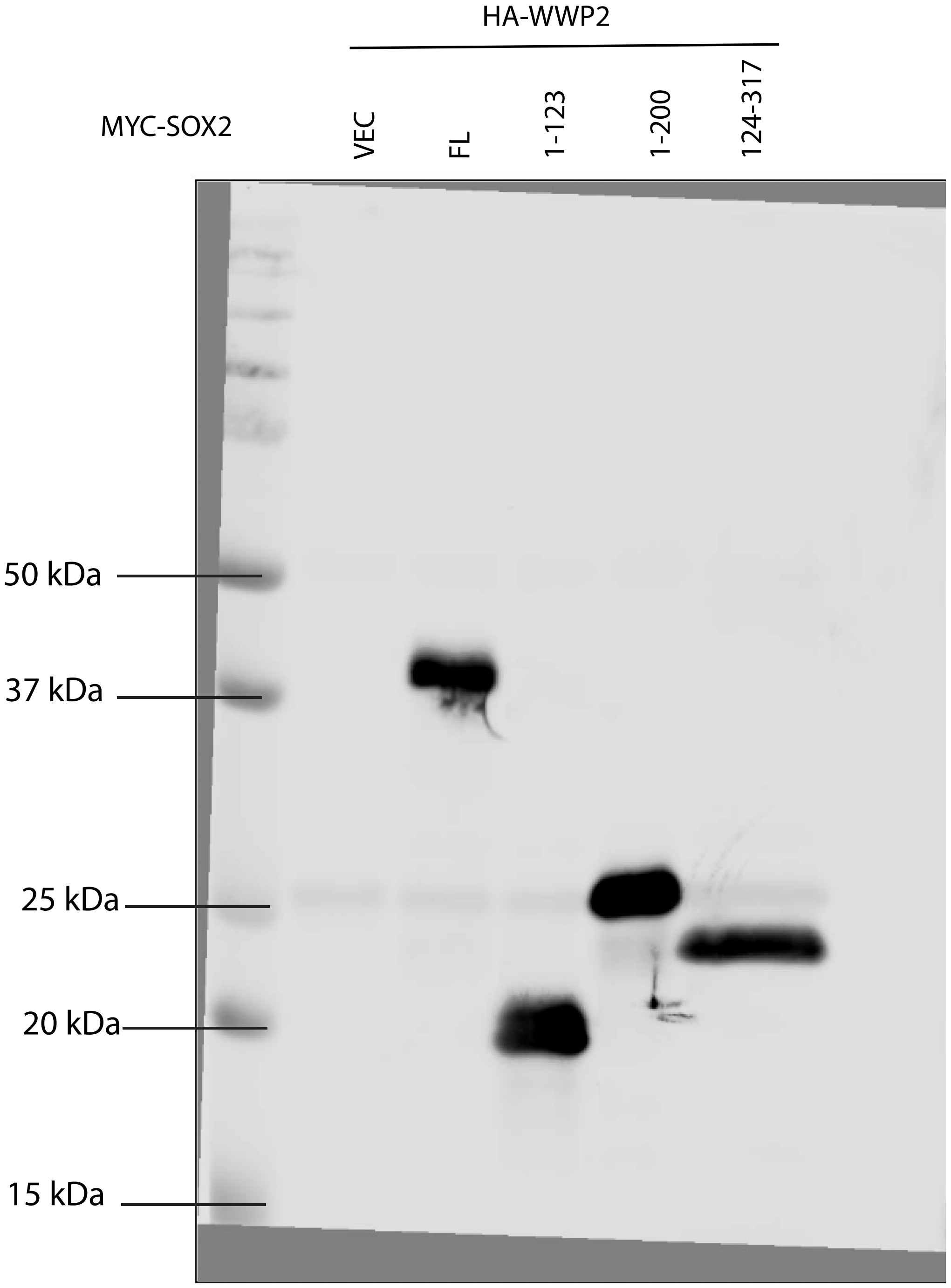

INPUT BLOT

MYC-TAG ANTIBODY

FIG 5B

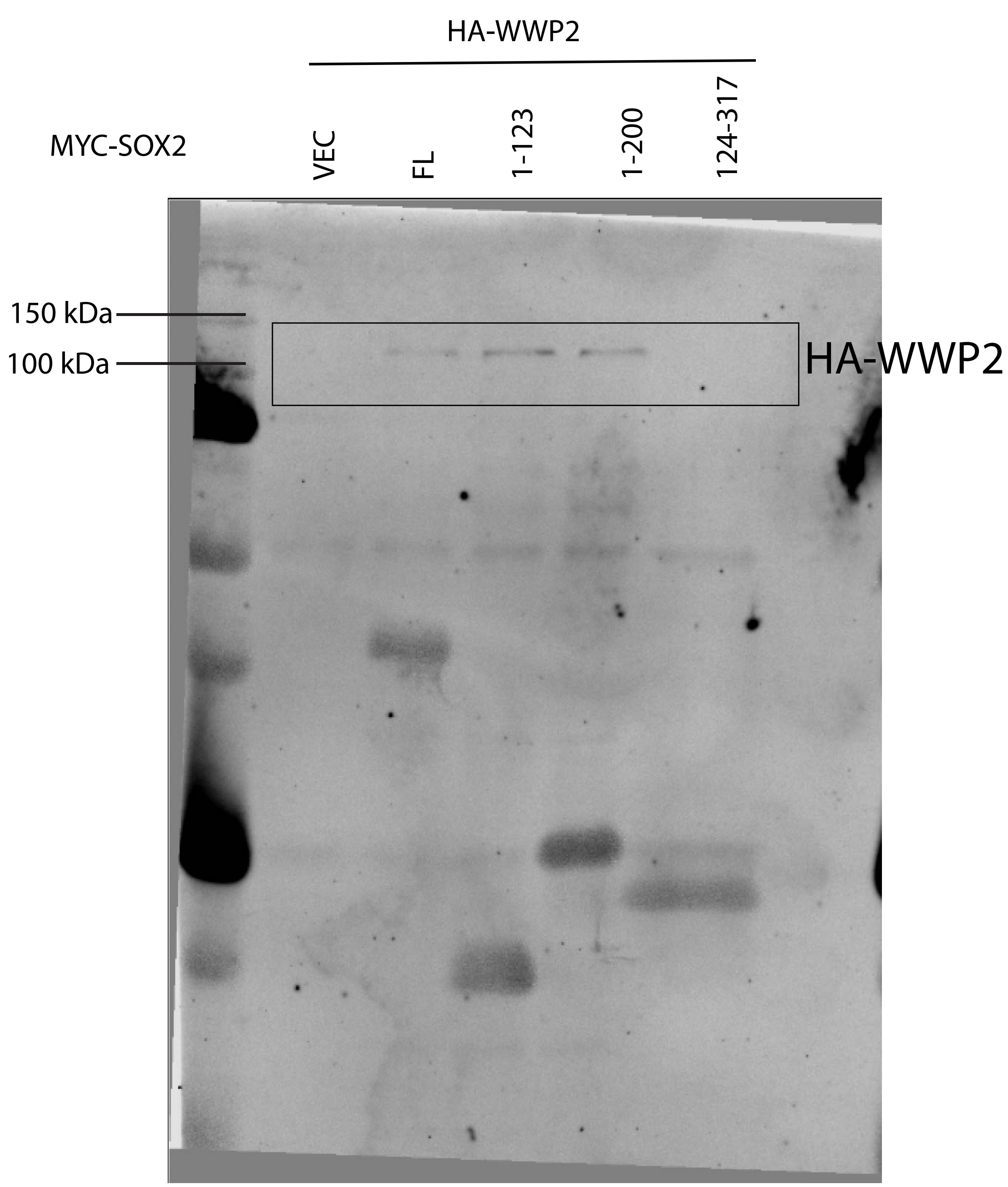

MYC IP BLOT

HA-TAG ANTIBODY

FIG 5B

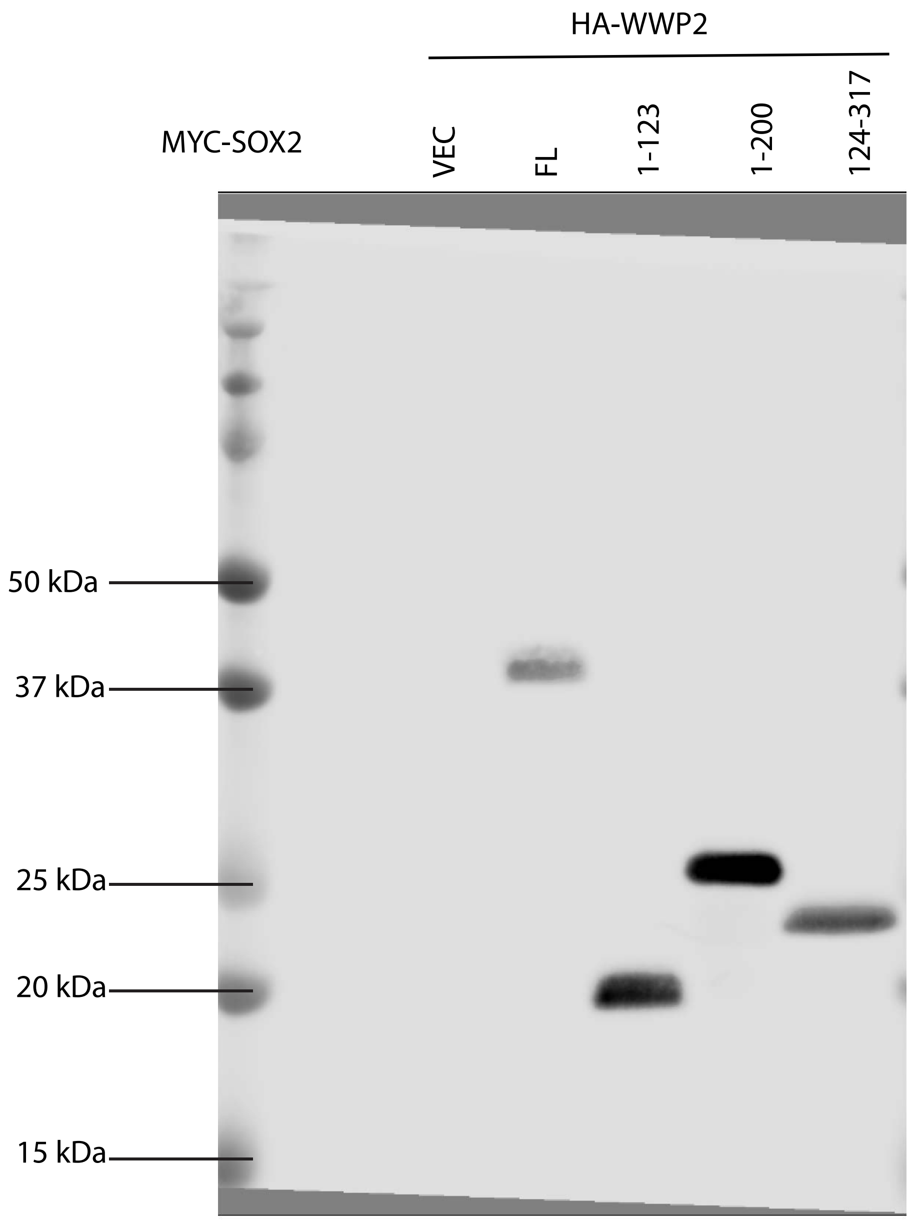

MYC IP BLOT

MYC-TAG ANTIBODY

FIG 5C

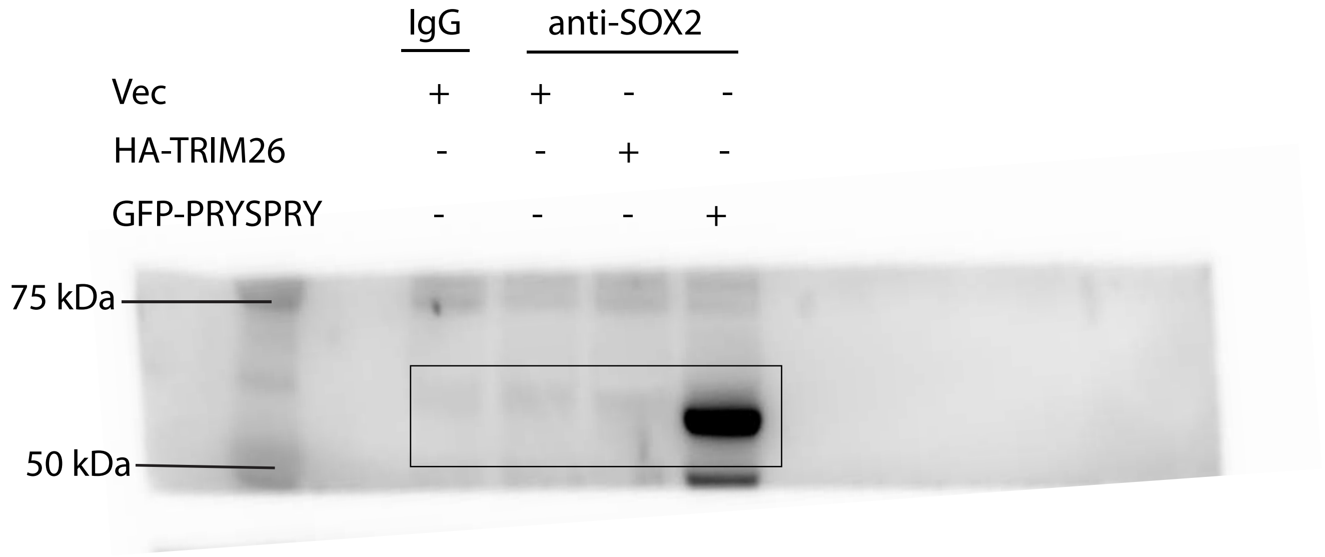

INPUT BLOT

GFP ANTIBODY

FIG 5C

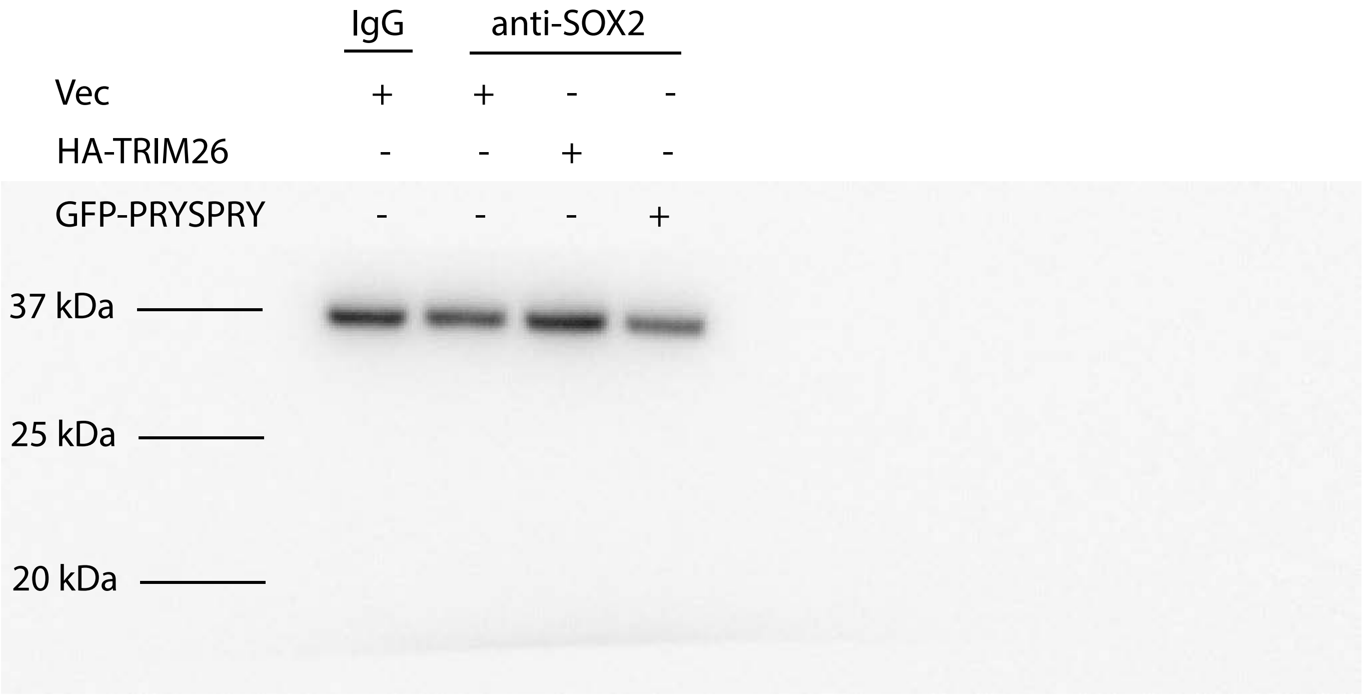

INPUT BLOT

SOX2 ANTIBODY

FIG 5C

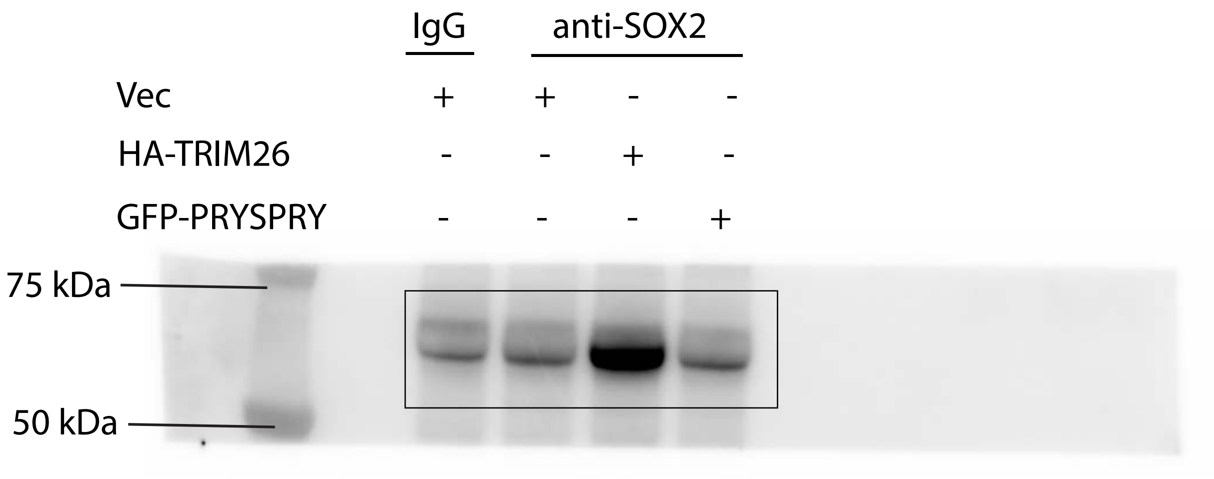

INPUT BLOT

TRIM26 ANTIBODY

FIG 5C

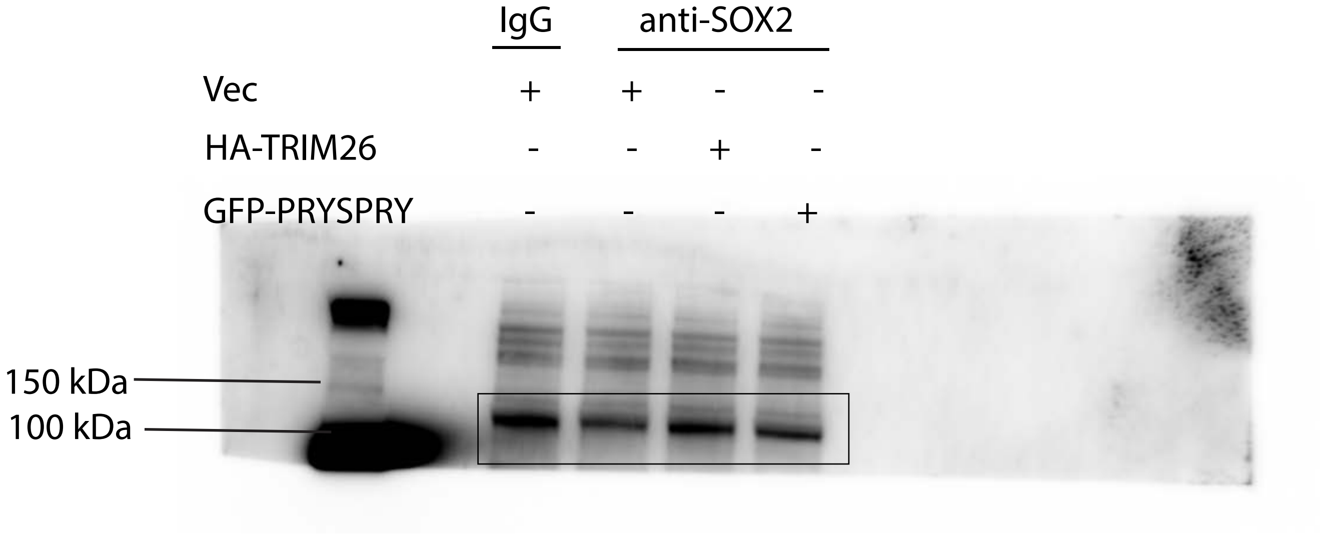

INPUT BLOT

WWP2 ANTIBODY

FIG 5C

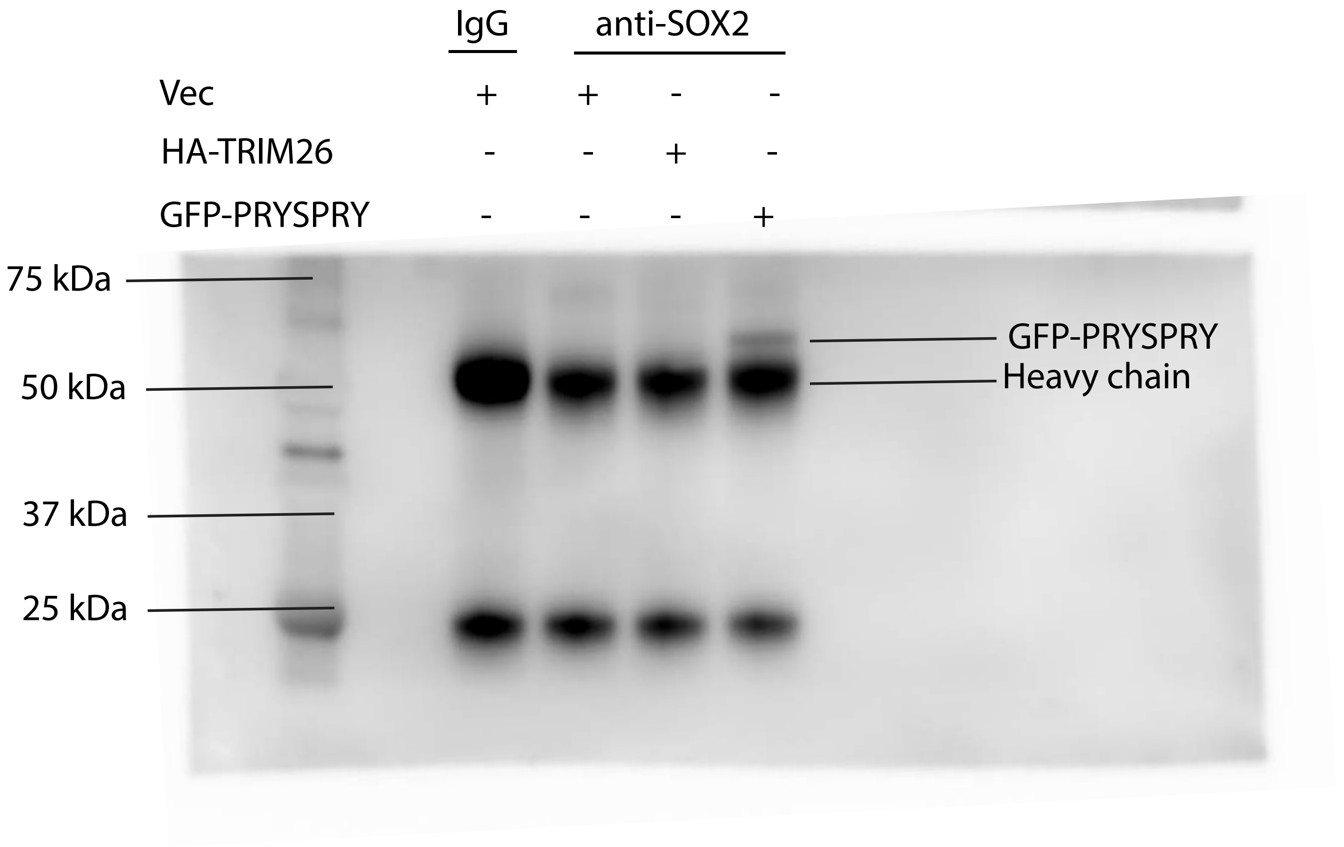

IP BLOT

GFP ANTIBODY

FIG 5C

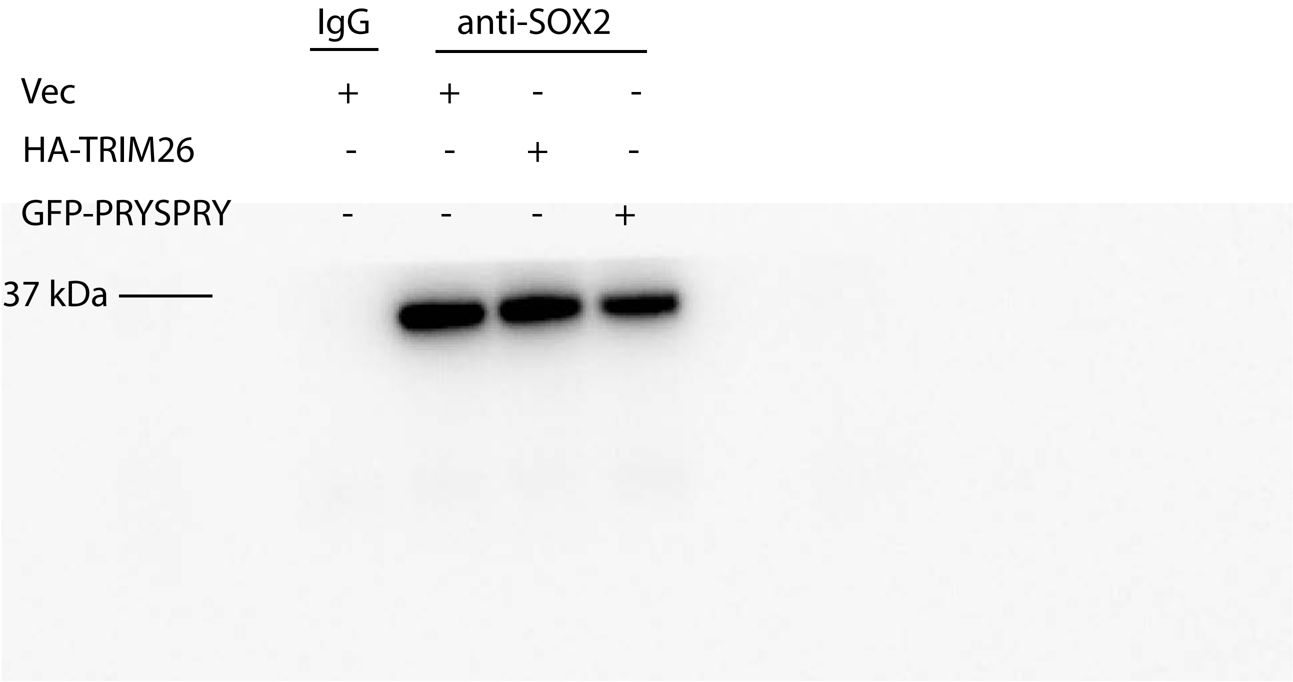

IP BLOT

SOX2 ANTIBODY

FIG 5C

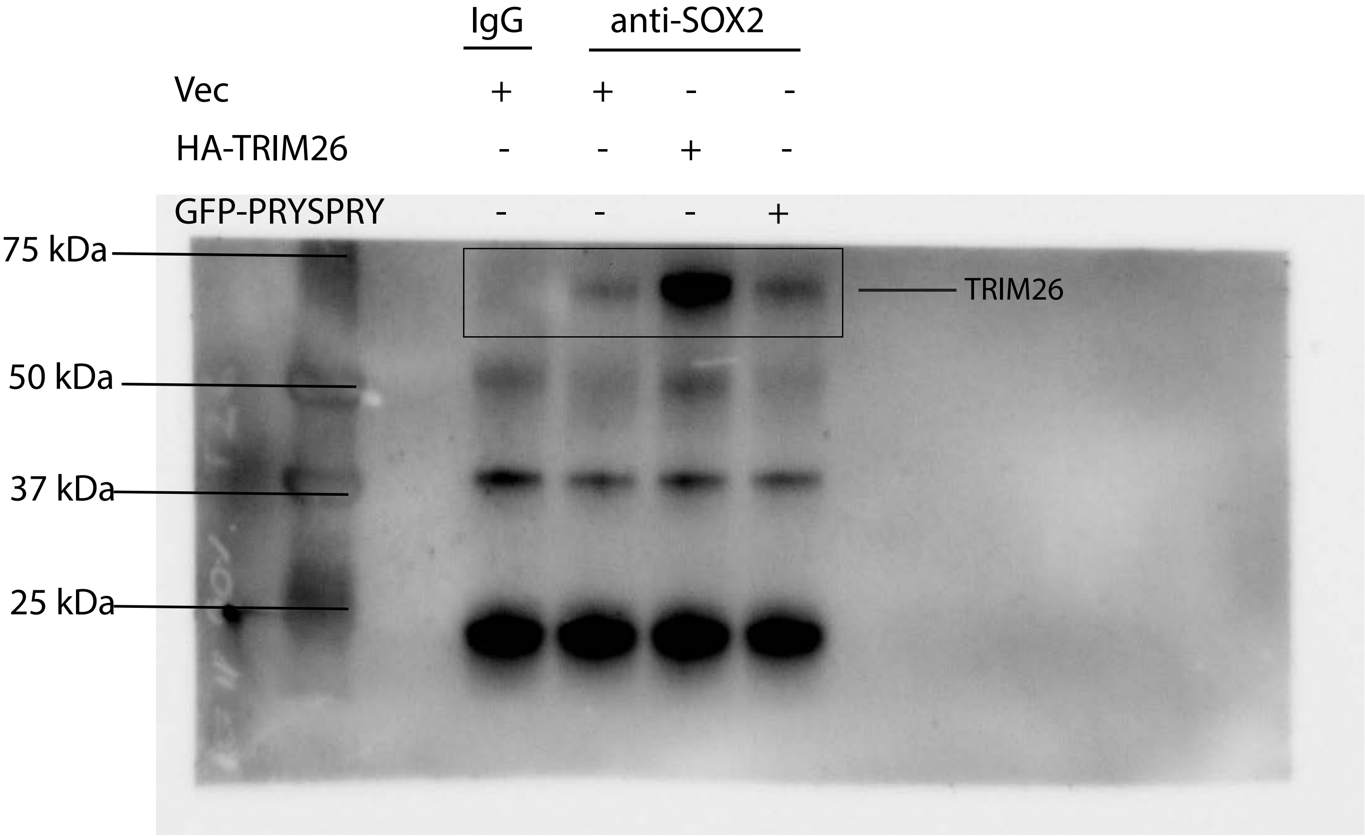

IP BLOT

TRIM26 ANTIBODY

FIG 5C

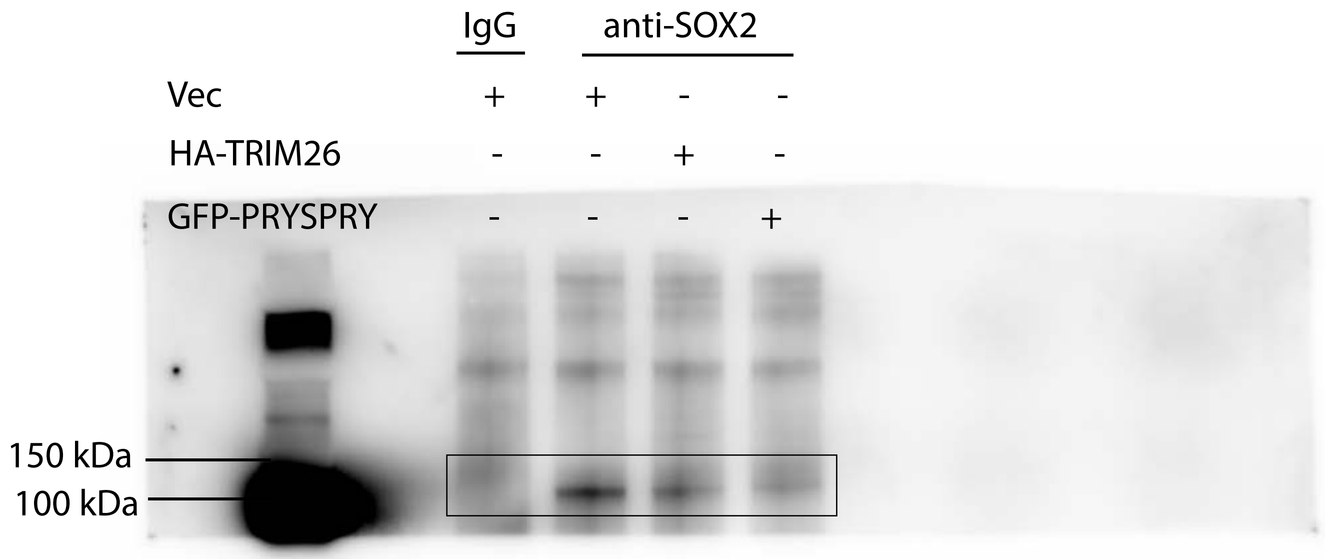

IP BLOT

WWP2 ANTIBODY

FIG 5D

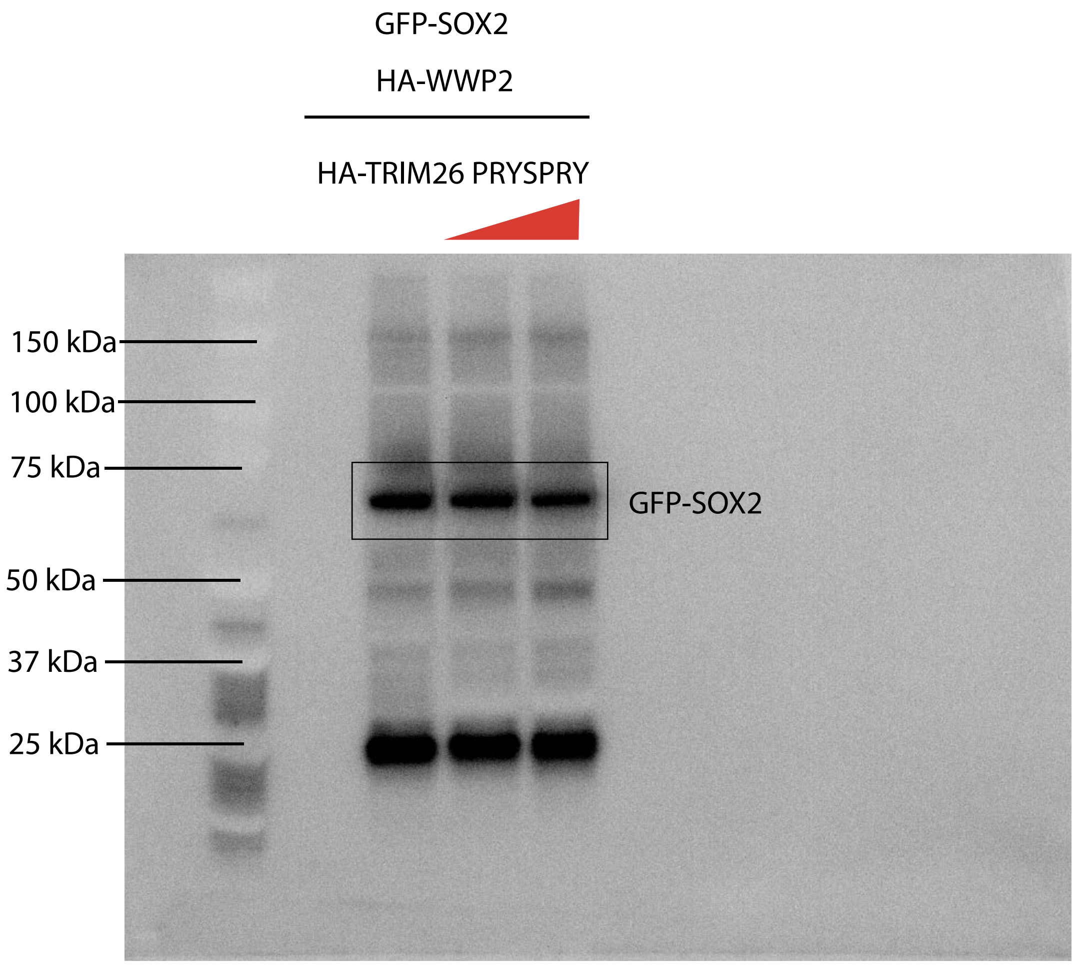

GFP ANTIBODY

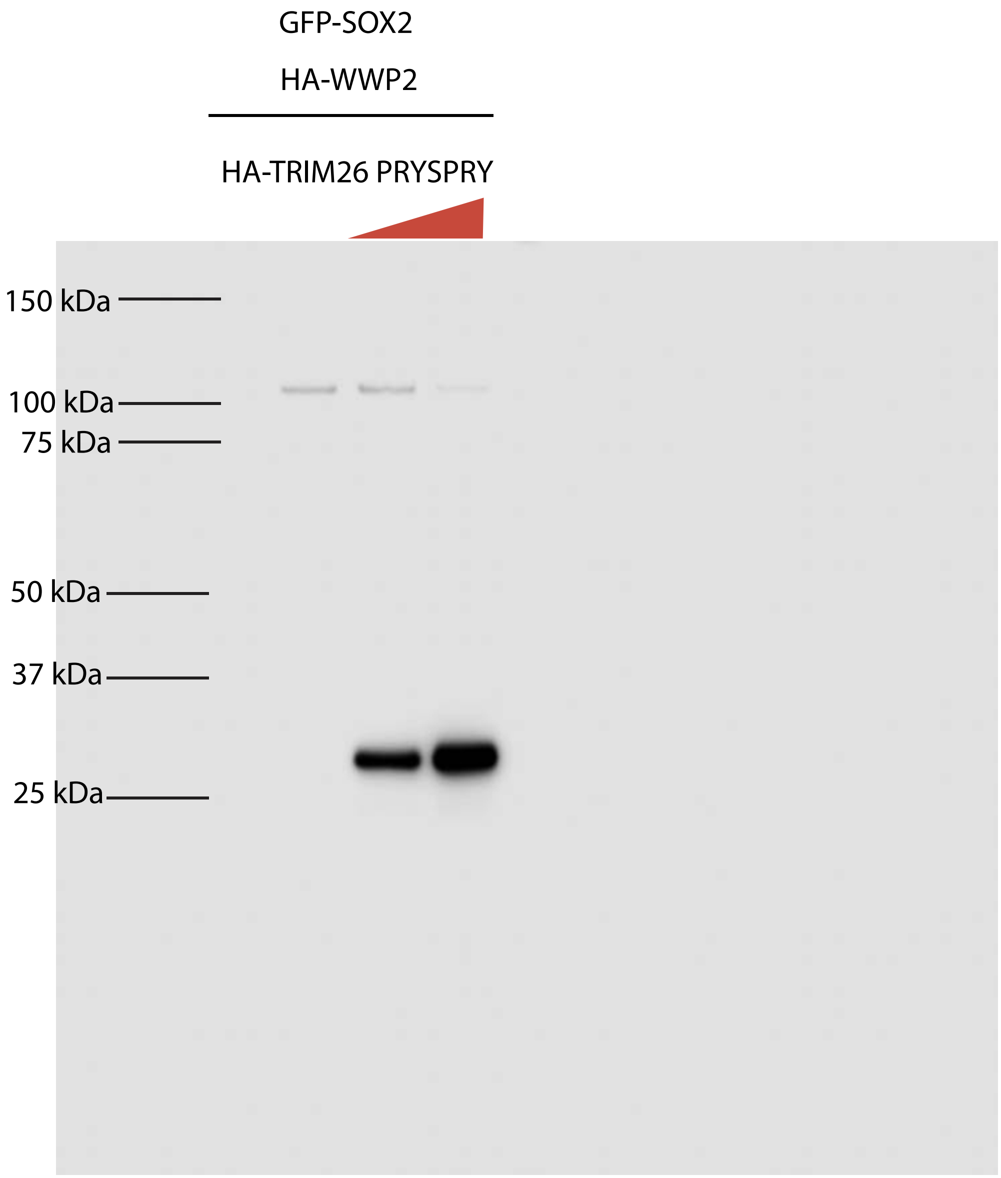

HA-TAG ANTIBODY

FIG 5E

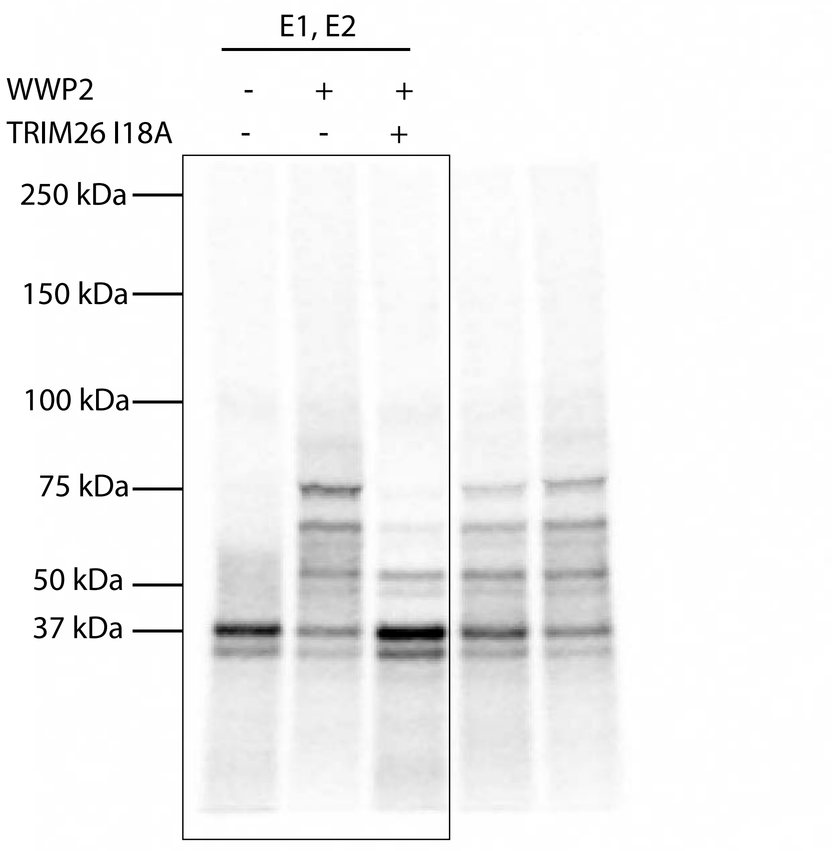

^{35}S AUTORADIOGRAPH

FIG 5F

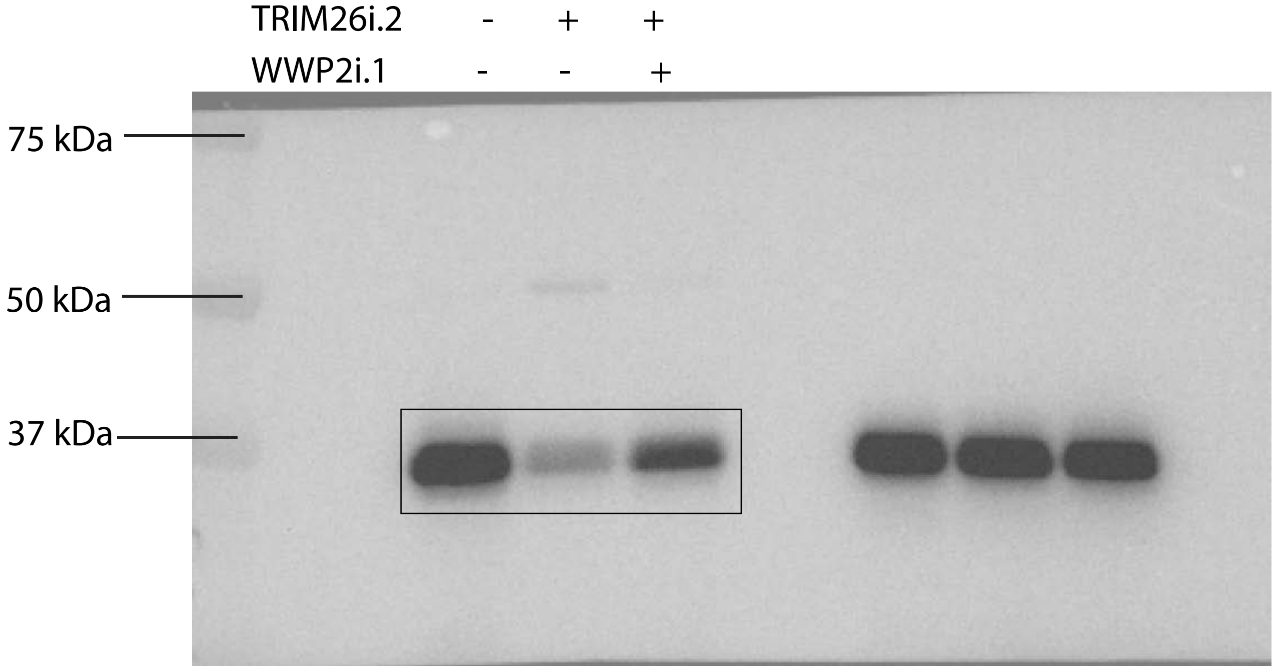

B67

SOX2 ANTIBODY

FIG 5F

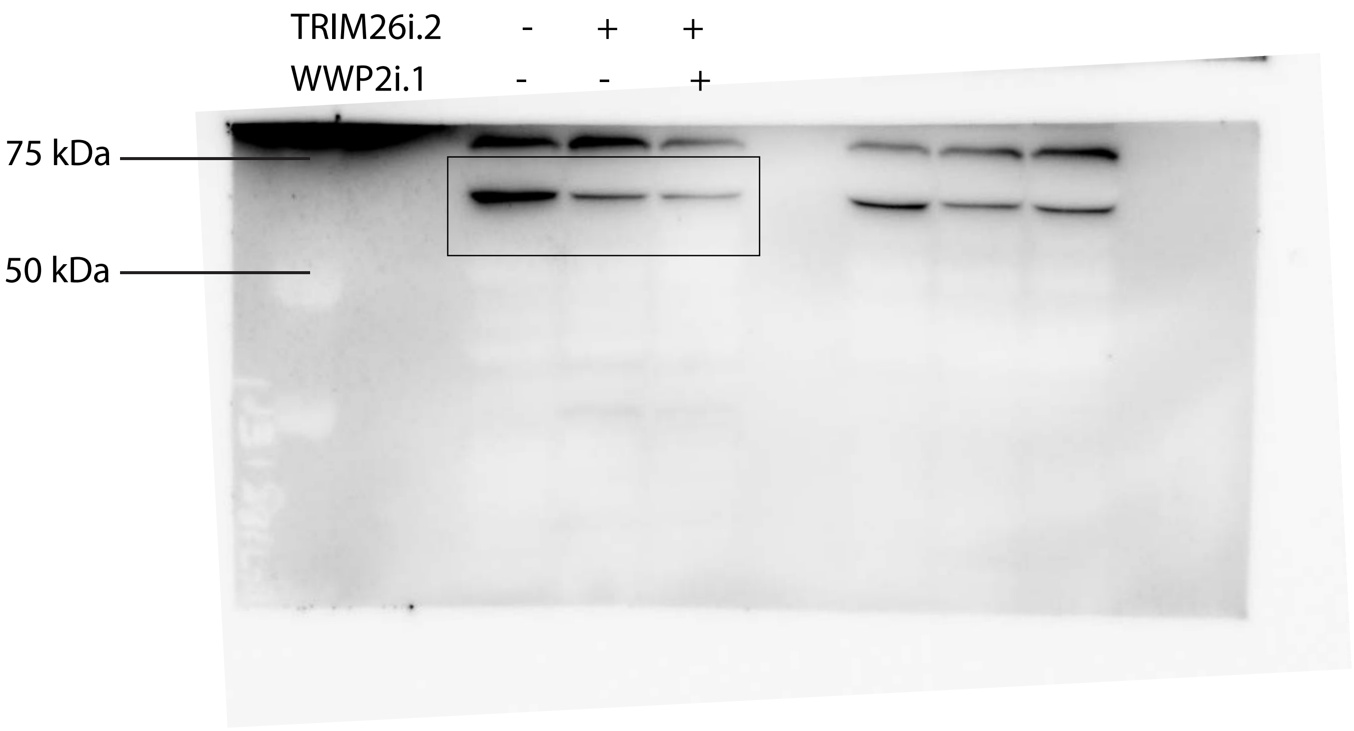

B67

TRIM26 ANTIBODY

FIG 5F

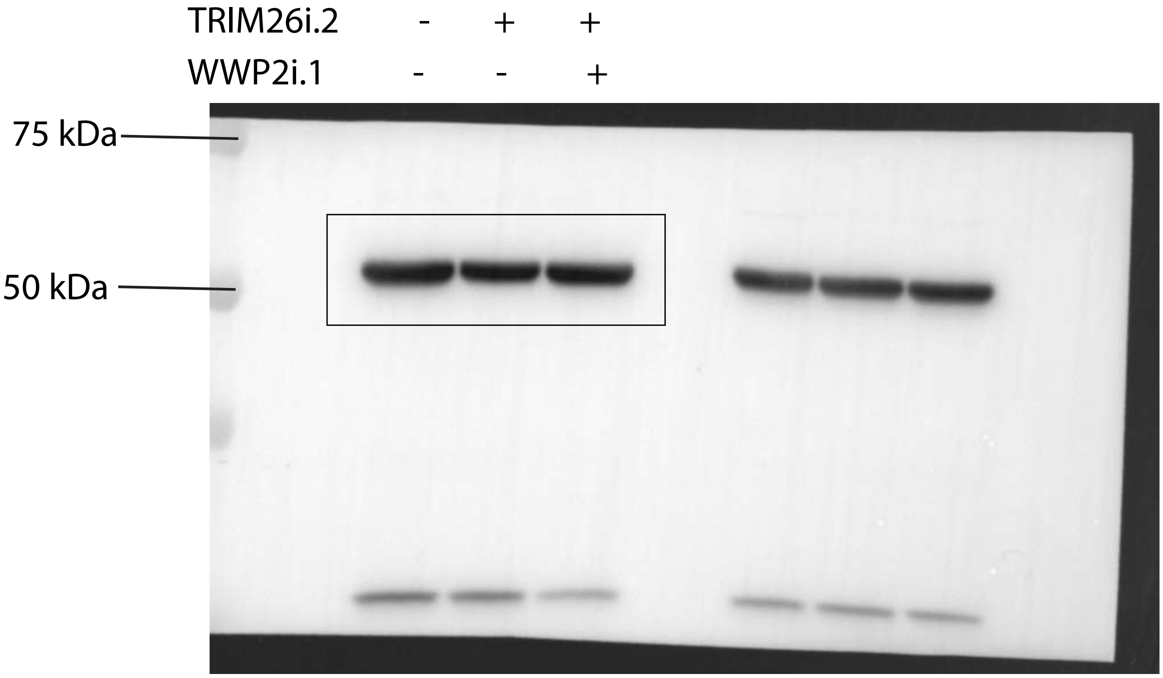

B67

TUBULIN ANTIBODY

FIG 5F

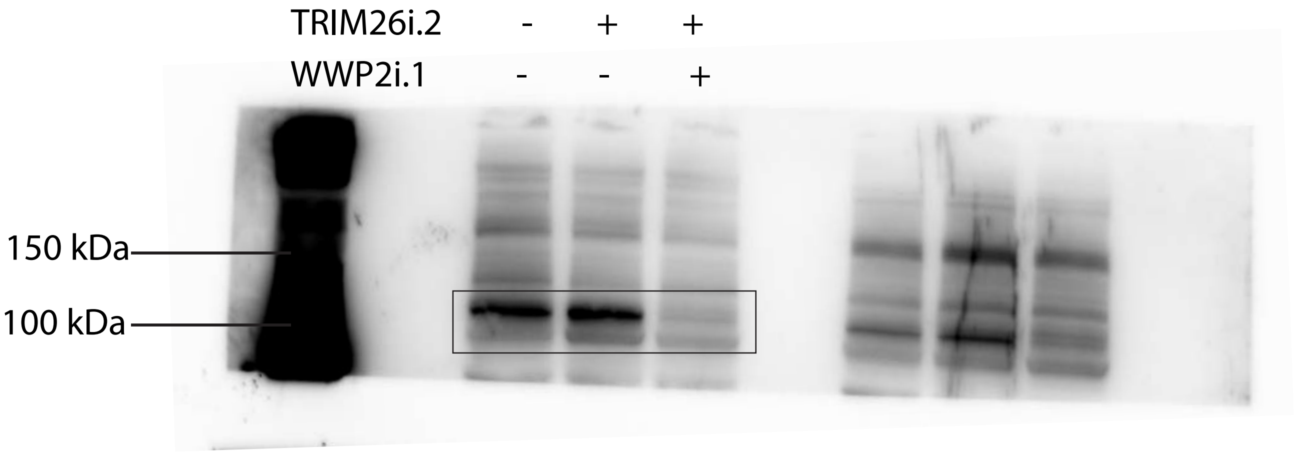

B67

WWP2 ANTIBODY

FIG 5F

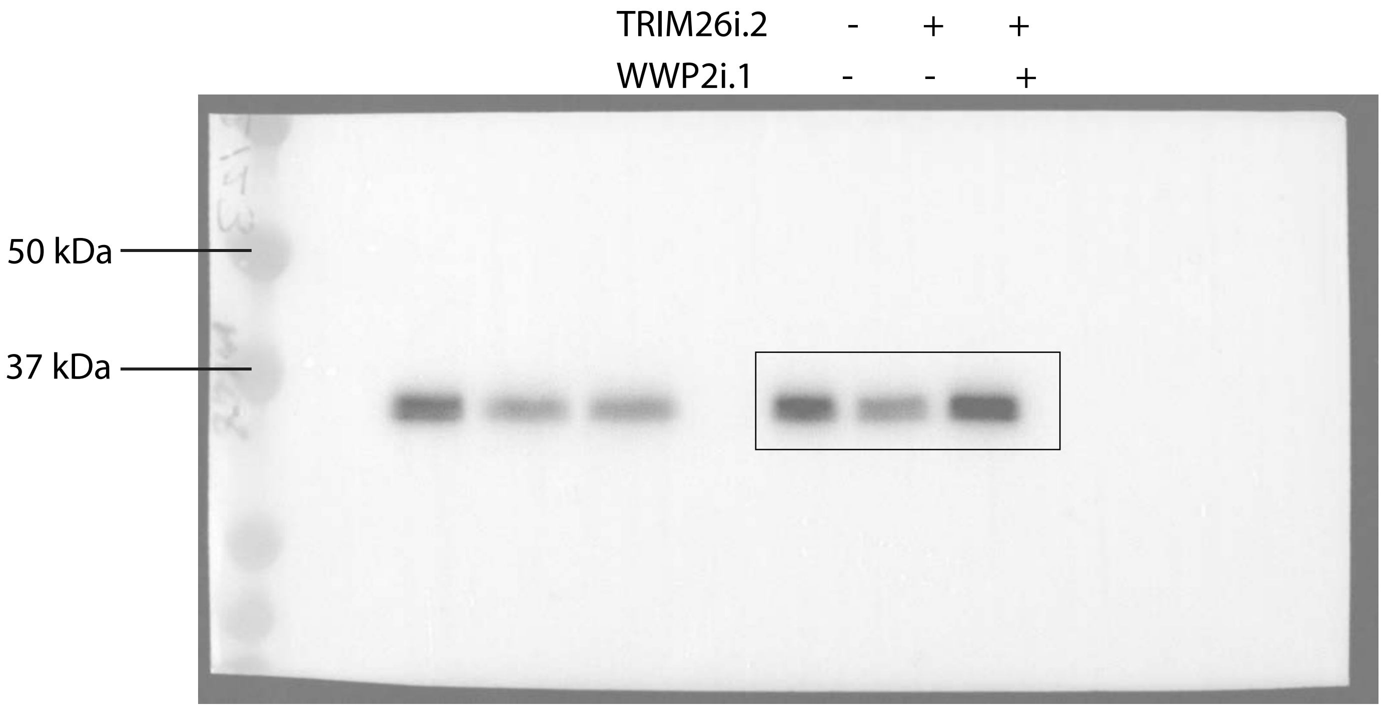

MGG8

SOX2 ANTIBODY

FIG 5F

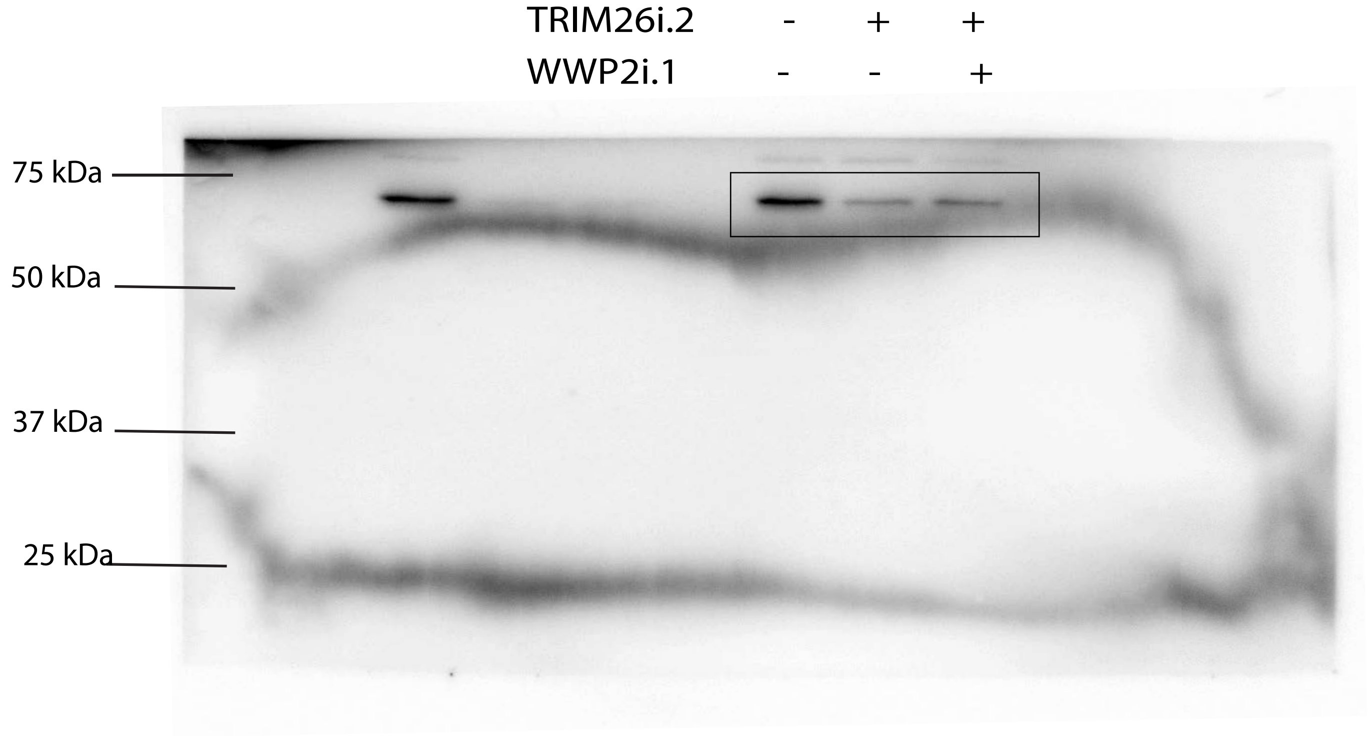

MGG8

TRIM26 ANTIBODY

FIG 5F

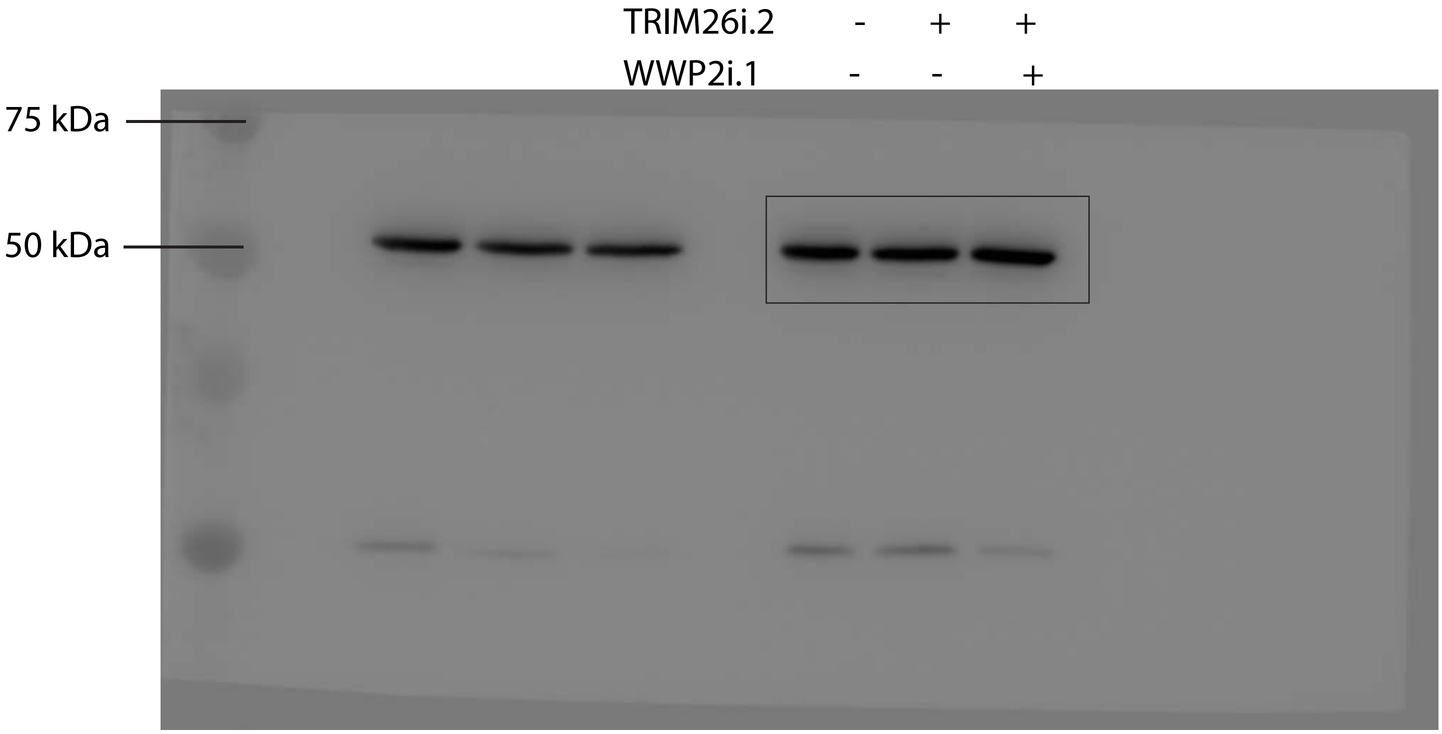

MGG8

TUBULIN ANTIBODY

FIG 5F

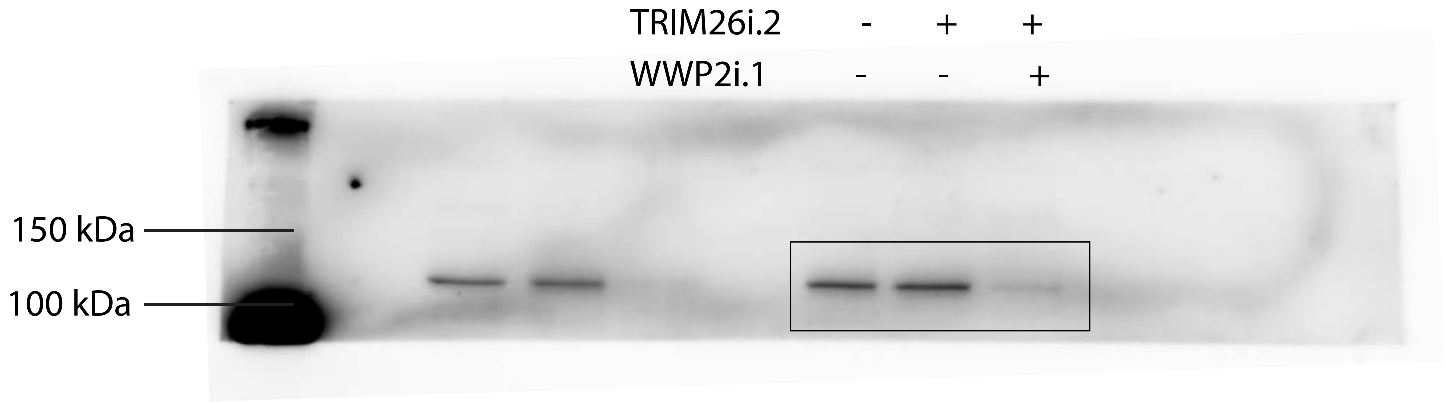

MGG8

WWP2 ANTIBODY

SUPPLEMENTARY FIG. 3A

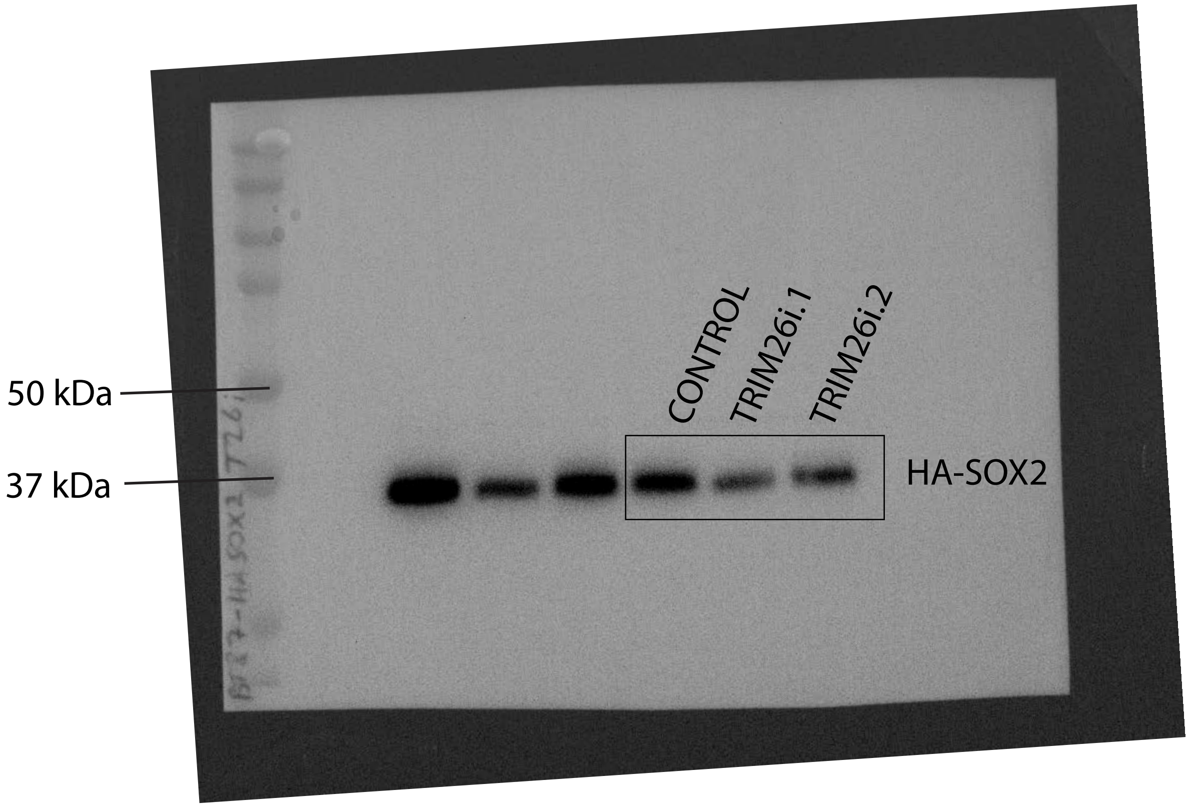

BT87-HA-SOX2 STABLE LINE

HA-TAG ANTIBODY

SUPPLEMENTARY FIG. 3A

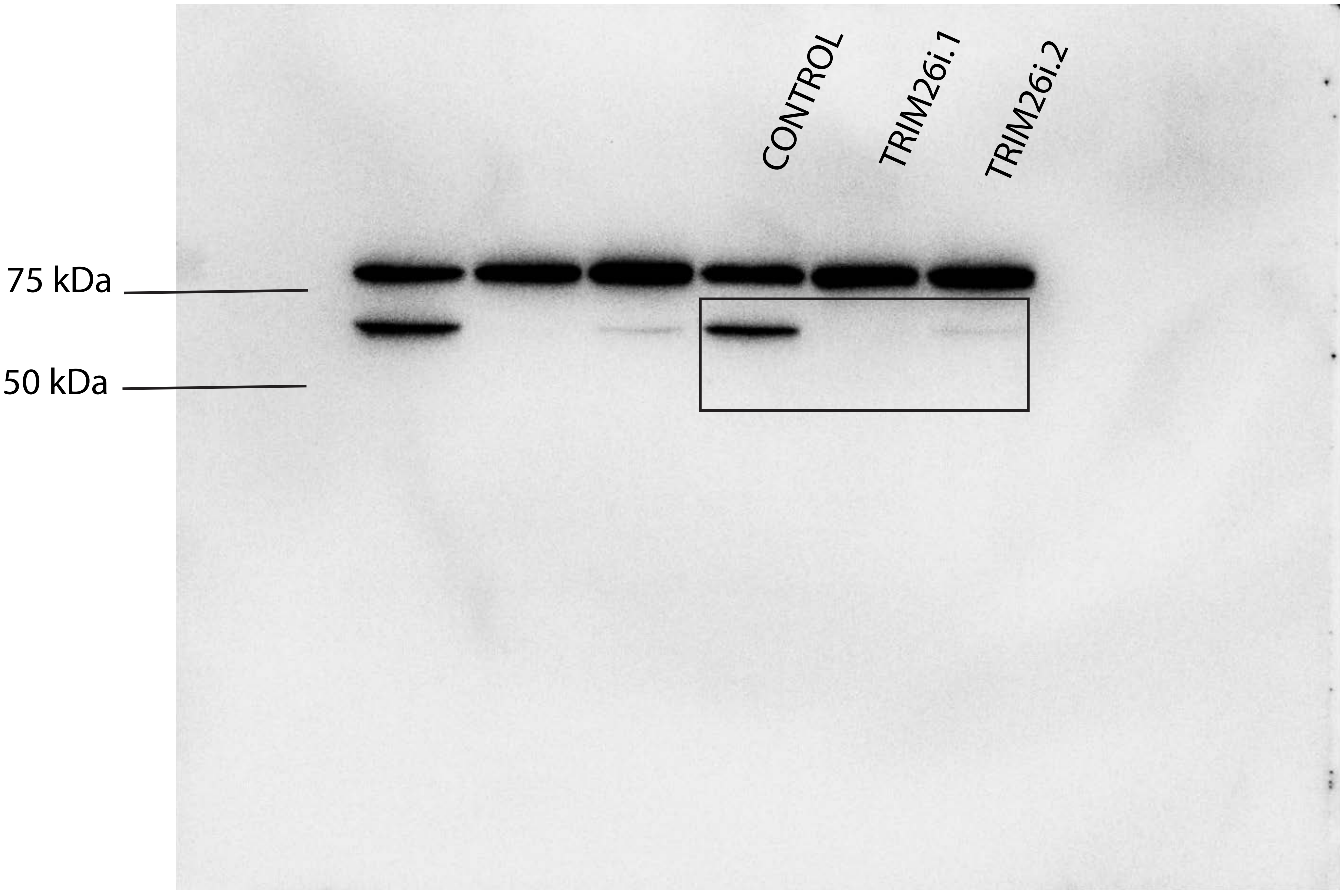

BT87-HA-SOX2 STABLE LINE

TRIM26 ANTIBODY

SUPPLEMENTARY FIG. 3A

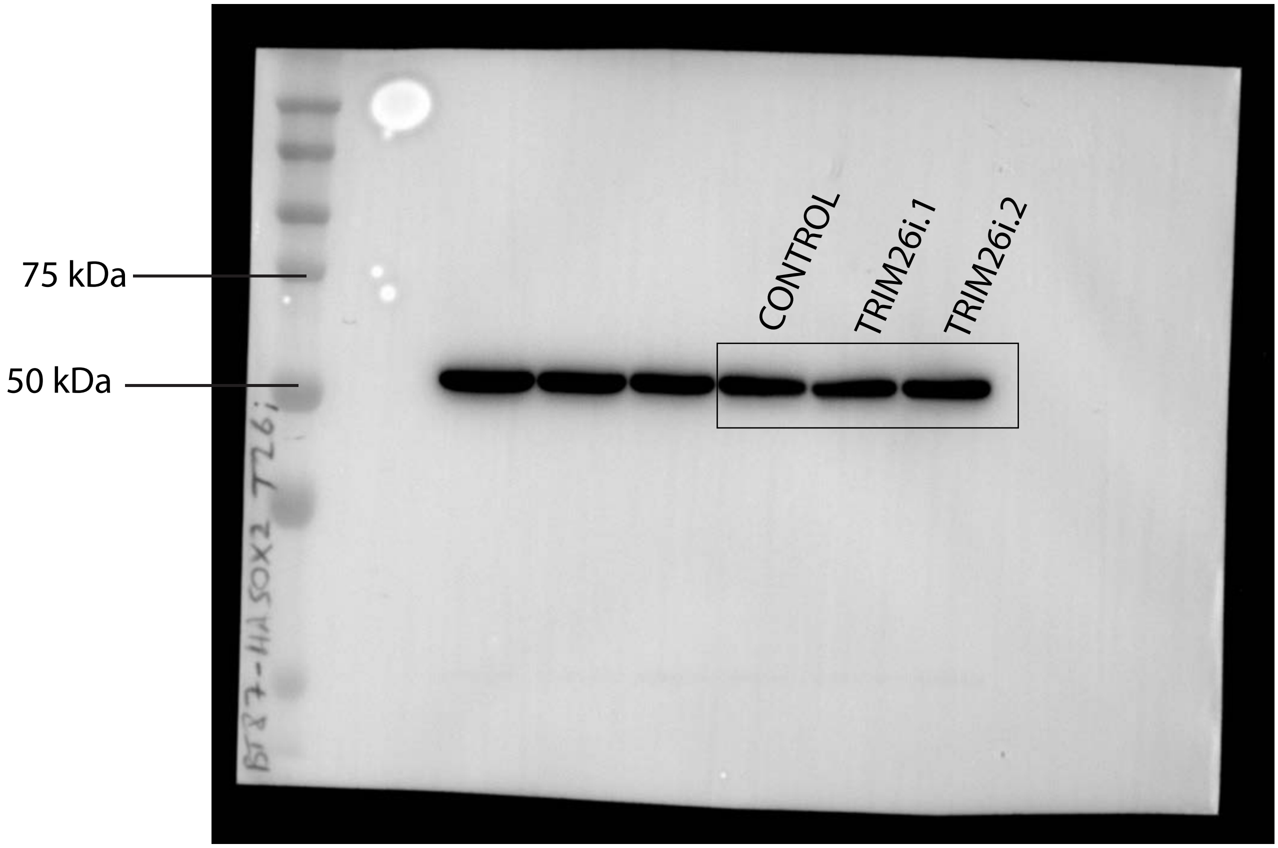

BT87-HA-SOX2 STABLE LINE

TUBULIN ANTIBODY

SUPPLEMENTARY FIG. 3B

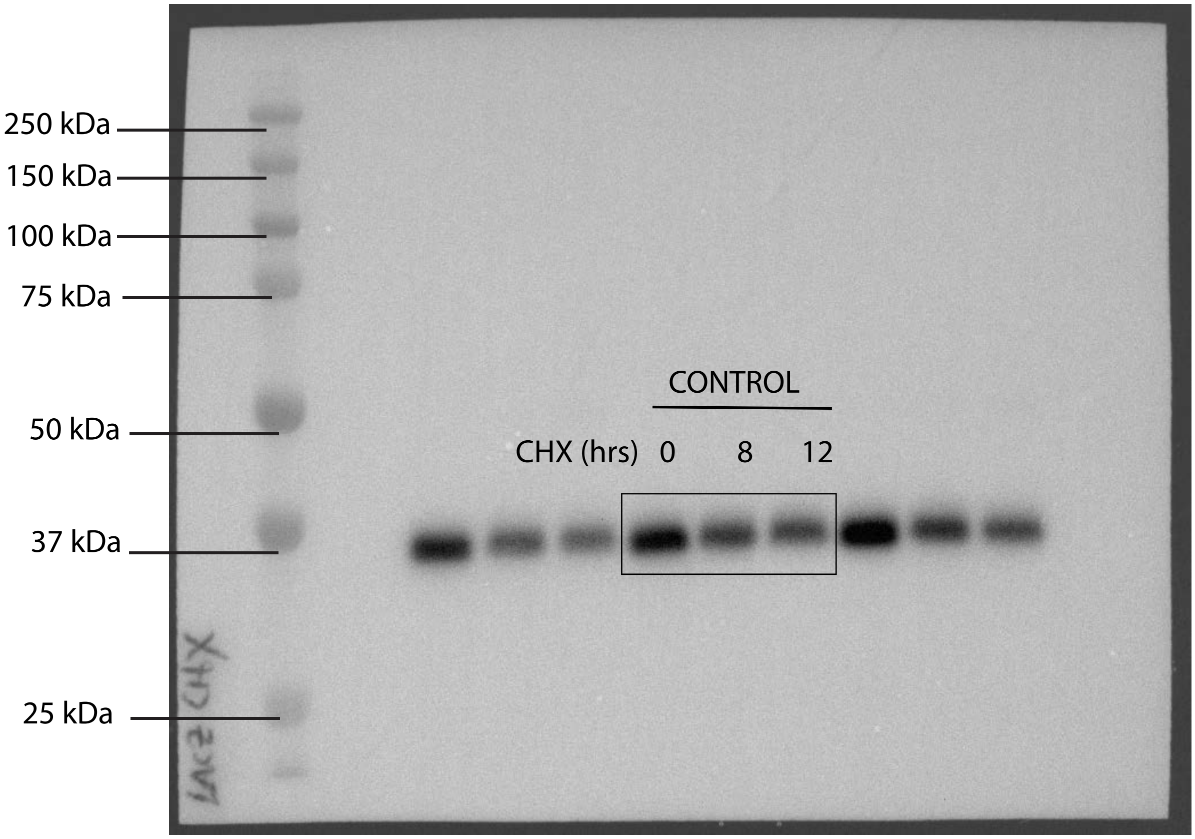

B67 CONTROL

SOX2 ANTIBODY

SUPPLEMENTARY FIG. 3B

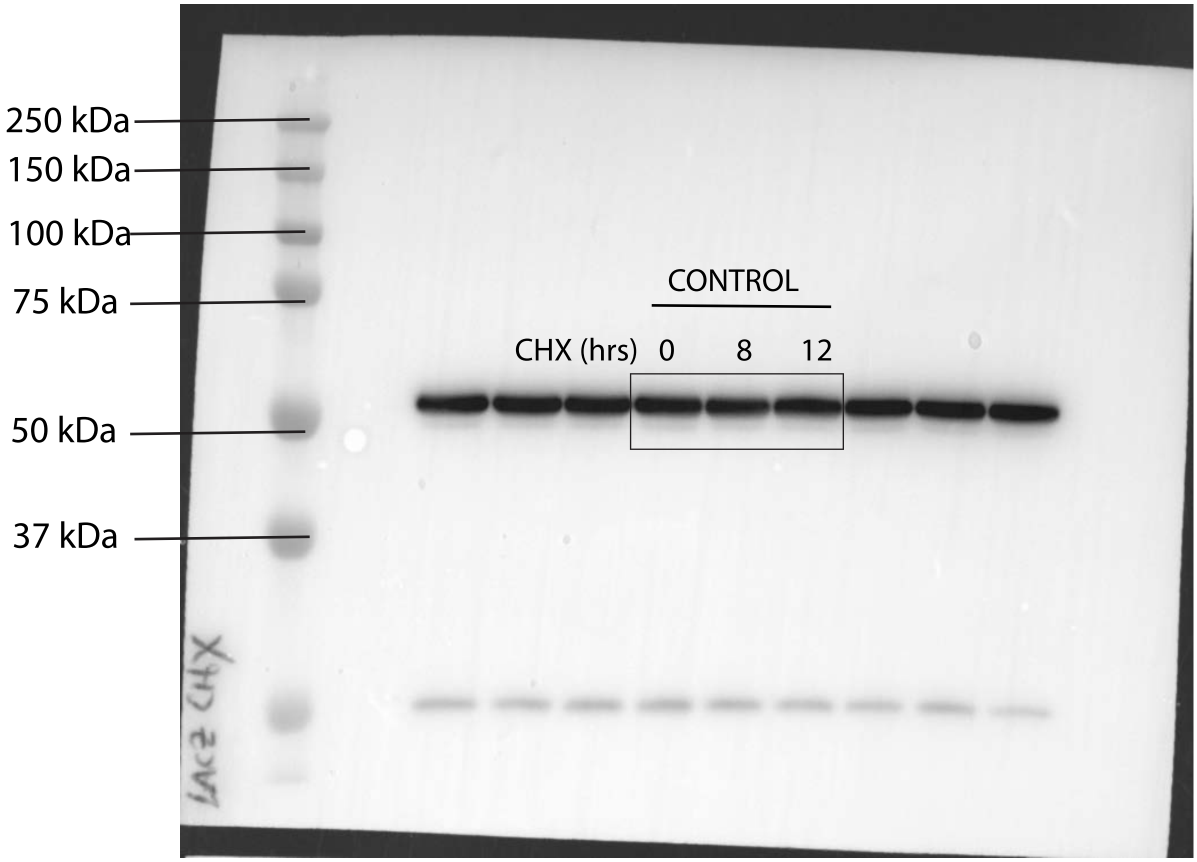

B67 CONTROL

TUBULIN ANTIBODY

SUPPLEMENTARY FIG. 3B

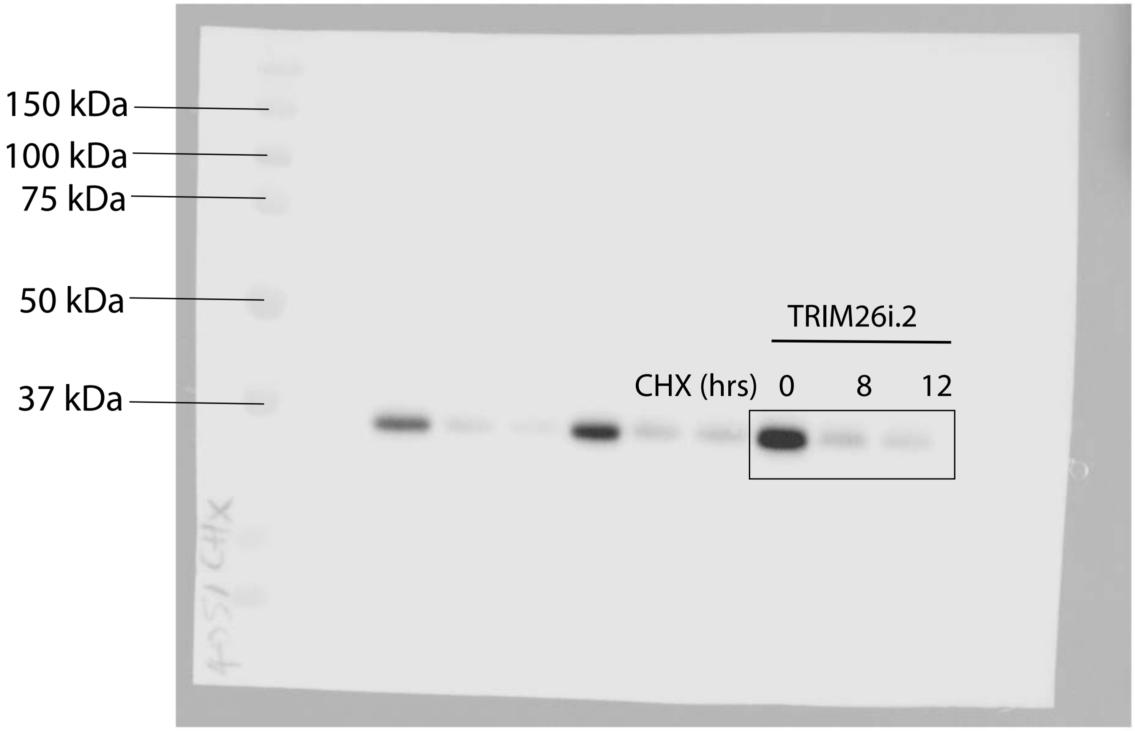

B67 TRIM26i.2

SOX2 ANTIBODY

SUPPLEMENTARY FIG. 3B

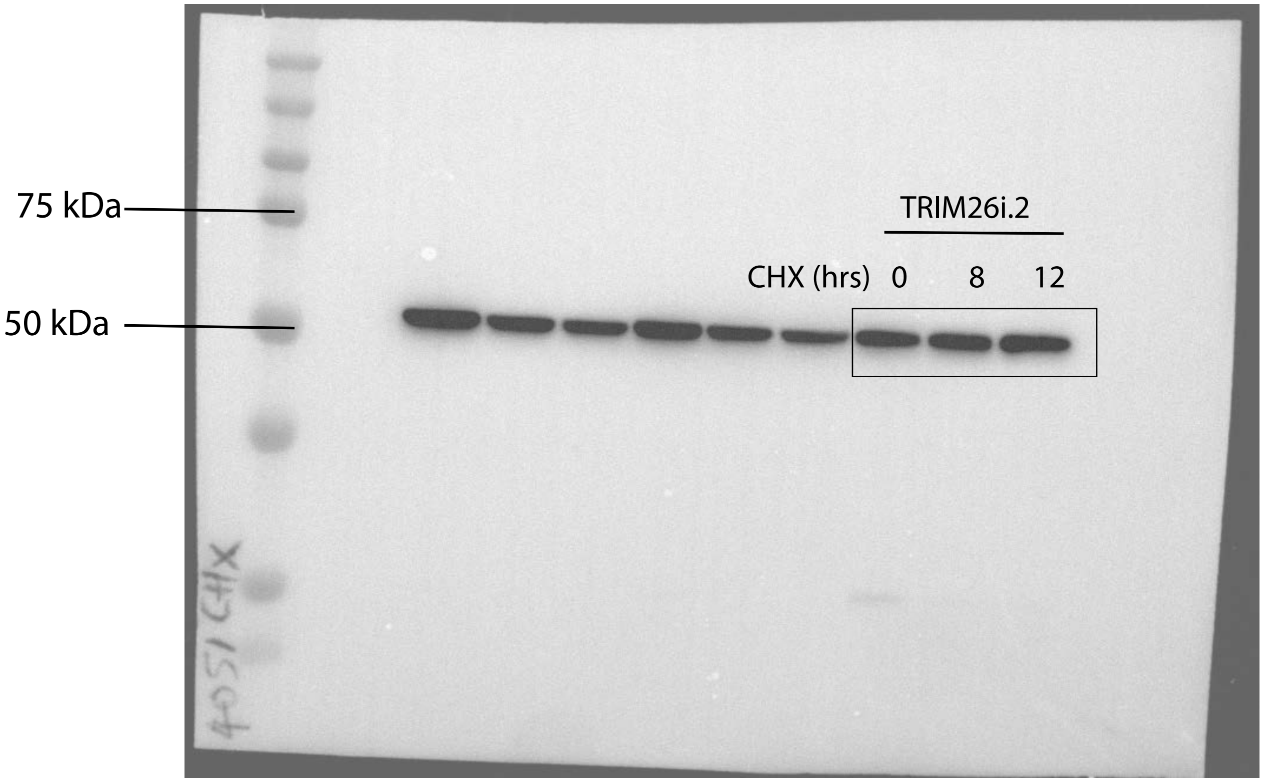

B67 TRIM26i.2

TUBULIN ANTIBODY

SUPPLEMENTARY FIG. 4E

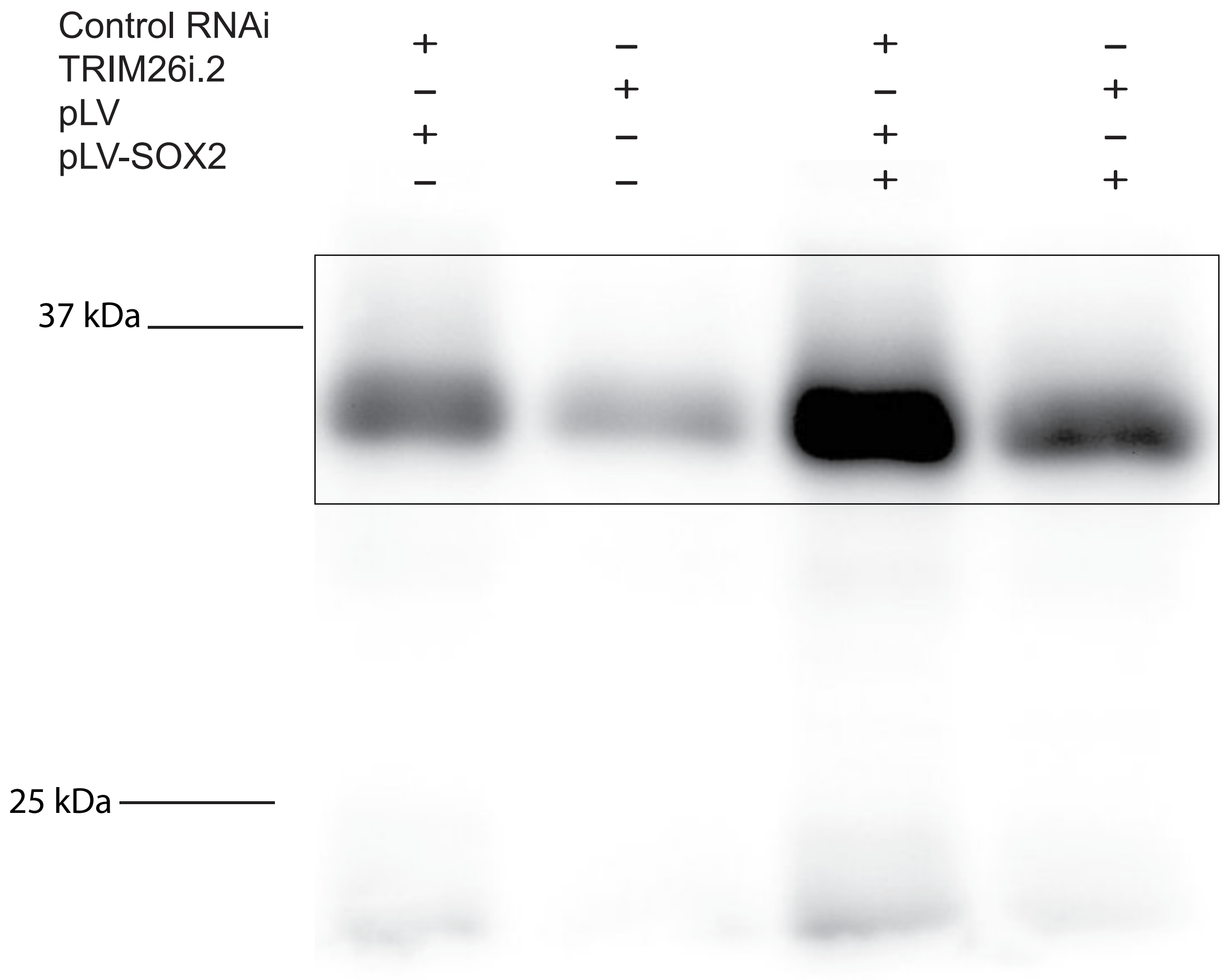

B67

SOX2 ANTIBODY

SUPPLEMENTARY FIG. 4E

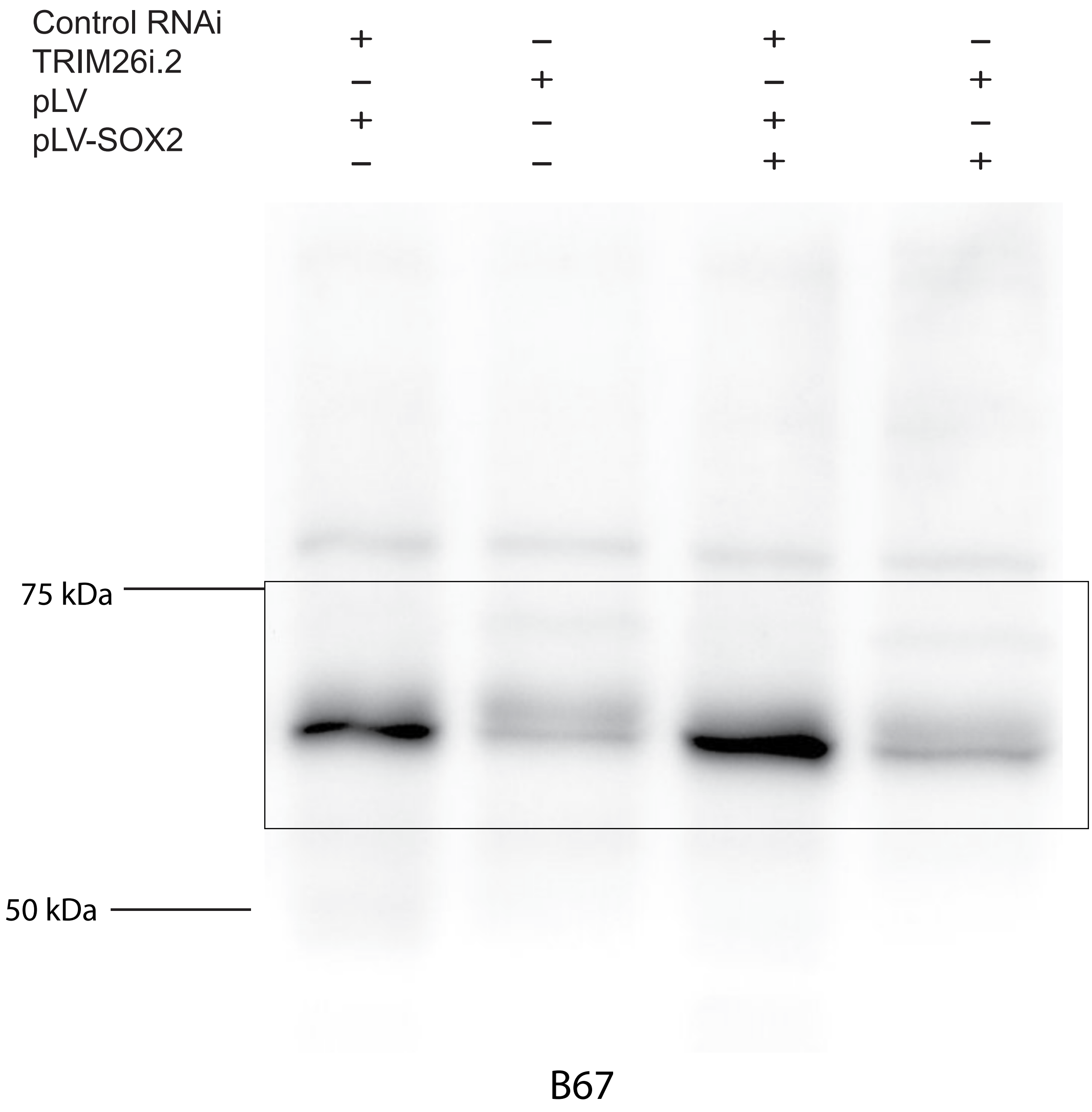

TRIM26 ANTIBODY

SUPPLEMENTARY FIG. 4E

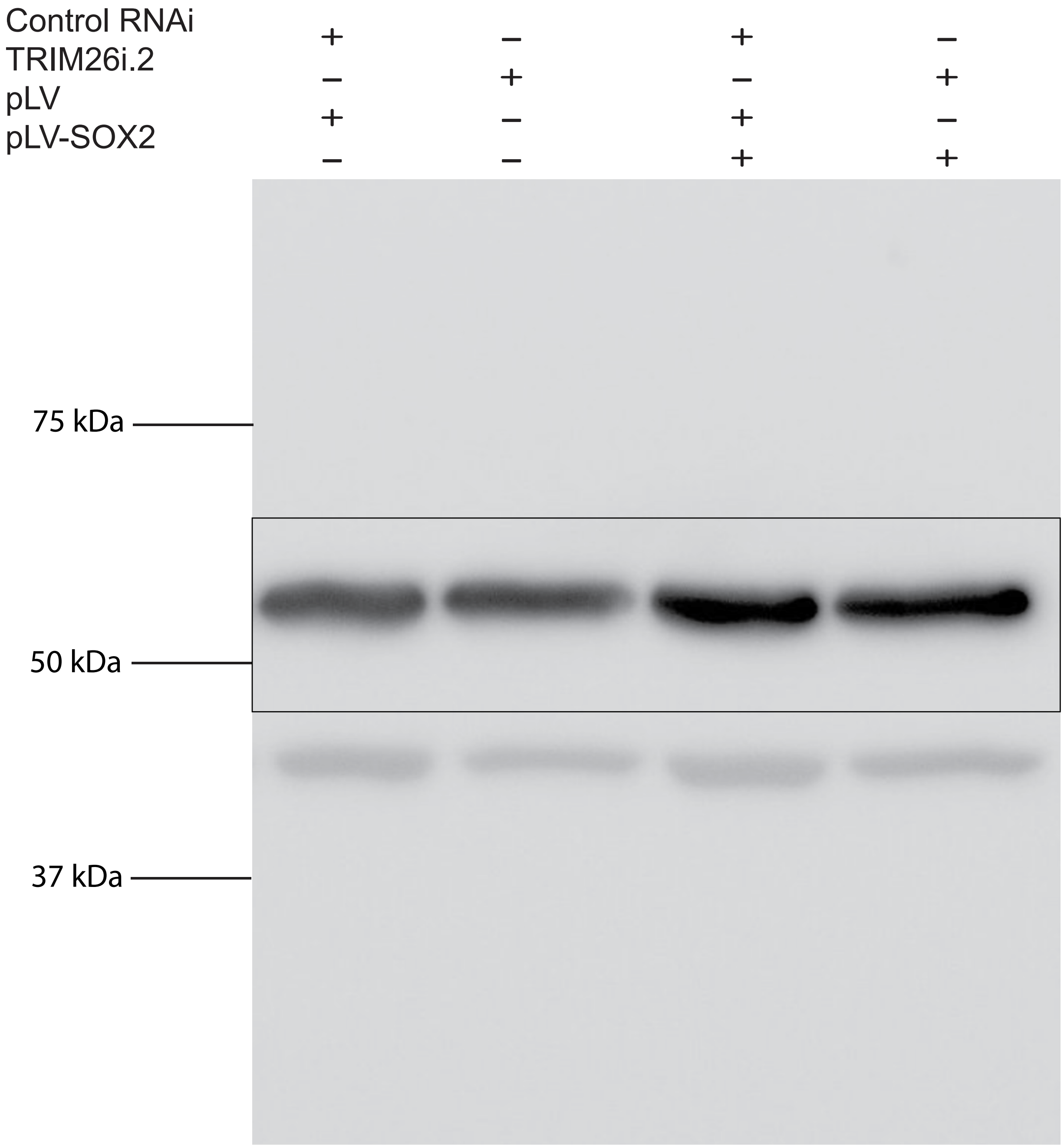

B67

TUBULIN ANTIBODY

SUPPLEMENTARY FIG. 5C

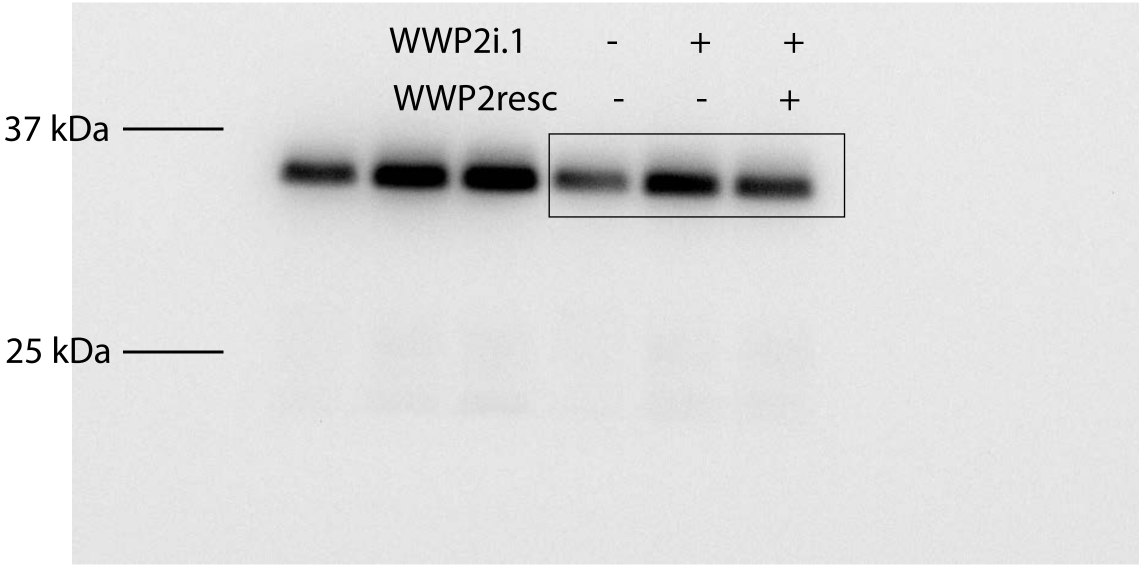

B67

SOX2 ANTIBODY

SUPPLEMENTARY FIG. 5C

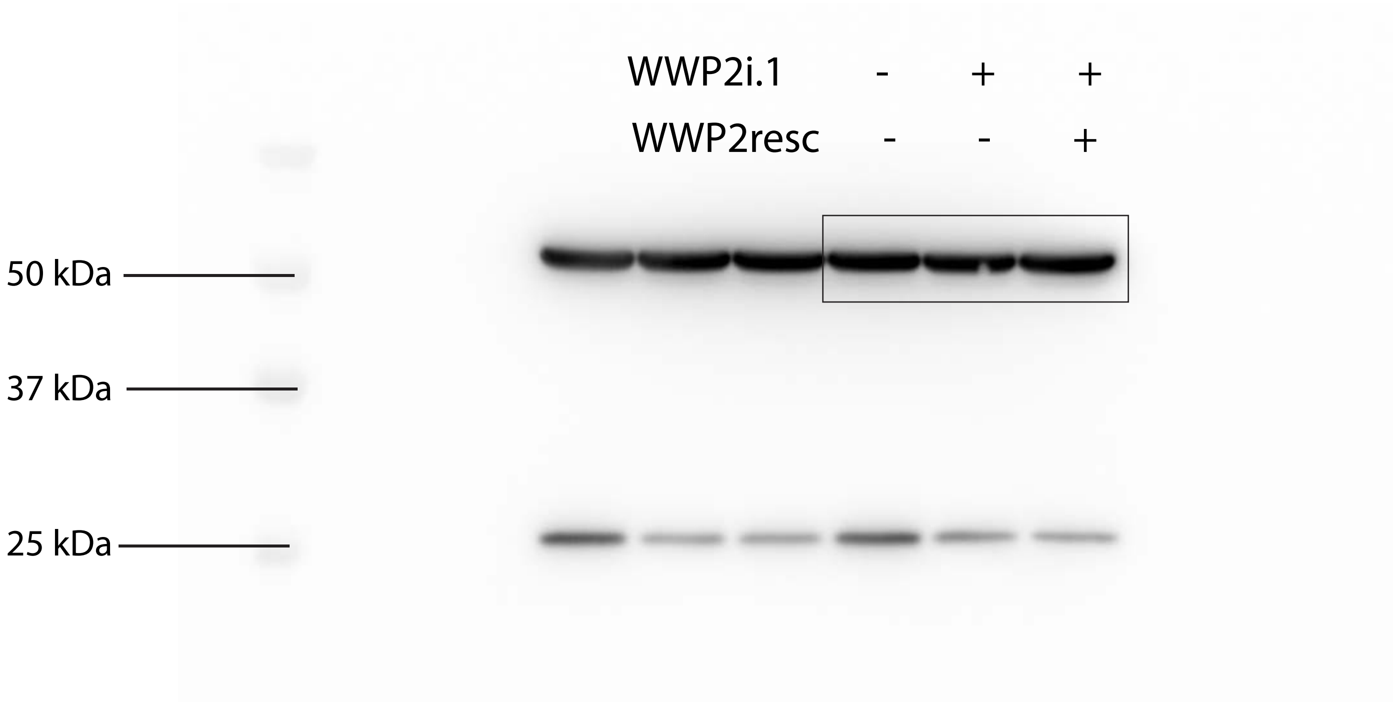

B67

TUBULIN ANTIBODY

SUPPLEMENTARY FIG. 5C

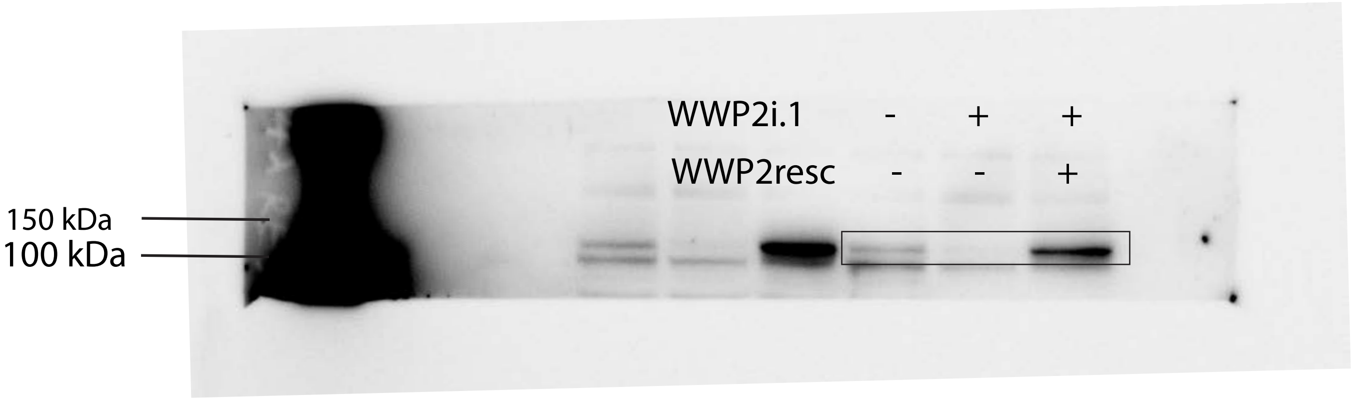

B67

WWP2 ANTIBODY
